# Lasp1 regulates adherens junction dynamics and fibroblast transformation in destructive arthritis

**DOI:** 10.1038/s41467-021-23706-8

**Published:** 2021-06-15

**Authors:** Denise Beckmann, Anja Römer-Hillmann, Annika Krause, Uwe Hansen, Corinna Wehmeyer, Johanna Intemann, David J. J. de Gorter, Berno Dankbar, Jan Hillen, Marianne Heitzmann, Isabell Begemann, Milos Galic, Toni Weinhage, Dirk Foell, Rizi Ai, Joachim Kremerskothen, Hans P. Kiener, Sylvia Müller, Thomas Kamradt, Christopher Schröder, Elsa Leitão, Bernhard Horsthemke, Philip Rosenstiel, Karl Nordström, Gilles Gasparoni, Nina Gasparoni, Jörn Walter, Na Li, Xinyi Yang, Ho-Ryun Chung, Hermann Pavenstädt, Nico Lindemann, Hans J. Schnittler, Wei Wang, Gary S. Firestein, Thomas Pap, Adelheid Korb-Pap

**Affiliations:** 1 Institute of Musculoskeletal Medicine, University Hospital Münster, Albert-Schweitzer-Campus 1, Building D3, Münster, Germany; 2 Institute of Medical Physics and Biophysics, University of Münster, Robert-Koch Straße 31, Münster, Germany; 3 Department of Pediatric Rheumatology and Immunology, University Children’s Hospital Münster, Domagkstraße 3, Münster, Germany; 4 Department of Chemistry and Biochemistry, 9500 Gilman Drive, UC San Diego, La Jolla, CA USA; 5 Department of Nephrology and Rheumatology, Internal Medicine D, University Hospital Münster, Albert-Schweitzer-Campus 1, Münster, Germany; 6 Department of Medicine III, Division of Rheumatology, Medical University of Vienna, Waehringer Guertel 18-20, Vienna, Austria; 7 Institute for Immunology, Jena University Hospital, Friedrich Schiller University, Jena, Leutragraben 3, Jena, Germany; 8 Genome Informatics, Institute of Human Genetics, University of Duisburg-Essen, Virchowstraße 183, Essen, Germany; 9 Institute of Human Genetics, University Hospital of Essen, University of Duisburg-Essen, Hufelandstraße 55, Essen, Germany; 10 Institute of Clinical Molecular Biology, University of Kiel, Rosalind-Franklin-Straße 12, Kiel, Germany; 11 Department of Genetics/Epigenetics, Saarland University, Campus Saarbrücken, Building A2 4, Saarbrücken, Germany; 12 Max Planck Institute for Molecular Genetics, Otto-Warburg-Laboratories, Epigenomics, Ihnestraße 63-73, Berlin, Germany; 13 Institute of Medical Bioinformatics and Biostatistics, Philipps-University Marburg, Bunsenstraße 3, Marburg, Germany; 14 Institute of Anatomy and Vascular Biology, University of Münster, Vesaliusweg 2-4, Münster, Germany; 15 Department of Cellular and Molecular Medicine, 9500 Gilman Drive, UCSD School of Medicine, La Jolla, CA USA; 16 Division of Rheumatology, Allergy and Immunology, 9500 Gilman Drive, UCSD School of Medicine, La Jolla, CA USA

**Keywords:** Cell migration, Cytoskeleton, Rheumatoid arthritis

## Abstract

The LIM and SH3 domain protein 1 (Lasp1) was originally cloned from metastatic breast cancer and characterised as an adaptor molecule associated with tumourigenesis and cancer cell invasion. However, the regulation of Lasp1 and its function in the aggressive transformation of cells is unclear. Here we use integrative epigenomic profiling of invasive fibroblast-like synoviocytes (FLS) from patients with rheumatoid arthritis (RA) and from mouse models of the disease, to identify Lasp1 as an epigenomically co-modified region in chronic inflammatory arthritis and a functionally important binding partner of the Cadherin-11/β-Catenin complex in zipper-like cell-to-cell contacts. In vitro, loss or blocking of Lasp1 alters pathological tissue formation, migratory behaviour and platelet-derived growth factor response of arthritic FLS. In arthritic human TNF transgenic mice, deletion of *Lasp1* reduces arthritic joint destruction. Therefore, we show a function of Lasp1 in cellular junction formation and inflammatory tissue remodelling and identify Lasp1 as a potential target for treating inflammatory joint disorders associated with aggressive cellular transformation.

## Abstract (web summary)

Fibroblast-like synoviocytes are important mediators of joint pathology in rheumatoid arthritis (RA). Here the authors show that Lasp1 is epigenetically regulated and highly expressed by these cells in RA and its deletion can limit joint pathology in a mouse model of inflammatory arthritis.

## Introduction

Rheumatoid arthritis (RA) is a chronic autoimmune disease that mainly affects the joints and may lead to the progressive destruction of articular structures, particularly cartilage and bone^1,2^. Despite major advances in the therapy of RA, a substantial proportion of patients have limited treatment responses, presenting with persistent inflammation and progressive joint destruction. Fibroblast-like synoviocytes (FLS) are important effector cells in RA and contribute prominently to the transformation of the thin synovial membrane into an inflamed, hyperplastic and aggressive tissue that grows into adjacent joint structures. Growing evidence suggests that very early in the course of disease, RA-FLS undergo a transformation and acquire an autonomously aggressive phenotype that is maintained in these cells even in the absence of continuous inflammatory stimulation^3^. This phenotype is characterised by a high degree of invasiveness particularly towards articular cartilage, which has been associated with their ability to form invadopodia-like structures and their secretion of matrix degrading enzymes such as matrix metalloproteinases (MMPs)^4^. In addition, RA-FLS exhibit an increased migratory potential, anchorage-independent growth and reduced contact inhibition along with high expression of cell-to-cell contact molecules such as Cadherin-11. They also show a prominent response to inflammatory cytokines such as interleukin 1 (IL-1) and tumour necrosis factor (TNF) as well as to growth factors such as the platelet-derived growth factor (PDGF)^5^. Moreover, RA-FLS can migrate from joint to joint, which potentially contributes to the dissemination of disease^4^. The mechanisms by which this specific phenotype is imprinted and then maintained in RA-FLS remains largely unclear, but growing evidence suggests that epigenetic mechanisms contribute prominently to the transformation of these cells^6^. A high-resolution epigenomic landscape of RA has been established indicating potential for integrative analyses to identify unanticipated therapeutic targets^7^. Here we extend upon this work and identify the LIM and SH3 domain protein 1 (Lasp1), a tumour-associated adaptor molecule originally cloned from metastatic breast cancer^8,9^, not only as part of one epigenomic cluster associated with autonomously aggressive RA-FLS, but also as one prominent epigenetic mark in two different animal models of the disease. We show that during chronic destructive arthritis, Lasp1 is induced in RA-FLS and constitutes a functional part of cadherin-11 adhesion structures. By independent binding to the Cadherin-11/β-Catenin complex and to F-Actin, Lasp1 is involved in the formation and dynamic resolution of zipper-like cell-to-cell contacts resembling adherens junctions (AJs). The loss or pharmacological blocking of Lasp1 has profound effects on the ability of arthritic FLS to form and remodel these zipper-like structures and resulted in a loss of important features of their aggressive phenotype.

## Results and discussion

### Lasp1 is associated with epigenetically co-modified regions in RA

Following the work of other groups to study epigenomic changes in arthritic FLS, we used an integrative approach to analyse 191 genome-wide datasets that in a previous study^7^ had been generated across 11 RA and 11 OA (osteoarthritis, control) patient samples and that are described in detail there^7^. Based on the diverse epigenomic data (including six histone modification marks, open chromatin, RNA-seq and DNA methylation), the integrative analysis described genomic regions sharing similar epigenomic profiles and identified 125 clusters^7^. Statistically significant differentially modified pathways involving the genes in the clusters with differentially modified epigenetic regions (DMER) were found in eight clusters. Among them, cluster 8 showed the highest DMER enrichment with 52% of the DMER differences between RA and OA due to the H3K27ac mark, which is associated with active enhancer regions.

**Fig. 1:**
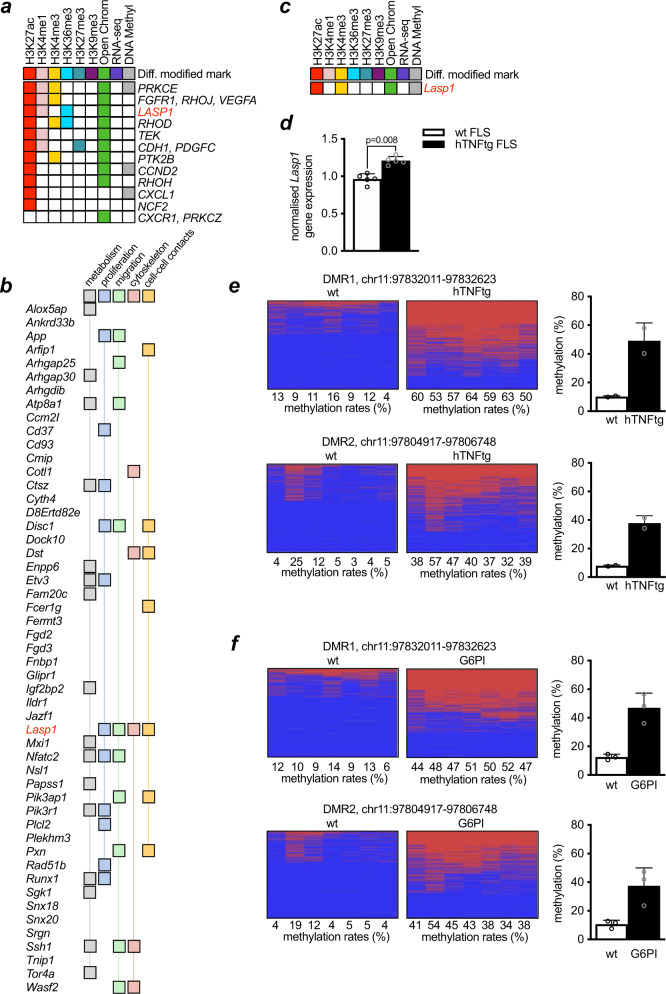
Lasp1 is an epigenetically modified gene under RA conditions. **a** Analysis of the epigenetically modified genes associated with differentially marked regions of the IL-8 signalling pathways identified *LASP1* as one candidate gene in RA-FLS. **b** Functional analysis of the 52 epigenetically modified genes in hTNFtg FLS with alterations in both chromatin structure and in DNA-methylation according to their known involvement in key cellular processes associated with the aggressive phenotype of arthritic FLS (proliferation, migration, cytoskeletal rearrangement and cell-to-cell contacts). **c** Summary of the epigenetic changes found in the *Lasp1* gene of hTNFtg FLS vs. wt FLS. **d** qPCR analysis of *Lasp1* mRNA expression levels in FLS from wt and hTNFtg mice (*n* = 5 each group). All data presented as mean ± SEM, *P < 0.05, **P < 0.001 (two-tailed Mann–Whitney *U* test). **e** Methylation analysis of two identified DMRs in FLS from hTNFtg and wt mice and analysis of changes in these DMRs (*n* = 2 each group). **f** Methylation analysis of the two identified DMRs in a second mouse model of RA, the G6PI arthritis, and statistical analysis of these DMRs (*n* = 3 each group). Source data are provided as a Source Data file.

Interestingly, the differentially modified pathway “IL-8 Signalling” was identified as significantly enriched in cluster 8 (Supplementary Fig. 1). The *LASP1* gene is differentially modified and plays an important role in the pathway (Fig. 1a). The genomic landscape with marked DMERs is shown in one RA and one OA patient sample within 500 kb of *LASP1* across nine marks, and is representative of differences between RA and OA FLS (Supplementary Fig. 2).

Based on these explorative human data, we were interested if chronic inflammation also leads to epigenetic modifications of *Lasp1* in FLS from animal models of RA. To this end, we used the human *TNFA* transgenic (hTNFtg) mouse model of RA^10^, in which chronic overexpression of human TNF leads to an RA-like destructive arthritis. We analysed the data that we had obtained when generating high resolution class 1 epigenomes of FLS from two wild type (wt) and two hTNFtg mice in frame of the German National Epigenome programme (DEEP), and which included the same epigenetic marks as in the human study. In the top 500 genes with alterations in chromatin structure, we identified 52 with additional changes in DNA-methylation. We next used the gene ontology annotation tools PANTHER (Protein ANalysis THrough Evolutionary Relationships, http://pantherdb.org)^11^ and GeneCards (https://www.genecards.org)^12^ to cluster these genes according to their known involvement in the cellular processes of proliferation, migration, cytoskeletal rearrangement and cell-to-cell contacts, which all have been associated with the aggressive transformation of RA FLS (Fig. 1b). In our analysis, Lasp1 was found to appear in all of these pathways, while no association was found with cell metabolism that we included additionally (Fig. 1b). Based on the occurrence of Lasp1 both in the data from aforementioned human analysis and in the analysis of our murine data, we performed in depth evaluation of the epigenetic changes within the *Lasp1* locus in hTNFtg FLS (Supplementary Fig. 3) and found that the *Lasp1* gene showed epigenetic changes in H3K27ac, H3K4me1, chromatin structure and DNA methylation (Fig. 1c). Although analysis of RNA-seq data did not show differences in *Lasp1* between wt and hTNFtg FLS, quantitative real-time PCR demonstrate that the *Lasp1* mRNA levels were significantly increased in hTNFtg FLS in comparison to FLS from wt mice (Fig. 1d).

As part of the epigenetic analyses, we performed whole genome bisulfite sequencing (WGBS) on control and hTNFtg FLS and found two differentially methylated regions (DMRs) within *the Lasp1* locus between the two mice groups. Both regions were hypermethylated in hTNFtg FLS, and the results were validated by targeted deep bisulfite sequencing (Fig. 1e). In addition, the intron containing DMR1 also hosted one differentially open region (DOR), also differing for the histone mark H3K27ac and another locus differential in H3K4me1. The DOR is more open in hTNFtg samples. To analyse the similarity of our replicates, we performed principal component analysis (PCA) of the data and PCA-plots indicated that overall, both conditions (control and hTNFtg) clustered together suggesting sufficiently small intra replicate variation as compared to variation between the two conditions (Supplementary Fig. 4). To confirm our data in a second murine model of RA, we used glucose-6-phosphate-isomerase (G6PI) mice^13^. In this model, arthritis is induced by immunisation with recombinant human G6PI and turns into a non-remitting, chronically destructive and progressive arthritis by depletion of regulatory T-cells^14,15^. Most interestingly, targeted deep bisulfite sequencing confirmed the results obtained for the first murine model and identified the same DMRs (Fig. 1f). It needs to be emphasised that the limited number of murine samples used for the epigenetic analyses, and particularly for generating the class I epigenome, imposes limitations to the data and requires that conclusions be drawn very cautiously. However, the fact that at least for some DMRs, similar changes were found in two different animal models of destructive arthritis, and that qPCR demonstrated increased expression of Lasp1 mRNA in hTNFtg FLS versus wt FLS, made us hypothesise that destructive arthritis may be associated with alterations in Lasp1.

**Fig. 2:**
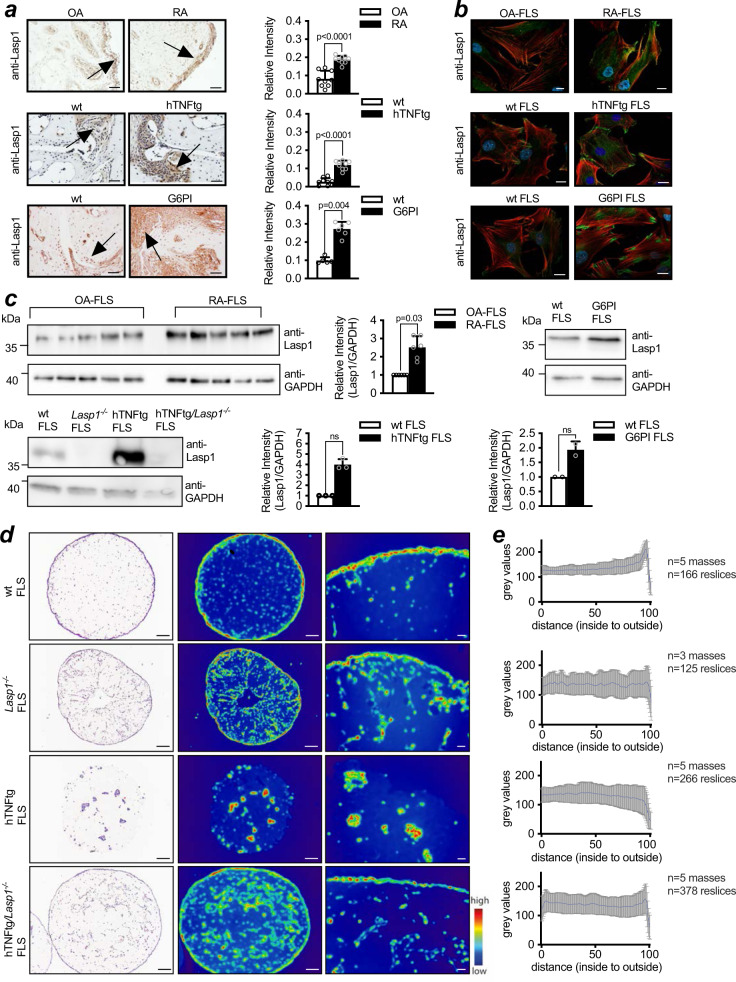
Lasp1 overexpression in RA synovium contributes to pathological 3D tissue formation. **a** Immunohistochemical staining and quantification for Lasp1 in synovial tissues from human patients with RA and OA (control) and in tarsal hind paws of mice with hTNFtg and G6PI arthritis vs. wild type (wt) control mice. Lasp1 positive cells are labelled in brown (black arrows) and the nuclei in blue. Haematoxylin was used for counterstaining (representative images of *n* ≥ 3 per group, scale bar: 50 µm). All data presented as mean ± SEM, *P < 0.05, **P < 0.001 (two-tailed Mann–Whitney *U* test). **b** Immunostaining of Lasp1 in FLS from these tissues in green. The F-Actin cytoskeleton is stained with Phalloidin-Rhodamine in red and the nuclei with DAPI in blue (representative images of *n* = 7 per group, scale bar: 10 µm). **c** Immunoblotting and quantification of Lasp1 expression in FLS from human patients with RA and OA (control) and in FLS from synovial tissues of mice with hTNFtg and G6PI arthritis vs. wt control mice. Lysates from *Lasp1*^*−/−*^ FLS and hTNFtg*/Lasp1*^*−/−*^ FLS were used as negative controls (representative images from *n* ≥ 3 independent experiments). All data presented as mean ± SD, *P < 0.05 (Wilcoxon test). **d** Histological staining of sections from 3D FLS synovial organ cultures. After 17 days of culture, the three-dimensional structures were fixed, embedded in paraffin and sections were stained with H&E (representative images of *n* = 3 per group, scale bar: 200 µm). **e** Cellular distribution in synovial organ sections (from Fig. 2d) represented as colour-coded density maps for each condition (left) and quantification via radial intensity analysis (right) (scale bars: 200 µm, and 20 µm in magnified images). Mean of grey values along a linescan was plotted from inside to outside (wt FLS *n* = 166 reslices; *Lasp1*^*−/−*^ FLS *n* = 125 reslices; hTNFtg FLS *n* = 226 reslices and hTNFtg*/Lasp1*^*−/−*^ FLS *n* = 378 reslices). Source data are provided as a Source Data file.

Based on these data as well as on the interesting association of Lasp1 with invasive cellular behaviour of tumour cells^16^, we next asked the question if the expression of Lasp1 is altered at protein level in FLS during chronic destructive arthritis. Immunohistochemical stainings of paraffin sections from human RA synovial tissues demonstrated a distinct, disease-specific expression of Lasp1 with increased staining for Lasp1 in both the lining and sublining layers as compared to osteoarthritic controls (Fig. 2a). These data were supported by immunofluorescence stainings of isolated FLS from human RA-patients and osteoarthritic controls as well as by Western Blot analyses of protein extracts from these cells (Fig. 2b, c). Analyses of tissue sections and isolated FLS from hTNFtg mice and from the G6PI model of RA further confirmed the human data and demonstrated increased Lasp1 expression levels also in FLS from these murine RA models (Fig. 2a–c). When investigating the subcellular expression pattern of Lasp1, we found it mainly at the cell periphery (Fig. 2b). Together, these data indicated that chronic destructive joint inflammation may lead to epigenetic changes in the *Lasp1* locus and is associated with increased expression in FLS.

### Regulation of FLS organisation, migration and contact dynamics by Lasp1

The apparent differences in the expression of Lasp1 between non-inflamed and inflammatory and invasive FLS prompted us to ask the question if *Lasp1* deficiency in FLS would alter synovial tissue formation and inflammation-dependent synovial pathology in a three-dimensional (3D) organ culture system in vitro. In this system, FLS are resuspended into a preformed basement membrane-like matrix and placed as a drop onto poly-2-hydroxyethylenmetacrylate-coated culture dishes to from 3D organoids with a characteristic lining and sublining structure resembling the synovial membrane (Fig. 2d)^17^. The 3D synovial structure formed by *Lasp1*^*−/−*^ FLS was largely comparable to that of the wild type FLS, with a clear lining-sublining division but some more FLS clustering in the centre of the matrices. In contrast, hTNFtg FLS formed unorganised cellular condensations with no evident synovial architecture and a lacking lining layer (Fig. 2d). Remarkably, deletion of *Lasp1* in the hTNFtg inflammatory background resulted in a rescue into organised cellular layers, comparable with those seen in wild type FLS matrices (Fig. 2d). To assess these differences across the experiments, cell localisation was super-imposed with colour-coded density mapping and cell distribution was quantified via radial intensity analysis. These analyses confirmed the differences between hTNFtg and hTNFtg*/Lasp1*^*−/−*^ FLS by clearly showing that particularly the lack of synovial lining formation, as observed in the hTNFtg FLS, is reversed in the hTNFtg*/Lasp1*^*−/−*^ FLS (Fig. 2e).

**Fig. 3:**
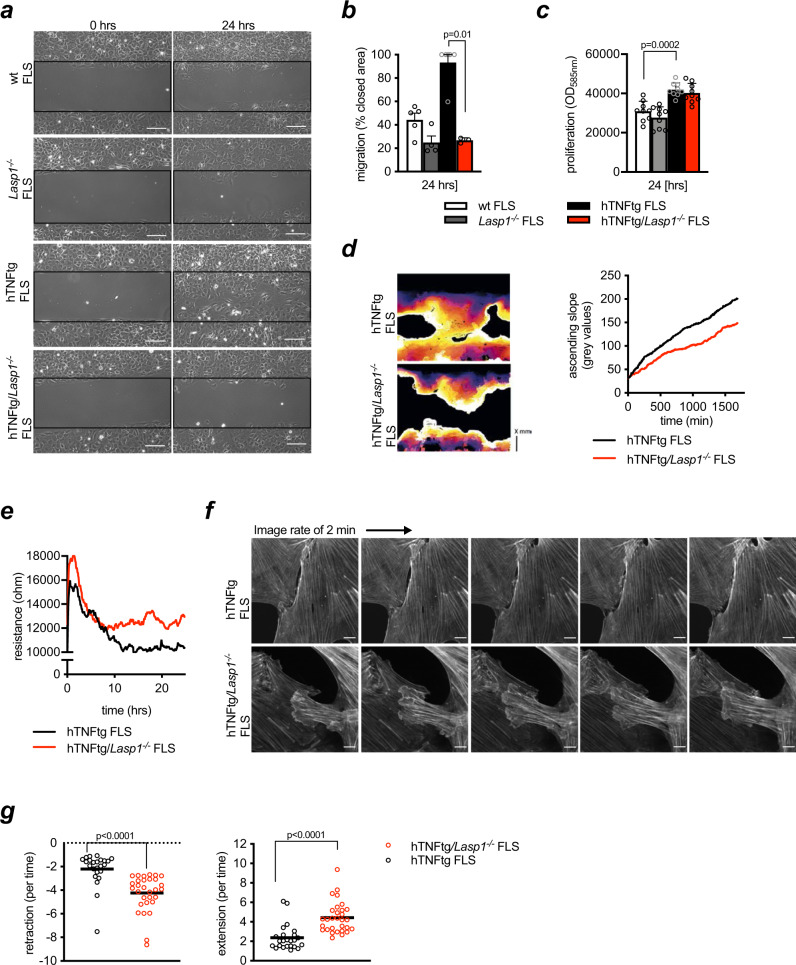
Lasp1 regulates arthritic FLS migration and cell-to-cell contact dynamics. **a** Cell migration of murine FLS from wt, *Lasp1*^*−/−*^, hTNFtg and hTNFtg*/Lasp1*^*−/−*^ mice as determined by a modified scratch assay after 0 and 24 h (representative images of *n* ≥ 3 per group, scale bar: 100 µm). **b** Quantification of the migration speed in this modified scratch assay (*n* ≥ 3 each group). All data presented as mean ± SEM, *P < 0.05 (two-tailed Mann–Whitney *U* test). **c** Analysis of the proliferation rate of adhered FLS from wt, *Lasp1*^*−/−*^, hTNFtg and hTNFtg*/Lasp1*^*−/−*^ mice as determined by the CyQuant^®^ Assay (*n* = 9 each group). All data presented as mean ± SEM, *P < 0.05, **P < 0.001, ***P < 0.0001 (two-tailed Mann–Whitney *U* test). **d** Exemplary analyses of live-cell imaging of cell migration of hTNFtg FLS and hTNFtg*/Lasp1*^*−/−*^ FLS in a modified scratch assay. The cell front was determined by edge detection and tracked over time. Linescan through front monitors front movement over time (graph hTNFtg FLS *n* = 18 linescans from 1 scratch assay; hTNFtg*/Lasp1*^*−/−*^ FLS *n* = 16 linescans from 1 scratch assay). The movies 1 and 2 are included in the Supplementary Data (scale bar: 100 µm). **e** Establishment of cell-to-cell contacts was analysed by electric cell/substrate impedance sensing (ECIS) and the total resistance reflecting the strength of cell-to-cell contacts was monitored over a time period of 26 h (representative measurement of *n* = 3 independent experiments). **f** Live-cell fluorescence imaging of FLS from hTNFtg and hTNFtg*/Lasp1*^*−/−*^ mice transduced with a lentiviral Life-Act-GFP construct. Imaging was performed for an hour with an image rate of two minutes (representative pictures of *n* = 3 per each group). The movies 3 and 4 are included in the Supplementary Data (scale bar: 10 µm). **g** Lamellipodial dynamics analysis. To quantify lamellipodia dynamics (from Fig. 3f), the extension-retraction behaviour was determined over time, and the difference in positive (left) and negative (right) displacement (hTNFtg FLS *n* = 3 reslices from 24 cells; hTNFtg/*Lasp1*^*−/−*^ FLS *n* = 3 reslices from 30 cells) was calculated. Source data are provided as a Source Data file.

Based on these observations as well as on the established role of Lasp1 in cell migration^9^, we next sought to determine potential effects of *Lasp1* deficiency on the migratory potential of arthritic FLS using a modified scratch assay. As expected, hTNFtg FLS exhibited a higher migration rate than wild type FLS, but the loss of *Lasp1* led to a reduction in FLS migration. More importantly, hTNFtg*/Lasp1*^*−/−*^ FLS exhibited a nearly normal migration rate in spite of their inflammatory background (−69.1% vs. hTNFtg FLS, *p* < 0.05, two-tailed Mann–Whitney *U* test, Fig. 3a, b). CyQuant^®^ proliferation assay showed no differences in proliferation rate between hTNFtg FLS and hTNFtg/*Lasp1*^*−/−*^ FLS, indicating that *Lasp1* deficiency over the observed period of time did not affect the proliferation (Fig. 3c). Live-cell imaging using time-lapse microscopy showed that hTNFtg FLS migrated with high speed and extensively spread across the culture surface exhibiting large numbers of stress fibres and highly dynamic cell-to-cell contacts (Fig. 3d, Supplementary Movie 1). In contrast, hTNFtg*/Lasp1*^*−/−*^ FLS migrated very slowly, with no extensive spreading and striking differences also in leading edge morphology and overall cell shape (Fig. 3d, Supplementary Movie 2).

These data along with the restoration of synovial lining formation that occurred when inflammatory hTNFtg FLS lacked the expression of Lasp1 made us wonder if Lasp1-dependent changes in the establishment and resolution of cell-to-cell contacts between FLS may cause the alterations seen both in the 3D synovial tissue model and the cell migration assay. We first performed electric cell-substrate impedance sensing (ECIS) to monitor cell-to-cell contacts and compare them between inflammatory hTNFtg FLS and hTNFtg/*Lasp1*^*−/−*^ FLS. In this system the initial establishment of cell-to-cell contacts and subsequent steady states are assessed by measuring the resistance of a freshly seeded cell monolayer to an electric current passed through. When seeded onto the measurement grids, hTNFtg FLS rapidly established cell-to-cell contacts as seen from an increase in the overall resistance that was followed by a lower steady state indicating a rapid and dynamic remodelling of cell-to-cell contacts (Fig. 3e and Supplementary Fig. 6a–c). Freshly seeded hTNFtg/*Lasp1*^*−/−*^ FLS, however, showed a slightly delayed but higher initial resistance followed by a higher steady state. These data suggested that the loss of *Lasp1* leads to more stable but far less dynamic cell-to-cell contacts (Fig. 3e and Supplementary Fig. 6a–c). By contrast and as expected, in epithelial cell lines such as caco-2 colon cells, formation of resistance occurred later but was overall higher (up to 80.000 ohm over 72 h, Supplementary Fig. 6d). Interestingly, we could also show that human RA-FLS exhibited a higher resistance than FLS from OA patients (Supplementary Fig. 6e–g). Moreover, the curve characteristics of the human RA-FLS were similar to those from arthritic hTNFtg mice while the curves of human OA-FLS were more comparable to hTNFtg*/Lasp1*^*−/−*^ FLS (Supplementary Fig. 6e–g). These data supported the notion that the aggressive phenotype of RA-FLS is associated with alterations in cell-cell contacts similar to those seen in arthritic hTNFtg mice. To substantiate the notion that the loss of *Lasp1* alters actin dynamics in inflammatory FLS, we assessed the F-Actin cytoskeleton remodelling in hTNFtg FLS and hTNFtg/*Lasp1*^*−/−*^ FLS by high resolution live-cell imaging using FLS that were lentivirally transduced with Life-Act-GFP. This method has been described before^18,19^ and allows for fluorescent visualisation of F-Actin dynamics in live cells. FLS from hTNFtg mice were observed to have different sites where they created cellular protrusions that underwent rapid remodelling along the interacting surfaces (Fig. 3f, Supplementary Movie 3). The dynamics and morphology of these F-Actin containing contacts was strikingly different in hTNFtg/*Lasp1*^*−/−*^ FLS. Particularly, we found that hTNFtg/*Lasp1*^*−/−*^ FLS formed lamellipodia-like contacts that were larger and more irregular than in hTNFtg FLS and failed to undergo the same remodelling (Fig. 3f, Supplementary Movie 4). To quantify the lamellipodia dynamics, we extracted the velocity from line-scans perpendicular to the cell edge and found that hTNFtg/*Lasp1*^*−/−*^ FLS exhibited significantly higher lamellipodial dynamics than hTNFtg FLS (Fig. 3g). These results made us hypothesise that in aggressively transformed arthritic FLS, the loss of *Lasp1* altered synovial tissue formation in vitro through affecting the dynamics of cellular contacts and migration rates.

### Cell-to-cell contacts in arthritic FLS are altered in the absence of *Lasp1*

**Fig. 4:**
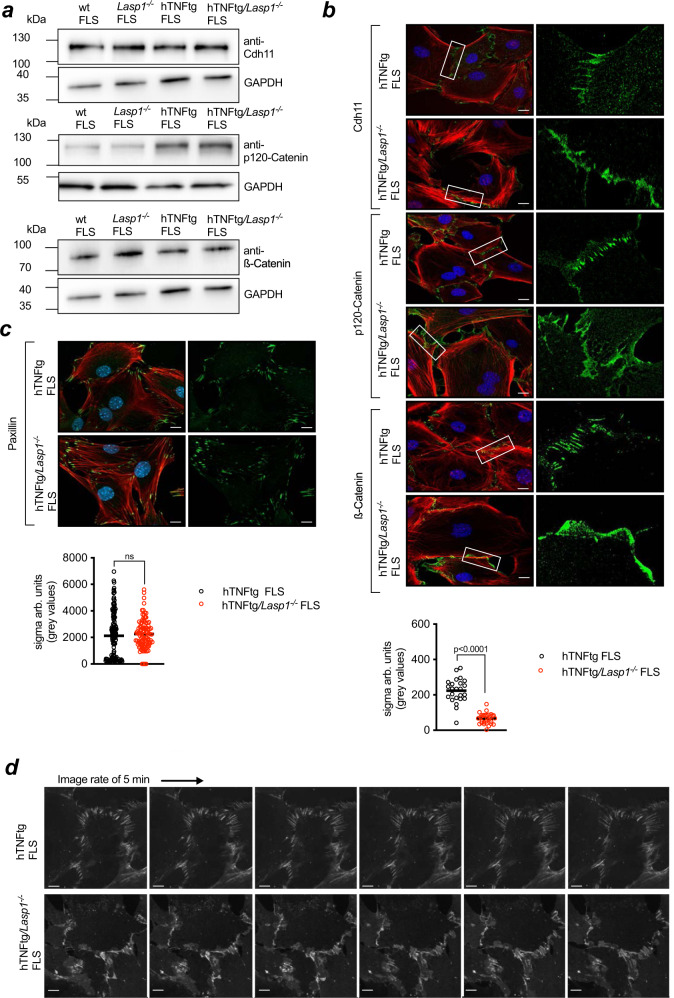
*Lasp1* deficiency alters cadherin-11 containing cell-to-cell contacts. **a** Immunoblotting for adherens junction proteins (Cdh11 = Cadherin-11, p120-Catenin and β-Catenin) in FLS from wt, *Lasp1*^*−/−*^, hTNFtg and hTNFtg*/Lasp1*^*−/−*^ mice (representative images from *n* = 4 independent experiments). **b** Immunocytochemical staining of these AJ proteins in FLS from hTNFtg and hTNFtg*/Lasp1*^*−/−*^ mice in green, the F-Actin cytoskeleton with Phalloidin-Rhodamine in red and the nuclei with DAPI in blue (representative images of *n* = 9 per group, scale bar: 10 µm). Furthermore, the differences in zipper-formation quantified by variance analysis. Intensity profile of β-Catenin staining was determined by measuring fluctuation of signal intensity in linescans parallel to cell periphery (hTNFtg FLS *n* = 7 reslices from 24 cells; hTNFtg*/Lasp1*^*−/−*^ FLS *n* = 7 reslices from 30 cells). **c** Immunocytochemical staining of the cell-matrix marker Paxillin in FLS from hTNFtg and hTNFtg*/Lasp1*^*−/−*^ mice in green, the F-Actin cytoskeleton with Phalloidin-Rhodamine in red and the nuclei with DAPI in blue (representative images of *n* = 4 per group, scale bar: 10 µm). Differences in formation quantified by variance analysis. The stainings were quantified by measuring fluctuation of signal intensity in linescans parallel to cell periphery (hTNFtg FLS *n* = 4 reslices from 144 cells; hTNFtg*/Lasp1*^*−/−*^ FLS *n* = 4 reslices from 102 cells). **d** Live-cell fluorescence imaging of FLS from hTNFtg and hTNFtg*/Lasp1*^*−/−*^ mice transduced with a lentiviral β-Catenin-Halo construct. Imaging was performed for an hour with an image rate of five minutes (representative pictures of *n* = 3 per each group). The movies 5 and 6 are included within the Supplementary Data (scale bar: 10 µm). Source data are provided as a Source Data file.

It was previously demonstrated that Cadherin-11 is a critical mediator of cell-to-cell adhesions in FLS and mediates the proper formation of AJs between these cells^17,20^. To test whether the deletion of *Lasp1* in arthritic FLS indeed leads to an alteration in formation of AJ- like cell-to-cell contacts, we analysed the expression and structural composition of Cadherin-11 complexes in hTNFtg and hTNFtg/*Lasp1*^*−/−*^ FLS. The complex has been characterised in other cells^21,22^ and is composed of Cadherin-11, p120-Catenin, β-Catenin and α-Catenin. Using immunocytochemical stainings and Western Blot analyses, we found no differences in the overall expression levels of Cadherin-11, p120-Catenin and β-Catenin between hTNFtg FLS and hTNFtg/*Lasp1*^*−/−*^ FLS (Fig. 4a). However, immunofluorescence imaging of Cadherin-11, p120-Catenin and β-Catenin along with F-Actin cytoskeletal staining revealed striking effects of a loss of *Lasp1* on the structure of the zipper-like adhesion complexes (Fig. 4b). In hTNFtg FLS the expression of the Cadherin-complex proteins was concentrated in structures of cell-to-cell contacts of adjacent cells forming typical zipper-like structures (Fig. 4b). However, these zipper-like structures were most strongly disrupted in hTNFtg/*Lasp1*^*−/−*^ FLS, and hTNFtg/*Lasp1*^*−/−*^ FLS showed uniformly dense and unorganized cell-to-cell adhesions without the typical zipper-like appearance (Fig. 4b). Computational analyses of the β-Catenin staining pattern via calculation of grey-value distribution along cell boundaries demonstrated that hTNFtg/*Lasp1*^*−/−*^ FLS exhibited significantly lower variance than hTNFtg FLS (−1.2% vs. hTNFtg FLS, *p* < 0.001, two-tailed Mann–Whitney *U* test), arguing that significantly fewer zipper-like, but more stable laminar structures were present (Fig. 4b). In contrast, both genotypes showed no alteration in the expression levels of the well-known cell-matrix marker Paxillin (Fig. 4c). The described β-Catenin pattern was also seen in live-cell fluorescence imaging of hTNFtg and hTNFtg*/Lasp1*^*−/−*^ FLS that had been lentivirally transduced with a β-Catenin-Halo construct^19^ (Fig. 4d, Supplementary Movie 5). Again, hTNFtg FLS formed very dynamical β-Catenin-containing, zipper-like structures, while *Lasp1* deficiency led to disruption of these structures and resulted in a far less dynamic establishment of denser and less organised β-Catenin containing cell-to-cell contacts (Fig. 4d, Supplementary Movie 6). All together, these data indicated that in the absence of *Lasp1*, arthritic FLS exhibit an impaired ability to form zipper-like cell-to-cell contacts associated with the Cadherin-11 complex.

### Lasp1 is part of Cadherin-11-mediated contacts and binds directly to the AJ complex

**Fig. 5:**
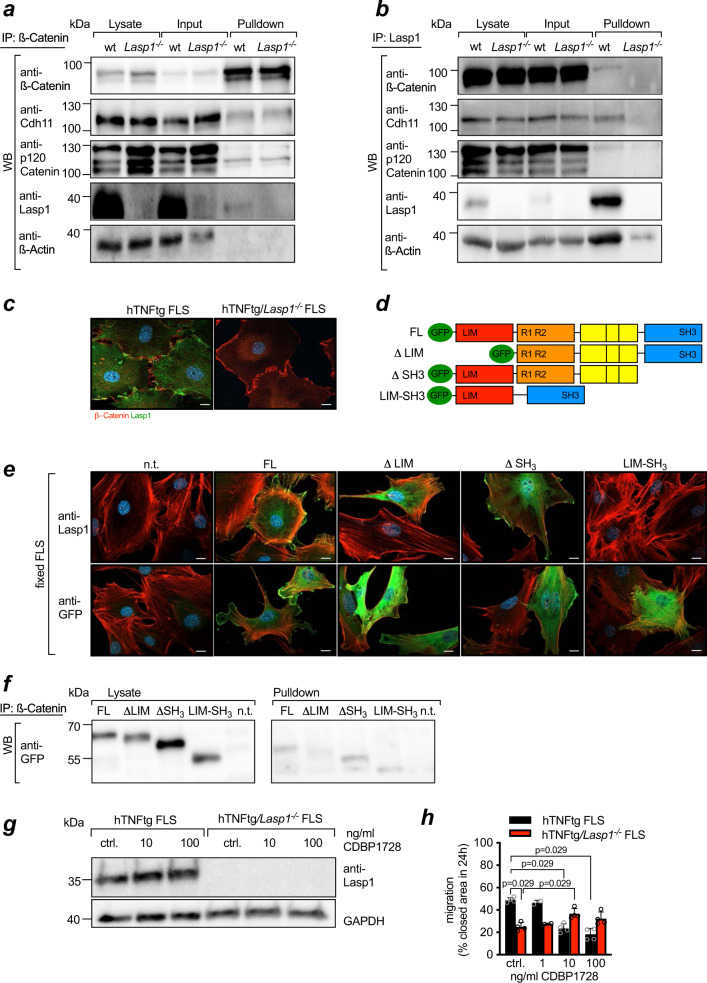
Lasp1 is a functional part of the cadherin-11/β-catenin complex. **a**, **b** Co-Immunoprecipitation (Co-IP) of AJ proteins, Lasp1 and β-Actin in cell lysates from wt FLS and *Lasp1*^*−/−*^ FLS (representative images of *n* = 3 independent experiments). **c** Co-localisation between Lasp1 (green) and β-Catenin (red) using immunofluorescence stainings (representative images of *n* = 3 independent experiments, scale bar: 10 µm). **d** Schematic overview of the GFP-tagged Lasp1 used constructs. A Lasp1 full length (FL) construct, a deletion construct missing the LIM domain (Δ LIM), a deletion construct missing the SH3 domain (ΔSH3) and a deletion mutant without the F-Actin binding nebulin-repeats (LIM-SH3). **e** Immunofluorescent stainings demonstrating the expression of Lasp1 and GFP in *Lasp1*^*−/−*^ FLS reconstituted with the Lasp1 constructs. Antibody staining is in green, the F-Actin cytoskeleton with Phalloidin-Rhodamine in red and the nuclei with DAPI in blue (representative images of *n* = 3 independent experiments, scale bar: 10 µm). **f** Co-IP of β-Catenin and Lasp1 in cell lysates from *Lasp1*^*−/−*^ mouse embryonic fibroblasts (representative images of *n* = 3 independent experiments). **g** Immunoblotting for Lasp1 in the absence or presence of the Lasp1 blocking peptide CDBP1728 (representative blot of *n* = 3 independent experiments). **h** Quantification of the cell migration speed in a modified scratch assay in the absence or presence of the Lasp1 blocking peptide CDBP1728 (*n* = 4 each group). Data presented as mean ± SEM, *P < 0.05 (two-tailed Mann–Whitney *U* test). Source data are provided as a Source Data file.

Next, we sought to analyse whether Lasp1 is part of the AJ protein complex and performed co-immunoprecipitation experiments using antibodies against the individual members of the complex along with wt or *Lasp1*^*−/−*^ FLS. Immunoprecipitation with antibodies against β-Catenin and subsequent immunoblot with antibodies against Lasp1 and the well-known interaction partners Cadherin-11, p120-Catenin and β-Catenin clearly demonstrated binding of β-Catenin with Cadherin-11 and p120-Catenin, but also showed binding of β-Catenin to Lasp1 (Fig. 5a). In *Lasp1*^*−/−*^ FLS, no such binding was seen (Fig. 5a). To confirm these data, we performed the reverse experiments, in which we immunoprecipitated with antibodies against Lasp1, and then subjected the precipitated proteins to an immunoblot with antibodies against the AJ complex members Cadherin-11, p120-Catenin and β-Catenin. Again, we found prominent binding of Lasp1 to both Cadherin-11 and β-Catenin in wt FLS, which was not seen in *Lasp1*^*−/−*^ cells (Fig. 5b). Investigating the co-localisation of Lasp1 and β-Catenin by immunofluorescence stainings and in transmission electron microscopy (TEM), we found Lasp1 and β-Catenin in close proximity to the F-Actin cytoskeleton (Fig. 5c and Supplementary Fig. 7b). While these data are descriptive, and the TEM-data have been obtained only from a limited number of samples and therefore require cautious interpretation, they further suggested that Lasp1 may serve as an interaction partner of both the Cadherin-11/β-Catenin complex and F-Actin and is part of the AJ machinery in FLS.

As Lasp1 is a well-established binding partner of F-Actin^23^, to which it binds through its two nebulin-repeats, we next sought to analyse if the observed binding of Lasp1 to the Cadherin-11/β-Catenin complex is indirectly mediated by the Actin-binding properties of Lasp1 or independent of F-Actin. To this end, we generated different GFP-tagged deletion mutants of Lasp1 in which either the LIM domain (ΔLIM), the SH3 domain (ΔSH3) or the F-Actin binding nebulin-repeats (LIM-SH3) were missing (Fig. 5d). We then reconstituted *Lasp1*^*−/−*^ fibroblasts with either a full length Lasp1 construct or one of these mutants (Supplementary Fig. 8) and in addition to immunohistochemical stainings again performed immunoprecipitation experiments. As expected, full length Lasp1 was found within structures of cell-matrix interactions and cell-to-cell contacts (Fig. 5e). Interestingly, fibroblasts that were transfected with the ΔLIM construct showed a more cytosolic Lasp1 expression, suggesting that the LIM domain of Lasp1 is required for its localisation into cell-to-cell contacts (Fig. 5e). Immunoprecipitation with antibodies against β-Catenin and subsequent immunoblot with an anti-GFP antibody demonstrated binding of β-Catenin not only to full length Lasp1 and the ΔSH3 mutant, but also to the LIM-SH3 mutant that cannot bind to F-Actin (Fig. 5f), which makes us hypothesise that the binding of Lasp1 to the adhesion complex is independent of the well-established F-Actin binding capability of Lasp1. As one important limitation of the present data, the strong interaction of Cadherin-11 with β-Catenin, our experiments do not clarify whether Cadherin-11 or β-Catenin or the complex of both constitutes the interaction partner of Lasp1 in mouse embryonic fibroblasts (MEFs). However, as no prominent binding could be seen in the lysates of *Lasp1*^*−/−*^ MEFs transfected with the ΔLIM construct, our immunoprecipitation results may support the data from our immunochemical stainings and suggest that Lasp1 binds to the Cadherin-11/β-Catenin complex via its LIM domain.

To further analyse whether the interaction of Lasp1 with the Cadherin-11/β-Catenin complex through the LIM domain mediates the observed effects, and to explore the possibility of therapeutically modulating the Lasp1/Cadherin-11/β-Catenin interaction, we used a blocking peptide against a LIM-domain sequence of Lasp1 (CDBP1728). CDBP1728 did not affect expression of Lasp1 in hTNFtg FLS and had no effect on hTNFtg/*Lasp1*^*−/−*^ FLS (Fig. 5g). However, when added to hTNFtg FLS, it largely reproduced the phenotype of the hTNFtg/*Lasp1*^*−/−*^ FLS with respect to cell migration and this effect was clearly concentration dependent (Fig. 5h).

### Regulation of FLS effector functions by Lasp1

**Fig. 6:**
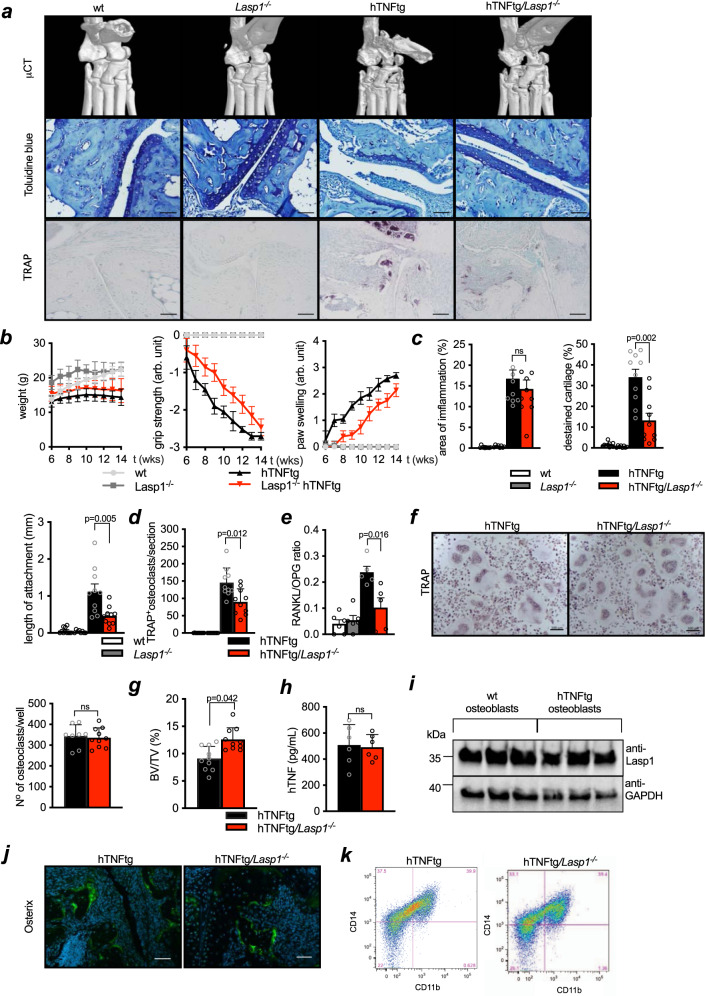
*Lasp1* deficiency reduces joint destruction in hTNFtg mice and FLS. **a** Bone destruction as assessed by microCT analysis and toluidine blue stainings (scale bar: 50 µm) of paraffin sections from hind paws from 12 weeks old wt, *Lasp1*^*−/−*^, hTNFtg and hTNFtg*/Lasp1*^*−/−*^ female mice (*n* ≥ 8 in each group). Osteoclasts were visualised by TRAP stainings (*n* ≥ 8 in each group, scale bar: 50 µm). **b** Body weight and clinical joint scores of wt, *Lasp1*^*−/−*^, hTNFtg and hTNFtg*/Lasp1*^*−/−*^ mice from week 6 to 14 of age (*n* ≥ 5 in each group). **c** Histomorphometric analysis of synovial pannus formation, length of synovial tissue attachment to joint cartilage, total cartilage area and destained cartilage in tarsal joints of hTNFtg and hTNFtg*/Lasp1*^*−/−*^ female mice (*n* ≥ 8 each group). Data presented as mean ± SEM, *P < 0.05, **P < 0.001 (two-tailed Mann–Whitney *U* test). **d** Quantification of TRAP^+^ osteoclasts (*n* ≥ 8 in each group). Data presented as mean ± SEM, *P = 0.01 (two-tailed Mann–Whitney *U* test). **e** Evaluation of RANKL/OPG ratio in FLS from arthritic and control mice (*n* ≥ 5) by ELISA. Data presented as mean ± SEM, *P = 0.01 (two-tailed Mann–Whitney *U* test). **f** In vitro osteoclast differentiation assays and the tartrate resistant acid phosphatase (TRAP) stainings (representative images and numerical assessment of *n* = 3 independent experiments, scale bar: 100 µm). Data presented as mean ± SEM, *P = 0.01 (two-tailed Mann–Whitney *U* test). **g** MicroCT quantifications of bone volume/tissue volume (BV/TV) ratios in hTNFtg and hTNFtg*/Lasp1*^*−/−*^ mice (*n* = 10 each group). Data presented as mean ± SEM, *P < 0.05, **P < 0.001 (two-tailed Mann–Whitney *U* test). **h** Human TNF levels in the sera of hTNFtg and hTNFtg*/Lasp1*^*−/−*^ female mice (*n* = 6 each group). Data presented as mean ± SEM (two-tailed Mann–Whitney *U* test). **i** Immunoblotting for Lasp1 in osteoblasts from wt and hTNFtg mice (*n* = 3 each group). **j** Immunofluorescent stainings demonstrating the expression of Osterix in tissue from hTNFtg and hTNFtg*/Lasp1*^*−/−*^ mice. Antibody staining is in green and the nuclei with DAPI in blue (representative images of *n* = 3 independent experiments, scale bar: 50 µm). **k** Analysis of macrophages population in collagenase digested hind paws as determined by flow cytometry (representative images of *n* = 4 independent experiments). Source data are provided as a Source Data file.

Based on these data we asked the question if the lack of *Lasp1* would affect the onset and severity of RA-like disease in mice and the disease-associated FLS response. To this end, we used the hTNFtg mice that develop destructive arthritis at 5-6 weeks of age^24^ and crossed them with the *Lasp1*^*−/−*^ mice to generate hTNFtg/*Lasp1*^*−/−*^ animals (Fig. 6a). Interestingly, the lack of *Lasp1* resulted in reduced arthritis severity as seen from less paw swelling (Fig. 6b, Supplementary Fig. 12). Although grip strength as one established composite functional parameter most likely is influenced by different factors also including pain, these data provided a first hint that structural damage may be less pronounced in the absence of *Lasp1*. Interestingly, overall inflammation and body weight were not significantly different between the hTNFtg and hTNFtg*/Lasp1*^*−/−*^ mice. In fact, both genotypes showed similar degrees of inflammatory pannus formation in histomorphometric analyses (Fig. 6c) and similar levels of inflammatory cell infiltration when synovial tissues were examined for the presence of macrophages (CD-11b and CD-14 positive cells) using flow cytometry (Fig. 6k). However, histomorphometric analyses showed less cartilage damage in hTNFtg/*Lasp1*^*−/−*^ mice (−61.2% vs. hTNFtg, *p* < 0.01, two-tailed Mann–Whitney *U* test, Fig. 6b, c) and as well as less attachment of the synovial lining tissue to the cartilage (−58.9% vs. hTNFtg, *p* < 0.05, two-tailed Mann–Whitney *U* test, Fig. 6b, c).

Next, we performed microCT analyses to study potential effects of *Lasp1* deficiency on arthritic bone destruction in the hTNFtg mice (Fig. 6a). These analyses showed that the loss of *Lasp1* was associated with less bone destruction as seen from significantly increased bone volume per tissue volume (BV/TV) ratios in hTNFtg*/Lasp1*^*−/−*^ mice (+3.5% vs. hTNFtg, *p* < 0.05, two-tailed Mann–Whitney *U* test, Fig. 6g). In line with these observations, quantification of osteoclast numbers in tartrate-resistant acid phosphatase (TRAP)- stained tissue sections of hind paws from 12 weeks old mice demonstrated significantly fewer osteoclasts in hTNFtg*/Lasp1*^*−/−*^ animals (−38.0% vs. hTNFtg, *p* < 0.05, two-tailed Mann–Whitney *U* test, Fig. 6a, d).

To study potential links between *Lasp1* deficiency and reduced osteoclast numbers in vivo, we performed ELISA analyses in cultured FLS to measure RANKL and OPG levels. These analyses showed significantly lower RANKL/OPG ratios in hTNFtg/*Lasp1*^*−/−*^ FLS in comparison to hTNFtg FLS (−58.3% vs. hTNFtg, *p* < 0.05, two-tailed Mann–Whitney *U* test, Fig. 6e).

Next, we performed in vitro osteoclast differentiation assays to see if there is an intrinsic defect in osteoclastogenesis upon the loss of *Lasp1*. In vitro TRAP stainings demonstrated no differences in the number of osteoclasts differentiated in vitro from hTNFtg and hTNFtg/*Lasp1*^*−/−*^ precursors (Fig. 6f). These data showed that the global loss of *Lasp1* in the inflammatory hTNFtg background is not associated with defects in osteoclastogenesis per se but only occurred in the in vivo situation and that at least in vitro, the loss of *Lasp1* is associated with reduced RANKL/OPG ratios in hTNFtg mice. Interestingly, we found no differences in expression levels of *Lasp1* in isolated osteoblasts from wt and hTNFtg mice (Fig. 6i) and there was the same pattern of Osterix staining in sections from hTNFtg and hTNFtg/*Lasp1*^*−/−*^ mice (Fig. 6j), providing no evidence for altered osteoblastogenesis as a key contributing factor.

While based on these data it may be hypothesised that the mesenchymal FLS compartment is largely responsible for reduced joint damage in hTNFtg*/Lasp1*^*−/−*^ mice, cell-type specific deletion of *Lasp1* along with additional functional characterisation of other cell types would be required to substantiate this hypothesis and to definitively exclude that other cellular compartments or interactions significantly contribute to or mediate the association between a loss of *Lasp1* and reduced joint destruction in the hTNFtg mice. This is also because analyses of MMP3 as well as MMP9- levels by ELISA did not demonstrate any differences between FLS from wt and *Lasp1*^*−/−*^ mice regardless of their exposure to TNF (Supplementary Fig. 9b, c) indicating that the differences in their destructive potential are not related directly to MMP secretion or activation but involve indirect mechanisms which may be related to altered cellular interactions as seen here.

**Fig. 7:**
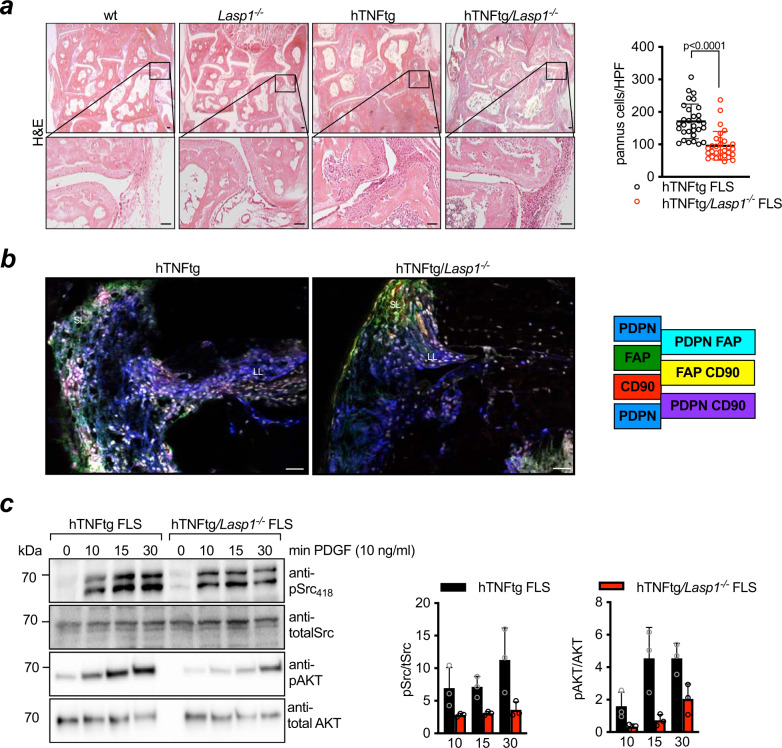
Lasp1 affects tissue architecture in the synovium of hTNFtg mice. **a** H&E stainings (scale bar: 50 µm) of paraffin sections from hind paws from 12 weeks old wt, *Lasp1*^*−/−*^, hTNFtg and hTNFtg*/Lasp1*^*−/−*^ female mice (*n* = 3 in each group). Evaluation of synovial cell density was performed by counting cell numbers per high power field (HPF, 10.000 µm^2^) (*n* = 10, 3 HPF/animal). Data presented as mean ± SEM, *P < 0.05, **P < 0.001, ***P < 0.0001 (two-tailed Mann–Whitney *U* test). **b** Immunofluorescence staining for PDPN, FAPα, THY1 expression and the nuclei with DAPI in white in 12 weeks old joints (representative images of *n* = 5 per group, scale bar: 10 µm). **c** Immunoblotting and quantification for phospho-specific Src kinase (pSrc_418_) and total Src kinase expression as well as for phospho-specific AKT kinase (pAKT) and total AKT in FLS from hTNFtg and hTNFtg*/Lasp1*^*−/−*^ mice stimulated with recombinant murine PDGF for the indicated time periods (*n* = 3 each group). Source data are provided as a Source Data file.

Next, and based on these data, we sought to determine if the alterations in cell-to-cell contacts of FLS and in 3D organoid formation translate into changes of synovial tissue composition in vivo. To this end, we analysed the synovial morphology of hTNFtg and hTNFtg*/Lasp1*^*−/−*^ mice in more detail^20^. Performing H&E stainings of hind paws from hTNFtg and hTNFtg*/Lasp1*^*−/−*^ mice we observed a more dense synovial structure in hTNFtg mice with the pannus tissue deeply invading cartilage and bone. By contrast, the synovium of hTNFtg*/Lasp1*^*−/−*^ mice showed a looser synovial organisation that despite the presence of inflammatory infiltrates exhibit a more normal morphology and a reduced cellular density (−44,12% vs. hTNFtg, *p* < 0.001, two-tailed Mann–Whitney *U* test, Fig. 7a). In order to substantiate this picture and establish a quantifiable link between *Lasp1* deficiency and altered synovial tissue architecture and FLS biology in destructive arthritis, we sought to better characterise FLS populations in hTNFtg and hTNFtg*/Lasp1*^*−/−*^ mice. Based on recently published studies^25,26^ in which FLS subsets were described and characterised by distinct surface markers, we performed additional analyses to investigate if similar FLS subsets were also detectable in hTNFtg mice and whether these subsets would be affected by *Lasp1* deficiency. To this end, we used immunofluorescence stainings and flow cytometry as described before^26^ to obtain a more detailed profiling of FLS in lining layer and the sublining layer of the synovium. We analysed the localisation of FLS subtypes at the sites of pannus invasion in hTNFtg and hTNFtg*/Lasp1*^*−/−*^ mice using fibroblast activation protein-α (FAPα), Podoplanin (PDPN) and thymus cell antigen 1 (THY1, also known as CD90) antibodies. Interestingly, hTNFtg*/Lasp1*^*−/−*^ mice had more PDPN^+^ cells in the lining layer and more PDPN^+^ CD90^+^ cells in the sublining layers of the synovium than hTNFtg animals as shown by representative immunofluorescence images (Fig. 7b). These findings were also supported by some exploratory FACS analyses of freshly isolated FLS derived from the hind paws of two hTNFtg and two hTNFtg*/Lasp1*^*−/−*^ mice (Supplementary Fig. 10). Finally, having shown that Lasp1 as part of the Cadherin-11/β-Catenin complex regulates FLS migration and synovial tissue organisation in destructive arthritis, we asked if Lasp1, through this role, also regulates the response of arthritic FLS to soluble signals such as the platelet-derived growth factor (PDGF). The PDGF signalling pathway has been associated with the aggressive transformation of RA-FLS and it has been demonstrated that PDGF not only promotes FLS proliferation^27^, but also the formation of invasive adhesion structures^28^. Moreover, some data suggest that the PDGF receptor (PDGFR) associates with the Cadherin/β-Catenin complex to modulate cell migration^29^, and an even more recent study has shown that interactions between Cadherin-11 and PDGFR signalling links cell adhesion and proliferation via the PI3K/Akt pathway^30^. Therefore, we wondered if the PDGF response of arthritic FLS would be affected by the lack of *Lasp1*. Indeed, *Lasp1* deficiency in the hTNFtg background led to a decreased phosphorylation of Src at tyrosine 418 and AKT at serine 473 following PDGF stimulation in comparison to hTNFtg FLS (Fig. 7c) providing a potential explanation for the prominent effects of *Lasp1* deficiency or its therapeutic inhibition in the context of inflammatory arthritis. Moreover, these data may further support the concept that the Cadherin-based AJs not only mediate organised cell-to-cell adhesion but also serve for the integration of extracellular signals.

Collectively, our in vivo and ex vivo findings together with the in vitro experiments as shown in Fig. 2d to us aid the hypothesis that the deficiency of *Lasp1* alters the establishment and dynamics of cell-to-cell contacts and leads to changes in synovial tissue composition that is associated with a less destructive phenotype in the hTNFtg mouse model of human RA as also shown by the reduced synovial cell density (Fig. 7a). It may be argued that the higher number of lining FLS in hTNFtg*/Lasp1*^*−/−*^ mice as observed in our immunofluorescence studies as well as in our explorative FACS analyses in a limited number of mice, together with the less severe invasion and destruction as shown in Fig. 6 contrasts the concepts on the role of lining FLS in joint destruction as proposed recently^26^. However, this would only be the case if the destructive potential of these FLS remains the same. Here, we hypothesise that the loss of *Lasp1* and subsequently altered cellular interactions at least partially revert the aggressive phenotype, in which case restoration of tissue architecture with a higher number of lining cells may well lead to less destruction. Obviously, our analyses of FLS subsets may be considered speculative as they have important limitations and conclusions from these data need to be drawn very cautiously, particularly because of the limited number of animals and, thus, the lack of statistical significance, the limited number of parameters analysed and the lack of a FLS-specific deletion of *Lasp1*. However, our data all together provide evidence for a novel role of Lasp1 as a component of Cadherin-11-mediated adherens junctions that contributes to the autonomously aggressive behaviour of FLS in destructive arthritis by altering the dynamics of cell-to-cell contacts between FLS and, thus, tissue composition. We also show that the genetic deletion of *Lasp1* or the blocking of the LIM- interaction domain of Lasp1 may counteract important aspects of the invasive phenotype of arthritic FLS, which in the light of increasing data suggesting a role for both Cadherins and Lasp1 in cell invasion, transmigration and tumourigenesis will have broad implications.

## Methods

### Human synovial tissues

The ethics committees of the Medical University of the University Hospital Münster approved all studies with human samples. Samples of synovial tissues from subjects with RA or OA (according to the 1987 revised American College of Rheumatology criteria for RA and OA) were obtained as operational waste at joint replacement surgery. In addition, synovial tissues of patients included in the early arthritis patient cohort in Birmingham (BEACON) were used in this study. All patients were naïve to treatment with disease modifying anti-rheumatic drugs (DMARDs) and corticosteroids at inclusion. The tissue samples were kindly provided by Prof. Dr. Christopher Buckley (Birmingham, UK). All individuals provided informed consent prior to surgery.

### Animals

The hTNFtg mice (strain Tg197; obtained from Alexander Fleming Biomedical Science Research Center, Vari, Greece) and the B6.129×1-Lasp1tm1Chew/J (*Lasp1*^*−/−*^*)* mice have been described previously^10,31^. Both mouse strains were maintained in the C57BL/6 genetic background, interbred with each other and analysed up to week 14. The primers for genotyping are listed in Suppl. Table 1. To investigate the severity of the disease an arbitrary score was used, including weight, paw swelling and grip strength^32^. All data were generated from sex and age-matched littermates. Mice were kept under individually ventilated cage (IVC) conditions with alternate 12 h cycles of dark and light. Animals were allowed free excess to feed and water ad libitum. All experimental procedures were carried out in strict adherence to the rules and guidelines for the ethical use of animals in research and were approved by the State Office for Nature, Environment and Consumer Affairs (Landesamt für Natur, Umwelt und Verbraucherschutz (LANUV) NRW, Germany) under the reference 8.87–51.05.2011.033 and 84-02.04.2014.A519. The G6PI-induced arthritis model was performed as described previously in the group of Kamradt^32^ and the samples were analysed 56 days after immunisation with G6PI.

### Integrative analysis in human samples

The previous established high-resolution global epigenomic landscape for RA grouped the RA genome into 125 clusters based on epigenetic marks in multiple regulatory/functional elements. Pathway analysis was performed using Ingenuity pathway analysis (IPA, QIAGEN Redwood City) on cluster 8, which has the highest DMER enrichment. The genome browser screenshot of *LASP1* was generated by IGV^33^.

### DNA methylation analysis

Whole genome bisulfite sequencing was performed^34,35^. Differentially methylated regions (DMRs) with a minimum size of four CpGs and a minimum methylation difference of 0.3 were detected by applying bsmooth^36^ and metilene^37^. Targeted deep bisulfite sequencing was performed^38^ using the primer pairs and conditions described in Suppl. Table 2.

### DNase I sequencing

Nuclei were isolated by using buffer A (60 mM KCl, 15 mM Tris-HCl (pH 8.0), 15 mM NaCl, 1 mM EDTA (pH 8.0), 0.5 mM EGTA (pH 8.0), 0.5 mM spermidine free base) supplemented with 0,35% IGEPAL and incubation on ice for 15 min. Nuclei were treated with DNase I (20-60U/ml) for 3 min at 37 °C and the reactions were stopped with stop buffer (50 mM Tris-Cl (pH 8.0), 100 mM NaCl, 0.1% SDS, 100 mM EDTA (pH 8.0), 1 mM spermidine and 0.3 mM spermine) supplemented with proteinase K (50 µg/ml) and incubation at 55 °C for 1 h. DNA was then purified using phenol chloroform extraction and double-hit fragments of 100–500 bp were selected by sequential purifications with Agencourt AMPure XP Beads (Beckman Coulter, Brea, USA). Sequencing libraries were prepared from 8 ng of purified DNA using the TruSeq ChIP Library Preparation kit (Illumina, San Diego, USA) according to the manufacturers protocol and sequenced on HiSeq v3 paired-end flow cells (HiSeq2500 system)^39^.

### Differentially open regions

The union of accessible regions from all samples was created by merging Nucleosome Depleted Regions (NDRs) from all samples with bedtools^40^. As each end of a sequenced fragment represent one instance of DNase I activity we counted the number of 5‘ start sites of both forward and reverse reads overlapping the union of NDRs. This was done with featureCounts^41^ only considering primary alignments and excluding duplicated fragments. Including technical replicates, control samples were contrasted against RA samples with edgeR^42^ and the limma/voom combination^43^. We considered the top 500 differential regions.

### ChIP-sequencing

Cells were crosslinked in 1% formaldehyde at a concentration of 1 × 10^6^ cells per ml for 5 min at room temperature under rotation, followed by quenching in 0.125 M glycine for 10 min. The crosslinked cells were then pelleted by centrifugation at 405 × *g* for 5 min at 4 °C. A total of 2 × 10^6^ fixed cells were lysed in 100 μl 1% SDS lysis buffer (100 mM NaCl, 50 mM Tris-HCl pH 8.1, 5 mM EDTA, 0.2% NaN_3_, 1% SDS and 3% Triton X) for 10 min on ice. The cell lysate was then diluted to 0.33% SDS (or 300 μl) with ChIP Dilution buffer (50 mM Tris-HCl pH 8.6, 100 mM NaCl, 5 mM EDTA and 0.2% NaN_3_) and resuspended several times using a syringe. The cell lysate was then divided into 150 μl aliquots and sheared on a Bioruptor Pico at high intensity for 2 × 8 cycles of 30 sec of 30 sec ON and 30 sec OFF. The sheared chromatin lysate was then centrifuged at 13 × *g* for 30 min at 4 °C to pellet debris. 300 µl of chromatin (2 × 10^6^ cells) was diluted with 950 µl ChIP dilution buffer and 6.25 µl protease inhibitor cocktail (Diagenode) to 1,256.5 μl and then mixed without vortexing. For the ChIPs 200 µl of chromatin were mixed with 1 µg antibodies against H3K4me1 (Diagenode, pAb-194–050, Lot A1863-001D), H3K4me3 (Diagenode, pAb-003-050, Lot A5051-001P), H3K4me9 (Diagenode, pAb-193-050, Lot A1671-001P), H3K27ac (Diagenode, pAb-196-050, Lot A1723-0040), H3K27me3 (Diagenode, pAb-195-050, A1811-001P) and H3K27me3 (Diagenode, pAb-192-050, Lot A1847-001P). ChIPs were performed on IP-Star Compact Automated System (Diagenode) using the ChIP-indirect method and the Auto histone ChIP Kit (Diagenode). For the control 20 µl chromatin was diluted with 80 µl elution buffer. 4 µl of 5 M NaCl was added to chromatin. Chromatin was de-crosslinked at 65 °C overnight, followed by 1 h of RNAse A treatment (2 µl) at 60 °C and 3 h Proteinase K treatment (3 μl) at 55 °C. The DNA was then purified from the solution via phenol chloroform precipitation at −80 °C overnight. The DNA resuspended in 15 μl of nuclease free dH20^44,45^. The purified DNA was quantified by Quibit. ChIP libraries were generated using Diagenode MicroPlex library preparation kits (C05010010) for Mf05 and Mf06 and NEBNext® Ultra DNA Library Prep Kit for Illumina® (E7370S/L) for Mf07 and Mf08 according to the manufacturer’s instructions. The libraries were paired-end sequenced on an Illumina HiSeq 2500 platform.

In addition, Chip-seq was normalised using the bamCoverage tool (version 3.4.3) from the deepTools2 package^46^. Used parameters are -binSize 10, -minMappingQuality 30, -normalizeUsing RPGC, -effectiveGenomeSize 2150570000, -ignoreForNormalization X. Duplicates as marked by Picard from the bam files were removed.

### Isolation of FLS and bone marrow macrophages

RA-FLS and OA-FLS were isolated by enzymatic digestion using the collagenase type IV (Worthington Biochemicals) and cultured in 10% heat-inactivated FCS-supplemented Dulbecco’s modified Eagle’s medium at 37 °C and 5% CO_2_. Murine FLS were isolated from hind paws of different genotypes. Paws were digested using collagenase type IV (Worthington Biochemicals) in DMEM for 1.5 h. Cell suspension was centrifuged at 405 × *g* for 5 min. The pellet was resuspended with DMEM and FLS were cultured under standard conditions. To eliminate initial contaminations with other cells, only cells at passages 3 to 5 were used for experiments. Additionally, the cell pellet after collagenase incubation was resuspended in DMEM and stained with anti-mouse CD11b APC (BioLegend clone M1/70, dilution 1:100) and CD14 AF647 (BioLegend #123327, dilution 1:100) antibodies and analysed using a flow sorter *FACS Canto II* from BD Biosciences.

Furthermore, all legs from hTNFtg and hTNFtg*/Lasp1*^*−/−*^ mice were dissected and tibia and femur with intact joints were digested for 1 h at 37 °C in DMEM containing 1 mg/ml Collagenase D (Worthington) and 10 mg/ml of DNase I (Sigma-Aldrich). Cells were centrifuged and the pellet resuspended in Red Bood Lysis Buffer (Sigma-Adrich) and washed with MACS buffer (20 mM EDTA, 5% BSA, PBS). In addition, cells were excluded using Zombie Violet staining (BioLegend). Cells were stained at 4 °C in MACS buffer using the following antibodies: anti-CD45 PerCP (BioLegend #30-F11, dilution 1:100), anti-CD31 Alexa488 (eBioscience clone 390, dilution 1:100), anti-THY1.2 APC (Invitrogen #17-0902-81, dilution 1:50), anti-Podoplanin PE (Invitrogen clone 8.1.1, dilution 1:100). Samples were acquired using a flow cytometer (Cytoflex S, Beckman Coulter, Krefeld Germany) and analysed by FlowJo Version 10.6.1 (FlowJo LLC, Ashland, OR, USA).

Additionally, bone marrow macrophages (BMM) were isolated from 8-weeks-old mice. Femora and tibiae were removed and the bone marrow was flushed out of the bone marrow cavity using α-MEM containing 10% FCS. Differentiation of BMM to osteoclasts was achieved by culturing the cells in α-MEM medium supplemented with 10% FCS and 30 ng/ml M-CSF (R&D) for two days followed by incubation in presence of 30 ng/ml M-CSF (R&D) and 50 ng/ml RANKL (R&D) for another five days. Furthermore, the sera from arthritic mice were taken and the human TNF levels were measured via an ELISA Kit (R&D Systems). The assay was performed according to the manufacturer’s protocol.

### Isolation of MEFs

MEFs were isolated from pregnant mice between 12.5 and 13.5 days after coitum. Embryos were released from the placenta. Then, heads and internal organs were removed from all embryos, followed by digesting with 0.25% Trypsin/EDTA in PBS for 5 min by 37 °C. Adding culture medium inhibited the trypsin activity and this cell suspension was further centrifuged at 405 × *g* for 5 min. The supernatant was discarded and the pellet was resuspended in DMEM and MEFs were then cultured under standard conditions^47^.

### **Isolation of osteoblasts**

Osteoblasts were isolated from 12-weeks-old wt and hTNFtg mice. Femora and tibiae were removed and the bone marrow was flushed out. Bones were crushed and digested using 0.1% collagenase B (Sigma) and 0.2% dispase grade II (Roche) in α-MEM. Cells were seeded at 15 × 10^4^ cells per well in 6-well plates. Confluent pre-osteoblasts (after 24 to 48 h) were subjected to differentiation medium (α-MEM supplemented with 0.2 mM L-ascorbate-2-phosphate, 10 nM dexamethasone and 10 mM glycerolphosphate). After differentiation cells were lysed and analysed via Western Blot.

### 3D synovial organ cultures

Passaged FLS were resuspended in an ice-cold Matrigel-Matrix (BD Biosciences). Droplets of the suspension were placed onto poly-2-hydroxyethyl-methacrylate (poly-HEMA; Aldrich Chemical Company, Milwaukee)-coated culture dishes and cultured for 17 days under standard conditions. Thereafter, the three-dimensional culture was fixed with 2% (w/v) paraformaldehyde in HBS containing 1 mM CaCl_2_ for 2 h, rinsed with ethanol several times, and embedded in paraffin^17^. Sections of the tissue-like structure were stained with haematoxylin and eosin.

### Cell motility assay and CyQuant cell proliferation assay

Cell migration was evaluated by a modified wound scratch assay. Cells were plated into cell culture dishes from ibidi_®_. At a cell confluence of 90% the chambers were removed and the cells were overlaid with culture medium. The images (magnification 100x) were acquired immediately after removing the chambers and at the complete closure of the gap using an inverted microscope as well as live-cell microscope. The quantification was performed with the *Axio Vision 4.8* software. CyQuant Cell Proliferation Assay Kit (Thermo Fisher Scientific) was used to measure the cellular DNA content via fluorescent dye binding. The assay was performed according to the manufacturer’s protocol.

### Analysis of the cytoskeleton of FLS by transmission electron microscopy

 FLS were seeded on sterile coverslips in FCS-supplemented DMEM until 80% confluence. The cytoskeleton was stabilised with 2 μM Taxol (Sigma-Aldrich) and 2 μM Phalloidin (Sigma-Aldrich) using a specific extraction buffer. Furthermore, the cytoskeleton was dehydrated and after drying with hexamethyldisilazane (HDMS) coated with platinum and carbon and analysed by transmission electron microscopy (TEM)^48^. Specific proteins such as β-Actin (Abcam #8227, dilution 1:200), β-Catenin (Cell Signaling #9562, dilution 1:200) and Lasp1 (Abcam #ab130109, dilution 1:600) were stained using immunogold particles different in size (12 and 18 nm).

### Electric cell/substrate impedance sensing assay

FLS were seeded in ECIS arrays with FCS-supplemented DMEM until 80% confluence. A typical electric cell/substrate impedance sensing assay (ECIS) array consists of 10 active in parallel working electrodes and a counter electrode much bigger in surface area. A non-invasive alternating current (<1 μA) is applied to the electrodes. ECIS detects the associated voltage drop across the system and determines the electrical resistance of the cell-covered electrodes^49^.

### Lentiviral gene transfer and live-cell imaging

Expression of Life-Act green fluorescent protein (EGFP) was performed by lentiviral gene transfer^19^. FLS were seeded on glass-bottom dishes in FCS-supplemented DMEM until 80% confluent and infected with replication-deficient lentivirus carrying Life-Act-EGFP or a β-Catenin-Halo construct^19^. This construct has a self-labelling tag, which gets active by adding 5 μM of a Halo Tag^®^ TMR ligand (Promega) 15 min before the imaging studies. After 2 to 3 days, cells displayed a sufficient signal usable to perform fluorescent live-cell imaging by *confocal microscopy LSM 700* (Zeiss) at 37 °C and 5% CO_2_.

### Transfection of *Lasp1*^*−/−*^ FLS or MEFs

*Lasp1* knockout FLS or MEFs were seeded into 10 mm dishes in FCS-supplemented DMEM until 80% confluence. We performed the transfection via jetPRIME (Polyplus-transfection, Illkirch, France) according to the manufacturer’s protocol. The cells were stained or lysed in NP-40 buffer containing phosphatase as well as protease inhibitors (Sigma-Aldrich), 48 h after transfection.

### Co-Immunoprecipitation

Co-Immunoprecipitations (Co-IP) were performed using Dynabeads (Life Technologies) according to the manufacturer’s protocol. β-Catenin (BD Biosciences #610153, dilution 1:1000) or Lasp1 antibody (Abcam #ab130109, dilution 1:1000) was coupled to protein G–labelled Dynabeads. Equal amounts of cell lysates were transferred to the antibody-bead complex and incubated overnight at 4 °C. Antibodies against the following proteins were detected by immunoblotting: β-Actin (Abcam #ab8227, dilution 1:1000), β-Catenin (Cell Signaling #9562, dilution 1:1000), Cadherin-11 (Life Technologies #321700, dilution 1:1000), Lasp1 (Abcam #ab130109, dilution 1:2000), p120-Catenin (BD Biosciences #610134, dilution 1:1000) and GAPDH (Cell Signaling #3638 S, dilution 1:1000).

### Stimulation experiments

For stimulation experiments, FLS were seeded in 6-well plates, incubated with FCS-supplemented DMEM until 80% confluence and were then serum starved for 24 h. To examine signalling pathways cells were stimulated either with 10 ng/ml recombinant PDGF for 0, 10, 15 and 30 min or with 10 ng/ml recombinant murine IL-1β for the indicated time periods. Experimental controls were treated with PBS. Antibodies against the following proteins were detected by immunoblotting: pSrc_418_ (Novus Biologicals #100-92633, dilution 1:1000), total Src (R&D Systems, #AF3389, dilution 1:1000), pAKT_473_ (Cell Signaling, #4060 S, dilution 1:1000) and total AKT (Cell Signaling #9272, dilution 1:1000).

#### Quantitative real-time PCR

Total RNA of FLS from 12-weeks-old wt and hTNFtg mice were isolated via the RNeasy Mini-Kit (Qiagen, Germantown, Maryland, USA) according to the manufacturer’s instructions. RNA concentrations were measured using a Nanodrop NanoVue Plus Spectrophotometer (Th. Geyer, Esslingen, Germany). The isolated RNA (1 μg) was used for cDNA synthesis using the First Strand cDNA Synthesis Kit (Thermo Scientific, Waltham, Massachusetts, USA). For quantitative real-time PCR, 1 μg of synthesised cDNA per sample was analysed with IQ^TM^ SYBR Green Supermix (Bio-Rad, Hercules, California, USA) according to the manufacturer’s instructions and evaluated by Bio-Rad iQ5 programme (version 2.1). All samples were normalised to Actb mRNA. The relative expression of *Lasp1* mRNA was calculated using the delta-delta CT method. The primers used are listed in Suppl. Table 3.

#### ELISA experiments

3 × 10^5^ FLS were seeded with FCS-supplemented DMEM in 24-well plates and were then serum starved for 24 h. To investigate the MMP3 and MMP9 expression levels, cells were stimulated with 10 ng/ml recombinant human TNF and levels were measured via an ELISA Kit (R&D Systems). Furthermore, the RANKL as well as OPG expression levels were examined using ELISA Kits (R&D Systems). All assays were performed according to the manufacturer’s protocol.

### Histological methods

Human samples from OA and RA patients as well as the right hind paws from mice were fixed in 4% PFA at 4 °C overnight. Afterwards the samples were decalcified for eight weeks in Na-EDTA, pH 7.2 (weekly changed). Human tissue and decalcified mouse tissue were washed with PBS, dehydrated in a tissue processor and poured in paraffin via an embedding machine. Paraffin sections into 5 µm slices were performed with a *Microtome HM355S (Thermo Fisher Scientific)* and used for specific immunohistological and/or toluidine-blue stainings.

### Immunohistochemistry

Human and murine tissue sections were deparaffinised with xylene and were then incubated in 100%, 96%, 80% and 70% ethanol. After incubation in *aqua dest*, sections were washed with PBS. The tissue was incubated with 1x Trypsin/EDTA in a humid chamber for 10 min at 37 °C. After digestion, the probes were washed with PBS. To quench the endogenous peroxidases, the slices were treated with 30% H_2_O_2_ in methanol. Afterwards the tissue was blocked in 20% horse serum for an hour. Sections were stained with a monoclonal antibody to Lasp1 (Abcam #ab130109, dilution 1:200) or control immunoglobulin G (R&D Systems #M-AB-002) overnight by 4 °C. Immunostainings were performed using the secondary biotinylated antibody in combination with the peroxidase technique (Vectastain ABC-system) together with a DAB substrate (Vector Laboratories) substrate according to the manufacturer’s protocol. Slices were shortly washed with PBS followed by 100% ethanol, xylene and finally embedded in DPX.

### Haematoxylin and eosin staining of paraffin sections

Sections from mouse tissue were deparaffinised with xylene and were then incubated in 100, 96, 80 and 70% ethanol. After incubation in *aqua dest*, sections were incubated in Mayer’s Haemalaun (Sigma-Aldrich), washed in running tap water and then counterstained with an alcoholic solution of eosin Y (Sigma-Aldrich). In a next step slices were shortly incubated in ethanol (ethanol-series: 70, 80, 96 and 100%), followed by xylene and subsequently embedding using DPX. Following the staining, sections were used for synovial cell density evaluations by single-blinded counting of cell numbers per high power field (HPF, 10.000 µm^2^).

### Toluidine-blue staining of paraffin sections

Sections from mouse tissue were deparaffinised with xylene and were then incubated in 100, 96, 80 and 70% ethanol. After incubation in *aqua dest*, slices were stained in toluidine-blue and afterwards washed with *aqua dest*. In a next step slices were shortly incubated in ethanol (ethanol-series: 70, 80, 96 and 100%), followed by xylene and subsequently embedding using DPX.

### Immunofluorescence stainings of FLS

FLS were seeded on sterile coverslips in FCS-supplemented DMEM until 80% confluence. Then the cells were washed in PBS and fixed with 4% PFA, pH 7.4 for 20 min. In a next step cells were washed and quenched with 100 mM NH_4_Cl. Afterwards, cells were permeabilised with 0.1% Tween 20, followed by washing with PBS. Next the cells were treated with blocking solution containing 10% normal horse serum for an hour. All specimens were stained with a primary antibody and a labelled secondary Alexa Fluor 488 antibody (Life Technologies #A11008, dilution 1:5000 or Life Technologies #A11034, dilution 1:5000). Nuclei were stained using 4′,6-diamidino-2-phenylindole (DAPI) (Invitrogen #D1306, dilution 1:10.000), and the cytoskeleton was stained using Rhodamine-Phalloidin. To analyse cell-to-cell contacts we used following antibodies: polyclonal antibody to β-Catenin (Cell Signaling #9562, dilution 1:200), monoclonal antibody to Cadherin-11 (Life Technologies #321700, dilution 1:50), monoclonal antibody to Lasp1 (Abcam #130109, dilution 1:600), polyclonal antibody to Paxillin (Abcam #ab32084, dilution 1:100), monoclonal antibody to p120-Catenin (BD Biosciences #610134, dilution 1:100).

#### Immunofluorescence stainings of tissue

Frozen tissue sections from mouse hind paw joints were cut in 6 μm thick sections, washed in PBS, incubated for 30 min in 10% normal horse serum/PBS and incubated over night at 4 °C with primary antibodies, rabbit polyclonal to FAP (Abcam #ab53066, dilution 1:500), syrian hamster monoclonal to Podoplanin (Invitrogen clone 8.1.1, dilution 1:100) and rat monoclonal to THY1.2 (Invitrogen #14-0902-82, dilution 1:200). After washing with PBS, slices were incubated with secondary antibodies anti-hamster 647 (Abcam #ab173004, dilution 1:5000), anti-rat 488 (Life Technologies #A-11006, dilution 1:5000) and anti-rabbit 568 (Life Technologies #A-110011, dilution 1:5000) for 30 min, counterstained with DAPI (Invitrogen #D1306, dilution 1:10.000) and mounted in aqueous mounting media. Additionally, tissue sections from mouse hind paws were incubated over night at 4 °C with the primary antibody, rabbit polyclonal to Osterix (Abcam, #22552, dilution 1:1000).

### TRAP staining

Tartrate-resistant acid phosphatase (TRAP) staining on differentiated BMMs was performed for the detection of osteoclasts using a leukocyte acid phosphatase staining Kit (Sigma-Aldrich) according to the manufacturer’s protocol.

### Histomorphometric analysis

Histological analysis was performed on toluidine-blue stained paraffin sections from tarsal hind paws. Synovial pannus formation, length of attachment of synovial tissue to the cartilage and proteoglycan loss was quantified using the *Axio Vision 4.1* software. The percentage values of synovial pannus formation were calculated as a sum of pannus areas in tarsal joints in relation to the total tarsal joint area. The percentage values of total cartilage were calculated as a sum of cartilage in tarsal joints in relation to the total tarsal joint area. Destained cartilage was calculated by the ratio of destained (destroyed) cartilage to total toluidine-blue stained, intact cartilage^50^.

### Micro-computed tomographic analysis

Right hind paws of different mice were fixed in 4% PFA for 24 h followed by washing with PBS. The scans were examined via the *SkyScan 1176* scanner (Bruker). Scanning was performed at 40 kV, 600 μA, 0.2 filter and 0.5° rotation steps. Reconstruction and analyses of images were carried out using the manufacturer’s software (*Nrecon V1.7.4.2, CTvox version 3.3.0r1401*).

### Image data analysis

Images were processed using build-in functions of ImageJ/Fiji (Version 1.49p). Immunohistochemistry images of Lasp1 expression in tissue were quantified via the ImageJ/Fiji plugin called Colour Deconvolution as published by Ruifrok and Johnston^51^. This plugin enables to separate the image into the different channels. In order to analyse the intensity of the image, we set a threshold and ROI for each image. We were therefore able to measure the specific intensity per area. For three-dimensional culture, cell localisation was super-imposed with colour-coded density mapping and cell distribution was quantified via radial intensity analysis. For lamellipodial extension dynamics, velocity was extracted from line-scans perpendicular to the cell edge. Cell-to-cell and cell-matrix structures were assessed by grey-value distribution along cell boundaries.

### Statistical analysis

Bar-graphs presented as mean ± SEM. Statistical analysis was performed via the *GraphPad Prism* Software, version 9 (GraphPad Software Inc.). Differences between groups were investigated for statistical significance using two-tailed Mann–Whitney *U* test.

### Reporting summary

Further information on research design is available in the Nature Research Reporting Summary linked to this article.

## Supplementary information

Supplementary Information

Descriptions of Additional Supplementary Files

Supplementary Movie 1

Supplementary Movie 2

Supplementary Movie 3

Supplementary Movie 4

Supplementary Movie 5

Supplementary Movie 6

Reporting summary

## Data Availability

Human sequencing data shown in Fig. 1a, Suppl. Fig. 2 were accessed in Gene Expression Omnibus (GEO) with the primary accession code GSE112658. Mouse sequencing data shown in Fig. 1c–f and Suppl. Figs. 3, 4 have been deposited in the European Genome-Phenome Archive under the accession codes ERS2369617, ERS2369618, ERS2369619, and ERS2369620. All other data supporting the findings of this study are present in the article and its Supplementary files of from the corresponding author upon reasonable request. Source data are provided with this paper.

## Source data

Source Data
