# Supplementary material for: Lasp1 regulates adherens junction dynamics and fibroblast transformation in destructive arthritis
Source: Nat Commun. 2021 Jun 15;12:3624. doi: 10.1038/s41467-021-23706-8 (PMC8206096; doi:10.1038/s41467-021-23706-8)
Supplement: Supplementary file 1 — Supplementary Information [file 41467_2021_23706_MOESM1_ESM.pdf]

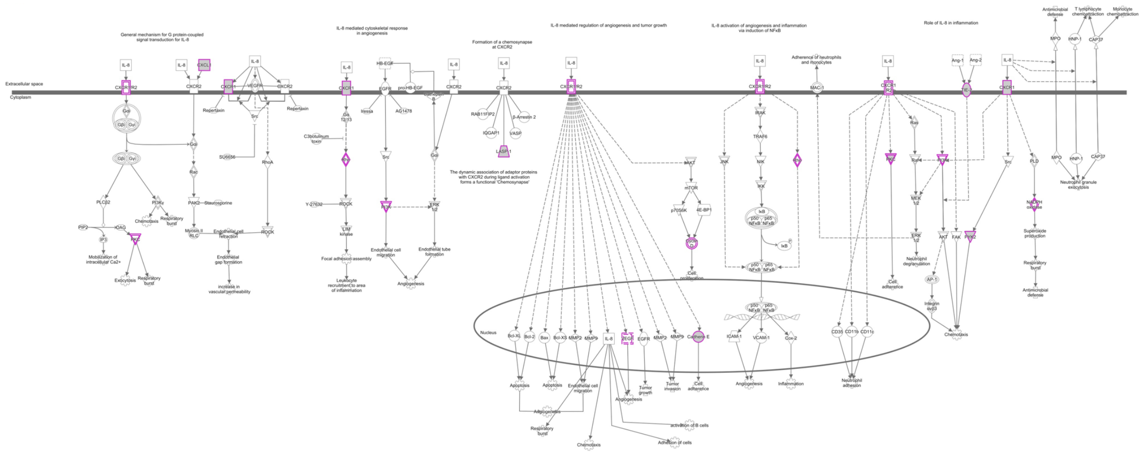

Supplementary Figure 1. The ingenuity pathway of "IL-8 signaling". Differentially modified molecules are indicated in magenta color. Legends: □Cytokine; ▯ G-protein coupled receptor; ▽Kinase; ⬭transcription regulator; ◇enzyme; ⬯ transmembrane receptor; ⬚ growth factor; ⬡peptidase; ⏢ transporter; ○other; double-sided shapes: complex/group. The pathway was generated through the use of QIAGEN's Ingenuity Pathway Analysis (IPA®, QIAGEN, www.qiagen.com/ingenuity).

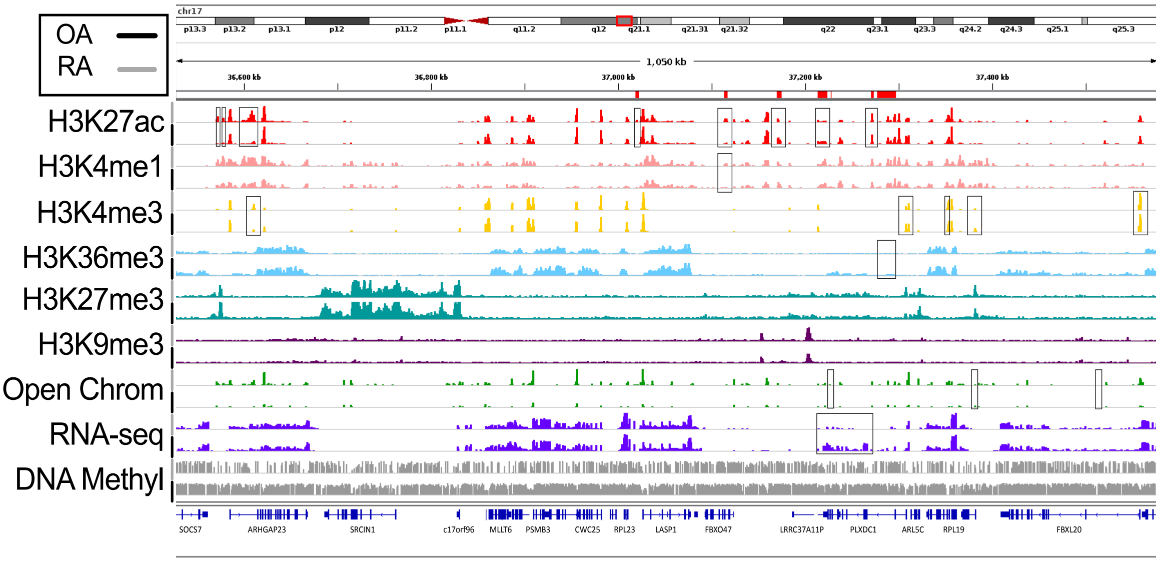

Supplementary Figure 2. Epigenomic landscape of human RA FLS with six histone modifications, open chromatin, RNA-seq and DNA methylation. The figure shows an example of the relative signal intensity across ±500 kb regions of *LASP1* gene for each mark in RA and OA FLS. In addition, the differentially marked regions between RA-FLS and OA-FLS are labeled.

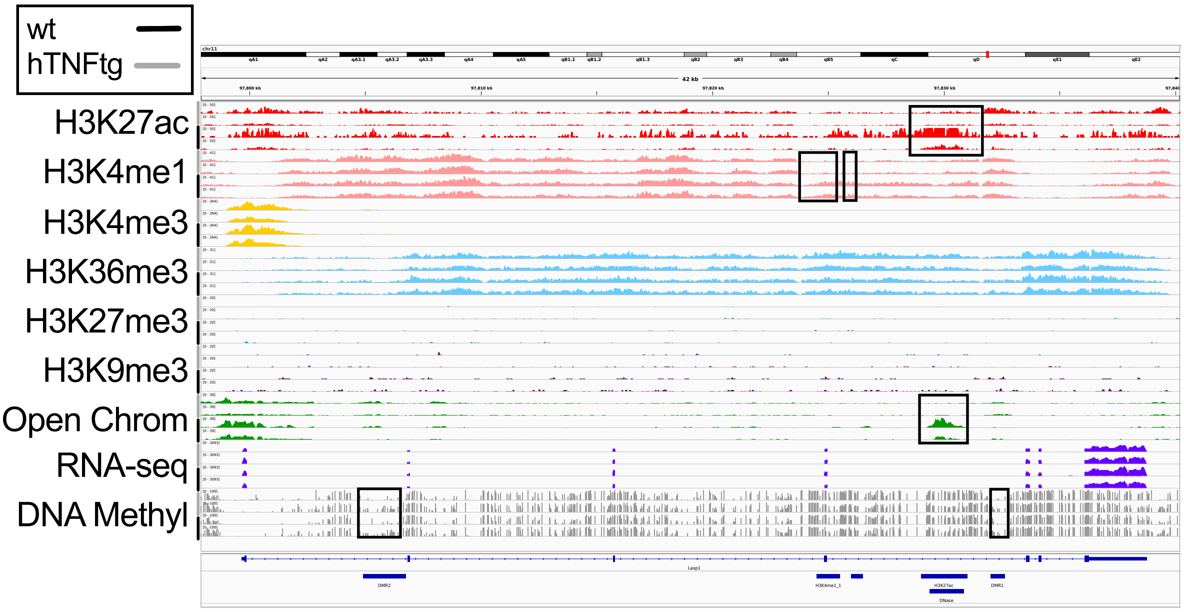

Supplementary Figure 3. Epigenomic landscape of the murine *Lasp1* gene with 6 histone modifications, open chromatin, RNA-seq and DNA methylation. The figure shows the relative signal intensity (normalized the bigwigs using DeepTools2 bamCoverage) in FLS from 2 hTNFtg and 2 wt animals. In addition, the differentially marked regions between wt and hTNFtg FLS are labeled.

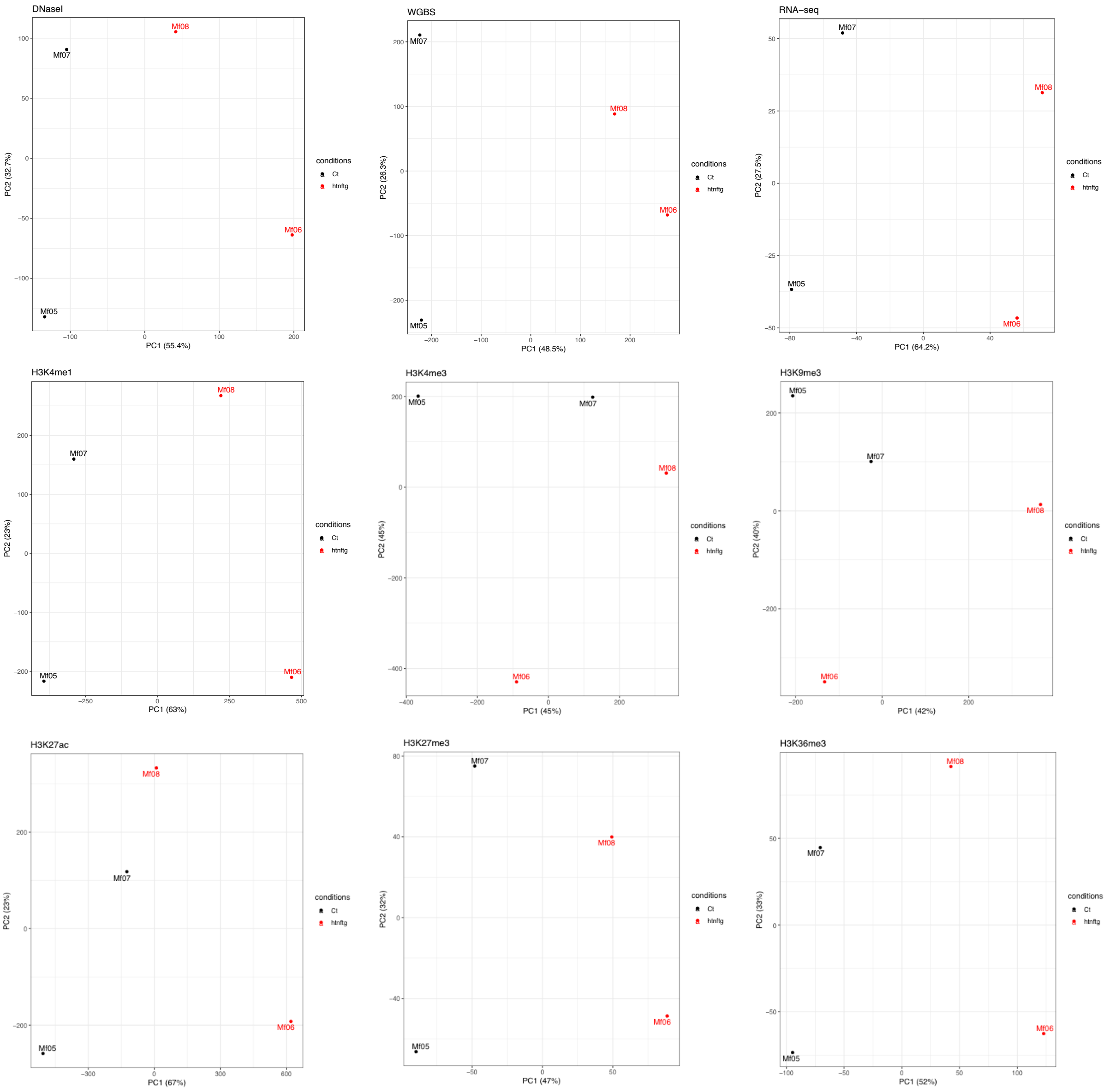

Supplementary Figure 4. The figure shows all PCA plots with variances in percent demonstrating that both conditions (wt and hTNFtg) cluster together.

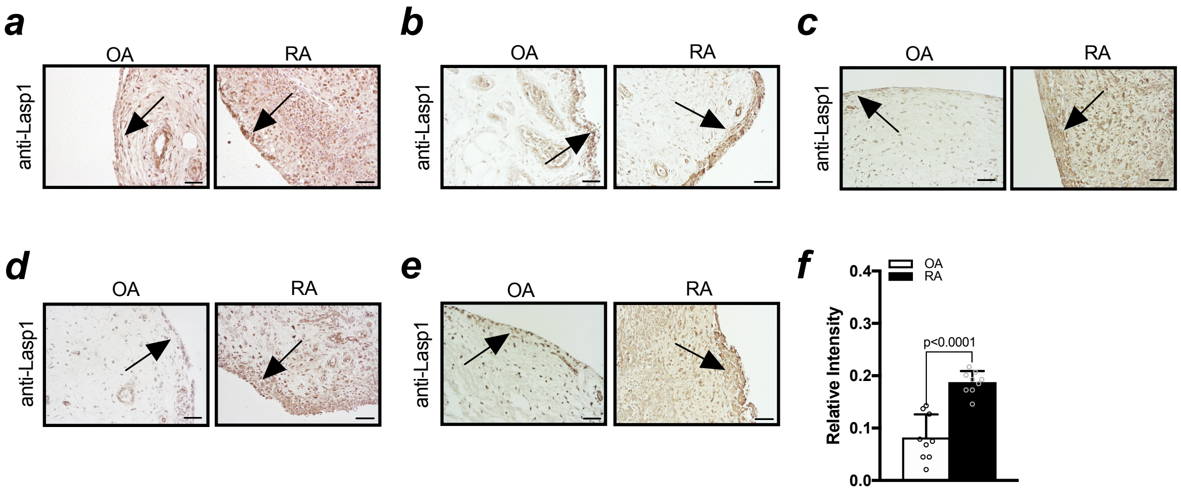

Supplementary Figure 5. Immunohistochemical staining and quantification for Lasp1 expresion in synovial tissues from human patients with RA and OA (representative images of n≥3 per group, scale bar: 50 μm). All data presented as mean ± SEM, * $P < 0.05$ (two-tailed Mann-Whitney *U* test).

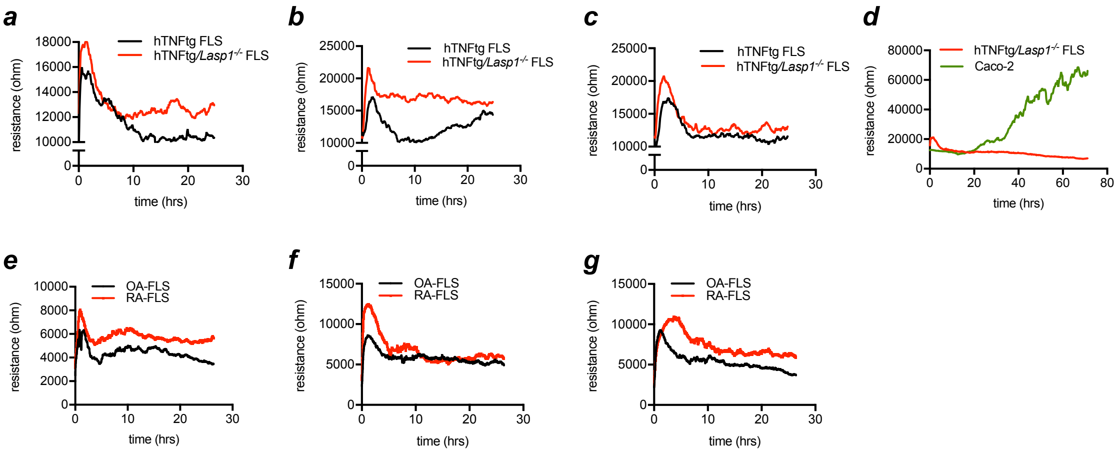

Supplementary Figure 6. Establishment of cell-to-cell contacts was analyzed by electric cell/substrate impedance sensing (ECIS) and the total resistance reflecting the strength of cell-to-cell contacts was monitored over a time in independent experiments. **a) – c)** ECIS studies using hTNFtg FLS and hTNFtg/*Lasp1*$^{-/-}$ FLS. **d)** ECIS measurements with hTNFtg/*Lasp1*$^{-/-}$ FLS and human epithelial Caco-2 cells. **e) – g**) ECIS studies using RA-FLS and OA-FLS.

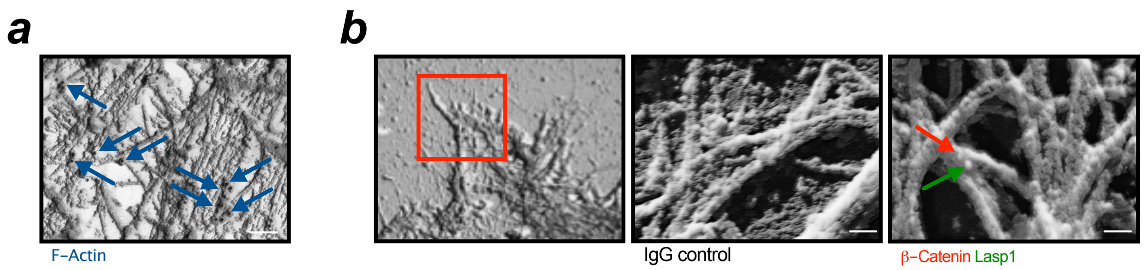

Supplementary Figure 7. Co-localization between Lasp1 and β-Catenin using TEM.
a) Demonstration of the F-Actin cytoskeleton in FLS using transmission electron microscopy (TEM). The cytoskeleton from wt FLS was fixed, stabilized with Taxol and Phalloidin and detected with an immunogold labelled F-Actin antibody (representative image of n=3 independent analyses, scale bar: 100 nm). **b)** Demonstration of Lasp1 and β-Catenin interaction in wt FLS with immunogold labelled antibodies as assessed by TEM (representative image of n=3 independent analyses, scale bar: 100 nm).

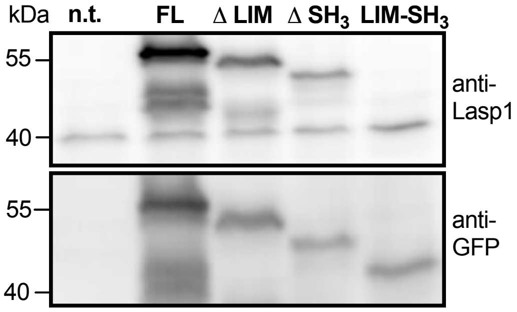

Supplementary Figure 8. Immunoblotting for Lasp1 and GFP expression in Lasp1$^{-/-}$ MEFs after 48 hours of transfection with different Lasp1 expression constructs (n=3 each group): Lasp1 full length (FL), a deletion construct missing the LIM domain (Δ LIM), a deletion construct missing the SH3 domain (ΔSH3) and a deletion mutant without the F-Actin binding nebulin-repeats (LIM-SH3). Non-transfected (n.t.) MEFs served as controls.

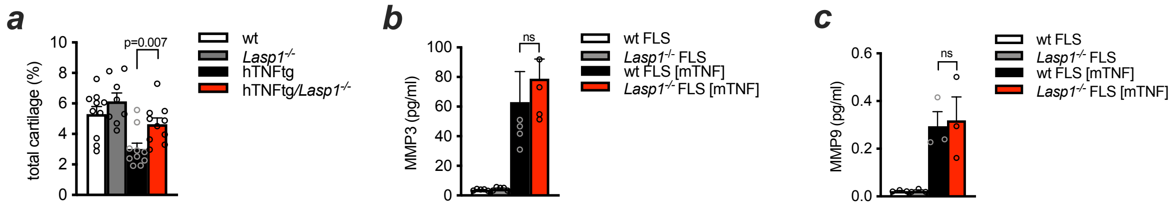

Supplementary Figure 9. a) Histomorphometric analysis of total cartilage area in tarsal joints of hTNFtg and hTNFtg/*Lasp1*$^{-/-}$ female mice (n≥4 each group). All data presented as mean ± SEM, * P < 0.05, ** P < 0.01 (two-tailed Mann-Whitney *U* test). **b)** Evaluation of MMP3 levels following TNF stimulation (n=5 each group). All data presented as mean ± SEM, * P < 0.05 (two-tailed Mann-Whitney *U* test). **c)** Evaluation of MMP9 levels following TNF stimulation (n=3 each group). All data presented as mean ± SEM, * P < 0.05 (two-tailed Mann-Whitney *U* test).

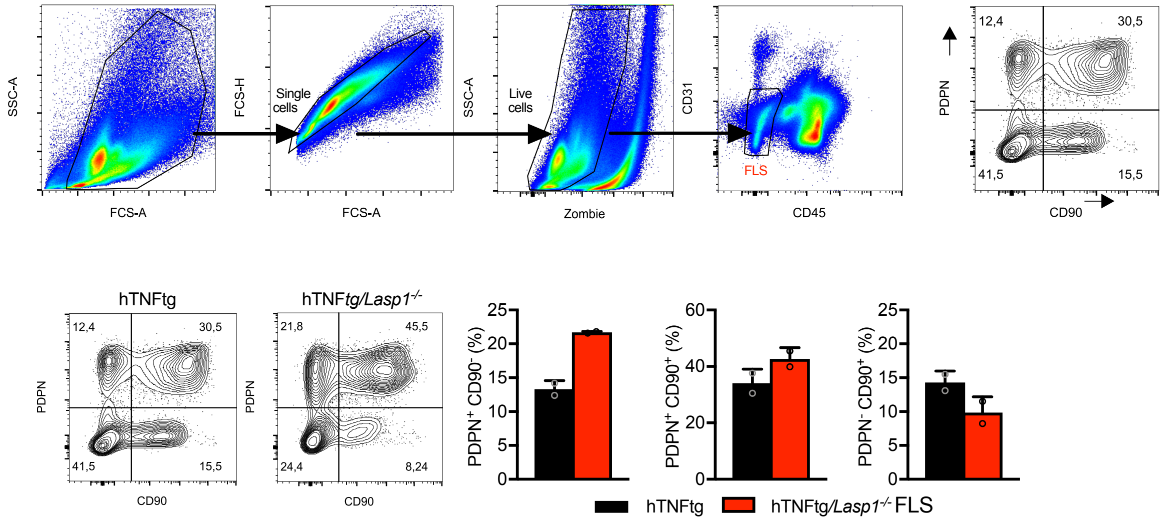

Supplementary Figure 10. Profiling of fibroblast-like synoviocytes (FLS) in lining (LL) and sublining (SL) layers of the synovium. 1.) SSC-A vs. FSC-A gating exclude debris. 2.) FSC-H vs. FSC-A gating to exclude doublets. 3.) SSC-A vs. Zombie gating to exclude dead cells. 4.) CD31 vs. CD 45 gating to quantify FLS. 5.) Identification of FLS subpopulations by Podoplanin (PDPN) and CD90 staining (representative images of n=2 independent experiments).

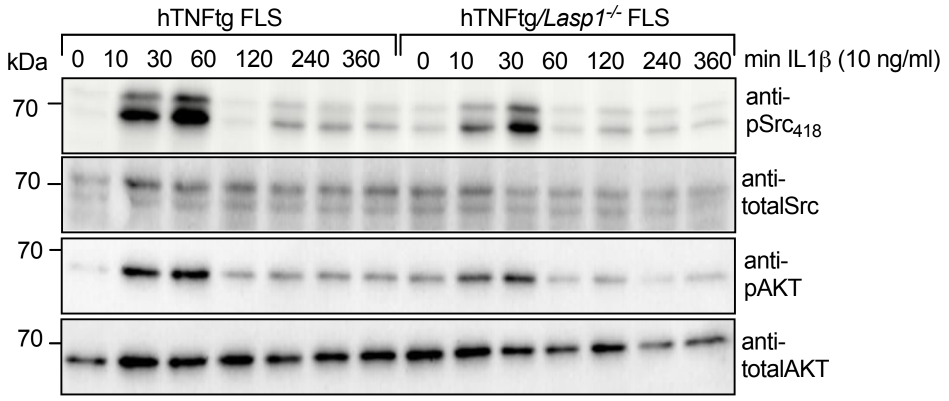

Supplementary Figure 11. Immunoblotting for phospho-specific Src kinase ($pSrc_{418}$) and total Src kinase expression as well as for phosho-specific AKT kinase (pAKT) and total AKT in FLS from hTNFtg and hTNFtg/*Lasp1*$^{-/-}$ mice following recombinant murine IL-1β stimulation for the indicated time periods (n=3 each group).

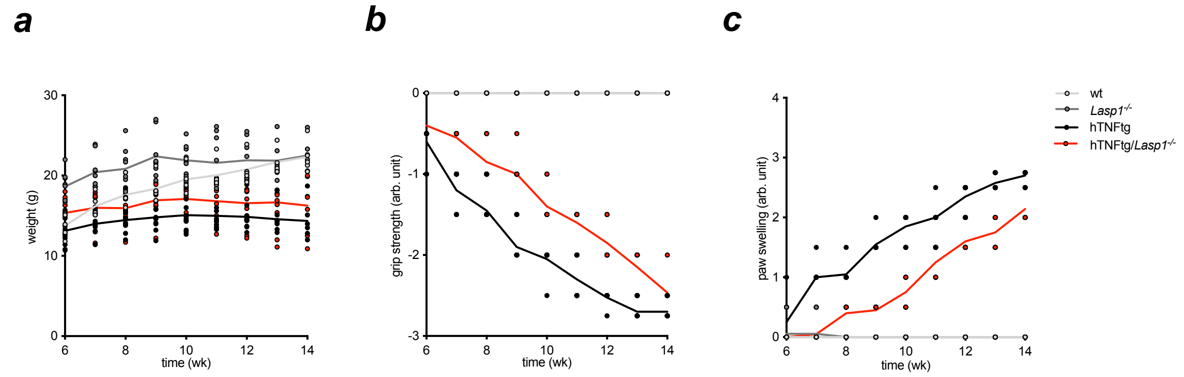

Supplementary Figure 12. Evaluation of clinical arthritis symptoms such as weight, grip strength reduction and paw swelling development over the disease course of wt, hTNFtg*, Lasp1$^{-/-}$* and hTNFtg/*Lasp1$^{-/-}$* mice. **a) – c)** Each dot represents a single mouse, in **b)** and **c)** the reduced dot number is caused by data overlay.

Supplementary Table 1

Primers for genotyping

Genotype	Forward primer sequence (5'-3')	Reverse primer sequence (5'-3')
Lasp1*$^{-/-}$*	CACACTCGCGTCTGTTTCTCCAGC	GCACCTCTAACCTCCTGCACACACC

PCR conditions
95°C 1 min
35x (68°C 1 min, 72°C 2 min)
72°C 6 min
10°C ∞

Genotype	Forward primer sequence (5'-3')	Reverse primer sequence (5'-3')
**hTNFtg**	TACCCCCTCCTTCAGACACC	GCCCTTCATAATATCCCCCA
**hTNFtg wt**	GAGGGCCGAAGCTGCGGCTGGGT	GGTGGCGATTGGCTTGGCGAAG

PCR conditions
95°C 30 s
35x (58.5°C 30 s, 72°C 45 s)
72°C 5 min
10°C ∞

Supplementary Table 2

Primers for sequencing

DMR	PCR target
**1**	chr11:97,831,969-97,832,197
**2**	chr11:97,805,999-97,806,286

DMR	Forward primer sequence (5'-3')	Reverse primer sequence (5'-3')
**1**	cttgcttcctggcacgagAGGGAGAGAGAGGTTTGATTTTATT	caggaaacagctatgacCCCCACCCTTAACATTCTCTATAC
**2**	cttgcttcctggcacgagTGTTAGGGGTAGAGGATTTTTTATG	caggaaacagctatgacTATATACCTCAACCACACCCATATC

1st PCR conditions
95°C 15 min
40x (Ta 61°C, C95°C 30 s, 72°C 1 min)
72°C 10 min

2nd PCR conditions
95°C 15 min
35x (Ta 60°C, 95°C 30 s, 72°C 1 min)
72°C 10 min

Ta = annealing temperature

Supplementary Table 3

Primers for Sequences used for Quantitative Real-Time PCR

Gene	Primer sequence (5'-3')
**m_*Lasp1*_for**	TCT CCG CCT CAA GCA ACAG
**m_*Lasp1*_rev**	CTG ATC TGG TC CTG GGT CTC C
**m_GAPDH_for**	AGC AAG GAC ACT GAG CAA GAG AGG
**m_GAPDH_rev**	GGG TCT GGG ATG GAA ATT GTG AGG
**m_Hprt_for**	AGC TAC TGT AAT GAT CAG TCAA CG
**m_Hprt_rev**	AGA GGT CCT TTT CAC CAG CA

qPCR conditions
95°C 3 min
40x (95°C 30 s, 72°C 45 s)
72°C 5 min

for = forward
rev = reverse
