# Supplementary material for: Lasp1 regulates adherens junction dynamics and fibroblast transformation in destructive arthritis
Source: Nat Commun. 2021 Jun 15;12:3624. doi: 10.1038/s41467-021-23706-8 (PMC8206096; doi:10.1038/s41467-021-23706-8)
Supplement: Supplementary file 2 — Descriptions of Additional Supplementary Files [file 41467_2021_23706_MOESM2_ESM.pdf]

Description of Additional Supplementary Files

Supplementary Movie 1 and 2 (Figure 3a)

Description: Lasp1 knockout reduced the migration rate in FLS. FLS from hTNFtg and hTNFtg/Lasp1-/- FLS were seeded into cell culture dishes from ibidi®. When a cell confluence of 90% was reached, the chambers were removed and the migration rates were recorded via timelapse microscopy for 24 hours with an image collection interval of five minutes. The hTNFtg FLS migrated with high speed and extensive spread across the culture surface (Movie/Audio/Data 1). In contrast, hTNFtg/Lasp1-/- FLS migrated very slowly, with no extensive spreading and striking differences in migration morphology and cell shape (Movie/Audio/Data 2).

Supplementary Movie 3 and 4 (Figure 3f)

Description: Dynamics of cell-to-cell contacts in FLS. 7*103 FLS from hTNFtg and hTNFtg/Lasp1-/- mice were transduced with a lentiviral Life-Act-GFP construct. The establishment and dynamic resolution of cell-to-cell contacts were investigated by live-cell fluorescence imaging for an hour with an image collection interval of two minutes. FLS from hTNFtg mice were observed to create contacts with adjacent cells via the formation of lamellipodia. These interactions were characterised by the arrangement of a zipper-like structure along the interacting surfaces (Movie/Audio/Data 3). The dynamics and morphology of cell-to-cell contact formation was strikingly different in hTNFtg/Lasp1-/- FLS and was characterized by more stable yet irregular cell-to-cell contacts and at the same time by the formation of large lamellipodia that failed to remodel into zipper-like structures (Movie/Audio/Data 4).

Movie 5 and 6 (Figure 4d)

Description: Arrangement of cell-to-cell contacts in FLS. 7*103 FLS from hTNFtg and hTNFtg/Lasp1-/- mice were transfected with a lentiviral β-Catenin-Halo construct. The arrangement of cell-to-cell contacts was investigated by live-cell fluorescence imaging for an hour with an image collection interval of five minutes. The hTNFtg FLS showed large numbers of cell-to-cell contacts consisting of a zipper-like structure (Movie/Audio/Data 5) in comparison to hTNFtg/Lasp1-/- FLS (Movie/Audio/Data 6).
