# Supplementary material for: Lasp1 regulates adherens junction dynamics and fibroblast transformation in destructive arthritis
Source: Nat Commun. 2021 Jun 15;12:3624. doi: 10.1038/s41467-021-23706-8 (PMC8206096; doi:10.1038/s41467-021-23706-8)
Supplement: Supplementary file 9 — Reporting summary [file 41467_2021_23706_MOESM9_ESM.pdf]

nature research

Corresponding author(s): Korb-Pap, Adelheid

Last updated by author(s): 2.5.2021

Reporting Summary

Nature Research wishes to improve the reproducibility of the work that we publish. This form provides structure for consistency and transparency in reporting. For further information on Nature Research policies, see Authors & Referees and the Editorial Policy Checklist .

Statistics

For all statistical analyses, confirm that the following items are present in the figure legend, table legend, main text, or Methods section.

n/a	Confirmed	
☐	☒	The exact sample size (*n*) for each experimental group/condition, given as a discrete number and unit of measurement
☐	☒	A statement on whether measurements were taken from distinct samples or whether the same sample was measured repeatedly
☐	☒	The statistical test(s) used AND whether they are one- or two-sided<br>*Only common tests should be described solely by name; describe more complex techniques in the Methods section.*
☐	☒	A description of all covariates tested
☐	☒	A description of any assumptions or corrections, such as tests of normality and adjustment for multiple comparisons
☐	☒	A full description of the statistical parameters including central tendency (e.g. means) or other basic estimates (e.g. regression coefficient) AND variation (e.g. standard deviation) or associated estimates of uncertainty (e.g. confidence intervals)
☐	☒	For null hypothesis testing, the test statistic (e.g. *F*, *t*, *r*) with confidence intervals, effect sizes, degrees of freedom and *P* value noted<br>*Give P values as exact values whenever suitable.*
☒	☐	For Bayesian analysis, information on the choice of priors and Markov chain Monte Carlo settings
☐	☒	For hierarchical and complex designs, identification of the appropriate level for tests and full reporting of outcomes
☒	☐	Estimates of effect sizes (e.g. Cohen's *d*, Pearson's *r*), indicating how they were calculated

Our web collection on statistics for biologists contains articles on many of the points above.

Software and code

Policy information about availability of computer code

Data collection	FACS Canto II, Cytoflex S flow cytometer (Beckman Coulter), Electron microscope Phillips EM-410 (Ditabis, Pforzheim, Germany), ECIS™ Model 1600R (Applied BioPhysics, Troy, NY), LSM 700 confocal microscopy (Zeiss), SkyScan 1176 scanner (Bruker)
Data analysis	Data analysis: deeptools 2.0 package, bamCoverage tool 3.4.3, FlowJo 10.6.1, Axio Vision 4.1 and 4.8, Bio-Rad iQ5 program 2.1, Nrecon V1.7.4.2, CTvox version 3.3.0r1401, ImageJ/Fiji 1.49p, GraphPad Prism Software version 9 (GraphPad Software Inc.), ECIS Data Analysis Software (Applied Biophysics, Troy, NY), Illumina HiSeq 2500 platform, normr 1.16.0

For manuscripts utilizing custom algorithms or software that are central to the research but not yet described in published literature, software must be made available to editors/reviewers. We strongly encourage code deposition in a community repository (e.g. GitHub). See the Nature Research guidelines for submitting code & software for further information.

Data

Policy information about availability of data

All manuscripts must include a data availability statement. This statement should provide the following information, where applicable:

- Accession codes, unique identifiers, or web links for publicly available datasets
- A list of figures that have associated raw data
- A description of any restrictions on data availability

The data supporting the findings of this study are available from the corresponding author upon reasonable request. The source data underlying Figs. 2a, c-e, 3b-e, g, 4b-c, 5a,b, f-h, 6a, c-i, k, 7a, c, Supplementary Figs. 6a-g, 8, 9a-c, 10, 11-15 are provided as a Source data file. Furthermore, all human sequencing data shown in Fig 1a, Suppl. Fig 2 have been accessed in Gene Expression Omnibus with the primary accession code GSE112658 (https://www.ncbi.nlm.nih.gov/geo/query/acc.cgi?acc=GSE112658). The murine sequencing data shown in Fig. 1c-f and Suppl. Fig. 3, 4 have been deposited at the European Genome-Phenome Archive under the accession numbers ERS2369617, ERS2369618, ERS2369619, ERS2369620 (https://www.ebi.ac.uk/ena/browser/view/ERS2369617, https://www.ebi.ac.uk/ena/browser/view/ERS2369618, https://www.ebi.ac.uk/ena/browser/view/ERS2369619, https://www.ebi.ac.uk/ena/browser/view/ERS2369620).

.

Field-specific reporting

Please select the one below that is the best fit for your research. If you are not sure, read the appropriate sections before making your selection.

- [x] Life sciences
- [ ] Behavioural & social sciences
- [ ] Ecological, evolutionary & environmental sciences

For a reference copy of the document with all sections, see nature.com/documents/nr-reporting-summary-flat.pdf

Life sciences study design

All studies must disclose on these points even when the disclosure is negative.

Sample size

The cell culture experiments showed high reproducibility and consistency of data. Therefore a sample size of at least n>2 was used in in vitro studies to ensure the validity of the data. In contrast, in in vivo studies a much higher sample size of n>7 was used because of higher variablity of the phenotype in the different genotypes.

Data exclusions

There were no data exclusions in this study.

Replication

All attempts of replication were successfull.

Randomization

Samples and animals were randomized regarding disease (human) or genotype (mice).

Blinding

No blinding of cells was performed in the study, as the evaluations were based on standardized quanitfication. In contrast, the evaluation of clinical parameters in the animal model as well as the analyses of histomorphopathological changes in the context of inflammatory joint destruction were blinded to the evaluator.

Reporting for specific materials, systems and methods

We require information from authors about some types of materials, experimental systems and methods used in many studies. Here, indicate whether each material, system or method listed is relevant to your study. If you are not sure if a list item applies to your research, read the appropriate section before selecting a response.

Materials & experimental systems

n/a	Involved in the study	
	x	Antibodies
x		Eukaryotic cell lines
x		Palaeontology
	x	Animals and other organisms
	x	Human research participants
x		Clinical data

Methods

n/a	Involved in the study	
	x	ChIP-seq
	x	Flow cytometry
x		MRI-based neuroimaging

Antibodies

Antibodies used

anti-pAKT 473 (Cell Signaling, #4060S), anti-Cadherin-11 (Life Technologies #321700, anti-CD11b APC (BioLegend clone M1/70), anti-CD14 AF647 (BioLegend #123327), anti-CD31 Alexa488 (eBioscience clone 390), anti-CD45 PerCP (BioLegend #30-F11), anti-FAP (Abcam #ab53066), anti-GAPDH (Cell Signaling #3638S), anti-hamster Alexa647 (Abcam #ab173004), anti-Lasp1 (Abcam #130109), anti-mouse Alexa488 (Life Technologies #A11008), anti-Osterix (Abcam, #22552), anti-p120-Catenin (BD Biosciences #610134), anti-p120-Catenin (BD Biosciences #610134), anti-Paxillin (Abcam #ab32084), anti-Podoplanin (Invitrogen clone 8.1.1), anti-Podoplanin PE (Invitrogen clone 8.1.1), anti-pSrc418 (Novus Biologicals #100-92633), anti-rabbit Alexa568 (Life Technologies #A-110011), anti-rat Alexa488 (Life Technologies #A-11006), anti-THY1.2 (Invitrogen #14-0902-82), anti-THY1.2 APC (Invitrogen #17-0902-81), anti-total AKT (Cell Signaling #9272), anti-total Src (R&D Systems, #AF3389), anti-β-Actin (Abcam #8227), anti-β-Actin (Abcam #ab8227), anti-β-Catenin (BD Biosciences #610153), anti-β-Catenin (Cell Signaling #9562)

Validation

anti-pAKT473 (Cell Signaling, #4060S, dilution 1:1000) = WB
anti-Cadherin-11 (Life Technologies #321700, dilution 1:1000) = WB
anti-Cadherin-11 (Life Technologies #321700, dilution 1:50) = ICC
anti-CD11b APC (BioLegend clone M1/70, dilution 1:100) = FACS
anti-CD14 AF647 (BioLegend #123327, dilution 1:100) = FACS
anti-CD31 Alexa488 (eBioscience clone 390, dilution 1:100) = FACS
anti-CD45 PerCP (BioLegend #30-F11, dilution 1:100) = FACS
anti-FAP (Abcam #ab53066, dilution 1:500) = IF
anti-GAPDH (Cell Signaling #3638S, dilution 1:1000) = WB

anti-hamster Alexa647 (Abcam #ab173004, dilution 1:5000) = IF
anti-Lasp1 (Abcam #130109, dilution 1:600) = IF
anti-Lasp1 (Abcam #ab130109, dilution 1:1000) = Co-IP
anti-Lasp1 (Abcam #ab130109, dilution 1:200) = IHC
anti-Lasp1 (Abcam #ab130109, dilution 1:2000) = WB
anti-Lasp1 (Abcam #ab130109, dilution 1:600) = TEM
anti-mouse Alexa488 (Life Technologies #A11008, dilution 1:5000 = ICC
anti-Osterix (Abcam, #22552, dilution 1:1000) = IF
anti-p120-Catenin (BD Biosciences #610134, dilution 1:100) = ICC
anti-p120-Catenin (BD Biosciences #610134, dilution 1:1000) = WB
anti-Paxillin (Abcam #ab32084, dilution 1:100) = ICC
anti-Podoplanin (Invitrogen clone 8.1.1, dilution 1:100) = IF
anti-Podoplanin PE (Invitrogen clone 8.1.1, dilution 1:100) = FACS
anti-pSrc418 (Novus Biologicals #100-92633, dilution 1:1000) = WB
anti-rabbit Alexa488 (Life Technologies #A11034, dilution 1:5000) = ICC
anti-rabbit Alexa568 (Life Technologies #A-110011, dilution 1:5000) = IF
anti-rat Alexa488 (Life Technologies #A-11006, dilution 1:5000) = IF
anti-THY1.2 (Invitrogen #14-0902-82, dilution 1:200) = IF
anti-THY1.2 APC (Invitrogen #17-0902-81, dilution 1:50) = FACS
anti-total AKT (Cell Signaling #9272, dilution 1:1000) = WB
anti-total Src (R&D Systems, #AF3389, dilution 1:1000) = WB
anti-β-Actin (Abcam #8227, dilution 1:200) = TEM
anti-β-Actin (Abcam #ab8227, dilution 1:1000) = WB
anti-β-Catenin (BD Biosciences #610153, dilution 1:1000) = Co-IP
anti-β-Catenin (Cell Signaling #9562, dilution 1:1000) = WB
anti-β-Catenin (Cell Signaling #9562, dilution 1:200) = ICC
anti-β-Catenin (Cell Signaling #9562, dilution 1:200) = TEM

Animals and other organisms

Policy information about studies involving animals; ARRIVE guidelines recommended for reporting animal research

Laboratory animals	Tg(TNF)197Gkl (hTNFtg), males, up to 14 weeks (http://www.informatics.jax.org/allele/MGI:3053711); B6.129X1-Lasp1tm1Chew/J (Lasp1-/-), males, up to 14 weeks (https://www.jax.org/strain/008789); wild type mice C57/Bl6 (Charles River); Mice were kept under individually ventilated cage (IVC) conditions with alternate 12 hours cycles of dark and light. Animals were allowed free excess to feed and water ad libitum. All experimental procedures were carried out in strict adherence to the rules and guidelines for the ethical use of animals in research and were approved by the State Office for Nature, Environment and Consumer Affairs (Landesamt für Natur, Umwelt und Verbraucherschutz (LANUV) NRW, Germany) under the reference 8.87-51.05.2011.033 and 84-02.04.2014.A519.
Wild animals	No wild animals were used.
Field-collected samples	No field-collected samples were used in this study.
Ethics oversight	The Animal Use Committee accepted all animal experiments under the reference number 8.87-51.05.2011.033 and 84-02.04.2014.A519 .

Note that full information on the approval of the study protocol must also be provided in the manuscript.

Human research participants

Policy information about studies involving human research participants

Population characteristics	Samples of synovial tissues from subjects with rheumatoid arthritis or osteoarthritis (according to the 1987 revised American College of Rheumatology criteria for RA and OA) were used, no further covariate-erlevant population characteristics were analysed.
Recruitment	The synovial tissue of both groups was obtained at joint replacement surgery.
Ethics oversight	The ethics committees of the Medical University of the University Hospital Münster approved all studies with human samples. Samples of synovial tissues from subjects with RA or OA (according to the 1987 revised American College of Rheumatology criteria for RA and OA) were obtained as operational waste at joint replacement surgery. In addition, synovial tissues of patients included in the early arthritis patient cohort in Birmingham (BEACON) were used in this study. All patients were naïve to treatment with disease modifying anti-rheumatic drugs (DMARDs) and corticosteroids at inclusion. The tissue samples were kindly provided by Prof. Dr. Christopher Buckley (Birmingham, UK). All subjects gave informed consent prior to surgery.

Note that full information on the approval of the study protocol must also be provided in the manuscript.

ChIP-seq

Data deposition

☒ Confirm that both raw and final processed data have been deposited in a public database such as GEO.

☒ Confirm that you have deposited or provided access to graph files (e.g. BED files) for the called peaks.

Data access links
May remain private before publication.

All human sequencing data are deposited in Gene Expression Omnibus (https://www.ncbi.nlm.nih.gov/geo/query/acc.cgi?acc=GSE112658) with the primary accession code GSE112658 (GSE112655) as published by Rizi Ai et al. Nat Commun 2018. The murine sequencing data are deposited at the European Genome-Phenome Archive (https://www.ebi.ac.uk) under the accession numbers ERS2369617, ERS2369618, ERS2369619, ERS2369620.

Files in database submission

53_Mf05_LPFi_Ct_H3K4me1_B_1
53_Mf05_LPFi_Ct_H3K4me3_B_1
53_Mf05_LPFi_Ct_H3K9me3_B_1
53_Mf05_LPFi_Ct_H3K27ac_B_1
53_Mf05_LPFi_Ct_H3K27me3_B_1
53_Mf05_LPFi_Ct_H3K36me3_B_1
53_Mf05_LPFi_Ct_Input_B_1

53_Mf06_LPFi_RA_H3K4me1_B_1
53_Mf06_LPFi_RA_H3K4me3_B_1
53_Mf06_LPFi_RA_H3K9me3_B_1
53_Mf06_LPFi_RA_H3K27ac_B_1
53_Mf06_LPFi_RA_H3K27me3_B_1
53_Mf06_LPFi_RA_H3K36me3_B_1
53_Mf06_LPFi_RA_Input_B_1

53_Mf07_LPFi_Ct_H3K4me1_B_1
53_Mf07_LPFi_Ct_H3K4me3_B_1
53_Mf07_LPFi_Ct_H3K9me3_B_1
53_Mf07_LPFi_Ct_H3K27ac_B_1
53_Mf07_LPFi_Ct_H3K27me3_B_1
53_Mf07_LPFi_Ct_H3K36me3_B_1
53_Mf07_LPFi_Ct_Input_B_1

53_Mf08_LPFi_RA_H3K4me1_B_1
53_Mf08_LPFi_RA_H3K4me3_B_1
53_Mf08_LPFi_RA_H3K9me3_B_1
53_Mf08_LPFi_RA_H3K27ac_B_1
53_Mf08_LPFi_RA_H3K27me3_B_1
53_Mf08_LPFi_RA_H3K36me3_B_1
53_Mf08_LPFi_RA_Input_B_1

Genome browser session
(e.g. UCSC)

https://genome.ucsc.edu

Methodology

Replicates

2 biological replicates for each condition each ChIP. Agreement: Person correlation for each replicates

	Ct	RA
H3K4me1	0.94	0.92
H3K4me3	0.97	0.97
H3K9me3	0.96	0.96
H3K27ac	0.69	0.61
H3K27me3	0.92	0.94
H3K36me3	0.97	0.96
Input	0.90	0.95

Sequencing depth

All ChIP-seq reads are Paired-end-50 read counts (pairs):

53_Mf05_LPFi_Ct_H3K4me1_B_1: 47,478,612
53_Mf05_LPFi_Ct_H3K4me3_B_1: 30,067,683
53_Mf05_LPFi_Ct_H3K9me3_B_1: 45,618,028
53_Mf05_LPFi_Ct_H3K27ac_B_1: 30,737,681
53_Mf05_LPFi_Ct_H3K27me3_B_1: 41,524,987
53_Mf05_LPFi_Ct_H3K36me3_B_1: 51,876,143

53_Mf05_LPFi_Ct_Input_B_1: 110,210,573

53_Mf06_LPFi_RA_H3K4me1_B_1: 45,638,851
53_Mf06_LPFi_RA_H3K4me3_B_1: 32,905,654
53_Mf06_LPFi_RA_H3K9me3_B_1: 38,523,904
53_Mf06_LPFi_RA_H3K27ac_B_1: 23,443,351
53_Mf06_LPFi_RA_H3K27me3_B_1: 47,137,556
53_Mf06_LPFi_RA_H3K36me3_B_1: 41,050,302
53_Mf06_LPFi_RA_Input_B_1: 116,876,193

53_Mf07_LPFi_Ct_H3K4me1_B_1: 51,654,286
53_Mf07_LPFi_Ct_H3K4me3_B_1: 47,243,190
53_Mf07_LPFi_Ct_H3K9me3_B_1: 90,085,392
53_Mf07_LPFi_Ct_H3K27ac_B_1: 39,512,183
53_Mf07_LPFi_Ct_H3K27me3_B_1: 49,598,358
53_Mf07_LPFi_Ct_H3K36me3_B_1: 58,750,908
53_Mf07_LPFi_Ct_Input_B_1: 124,015,168

53_Mf08_LPFi_RA_H3K4me1_B_1: 65,446,710
53_Mf08_LPFi_RA_H3K4me3_B_1: 41,294,146
53_Mf08_LPFi_RA_H3K9me3_B_1: 48,013,923
53_Mf08_LPFi_RA_H3K27ac_B_1: 45,478,326
53_Mf08_LPFi_RA_H3K27me3_B_1: 56,337,965
53_Mf08_LPFi_RA_H3K36me3_B_1: 51,639,830
53_Mf08_LPFi_RA_Input_B_1: 114,623,689

Antibodies

Input no Ab
H3K4me1 Diagenode pAb-194-050 A1863-001D
H3K4me3 Diagenode pAb-003-050 A5051-001P
H3K9me3 Diagenode pAb-193-050 A1671-001P
H3K27ac Diagenode pAb-196-050 A1723-0040
H3K27me3 Diagenode pAb-195-050 A1811-001P
H3K36me3 Diagenode pAb-192-050 A1847-001P

Peak calling parameters

DOI: 10.17617/1.2W

Data quality

sample// EpiRR ID//
antibody// ENA IDs// #read pairs// mapping efficiency// %duplicates// #peaks (FDR<0.05)// FrIP// #peaks (FDR<=0.05 & FC>=5)

53_Mf05_LPFi_Ct IHECRE00004584.1
H3K27ac ERX2579978, ERX2579979 61475362 98,0% 60,1% 104492 0,34 57512
H3K27me3 ERX2580037, ERX2580038 83049974 98,9% 14,3% 126781 0,28 1033
H3K36me3 ERX2580093, ERX2580094 103752286 99,2% 27,6% 55754 0,78 10760
H3K4me1 ERX2580148, ERX2580149 94957224 99,4% 18,0% 178604 0,69 52885
H3K4me3 ERX2580205, ERX2580206 60135366 99,0% 41,6% 41275 0,77 30876
H3K9me3 ERX2580262, ERX2580263 91236056 97,4% 28,2% 111567 0,4 2471
Input ERX2579784, ERX2579785 220421146 96,1% 28,6%

53_Mf06_LPFi_RA IHECRE00004585.1
H3K27ac ERX2579976, ERX2579977 46886702 98,4% 88,8% 56407 0,32 37000
H3K27me3 ERX2580035, ERX2580036 94275112 98,9% 19,7% 157476 0,38 2204
H3K36me3 ERX2580091, ERX2580092 82100604 99,3% 14,9% 51236 0,78 10473
H3K4me1 ERX2580146, ERX2580147 91277702 99,3% 18,8% 167368 0,69 39969
H3K4me3 ERX2580203, ERX2580204 65811308 98,8% 28,8% 43194 0,74 30890
H3K9me3 ERX2580260, ERX2580261 77047808 96,8% 37,8% 91343 0,45 3074
Input ERX2579782, ERX2579783 233752386 97,1% 23,3%

53_Mf07_LPFi_Ct IHECRE00004586.1
H3K27ac ERX2579974, ERX2579975 79024366 96,1% 18,3% 30805 0,13 19209
H3K27me3 ERX2580033, ERX2580034 99196716 97,4% 9,1% 204150 0,4 3580
H3K36me3 ERX2580089, ERX2580090 117501816 97,7% 9,0% 63795 0,67 11829
H3K4me1 ERX2580144, ERX2580145 103308572 98,2% 11,4% 168714 0,6 53214
H3K4me3 ERX2580201, ERX2580202 94486380 97,4% 13,3% 34148 0,43 23943
H3K9me3 ERX2580258, ERX2580259 180170784 96,3% 26,6% 191439 0,51 6134
Input ERX2579780, ERX2579781 248030336 98,1% 14,6%

53_Mf08_LPFi_RA IHECRE00004587.1

H3K27ac	ERX2579972, ERX2579973	90956652	89,3%	55,2%	23459	0,11	17023
H3K27me3	ERX2580031, ERX2580032	112675930	96,7%	21,5%	168426	0,35	2201
H3K36me3	ERX2580087, ERX2580088	103279660	97,4%	24,2%	81880	0,67	8444
H3K4me1	ERX2580142, ERX2580143	130893420	98,1%	26,9%	168645	0,5	29954
H3K4me3	ERX2580199, ERX2580200	82588292	96,2%	34,8%	35551	0,46	27656
H3K9me3	ERX2580256, ERX2580257	96027846	95,3%	34,5%	124673	0,39	4063

Software: Bioconductor package normr 1.16.0

Flow Cytometry

Plots

Confirm that:

- [x] The axis labels state the marker and fluorochrome used (e.g. CD4-FITC).
- [x] The axis scales are clearly visible. Include numbers along axes only for bottom left plot of group (a 'group' is an analysis of identical markers).
- [x] All plots are contour plots with outliers or pseudocolor plots.
- [x] A numerical value for number of cells or percentage (with statistics) is provided.

Methodology

Sample preparation

As described in the methods, murine FLS and inflammatory cells were isolated from hind paws of different genotypes. Paws were digested using collagenase type IV (Worthington Biochemicals) in DMEM for 1.5 hours, centrifuged at 405 xg for 5 minutes and resuspended with DMEM Aand stained with anti-mouse CD11b APC (BioLegend clone M1/70, dilution 1:100) and CD14 AF647 (BioLegend #123327, dilution 1:100) antibodies. For the FLS subtype detection studies, all legs from hTNFtg and hTNFtg/Lasp1-/- mice were dissected and tibia and femur with intact joints were digested for 1 hour at 37°C in DMEM containing 1 mg/ml Collagenase D (Worthington) and 10 mg/ml of DNase I (Sigma-Aldrich). Cells were centrifuged and the pellet resuspended in Red Bood Lysis Buffer (Sigma-Adrich) and washed with MACS buffer (20 mM EDTA, 5% BSA, PBS). In addition, cells were excluded using Zombie Violet staining (BioLegend). Cells were stained at 4°C in MACS buffer using the following antibodies: anti-CD45 PerCP (BioLegend #30-F11, dilution 1:100), anti-CD31 Alexa488 (eBioscience clone 390, dilution 1:100), anti-THY1.2 APC (Invitrogen #17-0902-81, dilution 1:50), anti-Podoplanin PE (Invitrogen clone 8.1.1, dilution 1:100).

Instrument

Macrophage detection: Canto II (BD Biosciences)
FLS subtype detection: Flow Cytometer Cytoflex S, Beckman Coulter, Germany

Software

Macrophage detection: Canto Clinical software II
FLS subtype detection: FlowJo Version 10.6.1

Cell population abundance

N/A

Gating strategy

1.) SSC-A vs. FSC-A gating to exclude debris. 2.) FSC-H vs. FSC-A gating to exclude doublets. 3.) SSC-A vs. Zombie gating to exclude dead cells. 4.) CD31 vs. CD45 gating to quantify FLS. 5) Identification of FLS subpopulations by Podoplanin and CD90 staining.

- [x] Tick this box to confirm that a figure exemplifying the gating strategy is provided in the Supplementary Information.
