# Supplementary material for: Lasp1 regulates adherens junction dynamics and fibroblast transformation in destructive arthritis
Source: Nat Commun. 2021 Jun 15;12:3624. doi: 10.1038/s41467-021-23706-8 (PMC8206096; doi:10.1038/s41467-021-23706-8)
Supplement: Supplementary file 10 — Source Data [file 41467_2021_23706_MOESM10_ESM.pdf]

Figure 1d   qPCR analysis: normalised Lasp1 expression in FLS

wt FLS	hTNFtg FLS
1,000	1,142
0,955	1,188
0,966	1,221
1,042	1,203
0,858	1,291

Figure 1e **Bsmooth validation wt and hTNFtg FLS (methylation rates %)**

wt FLS	hTNFtg FLS
10,6	58
9,9	40,3

Methylene validation wt and hTNFtg FLS (methylation rates %)

wt FLS	hTNFtg FLS
8,3	41,5
7,6	34,2

Figure 1f **Bsmooth validation wt and G6PI FLS (methylation rates %)**

wt FLS	G6PI FLS
10,6	48,3
12,5	36,1
14,6	56,6

Methylene validation wt and G6PI FLS (methylation rates %)

wt FLS	G6PI FLS
7,5	41,9
12,6	23,6
11,6	47,2

Figure 2a **IHC evaluation OA vs. RA (rel. intensity)**

OA	RA
0,143	0,201
0,127	0,185
0,137	0,193
0,021	0,193
0,045	0,207
0,045	0,217
0,068	0,173
0,079	0,173
0,075	0,146

IHC evaluation wt vs. hTNFtg (rel. intensity)

wt	*hTNFtg*
0,06	0,137
0,029	0,127
0,025	0,117
0,025	0,14
0,033	0,137
0,053	0,146
0,004	0,076
0,004	0,097
0,008	0,097

IHC evaluation wt vs G6PI (rel. intensity)

wt	G6PI
0,13	0,207
0,093	0,267
0,097	0,307
0,086	0,31
0,089	0,29
0,089	0,25

Figure 2c **WB OA-FLS vs. RA-FLS (rel. Intensity Lasp1 vs GAPDH)**

OA-FLS	RA-FLS
1	1,709
1	1,944
1	2,507
1	2,599
1	3,184
1	3,18

WB wt vs. hTNFtg FLS (rel. Intensity Lasp1 vs GAPDH)

wt FLS	hTNFtg FLS
1	4,596
1	3,703
1	3,676

WB wt vs G6PI FLS (rel. Intensity Lasp1 vs GAPDH)

wt FLS	G6PI FLS
1	1,734
1	2,136

Immunoblotting for Lasp1 expression

OA-FLS vs. RA-FLS

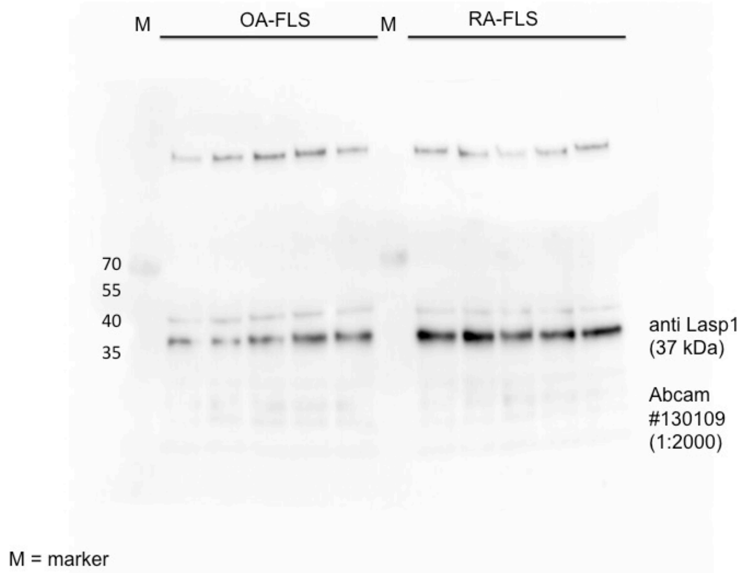

23.07.19

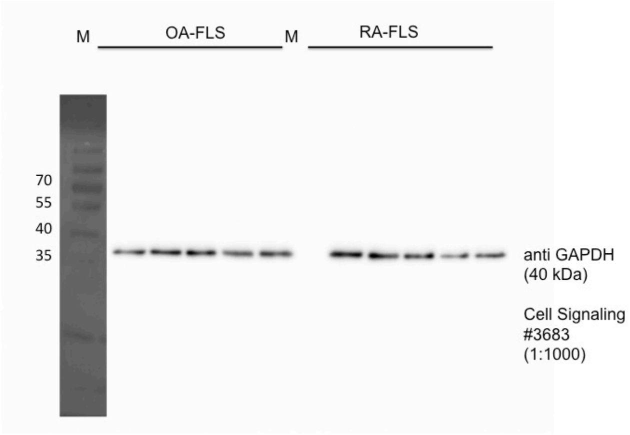

M = marker

25.07.19

wt vs. hTNFtg FLS

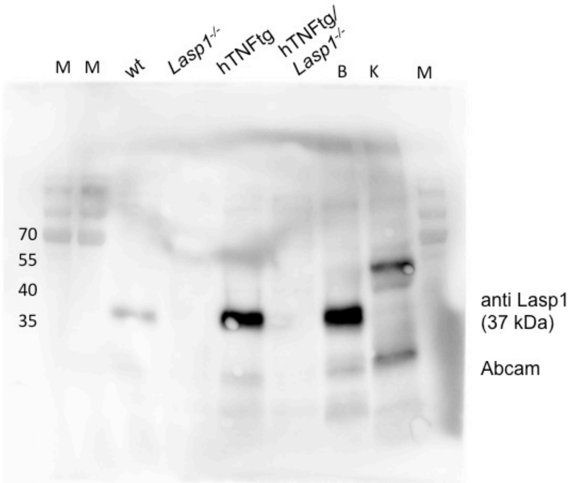

M = marker
B= brain (control)
K = kidney (control)

04.04.13

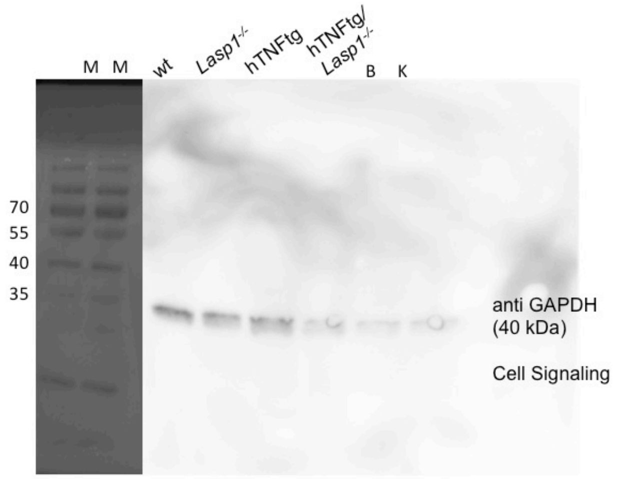

M = marker
B= brain (control)
K = kidney (control)
13.04.13

wt vs. G6PI FLS

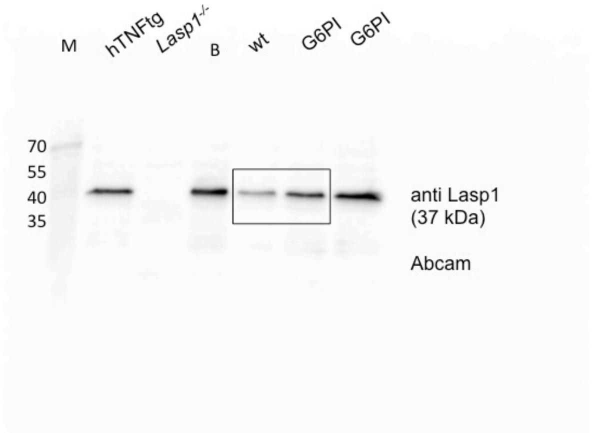

M = marker
B= brain (control)
K = kidney (control)
23.11.15

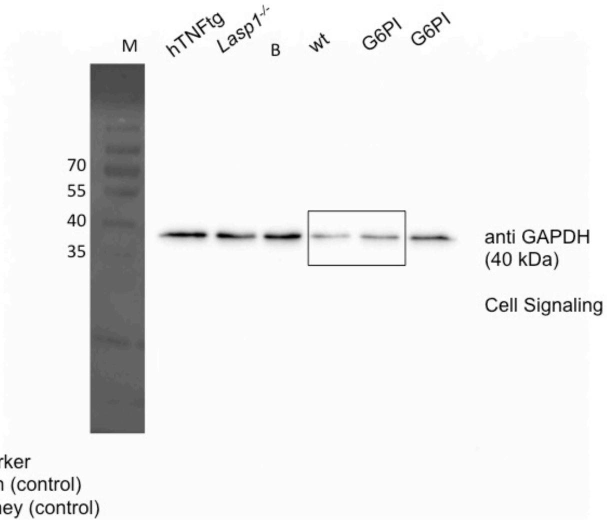

M = marker
B= brain (control)
K = kidney (control)

24.11.15

Figure 2e **Cellular distribution in synovial organ sections**

wt		*Lasp1-/-*	
Mean	SD	Mean	SD
127,22881	18	128,39162	30
127,468334	18	128,995907	29
128,053874	19	130,239871	29
127,714522	20	131,842459	29
126,969164	19	132,702311	30
126,911704	17	132,26653	29
127,094245	16	133,163087	29
127,166231	15	135,183036	31
126,954909	14	136,204907	31
126,30537	14	137,388756	33
125,464863	15	138,862639	34
124,821036	15	140,24604	36
124,826778	15	140,979237	36
125,413475	14	139,696627	35
125,661501	15	139,085433	36
125,662926	15	138,187997	36
124,902421	14	139,229602	37
124,346255	14	141,95106	37
124,504555	14	143,907775	37
124,241911	15	142,657362	36
124,732966	14	139,864206	32
125,285822	14	137,849494	30
125,023997	15	136,538751	29
125,139857	16	136,271807	30
124,901652	16	137,265847	32
125,327611	16	138,809191	34
126,03621	16	140,814141	37
126,771691	16	142,734914	39
126,89343	18	144,387031	40
126,606851	18	144,11846	39
126,745021	17	143,063839	38
126,859488	17	140,934883	36
127,862314	18	139,808939	34
128,641966	19	137,329427	32
128,640845	18	134,96567	31
128,169211	18	132,883587	31
128,352423	16	132,354211	31
128,513025	16	132,114183	30
128,126484	17	130,715415	29
128,072548	19	132,040838	29
128,615592	20	134,763613	31
129,093018	21	136,4765	34

128,987352	22	136,826016	37
128,930919	23	137,069418	37
128,862631	21	137,692531	37
129,165389	22	137,655144	38
128,477075	20	138,056108	39
127,854761	17	138,213712	39
126,866718	16	138,190155	39
127,17453	17	137,499636	37
128,375103	18	135,922844	34
128,710383	19	135,58854	34
128,411767	17	135,74253	34
128,502614	17	136,840555	35
128,466534	17	137,23345	35
128,007522	16	136,681118	36
128,317469	16	136,84045	37
128,726461	18	136,897652	37
129,228411	18	137,017393	37
129,647621	18	136,270984	37
129,251892	18	135,187033	36
128,911958	18	135,390123	35
128,797564	21	135,982335	35
128,457569	21	136,370067	35
128,183786	21	137,420926	34
128,215895	20	138,54361	35
128,334227	20	139,488107	38
129,373571	20	140,054804	39
130,067444	22	138,551059	38
130,155144	23	137,715164	36
130,458672	22	137,36213	37
130,753793	20	136,274623	36
130,881833	19	136,864465	36
131,264208	20	135,964789	34
131,527677	21	135,493804	33
131,926369	21	134,311444	32
131,567819	20	133,129798	31
131,220095	18	132,243672	31
131,455866	20	132,153342	33
130,941424	20	131,72209	35
130,636298	20	131,731324	37
131,010804	20	132,289124	37
131,082936	21	132,801476	39
131,516293	23	134,125701	41
132,821273	25	135,189923	44
133,617027	26	136,079377	45
133,596778	26	138,350572	45
133,834061	26	139,695795	46
134,22288	25	139,275603	44
133,760777	25	137,377998	44
133,664691	25	135,400072	45

134,191649	25	133,558775	44
134,745162	25	132,360577	43
134,957152	26	131,517321	43
135,163429	26	132,832519	44
135,321626	24	134,12158	43
134,936915	24	135,413196	43
134,76468	24	136,682029	43
134,653357	23	136,925928	42
134,71254	24	137,407611	42
135,05608	24	136,81142	41
135,804917	25	138,552161	42
136,295281	26	140,030764	42
135,871213	26	141,418459	42
135,429531	26	141,933986	43
136,842788	27	141,728707	45
139,025525	29	141,159053	45
140,704345	32	140,298557	44
140,911362	31	139,603362	44
140,963519	30	139,106527	46
140,234272	28	137,49207	46
140,312466	27	136,651901	46
141,419759	28	136,824774	47
142,061017	29	136,269238	47
141,333774	29	134,386769	46
140,881345	28	133,275409	45
141,074471	29	130,618494	42
140,8496	30	128,526486	41
141,080239	29	127,50874	41
141,520256	28	127,381452	42
141,681204	28	127,467457	42
141,757308	28	127,367398	41
141,390096	28	127,34351	40
141,319538	28	126,919329	39
142,381598	28	126,131112	38
143,962025	29	125,802774	37
144,98667	30	126,408007	35
145,597525	30	127,351936	34
147,370136	30	130,282809	35
148,637313	30	132,437948	37
148,656611	29	134,09238	39
148,722682	28	135,134466	39
149,586969	29	136,048665	41
150,688405	30	136,946606	42
150,543243	29	137,822815	43
150,205035	28	138,630495	44
151,761585	29	139,248934	46
153,220413	30	137,835352	46
155,203724	32	135,924565	46
156,909824	33	136,100972	47

158,244086	34	136,243462	47
158,683549	34	136,191095	45
157,870996	33	136,813128	44
157,996355	32	137,623266	43
158,696574	31	138,612315	44
160,113415	30	138,833669	44
162,982999	31	138,573948	43
167,567108	33	137,554858	43
173,390025	35	136,391977	43
179,911172	37	136,656521	45
187,278965	37	137,492208	46
193,846265	36	136,435771	45
200,98294	35	137,126117	45
207,853513	34	138,916903	44
211,594836	34	141,693102	49
209,189433	41	144,167038	52
196,899463	54	144,053894	53
176,015252	65	142,816626	56
147,717711	68	137,74164	59
112,912285	61	129,990432	63
81,9748898	45	120,865965	65
63,0041687	32	107,887049	67
54,5489283	27	88,5050137	62
		72,1247879	57

hTNFtg		hTNFtg/*Lasp1-/-*	
Mean	SD	Mean	SD
131,78821	22	123,506416	43
132,19356	23	125,569924	41
133,23587	25	129,953843	39
134,632515	27	136,004266	38
134,751591	27	142,914862	37
134,429572	25	147,904618	38
133,882662	24	150,635939	39
133,386237	24	150,038877	38
133,384396	25	148,660774	36
133,153407	25	147,57101	35
133,888256	26	146,236085	33
134,490103	25	145,212724	32
135,174174	25	144,384264	33
134,397952	24	144,639158	33
133,825278	24	144,226448	32
134,173848	22	143,440542	31
134,995598	21	143,437617	32
135,766253	22	143,814711	33
136,370548	24	143,728624	34
136,916547	24	143,736129	33
136,758696	24	144,009296	32
136,170924	25	144,515747	33
136,901512	26	144,453037	33
137,179785	27	144,211337	34
136,635282	26	144,273731	35
136,197843	25	144,314454	35
136,706131	26	143,95166	35
137,463956	28	143,834364	35
137,54978	29	143,638553	34
137,577574	29	143,418777	33
136,734774	27	143,020544	34
136,126329	26	142,49525	35
135,791413	26	142,653214	36
135,779231	27	142,168835	37
136,060042	28	141,409514	36
137,077548	30	140,883206	35
138,056106	31	140,725537	36
138,863139	32	140,321923	35

139,617678	31	139,933286	34
139,269575	31	139,355389	33
139,702295	32	138,458815	32
139,682568	32	138,325131	31
139,48885	32	138,111747	31
139,123402	32	138,926479	33
138,925515	32	139,175275	34
138,056985	31	138,623622	33
137,195638	32	138,746277	33
136,786308	32	139,135721	33
136,654155	32	139,550334	33
136,563904	31	139,835077	34
136,657429	30	139,692978	33
136,359937	30	139,564113	32
135,982282	30	139,680592	32
136,311962	30	139,65996	31
137,256518	32	139,797368	33
138,238681	33	140,064266	34
139,552925	35	141,135359	34
139,922727	37	141,083519	34
140,3319	36	140,382991	34
141,213572	36	139,605041	33
141,246485	37	139,013355	32
141,526849	38	138,45238	32
141,776128	37	137,32704	33
141,182466	36	136,697622	32
140,449359	35	136,226889	30
139,845945	35	135,869085	30
140,703796	36	136,055365	30
141,382747	36	136,886974	30
141,020014	37	137,319439	32
140,59204	37	137,500074	33
141,240873	37	137,808517	33
141,588377	36	138,30961	33
141,254348	37	139,249695	34
140,760512	38	139,922337	34
140,298397	36	140,651451	35
139,744394	35	141,506607	36
139,630551	34	141,368466	36
138,806826	34	140,773216	35
138,46721	35	140,700095	35
138,780008	35	141,077939	36
138,721544	35	140,529775	36
137,881059	34	139,652651	35
137,343273	33	139,124602	35
137,20489	32	138,717098	35
136,996438	31	137,675653	34
136,654808	31	136,462408	34
136,44142	31	135,991248	34

136,642255	33	135,735674	33
136,971751	34	135,20686	32
136,165589	34	134,884419	33
134,778412	34	135,006232	32
134,077939	33	134,552115	31
134,005426	32	134,088157	29
133,877818	30	134,351679	31
134,127569	30	134,39042	32
134,128467	31	133,941234	32
132,936647	31	133,598724	32
132,692212	31	132,999927	31
132,629206	32	131,928473	30
131,978172	32	131,288257	30
131,477011	31	131,023609	29
130,774128	31	131,302927	30
129,818356	31	131,506992	31
129,886859	32	131,627257	31
130,252302	34	132,086599	32
130,637721	34	131,935503	33
131,014728	34	131,711291	34
131,592383	35	132,192551	33
131,533124	35	132,880762	32
131,354622	36	132,919684	33
130,76595	36	132,095459	33
130,038424	37	131,006878	32
129,936196	37	130,768602	32
130,923389	38	130,675107	33
131,480414	39	129,91988	33
131,23722	40	129,830815	33
130,211046	40	129,14527	33
129,205608	39	128,516401	33
128,792563	40	128,688534	33
128,007624	40	128,947748	35
127,597288	41	129,250855	36
127,423985	40	129,91755	36
127,035379	40	130,69952	36
126,30384	40	131,383964	36
126,251915	41	131,493974	36
126,606863	41	131,169115	36
126,727641	42	130,90495	36
127,027774	42	131,287822	37
126,853734	41	131,336241	38
126,122874	41	131,146464	38
124,895199	41	131,619707	38
123,72462	42	131,092298	37
122,963855	41	131,429013	37
123,041054	41	131,789648	36
122,76237	41	131,559765	35
123,327915	43	131,847966	34

124,10572	43	131,882572	35
124,185333	44	131,915915	36
123,577399	45	132,354051	37
123,240964	46	133,369749	37
122,576367	47	135,044067	37
122,170586	46	137,042571	38
121,57283	44	137,444706	39
121,240803	44	137,656944	39
119,978917	44	137,721565	38
118,732528	44	138,49559	38
117,371196	44	139,323399	38
116,577834	45	140,611474	39
116,58259	47	141,109146	40
117,224486	48	141,831066	41
116,396528	48	142,072484	42
115,40339	49	142,565609	42
114,193567	49	144,184184	42
111,954759	50	147,653502	43
109,032498	52	152,482096	44
103,584054	52	155,600092	47
97,1639898	52	157,639495	50
90,150808	53	155,015872	54
81,7729743	52	141,885758	58
72,0523482	49	119,954124	58
61,6674035	42	95,616591	54
52,928542	35	76,7903627	48
47,1383535	31	65,4305722	44
44,3848456	29	59,59499	41

Figure 3b **% closed area after 24hrs**

wt FLS	*Lasp1-/- FLS*	hTNFtg FLS	hTNFtg/*Lasp1-/-* FLS
39,60028	18,34468	59,70198	28,599
24,86335	17,94582	100	27,22754
50,33853	22,53682	100	24,43078
55,67094	41,10585	100	
51,4593		100	

Figure 3c **Proliferation (OD)**

wt FLS	*Lasp1-/- FLS*	hTNFtg FLS	hTNFtg/*Lasp1-/-* FLS
36360	20505	44572	37234
30928	30797	39913	41106
33417	21219	42264	47699
39199	26256	36324	37846
27307	32812	40973	35771
25956	31944	48182	46506
31223	34590	44721	43685
31177	21791	40836	33168
23089	29537	39396	38895

Figure 3d **Migration analysis (ascending slope - grey values)**

time (min)	hTNFtg FLS	hTNFtg/*Lasp1-/-* FLS
0	31,96903	31,08269
2	32,97198	31,61495
4	32,53318	32,00608
6	32,84661	32,46516
8	31,59292	32,78338
10	32,65855	33,09489
12	31,15413	33,42146
14	33,6615	33,75826
16	33,78687	34,03994
18	33,41077	34,12946
20	32,72124	34,38263
22	32,90929	34,77015
24	34,4764	34,98684
26	32,40782	35,2084
28	33,16003	35,31159
30	35,10324	35,58431
32	35,41667	35,89353
34	34,78982	36,07313
36	35,54203	36,26292
38	36,67035	36,22124
40	36,29425	36,52177
42	36,60767	36,79875
44	38,04941	37,07803
46	38,61357	36,69382
48	39,11504	36,47199
50	40,05531	36,62034
52	41,37168	36,92312
54	43,81637	37,05037
56	40,61947	37,03571
58	41,81047	37,02244
60	42,06121	37,72732
62	41,49705	38,33565
64	40,68215	38,48147
66	41,309	38,70066
68	41,49705	38,81436
70	43,50295	39,25652
72	42,81342	39,56771
74	43,00148	39,74601
76	43,50295	39,98384
78	43,44027	40,10809
80	44,69395	40,26221
82	45,00737	40,40352
84	44,88201	40,37729
86	45,44617	40,37425
88	46,01033	40,33905
90	48,26696	40,05037
92	48,76844	39,99419
94	48,20428	40,03896

96	46,69985	40,24561
98	46,88791	40,15878
100	46,19838	40,33636
102	47,07596	40,66636
104	48,39233	41,00747
106	50,02213	41,22874
108	50,21018	41,51316
110	51,27581	41,66281
112	51,3385	41,87749
114	50,96239	42,05251
116	52,27876	42,08237
118	50,64897	42,20287
120	50,96239	42,2675
122	50,77434	42,01122
124	52,65487	42,01146
126	52,15339	42,33478
128	52,21608	42,05642
130	52,84292	42,18916
132	53,21902	42,13808
134	55,22493	42,26118
136	52,96829	42,54057
138	54,22198	42,52888
140	52,21608	42,44129
142	52,59218	42,7048
144	53,7205	42,78508
146	53,65782	42,8987
148	54,41003	42,79966
150	54,84882	42,9552
152	54,28466	42,93347
154	54,66077	43,03899
156	57,85767	43,17612
158	59,80088	43,31456
160	58,10841	43,72879
162	59,42478	44,08379
164	58,04572	44,30389
166	59,92625	44,81459
168	60,9292	45,14432
170	60,5531	45,37405
172	59,04867	45,57886
174	60,17699	45,63112
176	61,7441	45,8479
178	63,56195	45,94722
180	63,06047	45,99123
182	63,56195	46,49214
184	64,00074	46,90214
186	63,75	47,36591
188	62,37094	47,70338
190	62,74705	48,26592
192	62,99779	48,52872
194	64,31416	49,09016
196	64,25147	49,22752
198	63,56195	49,61216

200	65,69321	49,83964
202	66,32006	50,20263
204	65,88127	50,22701
206	65,50517	50,61722
208	65,06637	50,67802
210	68,38864	50,72326
212	68,26328	50,79686
214	70,26917	51,01679
216	68,26328	51,25944
218	67,19764	51,42877
220	69,45428	51,4311
222	69,57964	51,44485
224	70,77065	51,74878
226	71,08407	51,69287
228	72,27507	51,41443
230	69,26623	51,11631
232	71,02139	51,10051
234	73,90487	51,05673
236	71,14675	51,06704
238	72,27507	50,78437
240	71,20944	50,76454
242	71,58555	50,84098
244	75,09587	50,90949
246	74,34366	50,98689
248	76,34956	51,11256
250	76,09882	51,36509
252	77,03909	51,53196
254	75,97345	51,59521
256	77,35251	51,85572
258	77,72861	52,13223
260	76,9764	52,14783
262	78,23009	52,44872
264	77,85398	52,59399
266	78,79425	52,77698
268	76,66298	52,89554
270	77,41519	53,05507
272	77,7913	53,1427
274	77,85398	53,42328
276	78,1674	53,61414
278	78,1674	53,75348
280	78,04204	53,83225
282	79,10767	54,20239
284	78,91962	54,70721
286	79,29572	55,3496
288	79,29572	55,70539
290	78,04204	56,32905
292	78,91962	56,79417
294	79,42109	57,40234
296	80,80015	57,98775
298	80,67478	58,21666
300	80,9882	58,47219
302	80,29868	59,03891

304	80,86283	59,01521
306	80,04794	59,27382
308	78,91962	59,45935
310	81,17625	59,55479
312	81,30162	59,76896
314	81,8031	59,9716
316	82,05383	60,01561
318	79,60915	60,52821
320	82,11652	60,84604
322	80,1733	61,10841
324	82,49262	61,29105
326	83,24483	61,47377
328	82,42994	61,63037
330	81,67773	61,61058
332	81,61504	61,48151
334	81,3643	61,16044
336	83,809	61,43718
338	83,55826	61,5258
340	83,74632	61,87527
342	83,43289	62,07783
344	84,12242	62,55875
346	84,05974	62,9373
348	83,99705	63,42845
350	85,06268	63,24708
352	84,87463	63,36007
354	84,62389	63,41107
356	85,31342	63,29654
358	84,31047	63,3208
360	85,68953	62,92533
362	83,55826	62,88021
364	83,30753	63,29148
366	83,87168	63,50758
368	84,31047	63,39309
370	85,43879	63,43051
372	86,06564	63,40933
374	85,43879	64,05432
376	85,56416	64,29875
378	86,81785	64,48685
380	86,69247	64,85679
382	87,19395	65,05061
384	86,94321	65,41822
386	87,82079	65,70927
388	86,62979	65,99146
390	87,88348	66,47831
392	87,88348	66,71559
394	87,382	67,00597
396	88,1969	67,47428
398	88,38496	67,88053
400	87,94617	68,29666
402	88,69838	68,39096
404	89,07449	68,49313
406	87,88348	68,66747

408	89,8267	68,77757
410	89,51328	68,83233
412	89,19985	68,84821
414	88,82375	68,95943
416	89,07449	69,0753
418	90,26549	69,23502
420	92,08334	69,30527
422	92,33407	69,25233
424	91,64455	69,33479
426	91,8326	69,46697
428	91,58186	69,67549
430	92,02065	69,92339
432	92,39675	70,21915
434	92,33407	70,56313
436	93,0236	70,93521
438	94,84145	71,28074
440	91,76991	71,71294
442	92,77286	71,89776
444	92,58481	72,10928
446	94,27728	72,3204
448	94,59071	72,50012
450	94,65339	72,87034
452	95,4056	73,11604
454	95,71902	73,30882
456	93,90118	73,34754
458	94,96681	73,49084
460	94,15192	73,62634
462	95,34292	73,9057
464	95,65634	74,11508
466	95,71902	74,36789
468	94,33997	74,60458
470	96,53392	75,02019
472	96,28319	75,35864
474	96,91003	75,48732
476	96,84734	75,53998
478	96,97272	75,63638
480	97,53687	75,42738
482	97,8503	75,33549
484	97,47419	75,47199
486	96,84734	75,49245
488	98,41445	75,7069
490	97,0354	76,02481
492	97,34882	76,15309
494	98,97861	76,75249
496	99,79351	77,09545
498	98,41445	77,28718
500	102,3009	77,35165
502	100,295	77,47136
504	97,97566	77,46614
506	99,48009	77,58415
508	100,9845	77,7822
510	99,29204	78,19283

512	99,22935	78,51652
514	99,79351	79,09821
516	99,22935	79,55476
518	99,73083	79,93742
520	100,9845	80,31258
522	100,9218	80,41929
524	99,4174	80,40483
526	102,0501	80,64353
528	100,7965	80,76975
530	101,2979	80,80804
532	102,1128	80,9616
534	101,7367	81,06535
536	100,7338	81,37492
538	102,4889	81,59161
540	102,4889	81,56977
542	104,3695	81,79358
544	104,4322	82,1039
546	106,25	82,51888
548	103,4919	82,619
550	104,6829	82,98811
552	105,3724	83,18166
554	103,2411	83,44987
556	104,5575	83,39787
558	104,8709	83,46164
560	106,3127	83,31894
562	107,0022	83,5659
564	106,5007	83,47649
566	106,7515	83,74526
568	106,1246	83,8364
570	107,3783	84,12547
572	107,1903	84,44512
574	107,6291	84,71752
576	107,0022	84,67651
578	107,1903	84,74503
580	107,5664	84,6937
582	107,1903	84,85137
584	109,3842	84,78334
586	107,441	84,65368
588	108,9454	84,49001
590	107,6917	84,63681
592	109,3215	84,82186
594	108,6947	84,97637
596	109,5723	85,09308
598	108,8201	85,33901
600	109,6976	85,6069
602	110,5125	86,09272
604	111,4528	86,52307
606	110,5752	86,63918
608	110,7006	86,79978
610	110,1991	87,0222
612	110,1364	87,16969
614	111,4528	87,21247

616	111,8289	87,1894
618	111,8916	87,1534
620	113,7721	87,16846
622	112,3304	87,15155
624	112,205	87,02666
626	112,3931	86,89056
628	112,205	86,94702
630	113,5214	86,92107
632	112,4557	86,92406
634	112,8319	87,07941
636	112,8945	87,39689
638	112,7692	87,52844
640	112,7692	87,75838
642	113,1453	87,69892
644	112,4557	87,68434
646	112,8319	87,65012
648	112,8319	87,32459
650	113,1453	86,91652
652	113,8348	86,85967
654	112,3304	86,78599
656	112,9572	86,93975
658	115,0258	87,14001
660	114,399	87,41494
662	116,0914	87,86437
664	115,3392	88,22934
666	114,7124	88,48759
668	114,8378	88,76932
670	115,8407	88,99585
672	116,969	89,1743
674	116,781	89,22511
676	117,5959	89,17822
678	117,1571	89,28488
680	118,2227	89,32463
682	117,972	89,40297
684	119,5391	89,30136
686	118,4734	89,18011
688	119,2883	89,21085
690	118,4108	89,43801
692	118,2854	89,54449
694	119,1003	89,58474
696	120,6047	89,71828
698	121,3569	89,92217
700	120,6674	90,2564
702	120,6674	90,44216
704	120,1032	90,56827
706	121,3569	90,71567
708	120,8555	90,88496
710	121,6704	91,02955
712	121,4823	91,04286
714	122,4226	91,11027
716	120,7928	91,14803
718	121,7957	91,3285

720	122,4853	91,53959
722	122,6106	91,78595
724	121,9838	91,86939
726	122,6106	92,23815
728	123,4255	92,28753
730	122,4853	92,44311
732	122,6733	92,46519
734	122,736	92,32127
736	123,927	92,39622
738	124,5538	92,33099
740	123,927	92,1382
742	124,4911	92,11161
744	124,3658	91,95188
746	124,5538	91,93912
748	124,5538	91,89021
750	124,8673	91,83214
752	125,2434	91,86354
754	124,2404	91,90392
756	125,1807	92,0621
758	126,1209	92,18813
760	124,3031	92,19939
762	124,7419	92,38369
764	125,8702	92,44082
766	126,0583	92,41632
768	126,1209	92,20879
770	126,1209	92,20907
772	125,4941	92,40656
774	126,5597	92,48665
776	126,497	92,43521
778	127,9388	92,43967
780	126,0583	92,71298
782	127,5627	93,04721
784	127,4373	93,07767
786	127,0612	93,08739
788	128,8791	93,12128
790	128,4403	93,2031
792	128,3776	93,09411
794	127,8134	93,10169
796	128,8164	93,14925
798	127,4373	93,07834
800	128,6283	93,05989
802	129,7566	93,03765
804	129,1925	93,30878
806	128,691	93,61726
808	130,4462	93,60461
810	130,3208	93,69378
812	129,882	93,70449
814	130,4462	93,61698
816	130,8223	93,59221
818	131,5118	93,34901
820	131,7625	93,08846
822	131,5745	93,03465

824	131,8879	92,74589
826	132,8282	92,83996
828	133,2043	92,8878
830	132,264	93,01849
832	132,7028	93,12279
834	133,6431	93,21835
836	132,9535	93,3505
838	133,5804	93,6263
840	134,0192	93,76656
842	134,646	93,96681
844	135,0848	94,09422
846	135,3355	94,09383
848	135,2102	94,19173
850	134,8341	94,26035
852	136,2131	94,37449
854	135,7743	94,36655
856	136,0251	94,39606
858	136,2758	94,2436
860	136,6519	94,16814
862	136,5892	94,24973
864	136,1504	94,19414
866	137,4041	94,18758
868	136,84	94,24882
870	137,2161	94,17731
872	136,3385	94,33051
874	137,6549	94,35793
876	137,6549	94,14767
878	137,8429	94,24396
880	138,2817	94,26063
882	138,9712	94,31171
884	138,219	94,5294
886	138,6578	94,75008
888	138,2817	95,2067
890	138,8459	95,41581
892	138,031	95,54338
894	139,2847	95,82143
896	139,7234	96,04117
898	139,3474	96,20232
900	139,2847	96,27603
902	139,1593	96,03307
904	138,2817	96,33344
906	138,5325	96,4603
908	139,0339	96,65728
910	137,5922	96,7837
912	138,2817	96,90708
914	138,7205	97,00826
916	139,0339	97,3987
918	140,0369	97,52639
920	140,2876	97,72104
922	139,41	97,75316
924	138,5951	97,7182
926	139,4727	97,81661

928	139,222	97,97815
930	139,9742	98,05151
932	140,3503	98,10806
934	140,601	98,1205
936	140,3503	98,18868
938	139,7234	98,38701
940	141,1025	98,47247
942	140,0996	98,50198
944	139,222	98,53671
946	140,2876	98,90763
948	140,9771	98,93643
950	140,4757	99,21772
952	139,7234	99,51656
954	139,5981	99,76193
956	140,2876	100
958	139,41	100,1742
960	140,1622	99,88602
962	142,6696	100,2311
964	142,2935	100,1425
966	142,6069	100,1666
968	142,9203	100,2319
970	142,6069	100,3588
972	142,9203	100,6197
974	142,2935	100,8251
976	141,9801	100,9451
978	143,7352	101,0962
980	144,1114	101,2349
982	143,1084	101,3672
984	144,174	101,5692
986	144,4875	101,6884
988	144,5501	102,0579
990	143,7979	102,0471
992	143,8606	102,1982
994	143,1084	102,282
996	143,5472	102,3662
998	143,3591	102,3232
1000	144,9889	102,3647
1002	143,2965	102,368
1004	144,4875	102,5048
1006	144,6755	102,6093
1008	145,1143	102,6072
1010	144,7382	102,6548
1012	145,5531	102,7287
1014	145,7411	102,6519
1016	145,9292	102,5894
1018	145,9919	102,6303
1020	146,0546	102,5428
1022	146,0546	102,468
1024	146,1173	102,3198
1026	148,1858	102,1457
1028	146,4934	102,0521
1030	146,4934	101,9219

1032	146,6187	101,7823
1034	147,3083	101,7544
1036	145,6158	101,6634
1038	144,9263	101,5283
1040	146,556	101,5636
1042	147,1202	101,6096
1044	146,368	101,6441
1046	147,6217	101,6971
1048	147,6844	101,753
1050	147,559	101,9757
1052	147,9351	102,1163
1054	147,7471	102,3371
1056	147,6217	102,5968
1058	146,3053	102,9257
1060	147,1829	102,9395
1062	147,4336	103,092
1064	146,9322	103,1454
1066	147,8097	103,2721
1068	148,0605	103,1476
1070	147,9978	103,1864
1072	147,2456	103,2813
1074	147,3709	103,4935
1076	148,0605	103,6321
1078	148,2485	103,7987
1080	148,1232	103,9605
1082	147,9351	104,2428
1084	148,3739	104,3018
1086	147,9351	104,2501
1088	148,1858	104,2397
1090	148,3112	104,1321
1092	148,6246	103,9816
1094	148,2485	103,9657
1096	148,4993	103,9067
1098	148,938	103,9189
1100	148,3739	103,9816
1102	148,6246	103,9392
1104	148,8127	103,9306
1106	149,8156	104,0328
1108	149,1261	104,0797
1110	148,3739	103,9929
1112	149,0634	103,9194
1114	149,1888	103,8737
1116	149,5022	104,1081
1118	148,8754	104,1864
1120	149,5649	104,2386
1122	150,1917	104,2773
1124	149,4395	104,4481
1126	150,1917	104,5007
1128	150,1291	104,4355
1130	150,4425	104,3831
1132	151,0693	104,5616
1134	151,7589	104,9152

1136	151,132	105,1968
1138	152,4484	105,4902
1140	152,0723	105,6186
1142	152,0096	105,9731
1144	151,132	106,0374
1146	152,2603	106,0307
1148	152,5111	105,6901
1150	152,8245	105,7093
1152	152,9499	105,7909
1154	153,514	106,1425
1156	153,3886	106,353
1158	153,7021	106,8549
1160	153,326	107,2696
1162	153,4513	107,9698
1164	153,7648	108,2844
1166	154,6423	108,8197
1168	154,6423	109,0704
1170	155,8333	109,3992
1172	155,708	109,5457
1174	154,3289	109,5785
1176	154,9557	109,6813
1178	155,3319	110,0289
1180	155,2065	109,8728
1182	155,1438	110,1723
1184	156,2721	110,6616
1186	154,9557	110,9309
1188	156,1468	111,24
1190	156,1468	111,4562
1192	156,3975	111,4558
1194	156,2094	111,5837
1196	156,2721	111,5571
1198	156,6482	111,1995
1200	157,7139	111,0341
1202	156,6482	111,0461
1204	157,4004	110,9806
1206	156,5229	110,8957
1208	156,899	110,8556
1210	156,8363	110,7653
1212	158,8422	110,7062
1214	158,6541	110,8346
1216	157,8392	110,9093
1218	159,5317	110,8941
1220	156,899	111,0335
1222	157,5885	111,1002
1224	158,09	111,2111
1226	158,09	111,553
1228	157,3378	111,8177
1230	157,4631	112,1536
1232	157,7139	112,5594
1234	158,6541	112,7028
1236	158,278	113,1709
1238	159,7198	113,6611

1240	158,4661	113,9358
1242	157,1497	114,2332
1244	159,2183	114,2289
1246	157,1497	114,4712
1248	159,5317	114,9112
1250	158,5914	115,1009
1252	159,7198	115,294
1254	160,0332	115,2806
1256	158,1526	115,344
1258	160,7227	115,6216
1260	160,5347	115,599
1262	161,0988	115,6184
1264	160,2212	115,513
1266	160,2212	115,6274
1268	160,2839	115,7237
1270	160,7227	115,8
1272	161,2869	116,0416
1274	160,4093	116,2717
1276	162,3525	116,6551
1278	162,4779	117,0397
1280	163,6689	117,189
1282	163,7943	117,657
1284	163,2301	118,0975
1286	163,5435	118,2438
1288	164,9852	118,4637
1290	164,9852	118,5488
1292	163,3555	118,7378
1294	165,6121	118,9238
1296	165,1733	118,8659
1298	166,3643	118,9522
1300	165,6748	118,7205
1302	167,2419	118,6031
1304	165,1733	118,7963
1306	168,2448	119,0526
1308	167,1165	118,9078
1310	168,4329	119,0456
1312	169,6866	118,8854
1314	165,9255	119,2835
1316	165,9255	119,4063
1318	169,9373	119,2729
1320	165,3614	119,1843
1322	166,8031	119,5263
1324	165,6121	119,7285
1326	166,6151	119,9379
1328	167,4926	120,0403
1330	167,0538	120,2569
1332	166,2389	120,6018
1334	170	120,473
1336	171,3164	120,4964
1338	170,6895	120,6138
1340	170,4388	120,5367
1342	169,3105	120,7304

1344	169,1851	121,1349
1346	171,0656	121,5379
1348	173,3223	122,1544
1350	170,0627	122,7028
1352	172,2566	123,1404
1354	172,4447	124,1692
1356	173,0089	124,7061
1358	171,8805	125,0879
1360	170	125,3802
1362	172,5701	125,7797
1364	172,8208	126,0156
1366	173,1342	126,531
1368	173,0715	126,6835
1370	174,6386	126,8102
1372	174,576	127,0355
1374	175,0148	127,0972
1376	173,8864	127,5039
1378	175,5162	127,7713
1380	173,573	127,9431
1382	174,5133	128,2552
1384	174,1999	128,729
1386	175,8923	129,0302
1388	177,0206	129,4134
1390	175,3909	129,5312
1392	176,2684	129,8784
1394	177,3968	130,086
1396	176,2057	130,0235
1398	177,8355	130,234
1400	176,1431	130,3682
1402	174,764	130,5016
1404	177,8982	130,573
1406	176,8326	130,5426
1408	176,7072	130,4146
1410	177,7102	130,3784
1412	178,5251	130,3576
1414	178,9012	130,5241
1416	178,2117	130,7
1418	178,3997	130,7803
1420	178,5878	130,895
1422	179,2773	131,0103
1424	179,0892	131,2496
1426	178,149	131,2041
1428	180,3429	131,3914
1430	178,9639	131,6007
1432	179,7788	131,9162
1434	180,0295	132,0221
1436	178,9639	132,4678
1438	180,2802	133,0483
1440	180,719	133,4422
1442	179,9041	133,7443
1444	179,7788	133,9847
1446	180,3429	134,4562

1448	179,2773	135,3253
1450	179,7788	135,8861
1452	181,4086	136,404
1454	180,6563	137,0683
1456	181,2832	137,6714
1458	180,8444	138,1699
1460	182,3488	138,5077
1462	181,2205	138,8566
1464	182,8503	139,1013
1466	183,101	139,1273
1468	184,6681	139,1308
1470	184,8562	139,137
1472	184,5428	139,1747
1474	183,8532	139,2349
1476	183,9159	139,0761
1478	184,4801	139,0715
1480	183,7279	139,2811
1482	184,0413	139,539
1484	184,6055	139,5178
1486	183,9786	139,4383
1488	184,7308	139,4648
1490	185,0443	139,5245
1492	184,9189	139,7933
1494	184,9189	139,6152
1496	184,9816	139,6424
1498	185,1069	139,5816
1500	185,2323	139,8106
1502	185,2323	139,9294
1504	186,6114	139,8949
1506	186,1726	139,9203
1508	186,7994	140,053
1510	186,8621	140,1461
1512	186,7994	140,4205
1514	187,0501	140,5362
1516	187,0501	140,6683
1518	186,486	141,0166
1520	186,6114	140,9045
1522	187,0501	141,007
1524	187,0501	141,2415
1526	187,3636	141,3667
1528	187,9277	141,6405
1530	187,8651	141,7744
1532	188,868	141,5864
1534	187,8651	141,795
1536	188,0531	142,1248
1538	188,3665	142,3198
1540	188,4919	142,5968
1542	190,2471	142,4812
1544	188,7426	142,228
1546	188,8053	142,3174
1548	187,8651	142,2974
1550	188,4919	142,2207

1552	189,1814	141,9101
1554	188,6173	141,6786
1556	189,3695	141,4697
1558	190,3724	141,3885
1560	188,868	141,4943
1562	189,3695	141,5526
1564	191,3754	141,5469
1566	190,8112	141,7217
1568	190,5605	141,8663
1570	191,25	142,2295
1572	190,4978	142,6317
1574	190,5605	142,6559
1576	191,7515	142,6349
1578	191,5634	142,5256
1580	193,8201	142,2708
1582	191,7515	142,0579
1584	191,1246	142,0295
1586	191,8142	142,092
1588	191,7515	142,0713
1590	193,3186	142,1584
1592	193,5693	142,2129
1594	193,9454	142,4603
1596	193,2559	142,8391
1598	193,6947	143,0251
1600	194,0708	143,122
1602	194,5096	143,4175
1604	194,823	143,6041
1606	195,4499	143,9305
1608	197,2677	144,164
1610	197,017	144,1617
1612	197,8945	144,2315
1614	197,5184	144,1828
1616	197,017	143,9851
1618	197,7065	144,0297
1620	197,017	144,1438
1622	197,2677	144,2974
1624	197,5811	144,2229
1626	196,4528	144,2343
1628	198,0199	144,6115
1630	197,3304	144,8822
1632	197,7065	144,9435
1634	196,9543	144,9462
1636	197,7065	144,7781
1638	197,017	145,0164
1640	197,4557	144,8781
1642	197,7065	145,0715
1644	197,205	145,2339
1646	197,7065	145,5937
1648	199,7124	145,5597
1650	198,208	145,8164
1652	197,9572	146,0158
1654	198,5214	146,397

1656	198,7094	146,2601
1658	198,7721	146,1778
1660	198,9602	146,0931
1662	198,5841	146,2429
1664	199,7751	146,4899
1666	199,0229	146,6454
1668	199,8378	146,8132
1670	199,8378	146,8347
1672	201,4675	146,9912
1674	200,4646	147,2322
1676	200,778	147,4895
1678	200,59	147,5714
1680	200,9034	147,8033
1682	199,8378	147,9771
1684	200,4646	148,5449
1686	201,0914	148,7149
1688	200,778	148,7776

Figure 3e **ECIS murine cells (resistance ohm)**

time	hTNFtg FLS	hTNFtg/*Lasp1-/-* FLS
0,0036	9514	12323
0,0036	9514	12323
0,0783	11048	13151
0,1532	12641	14275
0,2281	13907	15703
0,3029	14615	16590
0,3776	14972	17198
0,4524	15301	17332
0,5271	15754	17427
0,602	15988	17524
0,6769	15520	17727
0,7516	15411	17774
0,8264	15488	17758
0,9012	15421	17623
0,976	15447	17567
1,0506	15291	17607
1,1253	15363	17898
1,2	15144	17988
1,2746	15069	18259
1,3493	15045	18208
1,424	15298	17992
1,4987	15355	18022
1,5733	15469	17958
1,648	15478	17717
1,7227	15725	17520
1,7974	15462	17487
1,8721	15430	17068
1,9468	15475	16934
2,0214	15442	17039
2,0961	15393	16932
2,1708	15219	16595
2,2454	14955	16637
2,3201	14646	16327
2,3948	14597	16216
2,4694	14629	15877
2,5441	14619	15462
2,619	14339	15498
2,6936	14251	15569
2,7683	13956	15509
2,843	13952	15306
2,9176	13886	15157
2,9923	13741	15013
3,067	13556	14960
3,1416	13627	14578
3,2165	13357	14399
3,2912	13293	14314
3,366	13310	14422
3,4407	13329	14184

3,5156	13308	14156
3,5902	13391	14177
3,6649	13190	14087
3,7395	13118	14110
3,8142	13064	13933
3,8891	13050	13784
3,9637	12999	13995
4,0384	12949	14004
4,113	12973	13756
4,1877	12955	13699
4,2623	12995	13432
4,337	13023	13475
4,4119	13191	13256
4,4867	13262	13464
4,5616	13337	13399
4,6364	13506	13393
4,7113	13500	13076
4,7863	13479	13229
4,861	13452	13128
4,9358	13269	13174
5,0106	13244	13122
5,0855	13436	13067
5,1603	13392	13133
5,2352	13402	13049
5,3098	13426	13060
5,3847	13367	12778
5,4595	13460	12685
5,5344	13488	12704
5,6092	13225	12706
5,6839	13126	12818
5,7585	13045	12693
5,8332	13276	12523
5,9081	13321	12569
5,983	13057	12461
6,0579	12939	12649
6,1327	12860	12537
6,2076	12913	12548
6,2825	12766	12630
6,3575	12837	12771
6,4324	12677	12664
6,5074	12498	12656
6,5823	12590	12886
6,6572	12485	12707
6,732	12372	12530
6,8069	12299	12513
6,8817	12105	12386
6,9566	12106	12428
7,0315	12064	12235
7,1065	11943	12237
7,1814	11878	12371
7,2564	11996	12435
7,3315	12155	12359

7,4065	12108	12317
7,4816	12015	12279
7,5565	11899	12282
7,6314	11862	12354
7,7063	11762	12175
7,7812	11640	12081
7,856	11810	12042
7,9309	12048	12081
8,0058	12027	11989
8,0807	12077	12052
8,1555	12039	12043
8,2304	11900	11919
8,3054	11751	11853
8,3803	11784	11822
8,4554	11617	12047
8,5302	11496	11879
8,6053	11379	12052
8,6803	11413	12165
8,7554	11208	12159
8,8304	11136	12249
8,9055	11073	12175
8,9805	11074	12097
9,0556	10982	12049
9,1306	11036	12080
9,2055	11064	12046
9,2804	11154	12209
9,3553	11193	12060
9,4302	11135	12038
9,5051	11174	12028
9,58	11165	12066
9,6548	11299	11872
9,7297	11275	11927
9,8046	11398	11855
9,8795	11292	11820
9,9543	11116	11966
10,0294	10941	12085
10,1042	10905	12272
10,1793	10965	12205
10,2541	11045	12383
10,329	10896	12340
10,4039	10833	12186
10,4787	10891	12242
10,5536	10789	12108
10,6286	10800	11959
10,7037	10813	12084
10,7787	10803	12170
10,8536	10827	12199
10,9286	10804	12316
11,0035	10737	12353
11,0784	10673	12292
11,1533	10671	12338
11,2282	10603	12399

11,303	10445	12489
11,3781	10366	12534
11,453	10368	12428
11,5279	10209	12511
11,6027	10180	12379
11,6776	10235	12323
11,7525	10095	12186
11,8273	10090	12173
11,9022	10172	12133
11,9773	10223	12176
12,0523	10273	12182
12,1274	10309	12144
12,2024	10345	12227
12,2775	10346	12234
12,3525	10492	12319
12,4274	10389	12355
12,5024	10287	12321
12,5773	10171	12297
12,6524	10295	12305
12,7272	10336	12277
12,8021	10406	12404
12,877	10478	12454
12,9519	10520	12294
13,0268	10522	12338
13,1017	10640	12332
13,1767	10676	12325
13,2516	10643	12603
13,3265	10510	12641
13,4013	10500	12769
13,4764	10583	12546
13,5514	10524	12552
13,6263	10513	12528
13,7013	10521	12663
13,7762	10428	12742
13,851	10409	12672
13,9259	10400	12717
14,001	9781	12466
14,0759	9855	12384
14,1509	9906	12284
14,2258	9910	12348
14,3008	10007	12421
14,3757	9972	12463
14,4506	10008	12481
14,5254	10015	12472
14,6003	10001	12392
14,6752	10118	12427
14,7501	10092	12436
14,825	10224	12440
14,8998	10254	12445
14,9747	10283	12568
15,0498	10271	12655
15,1246	10253	12588

15,1995	10399	12337
15,2743	10392	12353
15,3492	10391	12310
15,4241	10427	12300
15,4989	10372	12254
15,5738	10378	12289
15,6489	10286	12361
15,7237	10325	12566
15,7988	10296	12500
15,8736	10270	12502
15,9487	10237	12321
16,0237	10170	12282
16,0986	10170	12410
16,1736	10177	12599
16,2485	10184	12814
16,3234	10144	12779
16,3982	10188	12708
16,4731	10228	13022
16,548	10235	12920
16,6229	10236	13038
16,6978	10258	13240
16,7726	10189	13197
16,8475	10057	13208
16,9224	10029	13372
16,9974	10118	13242
17,0723	10213	13337
17,1474	10143	13278
17,2224	10081	13315
17,2974	10230	13319
17,3725	10282	13301
17,4475	10409	13200
17,5226	10292	13247
17,5974	10246	13368
17,6725	10059	13312
17,7475	10146	13312
17,8225	10108	13408
17,8974	10187	13385
17,9723	10212	13505
18,0472	10187	13322
18,1221	10250	13389
18,197	10282	13308
18,2718	10325	13278
18,3467	10281	13084
18,4216	10369	12982
18,4964	10465	12978
18,5713	10498	12922
18,6462	10416	12824
18,7212	10396	12818
18,7961	10310	12682
18,8709	10543	12712
18,9458	10507	12861
19,0207	10483	12831

19,0955	10276	12824
19,1704	10247	12656
19,2453	10243	12714
19,3201	10321	12728
19,395	10296	12697
19,47	10288	12589
19,5451	10163	12622
19,6201	10210	12534
19,695	10326	12411
19,7698	10558	12511
19,8447	10861	12580
19,9196	11042	12600
19,9944	10792	12488
20,0693	10623	12466
20,1442	10493	12475
20,2191	10510	12375
20,294	10556	12440
20,3688	10566	12351
20,4437	10426	12277
20,5188	10430	12203
20,5936	10420	12114
20,6687	10403	12091
20,7435	10413	12013
20,8185	10398	12024
20,8936	10444	11981
20,9686	10527	11984
21,0437	10373	12021
21,1187	10350	11964
21,1938	10490	11978
21,2686	10451	11876
21,3437	10443	11978
21,4187	10487	12084
21,4937	10436	12165
21,5686	10396	12143
21,6435	10300	12334
21,7184	10261	12304
21,7933	10284	12281
21,8681	10313	12257
21,943	10218	12318
22,0179	10277	12358
22,0927	10297	12325
22,1676	10238	12342
22,2425	10274	12422
22,3173	10183	12438
22,3924	10176	12496
22,4672	10115	12402
22,5421	10141	12426
22,6169	10091	12538
22,6918	10165	12531
22,7667	10249	12586
22,8415	10249	12506
22,9164	10141	12652

22,9913	10168	12714
23,0663	10231	12586
23,1413	10300	12403
23,2161	10432	12366
23,2909	10515	12195
23,3658	10808	12149
23,4404	10685	12270
23,5153	10679	12438
23,5901	10609	12377
23,6649	10528	12421
23,7396	10586	12498
23,8142	10590	12585
23,8889	10498	12716
23,9636	10509	12745
24,0383	10572	12889
24,113	10546	13006
24,1877	10455	13023
24,2624	10506	13110
24,337	10522	12971
24,4117	10499	13015
24,4864	10512	13174
24,5611	10446	13078
24,6358	10363	12975
24,7105	10308	13069
24,7852	10359	12935

Figure 3g **Retraction (per time)**

hTNFtg FLS	hTNFtg/*Lasp1-/-* FLS
-2,978495	-5,966102
-7,514286	-3,583333
-1,848837	-2,784615
-1,435897	-2,816092
-1,318519	-8,236363
-1,923077	-3,783333
-1,597315	-3,829268
-2,837209	-3,068965
-2,196532	-3,446154
-3,329032	-4,589041
-1,458904	-3,202532
-4,457143	-2,777778
-1,401961	-4,130435
-1,22619	-3,149254
-1,510417	-2,753425
-1,653061	-4,638889
-1,135593	-3,590909
-1,53	-5,926316
-2,474026	-3,072289
-1,067568	-2,728571
-1,962963	-5
-1,603896	-2,682353
-2,386243	-3,921053
-2,127119	-5,96
	-4,157895
	-5,054054
	-3,956522
	-4,609091
	-8,62963
	-5,203125

Extension (per time)

hTNFtg FLS	hTNFtg/*Lasp1-/-* FLS
2,647059	4,298701
6,116279	3,5625
2,048611	2,869565
1,524138	3,571429
1,434108	7,278481
2,045871	4,384615
1,483222	3,410959
3,050725	3,277778
2,346939	3,333333
3,410714	5,19403
1,638655	3,202899
5,904762	2,652174
1,530612	5,8
1,256757	3,169231
1,365854	2,345679
1,561404	4,380531
1,1	4,771084
1,300971	6,73913
2,666667	4,25
3,737374	4,205883
2,157895	9,372093
2,108108	2,947368
2,467626	4,206349
2,03125	5,171429
	5,511111
	5,280488
	2,964286
	4,945206
	6,916667
	3,064815

Figure 4a Immunoblotting for adherens junction proteins

Cadherin-11 expression

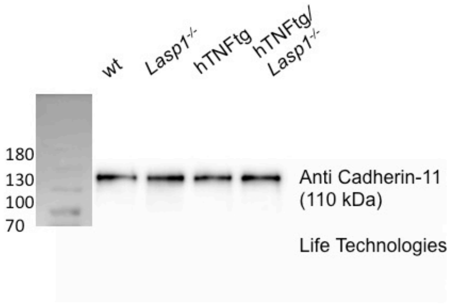

07.09.17

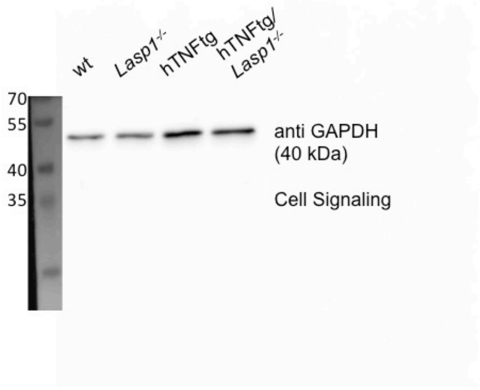

08.09.17

ß-Catenin Expression

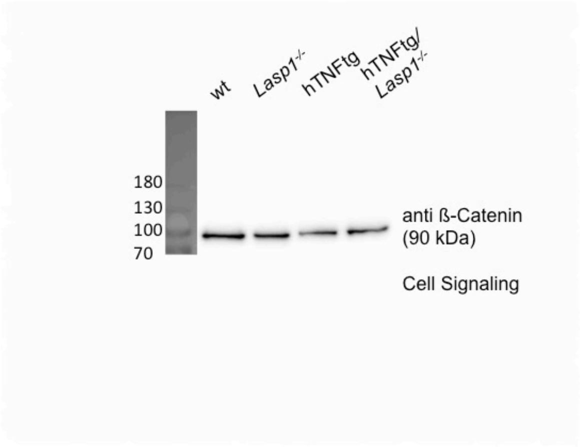

07.09.17

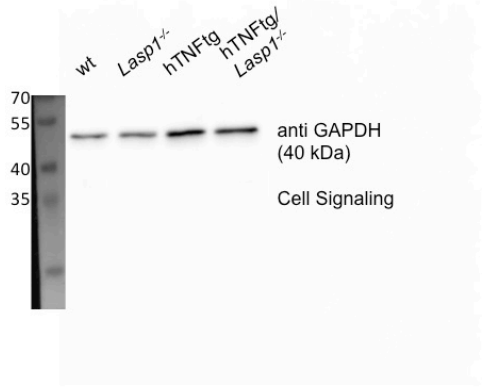

08.09.17

p120 expression

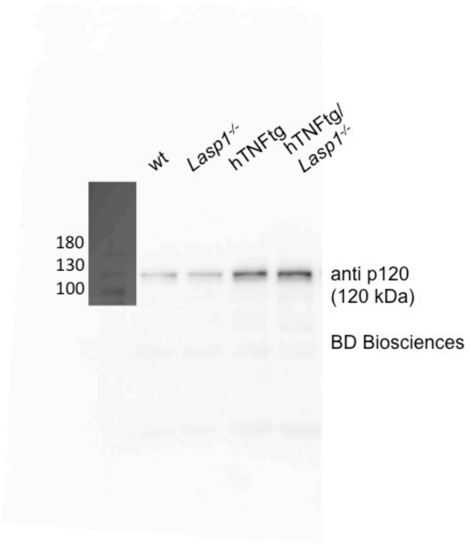

07.09.17

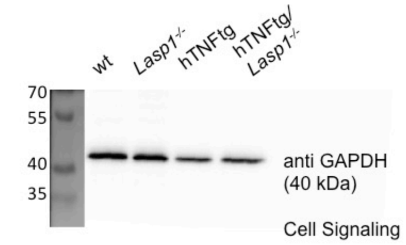

08.09.17

Figure 4b **Evaluation catenin expression pattern (sigma arb. units)**

hTNFtg FLS	hTNFtg/*Lasp1-/-* FLS
177	86
265	102
183	111
198	72
235	88
247	84
201	67
189	45
179	85
147	82
234	74
42	49
352	3
341	90
280	68
287	37
261	51
296	35
204	83
272	86
126	35
234	61
171	83
253	42
	33
	66
	148
	39
	23
	50

Figure 4c **Evaluation paxillin expression pattern (sigma arb. units)**

hTNFtg FLS	hTNFtg/*Lasp1-/-* FLS
376,5413	2693,493
208,7109	3200,584
232,3999	2786,166
302,5555	2015,258
336,455	1358,752
287,9312	1000,788
229,7308	1568,635
459,7326	1501,499
204,9305	1508,558
244,7007	2310,471
348,1205	1771,917
291,2025	1262,536
141,6213	1964,911
217,5767	924,3433
200,3596	961,3948
184,8968	1564,656
194,9411	1247,727
150,7066	2394,892
220,2689	1292,934
422,1725	4054,39
316,9942	3192,832
242,4768	1279,212
266,3568	2642,16
414,7472	5615,298
139,4357	1870,006
378,5877	1982,588
270,7061	2597,473
448,4899	3158,109
655,8609	1477,681
416,7563	2298,466
523,7132	2367,666
307,9212	963,5899
387,8976	1759,968
239,1063	1652,174
294,5979	1525,86
384,5105	1697,92
162,914	3532,663
301,5031	2637,488
421,7576	2328,683
140,7808	3447,452
289,1924	2728,401
267,447	2507,189
237,8935	2445,674
604,7309	3235,361
461,7687	1489,168
380,0751	4985,328
635,8204	5376,827
327,0759	2252,203

143,6391	3514,853
4019,083	2462,675
520,2087	2287,003
833,9373	3083,376
936,7808	970,269
970,1209	2789,662
1257,104	2898,32
1483,476	3112,585
1536,334	1684,351
1545,209	1932,996
1587,143	2294,648
1605,791	3514,518
1644,556	1808,817
1745,881	1346,354
1761,956	1267,963
1817,382	0,5299581
1920,926	0,4111382
1941,615	0,2192119
1943,864	0,2699221
1968,243	4575,518
2011,705	3740,307
2025,417	3729,911
2030,278	3285,042
2036,905	2728,135
2090,663	1686,084
2094,551	2728,748
2128,761	2694,241
2238,562	4059,163
2239,619	3845,137
2241,181	2695,883
2258,314	3093,136
2285,242	1994,669
2304,78	1458,84
2311,637	2128,677
2323,328	3098,497
2340,783	1281,604
2368,305	1905,077
2384,261	1772,055
2435,64	1802,369
2450,367	1653,652
2466,595	2372,011
2548,277	1681,235
2578,07	2230,915
2612,703	963,9384
2621,805	1190,786
2638,061	1057,451
2643,379	2688,495
2679,445	1534,539
2687,079	1801,95
2688,363	2743,588
2708,771	2603,659
2729,965	2226,367

2743,717	2352,661
2804,366	3877,388
2905,947	
2921,764	
2928,519	
2948,585	
3012,982	
3183,157	
3336,995	
3361,164	
3497,428	
3520,587	
3528,358	
3555,787	
3556,674	
3693,119	
3739,392	
3776,392	
3891,742	
3971,58	
3979,645	
4041,541	
4058,69	
4140,748	
4146,186	
4190,477	
4209,083	
4332,137	
4359,821	
4476,237	
4494,466	
4547,559	
4610,628	
4804,934	
4902,583	
4999,196	
5359,359	
5401,92	
5507,707	
5595,676	
5729,252	
6158,899	
6282,179	
6964,677	

Figure 5a Immunoblotting for Lasp1 and ß-Catenin interaction

Cadherin-11 expression

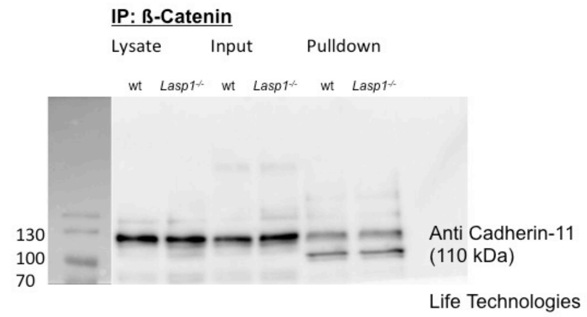

09.11.16

ß-Catenin expression

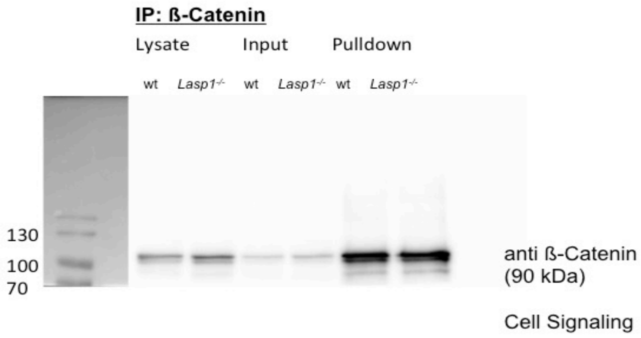

08.11.16

p120 expression

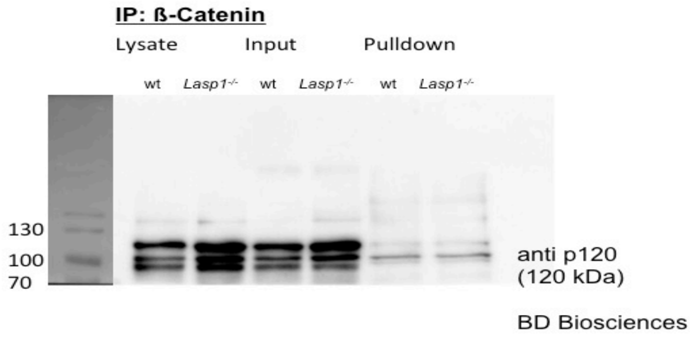

11.11.16

Lasp1 expression

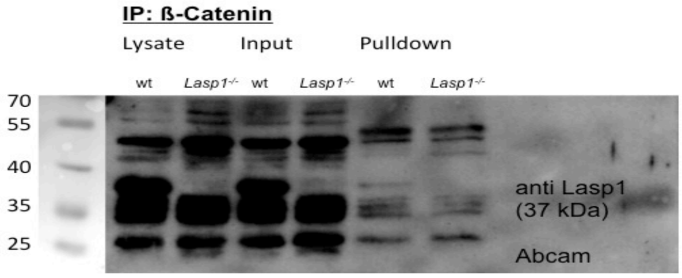

09.11.16

ß-Actin expression

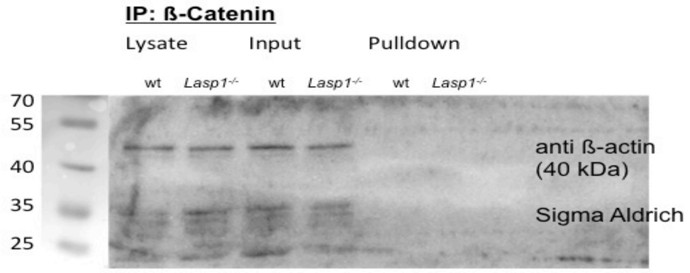

08.11.16

Figure 5b Immunoblotting for Lasp1 and ß-Catenin interaction

Cadherin-11 expression

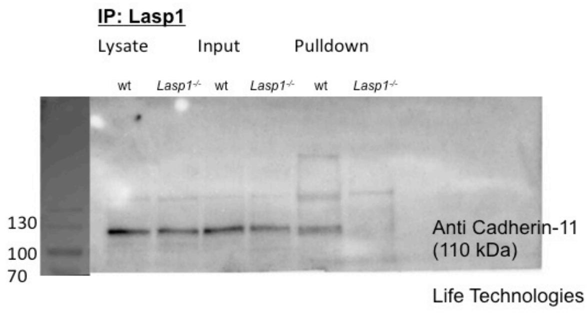

11.11.16

ß-Catenin expression

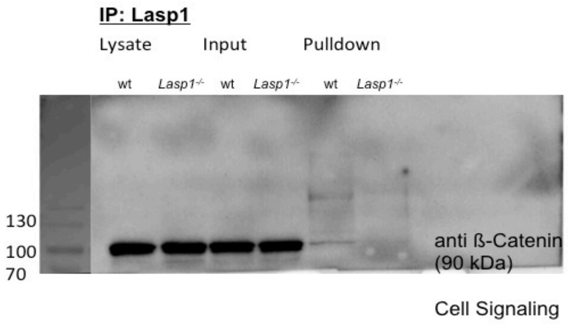

09.11.16

p120 expression

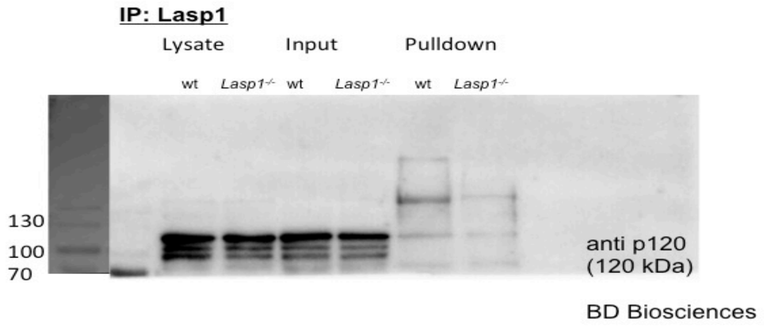

10.11.16

Lasp1 expression

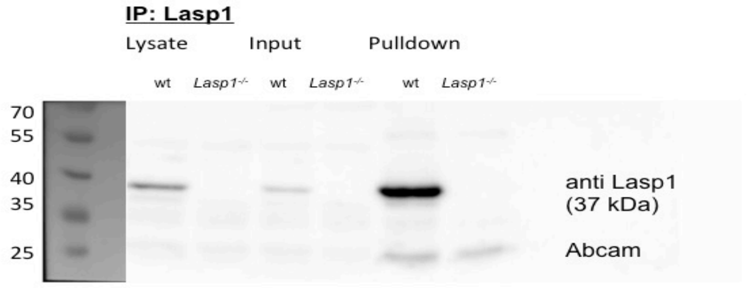

09.11.16

ß-Actin expression

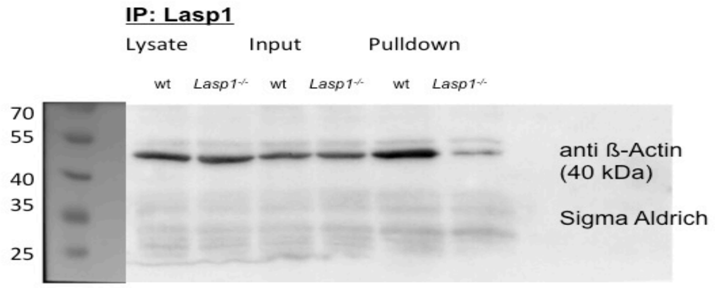

08.11.16

Figure 5f Immunoblotting for Lasp1 and ß-Catenin interaction

GFP expression -short exposure-

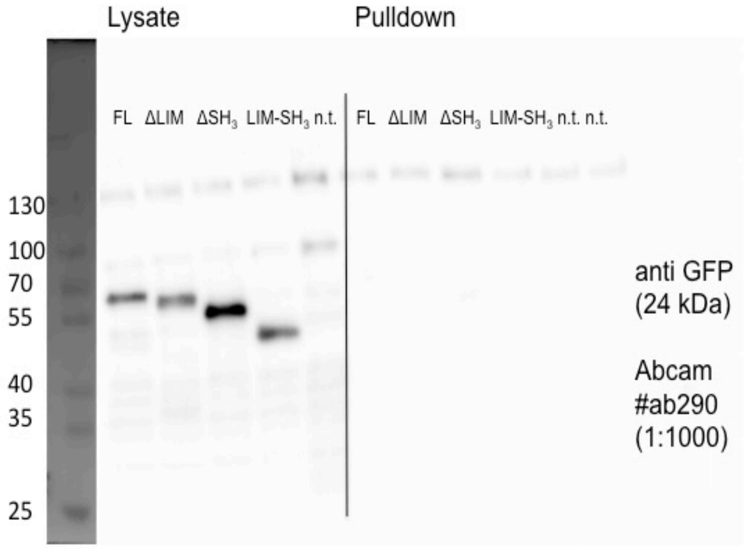

11.08.17

GFP expression -long exposure-

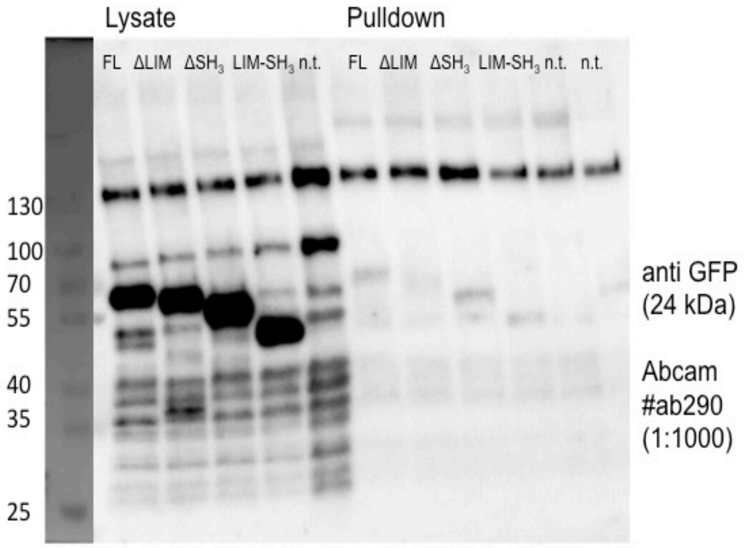

11.08.17

Figure 5g Immunoblotting for Lasp1 expression in FLS after CDBP1728 treatment

Lasp1 expression

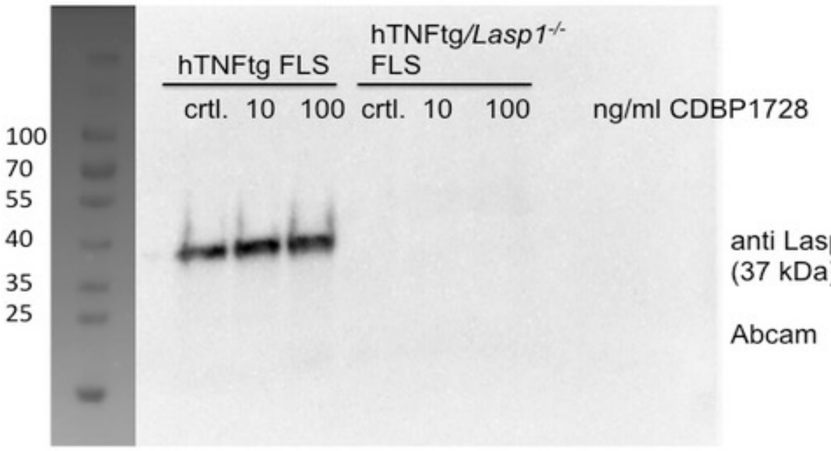

17.01.18

GAPDH expression

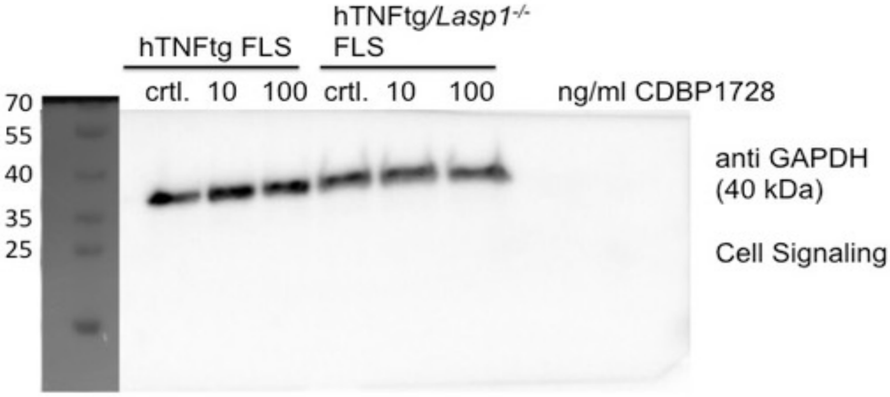

18.01.18

Figure 5h **Migration rate (% closed area in 24hrs)**

hTNFtg FLS

0 (ctrl)	1	10	100	ng/ml CDBP1728
47,85	47,98	28,73	23,05	
46,7	44,93	24,53	22,48	
49,55		21,96	13,96	
51,9		19,42	13,69	

hTNFtg/*Lasp1-/-* FLS

0 (ctrl)	1	10	100	ng/ml CDBP1728
24,96	28,1	35,26	27,71	
23,7	27,3	34,71	31,86	
21,15		33,01	28,93	
30,05		43,43	40,74	

Fig 6b Clinical scoring evaluations over the disease course

weight (g)

wt		animals 1	2	3	4	5
weeks	6	15,1	13,9	11,8	12,9	15,1
	7	15,3	16,8	15,4	15,1	18,3
	8	17,3	17,1	17,2	16	20,4
	9	17,8	17,8	18,1	16,9	21,2
	10	19,4	19,6	18,8	17,8	22,1
	11	19,4	19,5	18,9	19,1	23,3
	12	20,5	20,3	20	19,2	23,9
	13	21,1	20,9	21,6	20,5	24,4
	14	20,5	21,3	22,5	21,7	25,6

Lasp1-/-		animals 1	2	3	4	5	6	7	8	9
weeks	6	19,7	16,7	15,3	18,9	18,7	19,7	16,9	22	19,8
	7	20,6	18,6	18,6	20	20,7	18,7	23,9	19	23,7
	8	23	19,5	20,4	22	21,7	19,9	25,6	17,6	18
	9	22,6	19,4	20	21,7	21,5	20,9	26,7	21,8	27
	10	21,7	19,5	22,3	20,2	19,9	21,5	25,2	23,3	23,6
	11	19,3	19,7	26,1	19	19,5	21,9	24,7	25,6	18,4
	12	19,4	19,6	23,5	19,7	20	22,4	25,1	25,5	
	13	19,4	20,4	23,1	20,8	21,4	26,1			
	14	20,6	20,5	23,3	22,6	22	26			

hTNFtg		animals 1	2	3	4	5	6	7	8	9	10
weeks	6	15,1	15,7	11,4	14,7	13,3	12,9	12,2	12,7	12,5	10,8
	7	15,6	15,8	14	15,3	14,3	13,6	13	13,8	13,1	11,4
	8	18,5	14,6	13,8	15,5	14,6	14,1	12,8	14,7	14	12
	9	18,1	16,8	14,6	15,8	14,8	14,1	13,2	14,7	14	11,9
	10	18,5	17,1	14,9	16	15	14,3	13,2	14,4	14,6	12,7
	11	19,2	15,6	13,6	16		14,3	13,9	14,6	14,3	13,4
	12	18,9	13,7	13,6	16,3		14,4	14,4	14,5	15	12,9
	13	18,2		13,3	15,1		14,4	14	14,4	14,2	12,9
	14	18,7					13,5	12,8	15,1	14	12

		animals									
**hTNFtg/*Lasp1-/-***		**1**	**2**	**3**	**4**	**5**	**6**	**7**	**8**	**9**	**10**
**weeks**	**6**	13,9	10,7	18	17	17,3	16,3	14,9	15,5	16,3	13,7
	**7**	15,1	11,6	17,9	17,6	17,3	17,3	15,4	16,1	17,4	14,1
	**8**	15,3	11,7	17,7	15,3	16,3	17,1	15,7	17,2	18,3	14,9
	**9**	15,8	12,2	19,1	17,6	18,1	16,2	16,9	18	18,9	16,4
	**10**	15	13	18,7	17,6	18,4	18,2	17,3	18,1	19,1	15,4
	**11**	16,5	12,7	18,9	18,1	18,9	17,4	13,3	18,4	18,9	15
	**12**	15,9	12,5	19,7	19,3	18,3	17,1	12,2	18	18,1	14,6
	**13**	16,4	12	20,7	20,6	17,8	16,9	11,1	17,6	18,2	15,4
	**14**	15,8	12,5	19,9	20	17,4	17,3	10,9			

<u>grip strength (au)</u>

		animals				
**wt**		**1**	**2**	**3**	**4**	**5**
**weeks**	**6**	0	0	0	0	0
	**7**	0	0	0	0	0
	**8**	0	0	0	0	0
	**9**	0	0	0	0	0
	**10**	0	0	0	0	0
	**11**	0	0	0	0	0
	**12**	0	0	0	0	0
	**13**	0	0	0	0	0
	**14**	0	0	0	0	0

		animals								
Lasp1-/-		**1**	**2**	**3**	**4**	**5**	**6**	**7**	**8**	**9**
**weeks**	**6**	0	0	0	0	0	0	0	0	0
	**7**	0	0	0	0	0	0	0	0	0
	**8**	0	0	0	0	0	0	0	0	0
	**9**	0	0	0	0	0	0	0	0	0
	**10**	0	0	0	0	0	0	0	0	0
	**11**	0	0	0	0	0	0	0	0	0
	**12**	0	0	0	0	0	0	0	0	0
	**13**	0	0	0	0	0	0	0	0	0
	**14**	0	0	0	0	0	0	0	0	0

	animals									
**hTNFtg**	**1**	**2**	**3**	**4**	**5**	**6**	**7**	**8**	**9**	**10**
**weeks 6**	-0,5	-0,5	-0,5	0	-1	-1	-1	-0,5	-0,5	-0,5
**7**	-1,5	-1	-1,5	-1	-1,5	-1	-1,5	-1	-1	-1
**8**	-1,5	-1,5	-1,5	-1	-1,5	-1,5	-1,5	-1,5	-1,5	-1,5
**9**	-2	-2	-2	-2	-1,5	-2	-2	-1,5	-2	-2
**10**	-2	-2,5	-2	-2	-2	-2	-2	-2	-2	-2
**11**	-2,5	-2,5	-2,5	-2,5	-2	-2,5	-2,5	-2	-2	-2
**12**	-2,5	-2,5	-2,5	-2,5	-2,5	-2,5	-2,75	-2,5	-2,5	-2,5
**13**	-2,75	-2,75	-2,75	-2,75	-2,75	-2,5	-2,75	-2,75	-2,75	-2,5
**14**	-2,5	-2,75	-2,5	-2,75	-2,75	-2,75	-2,75	-2,75	-2,75	-2,75

	animals									
**hTNFtg/*Lasp1-/-***	**1**	**2**	**3**	**4**	**5**	**6**	**7**	**8**	**9**	**10**
**weeks 6**	0	0	-1	-0,5	-0,5	0	-0,5	-0,5	-0,5	-0,5
**7**	0	-0,5	-1	-0,5	-0,5	-0,5	-0,5	-1	-0,5	-0,5
**8**	-1	-1	-1	-1	-0,5	-0,5	-1	-1	-0,5	-1
**9**	-1	-1,5	-1	-1	-0,5	-1	-1	-1	-1	-1
**10**	-1	-1,5	-1,5	-1,5	-1,5	-1	-1,5	-1,5	-1,5	-1,5
**11**	-1,5	-1,5	-2	-1,5	-1,5	-1,5	-1,5	-2	-1,5	-1,5
**12**	-2	-2	-2	-1,5	-1,5	-2	-1,5	-2	-2	-2
**13**	-2	-2	-2,5	-2	-2	-2,5	-2	-2,5	-2	-2
**14**	-2,5	-2,5	-2,75	-2	-2,5	-2,5	-2,5			

<u>paw swelling (au)</u>

	animals				
**wt**	**1**	**2**	**3**	**4**	**5**
**weeks 6**	0	0	0	0	0
**7**	0	0	0	0	0
**8**	0	0	0	0	0
**9**	0	0	0	0	0
**10**	0	0	0	0	0
**11**	0	0	0	0	0
**12**	0	0	0	0	0
**13**	0	0	0	0	0
**14**	0	0	0	0	0

	animals									
Lasp1-/-		**1**	**2**	**3**	**4**	**5**	**6**	**7**	**8**	**9**
**weeks**	**6**	0	0	0	0	0,5	0	0	0	0
	**7**	0	0,5	0	0	0	0	0	0	0
	**8**	0	0	0	0	0	0	0	0	0
	**9**	0	0	0	0	0	0	0	0	0
	**10**	0	0	0	0	0	0	0	0	0
	**11**	0	0	0	0	0	0	0	0	0
	**12**	0	0	0	0	0	0	0	0	0
	**13**	0	0	0	0	0	0	0	0	0
	**14**	0	0	0	0	0	0	0	0	0

	animals										
**hTNFtg**		**1**	**2**	**3**	**4**	**5**	**6**	**7**	**8**	**9**	**10**
**weeks**	**6**	0	0,5	0	0	0,5	0	0	0,5	1	0
	**7**	1	1	1	1	1,5	1	1	1	1	0,5
	**8**	1	1	1	1	1,5	1	1	1	1	1
	**9**	1,5	1,5	1,5	1,5	1,5	2	1,5	1,5	1,5	1,5
	**10**	2	2	2	1,5	2	1,5	2	2	2	1,5
	**11**	2	2	2	2	2,5	2	2	1,5	2	2
	**12**	2,5	2,5	2,5	2	2,5	2	2,5	2	2,5	2,5
	**13**	2,5	2,75	2,5	2,5	2,75	2,5	2,5	2,5	2,5	2,75
	**14**	2,75	2,75	2,75	2,5	2,75	2,5	2,75	2,75	2,75	2,75

	animals										
**hTNFtg/*Lasp1-/-***		**1**	**2**	**3**	**4**	**5**	**6**	**7**	**8**	**9**	**10**
**weeks**	**6**	0	0	0	0	0	0	0	0	0	0
	**7**	0	0,5	0	0	0	0	0	0	0	0
	**8**	0,5	0,5	0,5	0,5	0,5	0,5	0	0	0,5	0,5
	**9**	0,5	0,5	0,5	0,5	0,5	0,5	0,5	0,5	0,5	0
	**10**	0,5	0,5	0,5	0,5	1	1	1	1	1	0,5
	**11**	1	1	1,5	1	1	1,5	1,5	1	1,5	1,5
	**12**	2	1,5	1,5	2	1,5	1,5	1,5	1,5	1,5	1,5
	**13**	2	2	2	2	1,5	1,5	2	1,5	1,5	1,5
	**14**	2,5	2	2	2,5	2	2	2			

Figure 6c **Area of inflammation (%)**

wt	*Lasp1* $^{-/-}$	hTNFtg	hTNFtg/*Lasp1* $^{-/-}$
0,16	0,81	10,24	9,91
0,17	0,29	11,56	9,98
0,03	0,15	17,79	16,79
0,12	0,10	11,41	11,95
0,20	0,60	12,00	2,87
0,83	0,30	13,00	11,88
0,08	0,06	28,83	28,76
0,05	0,56	16,13	18,18
0,27		19,52	14,00
0,09		27,00	18,41

Length of attachment (mm)

wt	*Lasp1* $^{-/-}$	hTNFtg	hTNFtg/*Lasp1* $^{-/-}$
0,21	0,10	0,46	0,44
0,17	0,07	1,07	0,32
0,15	0,00	1,14	0,51
0,05	0,05	1,53	0,71
0,00	0,04	2,44	0,12
0,00	0,00	1,74	0,97
0,04	0,00	0,60	0,52
0,00	0,00	0,74	0,50
0,00		1,09	0,26
0,00		0,42	0,25

Destained cartilage (%)

wt	*Lasp1* $^{-/-}$	hTNFtg	hTNFtg/*Lasp1* $^{-/-}$
2,06	1,11	24,44	6,34
4,05	0,12	28,51	3,96
0,82	0,00	47,14	2,10
1,43	0,08	18,00	4,20
0,87	0,39	41,08	6,11
1,02	0,38	46,60	19,79
0,51	0,96	14,37	29,01
1,46	1,02	41,16	33,48
2,31		35,14	15,99
0,34		44,62	11,28

Figure 6d **Number of TRAP pos. Osteoclasts (in vivo)**

wt	*Lasp1* $^{-/-}$	hTNFtg	hTNFtg/Lasp1-/-
0	0	184	38
0	0	132	58
0	0	166	64
0	0	161	53
0	0	121	123
0	0	237	137
0	0	115	149
0	0	123	101
1		90	98
0		125	81

Figure 6e **RANKL/OPG ratio**

wt	*Lasp1* $^{-/-}$	hTNFtg	hTNFtg/*Lasp1* $^{-/-}$
0,03	0,06	0,32	0,01
0,00	0,00	0,19	0,02
0,00	0,13	0,22	0,16
0,05	0,05	0,25	0,13
0,10	0,05	0,21	0,20
0,06	0,04		

Figure 6f **Number of osteoclasts per well (in vitro assay)**

hTNFtg	hTNFtg/*Lasp1* $^{-/-}$
262	384
411	377
407	273
353	325
274	418
350	320
351	291
346	284
	354
	332
	353
	376

Figure 6g **Evaluation of uCT images: BV/TV (%)**

hTNFtg	hTNFtg/*Lasp1* $^{-/-}$
8,3	11,4
11,6	17,2
11,6	14,2
9,2	12,2
5,6	14,5
12,2	11
8,7	12,6
9,9	10,65
7,12	10,7
7,019	11,75

Figure 6h **hTNF ELISA (pg/ml)**

hTNFtg	hTNFtg/*Lasp1* $^{-/-}$
281,99	412,5
566,03	531,92
697,91	575,43
637,8	617,01
477,01	435,76
391,42	375,26

Figure 6i Immunoblotting for Lasp1 expression in osteoblasts

Lasp1 expression

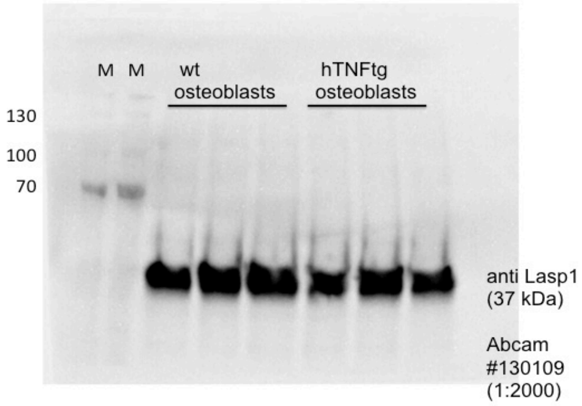

M = marker

04.10.19

GAPDH expression

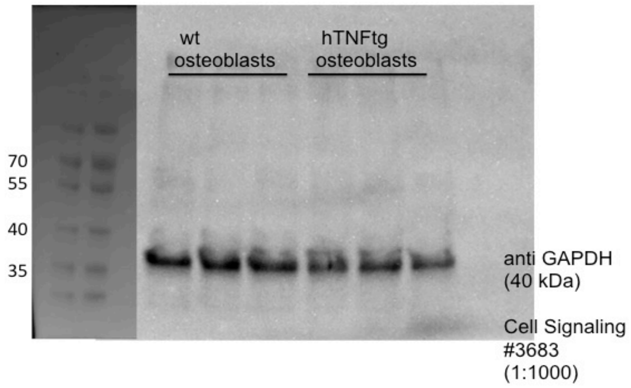

06.10.19

Figure 7a **Cell number pannus tissue per HPF (10.000µm2)**

hTNFtg	hTNFtg/Lasp1-/-
173	58
101	56
157	69
186	51
176	78
106	73
106	60
143	92
149	74
173	75
101	95
157	81
186	58
176	56
106	69
106	51
143	78
149	73
160	60
112	92
103	74
307	75
164	95
128	81
109	65
146	47
189	60
165	237
258	205
138	162
220	62
240	77
151	101
207	88
140	99
213	110
265	134
242	86
173	118
	115
	143
	94

Figure 7c **Immunoblotting for Src and AKT expression after PDGF stimulation**

phospho-src/src ratio

	hTNFtg			hTNFtg/*Lasp1-/-*		
min	Sample 1	Sample 2	Sample 3	Sample 1	Sample 2	Sample 3
10	6,10825243	10,424	4,27635328	2,87529976	2,53198653	3,05576208
15	8,72424242	5,50177936	7,16414687	3,09241706	2,77322404	3,35452794
15	6,26027397	11,6134752	16,0093897	2,81967213	3,07122507	4,99378882

phospho-AKT/AKT ratio

	hTNFtg			hTNFtg/*Lasp1-/-*		
min	Sample 1	Sample 2	Sample 3	Sample 1	Sample 2	Sample 3
10	1,24281609	2,57882353	0,9640914	0,47757848	0,37469586	0,17068063
15	5,03695652	6,15890411	2,44135429	1,1178344	0,65616798	0,40148699
30	4,68870968	5,39565217	3,5511811	2,93019802	2,12530121	1,0748156

Immunoblotting for Src and AKT expression after PDGF stimulation

pSrc expression PDGF stimulation

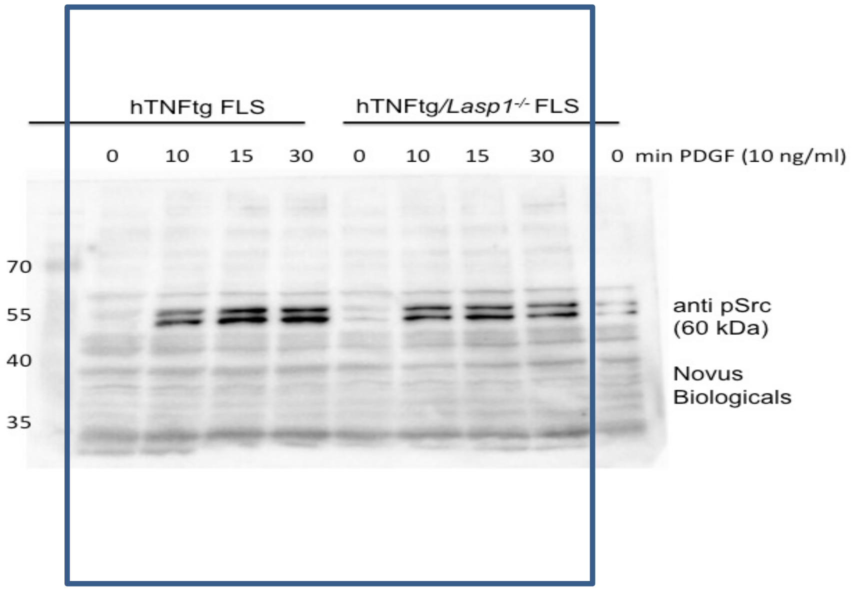

04.05.16

Src expression PDGF stimulation

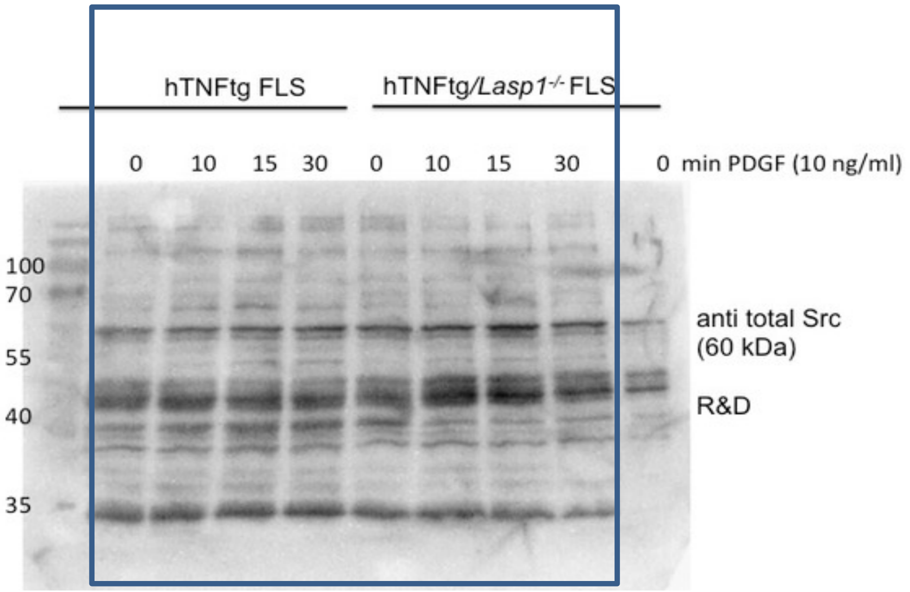

09.05.16

pAKT expression PDGF stimulation

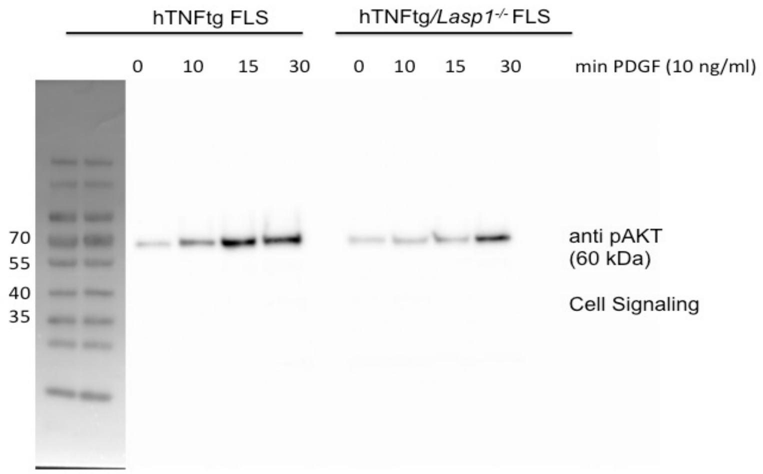

14.07.17

AKT expression PDGF stimulation

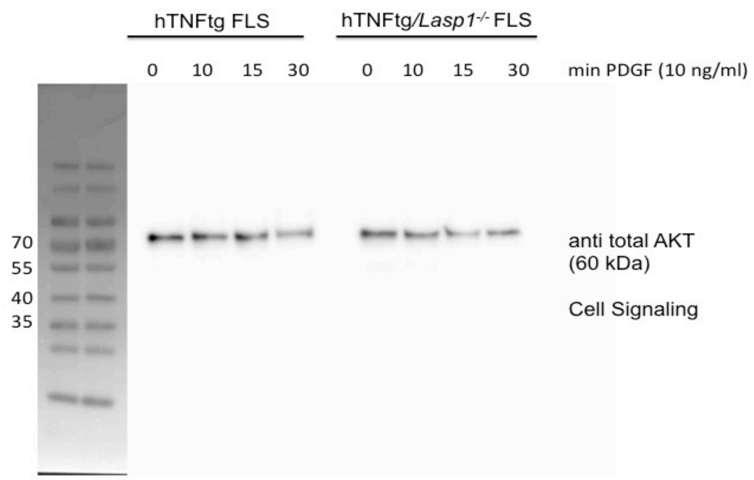

18.07.17

Supplementary Figure 5f

IHC evaluation OA vs. RA (rel. intensity)

OA	RA
0,143	0,201
0,127	0,185
0,137	0,193
0,021	0,193
0,045	0,207
0,045	0,217
0,068	0,173
0,079	0,173
0,075	0,146

Supplementary Figure 6a   ECIS: murine cells, 1st run (resistance - ohm)

time	hTNFtg FLS	hTNFtg/*Lasp1* $^{-/-}$ FLS
0,0036	9514	12323
0,0036	9514	12323
0,0783	11048	13151
0,1532	12641	14275
0,2281	13907	15703
0,3029	14615	16590
0,3776	14972	17198
0,4524	15301	17332
0,5271	15754	17427
0,602	15988	17524
0,6769	15520	17727
0,7516	15411	17774
0,8264	15488	17758
0,9012	15421	17623
0,976	15447	17567
1,0506	15291	17607
1,1253	15363	17898
1,2	15144	17988
1,2746	15069	18259
1,3493	15045	18208
1,424	15298	17992
1,4987	15355	18022
1,5733	15469	17958
1,648	15478	17717
1,7227	15725	17520
1,7974	15462	17487
1,8721	15430	17068
1,9468	15475	16934
2,0214	15442	17039
2,0961	15393	16932
2,1708	15219	16595
2,2454	14955	16637
2,3201	14646	16327
2,3948	14597	16216
2,4694	14629	15877
2,5441	14619	15462
2,619	14339	15498
2,6936	14251	15569
2,7683	13956	15509
2,843	13952	15306
2,9176	13886	15157
2,9923	13741	15013
3,067	13556	14960
3,1416	13627	14578
3,2165	13357	14399
3,2912	13293	14314
3,366	13310	14422

3,4407	13329	14184
3,5156	13308	14156
3,5902	13391	14177
3,6649	13190	14087
3,7395	13118	14110
3,8142	13064	13933
3,8891	13050	13784
3,9637	12999	13995
4,0384	12949	14004
4,113	12973	13756
4,1877	12955	13699
4,2623	12995	13432
4,337	13023	13475
4,4119	13191	13256
4,4867	13262	13464
4,5616	13337	13399
4,6364	13506	13393
4,7113	13500	13076
4,7863	13479	13229
4,861	13452	13128
4,9358	13269	13174
5,0106	13244	13122
5,0855	13436	13067
5,1603	13392	13133
5,2352	13402	13049
5,3098	13426	13060
5,3847	13367	12778
5,4595	13460	12685
5,5344	13488	12704
5,6092	13225	12706
5,6839	13126	12818
5,7585	13045	12693
5,8332	13276	12523
5,9081	13321	12569
5,983	13057	12461
6,0579	12939	12649
6,1327	12860	12537
6,2076	12913	12548
6,2825	12766	12630
6,3575	12837	12771
6,4324	12677	12664
6,5074	12498	12656
6,5823	12590	12886
6,6572	12485	12707
6,732	12372	12530
6,8069	12299	12513
6,8817	12105	12386
6,9566	12106	12428
7,0315	12064	12235
7,1065	11943	12237
7,1814	11878	12371
7,2564	11996	12435

7,3315	12155	12359
7,4065	12108	12317
7,4816	12015	12279
7,5565	11899	12282
7,6314	11862	12354
7,7063	11762	12175
7,7812	11640	12081
7,856	11810	12042
7,9309	12048	12081
8,0058	12027	11989
8,0807	12077	12052
8,1555	12039	12043
8,2304	11900	11919
8,3054	11751	11853
8,3803	11784	11822
8,4554	11617	12047
8,5302	11496	11879
8,6053	11379	12052
8,6803	11413	12165
8,7554	11208	12159
8,8304	11136	12249
8,9055	11073	12175
8,9805	11074	12097
9,0556	10982	12049
9,1306	11036	12080
9,2055	11064	12046
9,2804	11154	12209
9,3553	11193	12060
9,4302	11135	12038
9,5051	11174	12028
9,58	11165	12066
9,6548	11299	11872
9,7297	11275	11927
9,8046	11398	11855
9,8795	11292	11820
9,9543	11116	11966
10,0294	10941	12085
10,1042	10905	12272
10,1793	10965	12205
10,2541	11045	12383
10,329	10896	12340
10,4039	10833	12186
10,4787	10891	12242
10,5536	10789	12108
10,6286	10800	11959
10,7037	10813	12084
10,7787	10803	12170
10,8536	10827	12199
10,9286	10804	12316
11,0035	10737	12353
11,0784	10673	12292
11,1533	10671	12338

11,2282	10603	12399
11,303	10445	12489
11,3781	10366	12534
11,453	10368	12428
11,5279	10209	12511
11,6027	10180	12379
11,6776	10235	12323
11,7525	10095	12186
11,8273	10090	12173
11,9022	10172	12133
11,9773	10223	12176
12,0523	10273	12182
12,1274	10309	12144
12,2024	10345	12227
12,2775	10346	12234
12,3525	10492	12319
12,4274	10389	12355
12,5024	10287	12321
12,5773	10171	12297
12,6524	10295	12305
12,7272	10336	12277
12,8021	10406	12404
12,877	10478	12454
12,9519	10520	12294
13,0268	10522	12338
13,1017	10640	12332
13,1767	10676	12325
13,2516	10643	12603
13,3265	10510	12641
13,4013	10500	12769
13,4764	10583	12546
13,5514	10524	12552
13,6263	10513	12528
13,7013	10521	12663
13,7762	10428	12742
13,851	10409	12672
13,9259	10400	12717
14,001	9781	12466
14,0759	9855	12384
14,1509	9906	12284
14,2258	9910	12348
14,3008	10007	12421
14,3757	9972	12463
14,4506	10008	12481
14,5254	10015	12472
14,6003	10001	12392
14,6752	10118	12427
14,7501	10092	12436
14,825	10224	12440
14,8998	10254	12445
14,9747	10283	12568
15,0498	10271	12655

15,1246	10253	12588
15,1995	10399	12337
15,2743	10392	12353
15,3492	10391	12310
15,4241	10427	12300
15,4989	10372	12254
15,5738	10378	12289
15,6489	10286	12361
15,7237	10325	12566
15,7988	10296	12500
15,8736	10270	12502
15,9487	10237	12321
16,0237	10170	12282
16,0986	10170	12410
16,1736	10177	12599
16,2485	10184	12814
16,3234	10144	12779
16,3982	10188	12708
16,4731	10228	13022
16,548	10235	12920
16,6229	10236	13038
16,6978	10258	13240
16,7726	10189	13197
16,8475	10057	13208
16,9224	10029	13372
16,9974	10118	13242
17,0723	10213	13337
17,1474	10143	13278
17,2224	10081	13315
17,2974	10230	13319
17,3725	10282	13301
17,4475	10409	13200
17,5226	10292	13247
17,5974	10246	13368
17,6725	10059	13312
17,7475	10146	13312
17,8225	10108	13408
17,8974	10187	13385
17,9723	10212	13505
18,0472	10187	13322
18,1221	10250	13389
18,197	10282	13308
18,2718	10325	13278
18,3467	10281	13084
18,4216	10369	12982
18,4964	10465	12978
18,5713	10498	12922
18,6462	10416	12824
18,7212	10396	12818
18,7961	10310	12682
18,8709	10543	12712
18,9458	10507	12861

19,0207	10483	12831
19,0955	10276	12824
19,1704	10247	12656
19,2453	10243	12714
19,3201	10321	12728
19,395	10296	12697
19,47	10288	12589
19,5451	10163	12622
19,6201	10210	12534
19,695	10326	12411
19,7698	10558	12511
19,8447	10861	12580
19,9196	11042	12600
19,9944	10792	12488
20,0693	10623	12466
20,1442	10493	12475
20,2191	10510	12375
20,294	10556	12440
20,3688	10566	12351
20,4437	10426	12277
20,5188	10430	12203
20,5936	10420	12114
20,6687	10403	12091
20,7435	10413	12013
20,8185	10398	12024
20,8936	10444	11981
20,9686	10527	11984
21,0437	10373	12021
21,1187	10350	11964
21,1938	10490	11978
21,2686	10451	11876
21,3437	10443	11978
21,4187	10487	12084
21,4937	10436	12165
21,5686	10396	12143
21,6435	10300	12334
21,7184	10261	12304
21,7933	10284	12281
21,8681	10313	12257
21,943	10218	12318
22,0179	10277	12358
22,0927	10297	12325
22,1676	10238	12342
22,2425	10274	12422
22,3173	10183	12438
22,3924	10176	12496
22,4672	10115	12402
22,5421	10141	12426
22,6169	10091	12538
22,6918	10165	12531
22,7667	10249	12586
22,8415	10249	12506

22,9164	10141	12652
22,9913	10168	12714
23,0663	10231	12586
23,1413	10300	12403
23,2161	10432	12366
23,2909	10515	12195
23,3658	10808	12149
23,4404	10685	12270
23,5153	10679	12438
23,5901	10609	12377
23,6649	10528	12421
23,7396	10586	12498
23,8142	10590	12585
23,8889	10498	12716
23,9636	10509	12745
24,0383	10572	12889
24,113	10546	13006
24,1877	10455	13023
24,2624	10506	13110
24,337	10522	12971
24,4117	10499	13015
24,4864	10512	13174
24,5611	10446	13078
24,6358	10363	12975
24,7105	10308	13069
24,7852	10359	12935

Supplementary Figure 6b ECIS: murine cells, 2nd run (resistance - ohm)

time	hTNFtg FLS	hTNFtg/*Lasp1* $^{-/-}$ FLS
0,0036	11622	10924,5
0,0036	11622	10924,5
0,0783	11372,5	10674,5
0,1532	11209,5	11256
0,2281	11158,5	12125
0,3029	11303,5	12801,5
0,3776	11519	13707,5
0,4524	11647,5	14875,5
0,5271	11724,5	16140
0,602	11928	17059
0,6769	12158,5	17942,5
0,7516	12608,5	18728,5
0,8264	13086,5	19146
0,9012	13414	19672,5
0,976	14175	20127
1,0506	14840,5	20983
1,1253	15377,5	21700
1,2	15566	21565
1,2746	15880	21642
1,3493	16160,5	21463,5
1,424	16504,5	21462,5
1,4987	16390	20773,5
1,5733	16357	20565
1,648	16509,5	20545,5
1,7227	16697,5	20322
1,7974	16669	20040
1,8721	17154,5	19659,5
1,9468	16930	19658,5
2,0214	17143,5	19532
2,0961	17076,5	19468
2,1708	16885,5	19440,5
2,2454	16748	19123,5
2,3201	16819,5	19376
2,3948	16550	18913
2,4694	16292,5	18304
2,5441	16056,5	17978,5
2,619	15686	18029
2,6936	15692	18056
2,7683	15132,5	17787,5
2,843	14860,5	17669,5
2,9176	14845	17405,5
2,9923	14825,5	17437
3,067	14613	17595
3,1416	14544,5	17567
3,2165	14668,5	17605
3,2912	14531	17434,5
3,366	14369	17290

3,4407	14200,5	17179,5
3,5156	13979	17193
3,5902	13835	17162
3,6649	13388	17178
3,7395	13284,5	17275,5
3,8142	13318	17355
3,8891	13154,5	17125,5
3,9637	12997	17252,5
4,0384	13293,5	17273
4,113	13274,5	17352
4,1877	13397	17366
4,2623	13365,5	17412,5
4,337	13301	17408
4,4119	13209	17337,5
4,4867	13048,5	17234,5
4,5616	12792,5	17465
4,6364	12522,5	17204
4,7113	12324	17071
4,7863	12615	17033
4,861	12559,5	17142,5
4,9358	12682	17154
5,0106	12749	17107,5
5,0855	12689,5	16969
5,1603	12631,5	17073
5,2352	12445,5	17079
5,3098	12192,5	17377
5,3847	11973	17492
5,4595	11854,5	17609
5,5344	11855,5	17633
5,6092	11801,5	17433,5
5,6839	11709	17504,5
5,7585	11673	17716
5,8332	11621,5	17666,5
5,9081	11723,5	17606,5
5,983	11773,5	17535
6,0579	11641,5	17421,5
6,1327	11614	17155
6,2076	11538	17176
6,2825	11474,5	17471,5
6,3575	11403	17390,5
6,4324	11204	17383,5
6,5074	11241,5	17347
6,5823	10859	17405
6,6572	10627	17415,5
6,732	10422,5	17513
6,8069	10144,5	17502,5
6,8817	10279	17190,5
6,9566	10115,5	17212
7,0315	10228,5	17126
7,1065	10158,5	17269
7,1814	10233,5	17254,5
7,2564	10293	17030,5

7,3315	10450	17174,5
7,4065	10405,5	17041,5
7,4816	10496	17043,5
7,5565	10495,5	16909
7,6314	10512	16777,5
7,7063	10546	16944,5
7,7812	10558,5	17133,5
7,856	10393	17237,5
7,9309	10360,5	17150,5
8,0058	10466,5	17071,5
8,0807	10493	17186,5
8,1555	10631	17181,5
8,2304	10648	16791
8,3054	10692,5	16539
8,3803	10562,5	16514,5
8,4554	10482,5	16472,5
8,5302	10518	16377,5
8,6053	10538	16459
8,6803	10790	16262,5
8,7554	10803,5	16295
8,8304	10826,5	16342
8,9055	10733,5	16367
8,9805	10499,5	16419
9,0556	10229,5	16431
9,1306	10102,5	16663
9,2055	10187,5	16693
9,2804	10275,5	16804,5
9,3553	10299,5	16658,5
9,4302	10326,5	16593
9,5051	10276	16727
9,58	10450,5	16667,5
9,6548	10440	16645,5
9,7297	10594,5	16885,5
9,8046	10331	16753
9,8795	10250,5	16696
9,9543	10044,5	16710,5
10,0294	10264	16612,5
10,1042	10236,5	16620,5
10,1793	10170,5	16765
10,2541	10049	16893,5
10,329	10128	16961,5
10,4039	10272,5	17198
10,4787	10348	17217
10,5536	10248,5	17284,5
10,6286	10272	17184
10,7037	10273,5	17428
10,7787	10489,5	17504
10,8536	10264,5	17595
10,9286	10360,5	17608
11,0035	10282,5	17579,5
11,0784	10295,5	17473
11,1533	10189,5	17422,5

11,2282	10169	17405
11,303	10125	17440
11,3781	10256	17226,5
11,453	10321	17322,5
11,5279	10281	17320
11,6027	10317,5	17394
11,6776	10415,5	17269
11,7525	10428,5	17211
11,8273	10403,5	17273
11,9022	10402,5	17342
11,9773	10489,5	17289,5
12,0523	10586,5	17104
12,1274	10573,5	17080
12,2024	10611	17227
12,2775	10540,5	17295
12,3525	10496	17224
12,4274	10502	17316,5
12,5024	10589	17572
12,5773	10588	17641,5
12,6524	10613	17669,5
12,7272	10796	17695
12,8021	10886	17675,5
12,877	10820,5	17636
12,9519	10845,5	17595,5
13,0268	10778,5	17719
13,1017	10763	17590,5
13,1767	10915	17662,5
13,2516	11021,5	17770
13,3265	10868	17565,5
13,4013	10787,5	17413
13,4764	10822	17225,5
13,5514	10803,5	17208,5
13,6263	10838	16925,5
13,7013	10823	17013,5
13,7762	10829	16888,5
13,851	10964,5	17090
13,9259	11067,5	17174,5
14,001	11009,5	17154,5
14,0759	11117	17382
14,1509	11155,5	17306,5
14,2258	11227	17401
14,3008	11349	17438,5
14,3757	11216	17367,5
14,4506	11323,5	17303,5
14,5254	11340,5	17353
14,6003	11512,5	17368
14,6752	11528	17250
14,7501	11597,5	17137
14,825	11628,5	17154,5
14,8998	11538	16834,5
14,9747	11644,5	16985
15,0498	11615	16986,5

15,1246	11661	17076
15,1995	11878	17032
15,2743	11843	17016,5
15,3492	11856	17089,5
15,4241	11800,5	17123,5
15,4989	11780	17127,5
15,5738	11915	17038
15,6489	11791,5	16861
15,7237	12015	16822,5
15,7988	11977	16621,5
15,8736	11961	16605,5
15,9487	12248,5	16603
16,0237	12229	16644,5
16,0986	12397,5	16516,5
16,1736	12350,5	16331,5
16,2485	12388	16237,5
16,3234	12368	16185
16,3982	12374	16281,5
16,4731	12319,5	16503,5
16,548	12314	16382
16,6229	12307	16403,5
16,6978	12311	16467
16,7726	12292	16707
16,8475	12198	16907,5
16,9224	12481	16870,5
16,9974	12698,5	16745,5
17,0723	12677	16769
17,1474	12595,5	16818
17,2224	12697	16903
17,2974	12816	16892,5
17,3725	12709,5	16916,5
17,4475	12678,5	16888
17,5226	12732	16882,5
17,5974	12740	16790
17,6725	12826,5	16945,5
17,7475	12675	16962,5
17,8225	12747,5	17155
17,8974	12953,5	17105
17,9723	12824,5	17147,5
18,0472	12838	17052
18,1221	12655	17105,5
18,197	12614	17061,5
18,2718	12611	16972,5
18,3467	12577	16939,5
18,4216	12539,5	16938
18,4964	12646,5	16755
18,5713	12616,5	16736,5
18,6462	12686	16722
18,7212	12682	16659,5
18,7961	12591,5	16534
18,8709	12725,5	16747
18,9458	12572	16445,5

19,0207	12463,5	16334
19,0955	12374,5	16203,5
19,1704	12304	16303
19,2453	12462,5	16377
19,3201	12430,5	16575
19,395	12434,5	16580,5
19,47	12482,5	16405,5
19,5451	12474,5	16511,5
19,6201	12437	16417
19,695	12355	16543,5
19,7698	12561	16469
19,8447	12571	16425,5
19,9196	12635	16237
19,9944	12766,5	16261
20,0693	12872	16176,5
20,1442	12815	16185,5
20,2191	12680	16215,5
20,294	12775,5	16205,5
20,3688	12574,5	16122
20,4437	12643,5	16184,5
20,5188	12670,5	16181,5
20,5936	12717	16050
20,6687	12878	16075
20,7435	12819	16123
20,8185	13116	15937,5
20,8936	13109	15919
20,9686	13303,5	16189,5
21,0437	13318,5	16176,5
21,1187	13314	16169
21,1938	13429	16255
21,2686	13629,5	16249,5
21,3437	13641	16154
21,4187	13634	16065,5
21,4937	13701	16257
21,5686	13716	16304
21,6435	13902,5	16258
21,7184	13749,5	16274
21,7933	13783,5	16334,5
21,8681	13804	16433
21,943	13821	16351,5
22,0179	13815	16361,5
22,0927	13917	16363,5
22,1676	13960	16371,5
22,2425	13694,5	16260,5
22,3173	13537,5	16274
22,3924	13802,5	16312
22,4672	14398	16219
22,5421	14338	16052
22,6169	14374,5	16147,5
22,6918	14527	16121,5
22,7667	14483	15961
22,8415	14853,5	16076

22,9164	14923,5	16050,5
22,9913	15049	16119
23,0663	14962,5	16195,5
23,1413	14704	16290,5
23,2161	14867	16350
23,2909	14745	16077,5
23,3658	14356	16034
23,4404	14407,5	16000,5
23,5153	14394	15932,5
23,5901	14389	15887
23,6649	14437,5	15752,5
23,7396	14428	15776
23,8142	14665	15891
23,8889	14513	15985,5
23,9636	14542,5	16069
24,0383	14595	16122,5
24,113	14626	16270,5
24,1877	14754	16258,5
24,2624	14745,5	16392
24,337	14733,5	16204
24,4117	14574,5	16353,5
24,4864	14601	16183,5
24,5611	14669	16057,5
24,6358	14710	16327
24,7105	14451,5	16270
24,7852	14362	16366

Supplementary Figure 6c **ECIS: murine cells, 3rd run (resistance - ohm)**

time	hTNFtg FLS	hTNFtg/*Lasp1* $^{-/-}$ FLS
0,0036	10897,5	11522,5
0,0036	10897,5	11522,5
0,0783	10524,5	11240,5
0,1532	10280,5	11441,5
0,2281	10101,5	12137
0,3029	10118,5	13144,5
0,3776	10303	14105,5
0,4524	10793	15009
0,5271	10996	15698,5
0,602	11396	16428
0,6769	11769,5	17015
0,7516	12054,5	17545,5
0,8264	12405,5	17874,5
0,9012	13027	17974,5
0,976	13734	18447,5
1,0506	14353,5	19073
1,1253	14865	19531,5
1,2	15276	19754
1,2746	15604,5	19657
1,3493	16022,5	19900,5
1,424	16185	20183,5
1,4987	16447,5	20316
1,5733	16589	20468,5
1,648	16939,5	20615,5
1,7227	16736	20802
1,7974	16585	20707
1,8721	16788,5	20328
1,9468	16830,5	20100,5
2,0214	16914	20015,5
2,0961	16671	20006
2,1708	16698	19796,5
2,2454	16877	19795
2,3201	17086	19850
2,3948	16984	19750,5
2,4694	17405,5	19810,5
2,5441	17384	19396,5
2,619	17237	19538,5
2,6936	17396	19105,5
2,7683	17104,5	19089
2,843	16792	18952
2,9176	16741,5	18763
2,9923	16873	18262,5
3,067	16536	18192,5
3,1416	16508,5	18054,5
3,2165	16535,5	17850
3,2912	16566	17714,5
3,366	16708,5	17744,5

3,4407	16441,5	17318,5
3,5156	16323,5	17163,5
3,5902	16328	17074
3,6649	16085	16558,5
3,7395	16193	16393
3,8142	16124	16416,5
3,8891	16057	15970
3,9637	16015,5	15781,5
4,0384	15608,5	15768,5
4,113	15724,5	15373
4,1877	15556	15053
4,2623	15779	14840
4,337	15217,5	14559
4,4119	15026	14484,5
4,4867	14587,5	14478,5
4,5616	14456	14414,5
4,6364	14418	14369
4,7113	14285	14346
4,7863	14387,5	14228
4,861	13999	14136
4,9358	13738,5	13973,5
5,0106	13621,5	14220,5
5,0855	13489	14351
5,1603	13281	14655,5
5,2352	13214,5	14507
5,3098	13427	14335
5,3847	13469,5	14550,5
5,4595	13187,5	14544
5,5344	13335	14183
5,6092	13139,5	13539
5,6839	12879,5	13639,5
5,7585	12768,5	13693
5,8332	12865,5	13672,5
5,9081	12552	13683,5
5,983	12113,5	13550,5
6,0579	12009,5	13405
6,1327	12058	13594,5
6,2076	11988,5	13362,5
6,2825	11888,5	13502,5
6,3575	11907	12983,5
6,4324	11934,5	13022,5
6,5074	11887,5	13079,5
6,5823	11749,5	13198
6,6572	11637,5	13173,5
6,732	11587,5	12926,5
6,8069	11506	12985,5
6,8817	11505	12986
6,9566	11443,5	12900
7,0315	11412,5	12733
7,1065	11488	12568,5
7,1814	11377	12448
7,2564	11367,5	12521

7,3315	11404,5	12640
7,4065	11458	12723
7,4816	11672	12772
7,5565	11507	12943,5
7,6314	11500	13324
7,7063	11583,5	13545,5
7,7812	11739	13270
7,856	11611,5	12949,5
7,9309	11776,5	12708
8,0058	11536	12454
8,0807	11642,5	12281,5
8,1555	11674	12193
8,2304	11696	12191,5
8,3054	11640,5	12125,5
8,3803	11566	12080
8,4554	11397,5	12319,5
8,5302	11445	12335
8,6053	11487,5	12318
8,6803	11443	12360,5
8,7554	11418	12607
8,8304	11408	12631,5
8,9055	11502	12603,5
8,9805	11402	12687
9,0556	11389,5	12726,5
9,1306	11317,5	12693,5
9,2055	11336,5	12839
9,2804	11397	12924
9,3553	11465,5	13160,5
9,4302	11520	13108
9,5051	11479	12763
9,58	11589,5	12717
9,6548	11345	12699,5
9,7297	11484,5	12706,5
9,8046	11597,5	12739
9,8795	11497	12593
9,9543	11626	12824,5
10,0294	11640,5	12800
10,1042	11745,5	12898
10,1793	11894	12796
10,2541	11776	12649
10,329	11623	12361
10,4039	11559,5	12294
10,4787	11629	12334
10,5536	11475,5	12356
10,6286	11755,5	12283
10,7037	11545,5	12011,5
10,7787	11795,5	11876,5
10,8536	11831	11904
10,9286	11701	11926,5
11,0035	11672,5	11698
11,0784	11873,5	11904,5
11,1533	11948	11991,5

11,2282	11774,5	11982,5
11,303	11888	11879,5
11,3781	11640,5	12053
11,453	11516,5	12016
11,5279	11557,5	12167
11,6027	11493	12112,5
11,6776	11527	12246
11,7525	11473,5	12375
11,8273	11410	12443
11,9022	11425	12390
11,9773	11456,5	12601,5
12,0523	11562	12549,5
12,1274	11513,5	12629
12,2024	11623,5	12754
12,2775	11629	12650
12,3525	11821	12809
12,4274	11991,5	12781,5
12,5024	12145,5	12763
12,5773	12007,5	12794
12,6524	11886,5	12576
12,7272	12063,5	12526,5
12,8021	11849,5	12324
12,877	11787	12466,5
12,9519	11420,5	12409
13,0268	11594	12487
13,1017	11693,5	12403,5
13,1767	11490,5	12477
13,2516	11587,5	12553,5
13,3265	11797	12676,5
13,4013	11823	12560
13,4764	11805,5	12470,5
13,5514	11945,5	12607,5
13,6263	11937,5	12422
13,7013	11831	12480,5
13,7762	11767	12517,5
13,851	11644,5	12434,5
13,9259	11801,5	12418,5
14,001	12068,5	12647,5
14,0759	11879	12686
14,1509	11744,5	12634
14,2258	11644	12937,5
14,3008	11532,5	13057,5
14,3757	11581,5	13226
14,4506	11485	13247
14,5254	11608,5	13128,5
14,6003	11642,5	13150
14,6752	11484	13381,5
14,7501	11447	13508,5
14,825	11601	13371,5
14,8998	11652,5	13389
14,9747	11728	13224
15,0498	11672,5	13361,5

15,1246	11845,5	12881,5
15,1995	11733,5	12801
15,2743	11814,5	12778
15,3492	11895	12861
15,4241	12001	12891,5
15,4989	12121,5	12902
15,5738	11809,5	12966,5
15,6489	11555	12746,5
15,7237	11330,5	12730,5
15,7988	11431	12714
15,8736	11514,5	12553
15,9487	11621	12387,5
16,0237	11390	12336
16,0986	11479,5	12462,5
16,1736	11442,5	12400
16,2485	11413,5	12300
16,3234	11329,5	12064,5
16,3982	11275,5	12071
16,4731	11258,5	11982
16,548	11334	12025,5
16,6229	11640	11895,5
16,6978	11565	11998,5
16,7726	11526,5	12115,5
16,8475	11634	12202
16,9224	11620	12199,5
16,9974	11400	12242,5
17,0723	11602,5	12346,5
17,1474	11625	12119
17,2224	11653	12280,5
17,2974	12021,5	12240,5
17,3725	11879,5	12018
17,4475	11366	11977
17,5226	11242,5	11917
17,5974	11453	11965,5
17,6725	11627	11907,5
17,7475	11653,5	11997
17,8225	11699	12030
17,8974	11591	11961
17,9723	11612,5	12080,5
18,0472	11715,5	12126
18,1221	11822	12180,5
18,197	11754,5	12201,5
18,2718	11709,5	12256,5
18,3467	11594,5	12162
18,4216	11515	12244,5
18,4964	11447	12228
18,5713	11426	12219
18,6462	11490	12291
18,7212	11454,5	12289,5
18,7961	11246,5	12426,5
18,8709	11117	12324,5
18,9458	10986	12261

19,0207	10979,5	12179
19,0955	10895	12167
19,1704	10931,5	12268,5
19,2453	10919	12365,5
19,3201	11127	12377
19,395	10953	12445
19,47	11052	12362
19,5451	11141,5	12655,5
19,6201	11174,5	12724
19,695	10917	12630,5
19,7698	10893,5	12625
19,8447	10795,5	12813
19,9196	10800,5	12961,5
19,9944	10735	13132,5
20,0693	10640,5	13168,5
20,1442	10945	13068,5
20,2191	10952	13202
20,294	11028	13154
20,3688	10936	13410
20,4437	10964,5	13310,5
20,5188	10853,5	13286,5
20,5936	10629	13434
20,6687	10682,5	13607
20,7435	10745	13604
20,8185	10816,5	13710,5
20,8936	10722,5	13670
20,9686	10735	13636
21,0437	10827,5	13451
21,1187	10580,5	13507,5
21,1938	10292,5	13235
21,2686	10485	13274
21,3437	10494,5	13145,5
21,4187	10711,5	13098
21,4937	10725,5	12894,5
21,5686	10694	12755
21,6435	10870	12654
21,7184	10753	12584,5
21,7933	10862,5	12478
21,8681	10763	12332
21,943	10912	12405,5
22,0179	10866	12285
22,0927	11065	12288
22,1676	10896	12241,5
22,2425	10887	12130
22,3173	10475	12070
22,3924	10679,5	12214,5
22,4672	10739,5	12451,5
22,5421	10896,5	12712,5
22,6169	11234	12762
22,6918	11209	12654
22,7667	11249	12531
22,8415	11547,5	12537,5

22,9164	11570,5	12546,5
22,9913	11412	12654
23,0663	11467	12631
23,1413	11238	12762
23,2161	11029,5	12664,5
23,2909	10977,5	12660
23,3658	11108	12579
23,4404	11069,5	12410,5
23,5153	11135,5	12449
23,5901	11129,5	12729,5
23,6649	11177	12726,5
23,7396	11309,5	12812,5
23,8142	11372,5	12850,5
23,8889	11379	12984
23,9636	11434	13051,5
24,0383	11632,5	12837,5
24,113	11454	12830
24,1877	11210	12712,5
24,2624	11307	12471
24,337	11097	12670
24,4117	11204	12863,5
24,4864	11254,5	12854
24,5611	11338	12657
24,6358	11259,5	12658,5
24,7105	11530	12949,5
24,7852	11574,5	13054,5

Supplementary Figure 6d **ECIS: Comparison hTNFtg/Lasp1-/- FLS vs Colon-2 (Caco-2) cells**

time	*hTNFtg/Lasp1-/- FLS*	Caco-2
0,0036	15733	12502,5
0,0036	15733	12502,5
0,1528	17575	12610
0,302	19498	12651,5
0,4511	20695	12687
0,6	20886	12704
0,7491	20782	12705
0,8982	20551	12699
1,0479	20747	12676,5
1,197	20746	12635,5
1,3462	21131	12607,5
1,4953	21115	12571
1,6444	21109	12546,5
1,7936	20724	12515
1,9427	20206	12488,5
2,0918	19740	12461
2,2409	19583	12397
2,3901	19098	12338
2,5392	18900	12279,5
2,6884	18820	12234,5
2,8373	18519	12183,5
2,9863	18018	12137,5
3,1352	17742	12119,5
3,2844	17257	12107,5
3,4335	16830	12102,5
3,5824	16258	12103,5
3,7315	16164	12117,5
3,8807	15663	12110,5
4,0298	15473	12074
4,1789	15268	12019,5
4,3278	14944	11976,5
4,477	14772	11947
4,6261	14558	11938
4,7752	14217	11920
4,9244	14000	11941,5
5,0734	14011	11923,5
5,2225	13767	11886,5
5,3714	13751	11873
5,5205	13588	11834,5
5,6697	13351	11826
5,8188	13260	11845
5,9679	13184	11830,5
6,1171	13183	11791
6,2662	13093	11810
6,4154	13061	11764
6,5645	13033	11792
6,7136	12942	11795,5

6,8627	13315	11792
7,0117	13171	11774,5
7,1608	13252	11771,5
7,3099	13480	11760
7,4591	13226	11749
7,6082	12865	11685
7,7572	12887	11650,5
7,9063	12669	11601
8,0555	12726	11565,5
8,2046	12944	11572,5
8,3537	12704	11493,5
8,5028	12848	11244
8,652	12678	11227
8,8011	12717	11188
8,9502	12723	11209,5
9,0993	12590	11251,5
9,2485	12539	11292
9,3976	12596	11287,5
9,5467	12598	11200
9,6958	12527	11316
9,8448	12554	11305,5
9,9939	12604	11248
10,1429	12529	11134
10,292	12383	11030,5
10,4412	12283	10979,5
10,5903	12244	10928
10,7394	12091	10871,5
10,8886	12048	10837,5
11,0377	11910	10833,5
11,1868	11670	10798,5
11,3357	11825	10754,5
11,4849	11870	10667
11,634	12045	10572
11,7831	12098	10630
11,9322	11976	10492,5
12,0812	11918	10524
12,2303	11938	10298
12,3794	11832	9920,5
12,5284	11732	9770
12,6775	11634	9713
12,8266	11650	9712,5
12,9757	11556	9821
13,1249	11499	9885,5
13,274	11623	9937
13,4232	11515	9976
13,5723	11583	10077
13,7214	11467	10129,5
13,8706	11679	10215,5
14,0195	11644	10176,5
14,1686	11783	10155,5
14,3176	11733	10187,5
14,4667	11756	10312,5

14,6159	11792	10351,5
14,765	11794	10434
14,914	11677	10446,5
15,0631	11490	10471
15,2123	11481	10414,5
15,3614	11447	10321,5
15,5106	11455	10264,5
15,6597	11289	10331,5
15,8089	11260	10432
15,958	11068	10516
16,107	11140	10725
16,2561	11148	10822
16,4052	11076	11001,5
16,5544	11079	11081,5
16,7035	11104	11152
16,8524	11142	11190
17,0016	11141	11322
17,1507	11088	11428
17,2997	11057	11395
17,4486	10993	11326
17,5977	10933	11363,5
17,7469	10823	11550,5
17,896	10809	11667,5
18,0451	10742	11654
18,1941	10813	11590
18,3432	10901	11543,5
18,4923	10985	11505,5
18,6415	11018	11609
18,7906	11094	11798
18,9398	11055	11908,5
19,0887	11042	11800
19,2379	10947	11865
19,387	10983	12134,5
19,536	11059	12247
19,6849	11071	12114
19,834	11043	12298
19,9832	11061	12375
20,1323	11227	12492,5
20,2814	11340	12678
20,4305	11431	12844
20,5797	11436	12881,5
20,7288	11536	13223,5
20,8779	11560	13245,5
21,0269	11544	13338,5
21,176	11535	13469
21,3251	11528	13692
21,4743	11523	14195
21,6234	11526	14343,5
21,7726	11451	14647
21,9217	11401	14567,5
22,0706	11360	14983
22,2198	11393	15150

22,3689	11307	15314,5
22,518	11311	15501,5
22,6671	11444	15647
22,8163	11473	15916
22,9654	11383	16163
23,1146	11294	16009,5
23,2637	11218	15955,5
23,4128	11272	16232,5
23,5619	11257	16589
23,711	11230	16362,5
23,86	11272	16353
24,0091	11209	16552,5
24,1582	11308	16885,5
24,3073	11318	16033,5
24,4563	11242	16610
24,6055	11231	17451
24,7546	11281	17411
24,9037	11234	17856
25,0529	11226	18184,5
25,202	11387	18328,5
25,3511	11336	18409
25,5003	11312	18462,5
25,6494	11289	18840,5
25,7985	11364	18933,5
25,9475	11436	19031,5
26,0964	11368	19060,5
26,2456	11514	18877,5
26,3947	11455	18523,5
26,5438	11261	18851
26,693	11295	19197,5
26,8419	11229	18817,5
26,991	11315	18354
27,1401	11361	17965,5
27,2892	11290	18078
27,4383	11418	18231,5
27,5875	11526	18166,5
27,7366	11646	18337,5
27,8857	11480	18554
28,0347	11441	18490
28,1838	11415	18520,5
28,3327	11458	18231
28,4818	11485	18108
28,6309	11455	18208,5
28,7801	11345	18067,5
28,9292	11253	18322
29,0784	11214	18296
29,2273	11290	18414
29,3763	11259	18839,5
29,5254	11177	19171
29,6746	11248	19593
29,8237	11165	19592,5
29,9728	11216	19763,5

30,122	11100	20184,5
30,2711	11175	20072,5
30,4202	11315	20549,5
30,5694	11196	20792
30,7185	11162	20814
30,8674	11349	20804
31,0166	11493	19209,5
31,1657	11438	19374
31,3148	11419	19272
31,4639	11420	19172
31,6131	11297	19346
31,762	11336	19238
31,9112	11313	19743,5
32,0603	11364	19495
32,2095	11406	19002,5
32,3586	11424	19334
32,5077	11336	19650
32,6568	11318	19780,5
32,806	11283	20140,5
32,9551	11357	20265
33,104	11430	20371,5
33,2532	11397	20428
33,4023	11351	20061
33,5514	11232	20560
33,7006	11278	20756
33,8497	11217	21322,5
33,9988	11276	21775
34,1478	11259	22154,5
34,2969	11271	22623
34,4459	11277	22965
34,595	11298	23429
34,7441	11248	23675
34,8932	11273	24248
35,0422	11282	24463,5
35,1913	11336	25289
35,3404	11191	25886
35,4895	10952	26764,5
35,6387	10938	27031
35,7878	11020	27322
35,9369	10776	27389,5
36,086	10807	27700
36,2352	10761	28250
36,3843	10740	29056,5
36,5332	10710	29099
36,6822	10760	29210,5
36,8313	10706	29776
36,9805	10751	30496,5
37,1296	10727	30680
37,2787	10698	31437,5
37,4278	10710	31932
37,577	10794	32132,5
37,7261	10864	32172

37,875	10672	32464,5
38,0241	10687	31062
38,1733	10573	32577
38,3224	10564	33310
38,4716	10582	33624,5
38,6207	10605	34744
38,7698	10563	36267
38,9188	10589	36109,5
39,0679	10564	35871
39,217	10554	35986
39,3662	10596	35286
39,5153	10701	34291,5
39,6644	10720	34672
39,8136	10711	34315,5
39,9627	10721	34600,5
40,1117	10712	35017
40,2608	10597	36418
40,4099	10498	36875,5
40,559	10525	37768
40,7082	10568	37889,5
40,8573	10538	38152
41,0064	10447	37897
41,1556	10440	37656
41,3045	10435	36645
41,4535	10508	36285,5
41,6026	10534	35793,5
41,7517	10497	35686,5
41,9009	10519	35422,5
42,0498	10499	35904
42,1991	10491	37466
42,3485	10443	38668,5
42,4978	10403	39420
42,6471	10429	40351
42,7965	10364	39371
42,9458	10355	40139
43,0952	10253	42166,5
43,2445	10259	43284,5
43,3939	10183	44285,5
43,5432	10252	45723
43,6924	10091	45879,5
43,8417	9980	46843,5
43,991	9901	47579
44,1402	9889	48680,5
44,2894	9883	49172,5
44,4387	9844	46921,5
44,5881	9781	45108,5
44,7374	9758	45424,5
44,8865	9749	44770,5
45,0359	9816	44863
45,1853	9942	44575,5
45,3344	9928	44543,5
45,4837	9782	45515

45,6331	9769	46005
45,7824	9668	45317,5
45,9318	9710	44110
46,0811	9667	45121
46,2305	9739	45422
46,3798	9782	45297
46,5291	9667	45845,5
46,6785	9574	45869,5
46,8278	9543	46656,5
46,9772	9627	45759
47,1263	9588	45885
47,2755	9553	48123,5
47,4248	9537	49193,5
47,5742	9459	48799,5
47,7233	9373	48292,5
47,8726	9333	48447
48,022	9352	48807,5
48,1713	9228	49791
48,3207	9297	49764
48,47	9238	48203
48,6193	9283	49339
48,7686	9260	49927,5
48,918	9220	47051,5
49,0673	9343	46623,5
49,2167	9331	48445,5
49,366	9326	51574
49,5153	9229	52679,5
49,6647	9132	52404,5
49,814	9143	52688
49,9633	9151	52682
50,1127	9031	52553,5
50,2618	8985	52702,5
50,4112	8972	53363
50,5603	8990	54162
50,7097	8982	53952,5
50,859	8878	54374
51,0083	8792	53552
51,1577	8696	52999,5
51,307	8665	53030
51,4564	8638	53169
51,6058	8587	52802,5
51,7549	8602	53328,5
51,9042	8513	53499
52,0536	8447	53262,5
52,2029	8401	52460
52,3523	8408	51320,5
52,5016	8381	47529,5
52,6509	8430	46063,5
52,8003	8492	44884
52,9497	8495	44581
53,099	8491	44214
53,2482	8463	43370

53,3973	8501	43860,5
53,5467	8489	44901,5
53,696	8539	45656,5
53,8452	8412	46126,5
53,9945	8388	46793,5
54,1438	8320	45819
54,2932	8289	45946,5
54,4425	8285	46648,5
54,5918	8292	47380
54,7412	8349	48874,5
54,8905	8368	50424
55,0399	8280	50589
55,1892	8229	50777,5
55,3385	8217	50575
55,4879	8251	50974
55,6372	8316	51704
55,7866	8274	53270
55,9359	8228	54349,5
56,0853	8201	54693,5
56,2344	8319	55630,5
56,3838	8322	55409,5
56,5331	8226	56162,5
56,6825	8214	57168
56,8318	8191	57691
56,9812	8167	57675,5
57,1303	8094	56866
57,2797	8101	57388,5
57,429	8116	58707
57,5784	8081	59934,5
57,7277	8069	58336
57,8771	8073	55306
58,0264	8133	57129,5
58,1757	8106	58135
58,3251	8037	59516
58,4744	7943	60731
58,6237	7954	60662
58,773	7939	60920
58,9224	7912	62393,5
59,0717	7946	63812
59,2209	7931	65331,5
59,3702	7900	64586,5
59,5196	7714	63190,5
59,6689	7718	63306
59,8183	7755	62460,5
59,9676	7663	57506,5
60,1169	7636	56256
60,2663	7655	57517
60,4156	7693	57606
60,5647	7685	57411
60,7141	7669	57686
60,8634	7665	55712,5
61,0128	7627	57139

61,1621	7679	58391,5
61,3112	7638	58438
61,4606	7645	57097
61,6099	7676	56387
61,7592	7791	56183
61,9085	7803	54927
62,0577	7749	53135
62,2069	7745	52848
62,3562	7690	54250
62,5055	7579	55830
62,6549	7515	56756,5
62,8042	7538	57269
62,9535	7591	57048,5
63,1026	7587	57103
63,252	7625	57899,5
63,4011	7659	58678,5
63,5505	7567	59625
63,6998	7601	61802,5
63,8491	7596	62335,5
63,9985	7584	62475,5
64,1478	7587	64131,5
64,2972	7613	63775,5
64,4465	7651	62378
64,5958	7632	62102,5
64,7452	7593	62169
64,8945	7593	62399
65,0436	7539	63472
65,1928	7512	64099,5
65,3422	7405	64586,5
65,4915	7254	66236
65,6409	7193	67092
65,7902	7129	66452,5
65,9396	7106	65884
66,0889	7050	65308
66,238	6978	65655
66,3872	6963	67367
66,5365	6952	66753
66,6859	6970	68732,5
66,8352	6936	68599,5
66,9845	6940	68811,5
67,1338	6887	68099,5
67,2832	6879	67227,5
67,4327	6819	66233,5
67,5821	6848	64941,5
67,7314	6845	64600
67,8807	6844	63153,5
68,0299	6806	62997
68,1791	6799	62238,5
68,3282	6787	62919,5
68,4776	6780	62606,5
68,6269	6727	61865
68,7762	6696	62507

68,9255	6689	63330,5
69,0747	6688	64077,5
69,224	6689	65019,5
69,3733	6718	65254
69,5227	6741	64838
69,672	6763	65264,5
69,8215	6808	65417,5
69,9711	6800	64022
70,1206	6837	62414
70,2701	6942	62916,5
70,4197	6949	63251,5
70,5692	6949	63742,5
70,7187	6941	64279,5
70,868	6975	63448
71,0176	7012	63538,5
71,1673	7049	66135,5

Supplementary Figure 6e **ECIS: OA vs RA FLS, 1st run (resistance - ohm)**

time	OA-FLS	RA-FLS
0,00055028	2479,1	3130,4
0,01603889	2619	3297,1
0,02929861	2764,3	3451,8
0,04261917	2916,5	3604,5
0,05594	3056,1	3726,7
0,06926056	3195,1	3830,9
0,08257694	3332,9	3930,2
0,09589778	3441,3	4014,5
0,10921833	3547	4088,8
0,12253028	3651,9	4165,5
0,13584667	3752,9	4213,8
0,1488425	3843	4255,9
0,16216306	3876,2	4340,3
0,17547944	3918	4417,9
0,18879583	3957,5	4508,7
0,20211639	3972,4	4589,4
0,21543278	4017,1	4687,6
0,22840694	4077,1	4824,7
0,24171889	4139,9	4946,1
0,25503972	4193,1	5037
0,26837333	4245,8	5116
0,28169389	4286,8	5158,8
0,29501028	4344,7	5191,3
0,30833528	4418,4	5205,7
0,32166028	4496,3	5254,9
0,33473833	4560,3	5285,2
0,34806333	4618,1	5299,7
0,36138833	4689,4	5318,7
0,37471361	4754,4	5349,8
0,38803861	4800,1	5372,5
0,40136361	4845,9	5429,3
0,41468417	4883,6	5500,5
0,428005	4919,5	5582,5
0,44132556	4961,4	5639,1
0,45464639	5001,4	5704,8
0,46796694	5076,1	5831,8
0,48128778	5134,6	5914,5
0,49461806	5216,3	6011,9
0,50793861	5291	6126,1
0,521255	5403,2	6209,3
0,53457583	5497,7	6367,4
0,547905	5545,2	6457,7
0,56122583	5523,4	6622,4
0,57454639	5491,2	6787,4
0,58787139	5412,3	6928
0,60119222	5362,4	7009,5
0,61451278	5294,5	7075,4

0,62783778	5211,1	7203,5
0,64115417	5104,8	7280,3
0,65447056	5003,6	7332,3
0,66778694	4959,4	7373,8
0,68111194	4941,8	7402,8
0,69444139	4919,1	7431,1
0,70775778	4968,5	7478,7
0,72107417	5035,1	7544,7
0,73439028	5227,1	7588,2
0,74771111	5467,9	7640,8
0,7610275	5697,6	7704,1
0,77434806	5893,5	7774,6
0,78766444	6070,2	7786,8
0,80098944	6205,4	7820,9
0,81431444	6277	7854,4
0,82763083	6316,7	7895,2
0,84094722	6348	7905,8
0,85427222	6253,5	7949,7
0,86759306	6151,1	7965,4
0,88090583	6045,3	7941,6
0,89422667	5944,2	7952,2
0,90753861	5808	7998,7
0,92085083	5690,4	8036,8
0,93417139	5599,4	8022,3
0,94749194	5516,2	8021,4
0,96081278	5493,7	8057,2
0,97412917	5435,8	7990
0,98744556	5431	7944,8
1,0007619	5393,3	7904,2
1,0140825	5320,5	7859,5
1,0274042	5244,6	7823,3
1,0407292	5202	7831,3
1,0540542	5204,6	7822,1
1,0673661	5153,8	7774,1
1,0806869	5063,4	7840,5
1,0940033	5021,8	7819
1,1073283	4902	7821,4
1,1206447	4787,1	7791,2
1,1339567	4696,8	7765,6
1,1472686	4647	7776
1,1605936	4660	7751,7
1,17391	4687	7714
1,1872222	4812,7	7660,1
1,2005428	5062,4	7677,6
1,2138678	5340,7	7615,9
1,2271928	5572,2	7588,9
1,2405136	5730,7	7517,6
1,2538256	5849	7541,4
1,2671464	5973,9	7501,6
1,2804583	6075,3	7494,1
1,2937789	6140,6	7428,5
1,3071039	6147,4	7397,2

1,3204203	6170	7395,5
1,3337367	6169,3	7346,6
1,3470617	6181,9	7299,7
1,3603867	6196,5	7316,3
1,3737075	6191,6	7305,6
1,3870239	6199,4	7329,2
1,4003403	6143	7281,3
1,4136653	6146,4	7279,1
1,4269817	6150,3	7268,5
1,4402936	6187	7297,3
1,4536056	6217	7284,6
1,4669219	6239,9	7258,5
1,4802428	6226,2	7289,4
1,4935589	6188,2	7255,4
1,5068797	6157,4	7200,6
1,5201917	6173,4	7202,8
1,5335125	6180,7	7145,4
1,5468375	6228,4	7133,3
1,5601633	6248,1	7131,3
1,5734797	6284,5	7114,5
1,5868006	6305,8	7073
1,6001256	6302,2	7083,4
1,6134419	6317,6	7023,8
1,6267581	6322,5	7030,9
1,6400875	6296,1	7033,9
1,6534125	6252,8	7028,4
1,6667289	6254,4	6989,7
1,6800408	6208,8	6909,2
1,6933617	6193,5	6860,4
1,7066781	6136,7	6821,2
1,7199944	6092,4	6776,5
1,7333064	6078,2	6757,5
1,7466269	6032,9	6735,7
1,7599433	6049,3	6751,9
1,7732683	6056,7	6786,3
1,7865806	6017,7	6754,9
1,7998969	6030,9	6775,4
1,8132219	6004,5	6722,4
1,8265469	5981,9	6730,6
1,8398675	5968,7	6710,4
1,8531839	5927,4	6705,1
1,8665047	5913	6636,6
1,8798167	5896,2	6666,8
1,8931461	5870,4	6627,1
1,9064622	5863,8	6563,3
1,9197786	5869	6512,6
1,933095	5824,4	6450,8
1,94642	5763,5	6448,8
1,9597408	5670,4	6409,6
1,9730528	5638,4	6402,3
1,9863778	5565,4	6409,7
1,9997028	5564,9	6393,8

2,0130278	5563,9	6381
2,0263486	5577,9	6356,3
2,0396692	5511,4	6343,2
2,0529942	5471,8	6317
2,0663192	5502	6293,5
2,0796444	5487,5	6270,5
2,0929617	5464,8	6269,3
2,1062781	5465,1	6271,2
2,1195944	5435,1	6257,4
2,1329194	5395	6200
2,1462358	5391,7	6199,2
2,1595478	5349,2	6174,4
2,1728728	5355,9	6159,1
2,1861933	5388,6	6178,2
2,1995142	5370,4	6156,1
2,2128347	5337,2	6157,6
2,2261556	5313,6	6092,2
2,2394761	5338,9	6088,1
2,2527969	5354,1	6071
2,2661219	5330,6	6101
2,2794469	5296,5	6081,6
2,2927678	5269,7	6096,5
2,3060883	5243,7	6045,6
2,3194133	5202	6025,2
2,3327297	5150,7	5978,7
2,3460461	5119,1	5992,8
2,3593625	5049,2	5969,8
2,3726917	4978,6	5931,9
2,3860081	4916,3	5886,9
2,3993244	4889,7	5835,4
2,4126453	4893,3	5863,4
2,4259658	4890,9	5831,4
2,4392867	4918,3	5826,2
2,4526072	4933,8	5819,6
2,4659192	4932,4	5813,6
2,47924	4908	5818,5
2,492565	4913,1	5833,5
2,5058814	4883,7	5860,3
2,5191933	4852,7	5838,7
2,5325142	4843,6	5800,7
2,5458306	4854,9	5775,1
2,5591556	4816,4	5743,6
2,5724719	4816,9	5707,3
2,5857925	4813	5714
2,5991175	4807,7	5680,3
2,6124425	4797,2	5657,9
2,6257544	4786,9	5645,4
2,6390753	4772,8	5614,6
2,6523958	4760,8	5594,4
2,6657211	4750	5557,4
2,6790417	4716	5509
2,6923581	4700	5502,9

2,70567	4663,3	5520,2
2,7189864	4576,9	5532,9
2,7323114	4505,7	5521
2,7456322	4438,4	5522,8
2,7589528	4389,7	5510,5
2,7722647	4359,4	5502,1
2,7855897	4325,1	5515,6
2,7988811	4284,7	5498,2
2,8122019	4249,5	5478
2,8255225	4222,3	5479,8
2,8388389	4189,5	5476,9
2,8521508	4169,2	5468,5
2,8654803	4170,9	5450,8
2,8788011	4130,3	5429,7
2,8921131	4093,2	5420,4
2,9054381	4091	5451,2
2,9187631	4050,6	5460,7
2,9320836	4067,7	5477,9
2,9454044	4069,2	5474,3
2,9587164	4059	5449,6
2,9720414	4068,6	5417,7
2,9853664	4081,4	5392
2,9986828	4101,7	5394,9
3,0120036	4111,8	5398,6
3,02532	4118,6	5414,5
3,0386364	4102,3	5388,5
3,0519569	4127	5357,9
3,0652819	4105,7	5385
3,0785983	4114,1	5402
3,0919189	4099,4	5413,6
3,1052442	4061,9	5425,9
3,1185647	4046,8	5434,9
3,1318856	4017,2	5468
3,1452017	3995,3	5473,1
3,1585139	4008,1	5472,3
3,1718389	4016	5449,4
3,1851639	3995,3	5451,9
3,1984803	3967,4	5440,3
3,2118053	3958,5	5412,5
3,2251347	3934,1	5446,2
3,2384508	3930,2	5470,6
3,2517717	3909,6	5451,4
3,2650933	3918,6	5470,6
3,2784139	3930,8	5476,4
3,2917303	3949,7	5499,7
3,3050467	3949,8	5520,1
3,3183717	3948,2	5521,9
3,3316925	3939,8	5506,6
3,3450131	3941,5	5478,9
3,3583381	3957,1	5476,4
3,3716631	3965,9	5469,5
3,3849794	3943,6	5463,4

3,3982914	3930,2	5444,5
3,4116122	3915,8	5454
3,4249372	3909,8	5422,8
3,4382622	3922,5	5402,5
3,4515831	3927,6	5401,4
3,464895	3923,3	5366,3
3,4782114	3925	5342,3
3,4915278	3949,3	5324,3
3,5045581	3977,2	5301,6
3,5178875	3986	5281,5
3,5312125	3977,2	5267,8
3,5445375	3987,8	5256,8
3,5578539	4013,4	5250,3
3,5711744	4035,7	5248,9
3,5844908	4008,4	5254,9
3,5978117	4023,4	5253,1
3,6111278	4009	5259,2
3,6244486	4007,4	5244
3,6377736	4008	5212,9
3,6510942	4013	5160,7
3,6644106	3986,3	5183,4
3,6777228	3928	5165,5
3,6910433	3936,4	5150,8
3,7043597	3907,6	5145,2
3,7176847	3920,9	5132,8
3,7310056	3909,7	5114,5
3,7443217	3909,2	5096
3,7576425	3903,1	5093,8
3,7709544	3880,9	5097,1
3,7842753	3866,6	5132,3
3,7975958	3839,2	5126,9
3,8109122	3857,6	5132,2
3,8242331	3878,6	5162,2
3,8375581	3883,5	5139,9
3,8508786	3890,1	5141
3,8642036	3901,1	5146,1
3,8775244	3920	5157,9
3,890845	3931	5179,7
3,9041658	3924,5	5163,7
3,9174733	3905,2	5167
3,9307942	3911,8	5177,2
3,9441147	3891,4	5213,6
3,957445	3874,3	5208
3,9707658	3888,4	5204,7
3,9840822	3891,6	5190,9
3,9973942	3890,5	5185,1
4,010715	3879,4	5183,6
4,02404	3885,6	5185,8
4,0373606	3902,6	5184,9
4,0506822	3904,4	5189,9
4,0640031	3885,1	5177
4,0773192	3881,8	5213,6

4,09064	3866,4	5222,9
4,1039606	3857,1	5235,8
4,1172728	3834	5230,7
4,1304114	3837,3	5233,5
4,1437278	3836,8	5222,1
4,1570572	3835,6	5219,9
4,1703778	3826,9	5229,8
4,1836897	3812,1	5219,8
4,1970147	3804,5	5282,1
4,2103311	3775,9	5306,2
4,2236561	3755,3	5294,3
4,2369811	3750,8	5304,3
4,2503019	3742,6	5303,8
4,2636183	3732,1	5293,3
4,2769347	3721,2	5295,1
4,2902511	3719,5	5280,3
4,3035847	3720	5283,2
4,3168967	3709,7	5302,6
4,3302217	3704,1	5329
4,3435467	3706,1	5376,7
4,3568675	3706,4	5387,9
4,3701839	3697,1	5387,5
4,3835044	3692,8	5384,2
4,3965219	3691,7	5399,4
4,4098425	3670,4	5409,9
4,4231633	3680,7	5388
4,4364839	3672	5371,7
4,4498089	3636,2	5407,9
4,4631253	3600,5	5423,1
4,4764383	3596,8	5456,1
4,4897547	3595,7	5489,2
4,5030883	3583,9	5479,6
4,5164178	3566	5471,4
4,5297342	3548,1	5476,5
4,5430503	3537,8	5455,9
4,5563667	3509,2	5436,3
4,5696917	3490,4	5426,7
4,5830167	3481,8	5410,3
4,5963331	3487,4	5403,2
4,6096453	3511,8	5381,8
4,6229703	3496,6	5357
4,6362953	3510,6	5360,7
4,6494208	3522,5	5300,1
4,6627417	3501,5	5285,4
4,6760536	3493	5266,3
4,6893786	3497,8	5251,3
4,7026994	3490,8	5254,7
4,71602	3475,1	5285,4
4,7293364	3471,4	5314,7
4,7426658	3486,5	5367,8
4,7557092	3499,4	5397,2
4,7690256	3488	5442,5

4,7823506	3522,1	5479,5
4,7956711	3558,2	5468,9
4,8087925	3568,8	5476,6
4,8221133	3561,4	5517,8
4,8354253	3564,7	5547,9
4,8487511	3568,3	5564,4
4,8620719	3577,9	5578,1
4,8753969	3571,8	5602,5
4,8887175	3571,5	5609,9
4,9020383	3564,2	5627,2
4,9153633	3575,5	5618,1
4,9286842	3592	5617,7
4,9420047	3618	5617,7
4,9553297	3649,5	5624
4,9686506	3695,9	5606,6
4,9819711	3763,6	5632,4
4,9952919	3801,3	5643,7
5,0086039	3836,4	5640,1
5,0219289	3836,5	5638,6
5,0352539	3815,1	5639,7
5,0485744	3855,3	5646,3
5,0618908	3874,6	5651,9
5,0752072	3864,8	5665,1
5,0885194	3874,8	5686,5
5,10184	3891,4	5658,8
5,1151608	3916,9	5677,6
5,1284814	3922,9	5685,7
5,1417892	3937,3	5701,6
5,1551097	3952,2	5675
5,1684306	4003,8	5672,7
5,1817511	4008,2	5652,7
5,1950806	4035,5	5629,1
5,2083969	4077,9	5657,6
5,2217133	4138,4	5672,4
5,2350383	4174,9	5652
5,24806	4190,1	5644,7
5,2613817	4201,4	5620
5,2746981	4212,1	5598
5,28801	4224	5592,8
5,301335	4235,2	5581,4
5,31466	4219,4	5600,2
5,3279764	4230,7	5594,4
5,34131	4254	5593
5,354635	4246	5566
5,36796	4219,4	5576,1
5,3812764	4196,9	5578,4
5,3945928	4211,5	5573,6
5,4079136	4205,9	5555
5,4212428	4185	5563,5
5,4345636	4209,2	5523,6
5,44788	4235,8	5509,6
5,461205	4238,6	5483,7

5,4745256	4243,7	5439,5
5,4878419	4235,7	5428,5
5,5011628	4244,2	5433,8
5,5144878	4270,2	5424,5
5,5278083	4290,6	5424,8
5,5411247	4293,4	5445,6
5,5544411	4283,8	5446,1
5,5677669	4304,2	5464,4
5,5810886	4298,6	5464,6
5,5944094	4289	5465,8
5,6077344	4275,9	5482,7
5,6210594	4285,6	5457,6
5,6343803	4313,3	5479,2
5,6476922	4329,2	5468,7
5,6610172	4339,5	5486,2
5,6743344	4338,3	5483,6
5,6876508	4328,6	5491,8
5,7009758	4310,5	5489,7
5,7142967	4278,7	5507,8
5,7276172	4291,1	5543,7
5,7409381	4292,9	5550,7
5,7542672	4326,3	5571,9
5,7675836	4320,1	5581,5
5,7809044	4331,4	5592,2
5,7942208	4299	5622,4
5,8075414	4272,4	5651,1
5,8208664	4272,9	5641,9
5,8341828	4287,6	5629
5,8475022	4252,6	5645,2
5,8608208	4230,1	5653,3
5,8741372	4203,6	5636,2
5,8874622	4213,1	5646,8
5,9007917	4209,2	5634,3
5,9141122	4187,2	5622,1
5,9274286	4205,3	5611,8
5,9407581	4198,5	5630,3
5,9540831	4219,9	5637,5
5,9673994	4226,6	5630,9
5,98072	4209,2	5620,9
5,9940492	4219,3	5642,6
6,0073711	4204,1	5634,1
6,0206917	4202,4	5641,9
6,0340125	4194	5635,3
6,0473375	4187,8	5607,6
6,0606625	4183,8	5609,8
6,0739744	4184,4	5592
6,0872953	4211,4	5622,9
6,1006158	4228,2	5650,5
6,1139322	4224,3	5661,7
6,1272486	4227,7	5665,3
6,1405736	4213,6	5666
6,15389	4195,1	5640,8

6,1672064	4186	5664,1
6,1805183	4175,9	5681,9
6,1938389	4189,4	5673
6,2071597	4188,8	5688,9
6,2204847	4192,2	5703,7
6,2338097	4198,3	5708,2
6,2471392	4222,5	5718,5
6,2604597	4226,5	5706,5
6,2737806	4243,9	5699,4
6,2871056	4274,9	5681,6
6,3004306	4267,6	5698,1
6,3137511	4263,1	5719,3
6,3270589	4257,4	5760,5
6,3403797	4230,3	5793,4
6,3537014	4237,6	5793,5
6,3670142	4245,5	5825,6
6,3803436	4241	5819,6
6,3936642	4228,1	5853,9
6,4069806	4219,1	5851,7
6,4203056	4214,6	5887,2
6,4336264	4201,2	5905,2
6,4469469	4183,8	5910
6,4602719	4164,2	5893,8
6,4735928	4151,3	5857
6,4869047	4159,1	5852,9
6,5002297	4163,1	5856,1
6,5135506	4182,1	5845,2
6,5268756	4182,7	5849,7
6,5401919	4179,9	5867,3
6,5535083	4191,7	5871,8
6,5668289	4193,9	5836,6
6,5801583	4140	5793,3
6,5934747	4136,7	5767,3
6,6067953	4126,6	5736,7
6,6200997	4167,6	5705,8
6,6334117	4240,1	5693,9
6,6467281	4288,7	5681,2
6,6600531	4320,4	5684,8
6,6733736	4338,9	5737
6,6866858	4341,8	5770,1
6,7000064	4317,4	5812,3
6,7133314	4286,3	5797,4
6,7266564	4260,9	5790,7
6,7399728	4222	5810
6,7532936	4200,1	5822,8
6,7666228	4192,2	5833
6,7799481	4123,8	5852,7
6,7932686	4104,3	5875,6
6,806585	4118,2	5850,5
6,8199014	4154,1	5828,4
6,8332219	4218,1	5831,4
6,8465469	4251,4	5834,3

6,8598633	4268,3	5860,6
6,8731842	4288,1	5843,6
6,8865006	4260,3	5848,1
6,8998256	4257,5	5832
6,9131461	4236,6	5844
6,9264581	4224,8	5860,3
6,9397744	4204	5862,7
6,9530908	4174,8	5865,4
6,9664158	4176,5	5866,8
6,9797367	4190	5882,3
6,9930617	4170,3	5892,8
7,0063781	4175,4	5910,1
7,0197031	4163,6	5968,4
7,0330236	4159,1	5996,6
7,0463314	4189,4	6035,2
7,0596519	4211,9	6105,1
7,0729772	4224,8	6135,5
7,0862933	4272,7	6151,5
7,0996142	4306,6	6156,9
7,1129436	4328,7	6176,7
7,1262686	4359,8	6198,5
7,139585	4327,7	6207,3
7,1528894	4328,9	6241,3
7,1662103	4338	6221,8
7,1795361	4326,2	6228,6
7,1928611	4320,6	6223,7
7,2061819	4317,2	6224
7,2195069	4277,1	6197,3
7,2328275	4272,5	6201,5
7,2461483	4243,1	6213,3
7,2594689	4252,7	6188,8
7,2727853	4270,9	6140,5
7,2861061	4260,7	6104,4
7,2994267	4240,3	6099,1
7,3127475	4209,2	6131,3
7,3260636	4187,8	6149,2
7,3393889	4209,8	6184,2
7,3527094	4214,8	6188,6
7,3660303	4212,6	6198,1
7,3793464	4207	6201,5
7,3926628	4223,9	6147,5
7,4059836	4210,9	6118,3
7,4193	4207,5	6096
7,432625	4208,6	6071
7,4459456	4177,7	6072,9
7,459275	4147,4	6069,1
7,4725956	4145,1	6084,6
7,4859164	4190,6	6072,5
7,4992414	4195,7	6045,7
7,5125619	4212,6	6072
7,5258783	4204,6	6096,2
7,5392033	4212	6120,1

7,5525286	4221,6	6100,4
7,5658536	4238	6084,9
7,5791742	4276,4	6064,9
7,5924861	4297,3	6055
7,6058111	4329,1	6070,5
7,6191364	4365,4	6035,5
7,6324525	4429,9	6038,5
7,6457778	4459,2	6030,6
7,6590983	4480,5	6025
7,6724189	4531,1	6008,7
7,6857147	4545,5	6006,1
7,6990397	4554,8	6020,8
7,7123614	4562,5	6066,1
7,7256864	4526,2	6085,7
7,7390069	4528	6102,2
7,7523278	4548,1	6089,8
7,7656397	4538,3	6100,4
7,7789606	4568,2	6106,8
7,7922811	4598,8	6103
7,8055931	4619,5	6058,8
7,8189094	4642,7	6019,2
7,8322344	4636,2	5963,2
7,8455553	4611,3	5929,5
7,8588717	4565,5	5921,6
7,8721967	4555,9	6001,2
7,8855217	4513,3	6139
7,8988381	4551,3	6232,8
7,9121631	4593,4	6277,8
7,9254836	4618,9	6248,4
7,9388131	4645,2	6232,3
7,952125	4653,3	6188,1
7,9654458	4636,6	6134,3
7,9787664	4637,3	6084,4
7,9920914	4633,5	6049,7
8,0054078	4656,9	6041,8
8,0187372	4690,9	6038
8,0320578	4716,5	6040,3
8,0453786	4742,7	6053,2
8,0586992	4746,1	6074,7
8,0720156	4748,5	6107,2
8,0853406	4763,2	6117,8
8,0986569	4745,2	6103,8
8,1119778	4696,1	6106
8,1252942	4677,9	6125
8,1386233	4638,9	6163
8,1519497	4616,2	6178,5
8,1652661	4609,3	6164
8,1785825	4592	6160,2
8,1919031	4572,9	6174
8,2052281	4572,1	6181,9
8,2185531	4585,5	6188,4
8,2318694	4597,6	6212,4

8,2451858	4604,9	6235,2
8,2585108	4592,3	6277,6
8,2718317	4584,7	6321,2
8,2851567	4593,3	6319,1
8,2984772	4616,4	6315,9
8,3118022	4619,7	6356,9
8,3251272	4632,9	6340
8,3384522	4660,7	6299,5
8,35177	4624,2	6268,2
8,3650864	4619,7	6251,9
8,3784028	4597	6230,5
8,3917233	4582,3	6237,4
8,4050442	4571,4	6246,3
8,4183647	4577,7	6272,3
8,4316853	4628,8	6260
8,4450061	4661,9	6247,9
8,4583267	4734,6	6262,8
8,4716475	4786,9	6275
8,4849681	4847,9	6296,5
8,4982803	4844,7	6317,6
8,5116053	4866,6	6333
8,5249303	4867,8	6334,5
8,5382508	4829	6352,9
8,5515631	4798,5	6353,3
8,5648836	4810,4	6342,6
8,5782086	4776,2	6357,2
8,5915306	4725,4	6379,1
8,6048514	4727,1	6341,1
8,6181775	4705,7	6316,2
8,6314983	4686,7	6303,6
8,6448147	4661,2	6268,4
8,6581267	4614,6	6237,5
8,6714517	4573	6249,8
8,6847811	4565,5	6244
8,6980972	4579,4	6260,4
8,7114181	4576,5	6243,6
8,7247386	4590,5	6225,2
8,7380681	4607,8	6215,3
8,7513931	4579,5	6180,3
8,7647094	4560,4	6147,2
8,7780214	4541,2	6118,7
8,7913422	4548,8	6107
8,8046672	4543,2	6080,9
8,8179878	4586,8	6041,6
8,8313086	4554,3	6070,6
8,8446336	4564,6	6082
8,8579586	4562,8	6084,6
8,8712836	4588,2	6078,9
8,8846	4592,7	6106,1
8,8979208	4584	6077,7
8,91125	4573	6060,7
8,9245708	4576,9	6052,7

8,9378872	4607,2	6056,5
8,9512122	4595,6	6062,6
8,9645328	4584,5	6074,6
8,9778536	4567	6069,7
8,99117	4565,1	6072
9,0044697	4603,9	6093,9
9,0177861	4619	6092
9,0311069	4656,7	6097,6
9,0444319	4670,6	6095,3
9,0577569	4686,1	6096,8
9,0710775	4704,6	6112
9,0844028	4704,1	6103,6
9,0977278	4679,3	6074,1
9,1110442	4637,1	6062,8
9,1243647	4638,8	6077,6
9,1376767	4645,7	6072,3
9,1509975	4663,7	6041,8
9,1643181	4654,4	6050,8
9,1776389	4689,2	6028,2
9,1909639	4723,5	6026,6
9,2042844	4702,5	6035,3
9,2176008	4665,6	6020,5
9,2309172	4665,9	6019,4
9,2442422	4643,2	6031,8
9,2575631	4593,2	6050,7
9,2708806	4569,7	6068,1
9,2842056	4553,1	6065,6
9,2975275	4522,7	6076
9,3108483	4526,8	6077,9
9,3241775	4518,1	6087,8
9,3374939	4534,8	6064,4
9,3508189	4553,8	6062,9
9,3641397	4520,2	6039,1
9,3774603	4523,1	6023,4
9,3907853	4537,9	6026
9,4041103	4566,4	6000,1
9,4174311	4565,2	6002,3
9,4307517	4578,9	5989,9
9,4440681	4607,8	6008,7
9,4573931	4682,9	6020
9,4707139	4728,5	6043
9,4840389	4804,1	6102,7
9,4970606	4849,5	6134,1
9,5103769	4856,4	6182,4
9,5236975	4857,7	6262
9,5370183	4850	6304,4
9,5503433	4862,6	6341,5
9,5636639	4838,5	6375
9,5769761	4836,4	6324,9
9,5902922	4877,6	6306,9
9,6036131	4883,7	6303,7
9,6169336	4861,4	6282,7

9,63025	4824,8	6291,9
9,6435708	4838,2	6326
9,6568925	4882,8	6330,2
9,6702131	4901,4	6319,5
9,6835381	4890,4	6309,5
9,6968589	4905,6	6331,7
9,7101794	4889,5	6307,1
9,7235003	4845,2	6331,6
9,7368208	4844,2	6328,8
9,7501458	4814,1	6332,2
9,7634622	4802,1	6359,6
9,7767744	4819	6363,4
9,7900906	4827,8	6397,2
9,80342	4839,8	6394,6
9,8167494	4830,2	6386
9,83007	4826,8	6412,1
9,8433864	4809,9	6470,6
9,8567028	4829,3	6454,2
9,8700278	4809,4	6455,1
9,8833528	4811,9	6433,1
9,8966647	4859,4	6417,2
9,9099769	4829,3	6357,5
9,9232975	4807,8	6362,1
9,9366139	4816,1	6318,4
9,9499356	4830,7	6330
9,9632606	4861,6	6321
9,9765769	4926,5	6300
9,9898933	4951,3	6300
10,003214	4965,5	6290,4
10,016535	4980,2	6299,5
10,029851	4970,3	6293,4
10,043163	4996,7	6298,4
10,056479	4998,7	6298,1
10,069809	4987,3	6304,3
10,083134	4990,1	6314,7
10,096112	4994,9	6336,4
10,109424	4966,6	6327,9
10,122745	4971,6	6359
10,136061	4974,6	6369,6
10,149392	4987	6367,7
10,162712	4951,3	6379
10,176029	4943,5	6374,9
10,189342	4952,9	6415,7
10,202658	4939,2	6434,2
10,215979	4909,5	6443,4
10,229304	4908,7	6443,3
10,24263	4933,7	6427,6
10,255951	4948	6442,5
10,269276	4954,5	6385,6
10,282592	4944,9	6343,1
10,295917	4948,2	6313,6
10,309238	4907,2	6278,4

10,322558	4888,8	6280
10,335879	4874	6314,4
10,349204	4873	6384
10,362525	4867,4	6379,1
10,375845	4850	6392,1
10,389157	4855,3	6384,9
10,402478	4875	6401,3
10,415799	4899,3	6419,8
10,429119	4954,4	6403
10,44244	4932,6	6397,5
10,455756	4945,9	6408,1
10,469069	4960,7	6451,7
10,482391	4961,5	6457,6
10,495708	4899,1	6459,3
10,509037	4878,1	6464,4
10,522358	4909,1	6492,5
10,535679	4889,9	6472,7
10,548995	4867,2	6434,6
10,562316	4836,7	6420,5
10,575641	4853,5	6408,7
10,588961	4842,8	6426,3
10,602282	4862,1	6422,9
10,615611	4835,2	6390,7
10,628933	4777,8	6405,3
10,642258	4758,4	6426,3
10,655583	4722,2	6413,1
10,668904	4670	6415,8
10,682216	4620,3	6422
10,695541	4598,5	6443,8
10,708874	4580,2	6413,4
10,722199	4582,8	6400,5
10,735516	4611,1	6410,6
10,748845	4605,5	6430
10,762166	4607,8	6415,3
10,775487	4617	6380,1
10,788803	4598	6398,4
10,802115	4621,2	6435,9
10,81544	4648,4	6396,3
10,828761	4693,5	6375,2
10,842081	4715,1	6383,3
10,855398	4714,3	6375,8
10,868723	4718,9	6344,7
10,882048	4708,4	6331,5
10,895368	4683,4	6315
10,908681	4693,4	6298,2
10,922002	4696,2	6283,3
10,935327	4668,3	6261,1
10,948644	4687,6	6257,7
10,96196	4690,4	6250,7
10,975281	4681,3	6274,4
10,988592	4684,9	6303
11,001913	4681,6	6328,9

11,015238	4661,4	6334,2
11,028555	4673,2	6321,9
11,04188	4697,7	6323,8
11,0552	4715,5	6323
11,068521	4699	6329
11,081846	4679,1	6343
11,095167	4649,6	6311
11,108488	4608,3	6277,1
11,121813	4592,5	6264,6
11,135126	4607,6	6257
11,148451	4600,1	6284,4
11,161772	4598	6248,2
11,175102	4588,9	6227,2
11,188418	4614,5	6215,7
11,201734	4647,3	6189,5
11,215046	4689,2	6198,6
11,228363	4684,2	6153,1
11,241684	4687,8	6163,8
11,255001	4659,7	6175,5
11,268326	4647,1	6205,1
11,281642	4597,6	6194,6
11,294963	4586,1	6200,3
11,308284	4606,5	6223,1
11,321604	4622,3	6209,4
11,334925	4637,1	6191,2
11,348241	4635,8	6185,5
11,361566	4636,6	6137,8
11,374887	4642,8	6116,2
11,388208	4649,7	6100,4
11,401524	4659,5	6096,3
11,414845	4668,6	6121,3
11,428166	4705,1	6113,4
11,441482	4721,7	6131,6
11,454804	4739,9	6129,7
11,468124	4718,6	6145,6
11,481445	4707,6	6137,7
11,494766	4720,7	6147,6
11,508091	4719	6115,4
11,521416	4727,1	6094,2
11,534737	4718,5	6093,5
11,548053	4714,5	6098
11,56137	4731,7	6120,7
11,574687	4733,1	6143,4
11,588003	4713,8	6146,8
11,601324	4715,7	6151,7
11,61464	4704,7	6132
11,627965	4703	6076
11,641286	4727,2	6077,9
11,654606	4740,6	6068,9
11,667927	4714,6	6048,9
11,681252	4704,3	6058,4
11,694577	4721,1	6093,9

11,707898	4728	6128,6
11,721223	4725,8	6146
11,734544	4730,6	6192,6
11,74786	4726,2	6158,5
11,761181	4725,8	6127,9
11,774497	4733,8	6087,4
11,787813	4712,5	6064
11,801138	4735,7	6044
11,81445	4757,6	6041
11,827763	4736,2	6005,6
11,841074	4757,5	6009,8
11,854395	4777	6029,7
11,867711	4800,6	6001,9
11,881036	4784,1	5978,6
11,894357	4788,9	5998,5
11,907682	4796	5988,3
11,921003	4808,1	5984,2
11,934328	4810,9	5994,7
11,947644	4817,7	5999,2
11,960965	4840,9	5996,9
11,974286	4854,7	5975,1
11,987612	4871	5979,3
12,000937	4889,3	5957,2
12,014253	4918,7	5951,2
12,027574	4945,4	5980,4
12,04089	4938,3	5999,5
12,05419	4936,6	5988,6
12,067502	4898,2	5990
12,080832	4854,1	5973,5
12,094152	4848,2	5983,9
12,107473	4850,8	5964,5
12,120794	4873,3	5965,6
12,134119	4858	5956,3
12,147445	4836,7	5956,2
12,160764	4793,9	5930,8
12,174088	4766,7	5908,7
12,187413	4752,1	5917,7
12,200724	4776	5938,7
12,214041	4756,7	5970,1
12,227361	4776,9	5924,5
12,240682	4778,6	5939,4
12,253999	4769,5	5919,6
12,267315	4768	5917
12,280636	4746,5	5918,9
12,293969	4750,2	5900,9
12,307299	4767,9	5889,7
12,320619	4774	5892,4
12,333936	4749,1	5894,6
12,347265	4774,2	5905,1
12,36059	4735,5	5911
12,373902	4755,8	5937,2
12,387214	4773,1	5930,1

12,400539	4758,4	5945
12,413864	4761,2	5989,9
12,427181	4759,6	5992,1
12,440497	4729,4	5972,6
12,453817	4719,5	5930,3
12,467147	4710,8	5949,8
12,480469	4694,9	5951,6
12,493794	4682,9	5946,1
12,50711	4654,8	5929,6
12,520422	4648,9	5943,9
12,533739	4646,6	5931,6
12,547056	4648	5954,5
12,560381	4643,6	5962,3
12,573697	4650,3	5947,7
12,587018	4648,9	5947,4
12,600334	4627,7	5959,8
12,613655	4597,9	5992,1
12,626976	4583,2	5977
12,640296	4600,8	5967,3
12,653613	4605,3	5976,6
12,666929	4589,4	5964,3
12,680254	4593,3	5954,6
12,693579	4595,5	5950,8
12,706896	4623,2	5908,1
12,720212	4614,4	5881,6
12,733533	4584,9	5876,4
12,746849	4550,2	5884,6
12,760165	4524,8	5896,6
12,77349	4524,2	5877,1
12,786811	4528,4	5884,2
12,800127	4544,3	5888,7
12,813454	4543,1	5884,2
12,826783	4551,1	5844,3
12,840099	4551,6	5848,7
12,85342	4539,6	5855,5
12,866741	4523,2	5847,3
12,880066	4490,2	5797
12,893391	4470,7	5798,5
12,906703	4476,5	5781,7
12,920015	4466,2	5770,2
12,933336	4483,9	5760,6
12,946652	4466,2	5754,2
12,959978	4450,7	5720,8
12,973303	4437,7	5712,3
12,986619	4444	5684,9
12,99994	4453,6	5686
13,013261	4463,4	5687,9
13,026581	4470,8	5677,9
13,039902	4484,3	5674,9
13,053223	4489,2	5683,7
13,066539	4507	5705,9
13,079864	4489	5728,9

13,093193	4489,1	5763,1
13,10651	4493,8	5770,9
13,119826	4484,6	5805,4
13,133151	4474,3	5806,6
13,146467	4481,2	5797,2
13,159785	4481,2	5780,5
13,173101	4471,5	5774,9
13,186413	4452,3	5760,1
13,199738	4437,9	5746,4
13,213059	4441,3	5758,7
13,226375	4461,7	5733,5
13,239696	4483	5710,9
13,253012	4460,3	5704,3
13,266324	4454,5	5701,3
13,279641	4430,7	5711,3
13,292961	4427,9	5695,8
13,30628	4411,3	5685,8
13,319605	4413,5	5676,9
13,332921	4406,6	5650,7
13,346242	4393	5660,7
13,359567	4438,2	5678
13,372896	4455,3	5661,1
13,386217	4437,7	5679,6
13,399529	4431,5	5670,7
13,412841	4426,9	5666,8
13,426161	4469,4	5691,3
13,439201	4495	5661,6
13,45253	4523,2	5663,1
13,465851	4543,6	5672,2
13,479176	4520,6	5709,6
13,492505	4505	5714,6
13,50583	4494,6	5734,3
13,519142	4487,7	5775,2
13,532467	4447,4	5771,1
13,545796	4449,5	5787,6
13,559117	4449	5759,8
13,572433	4427,2	5781,1
13,585758	4433,8	5773,6
13,599079	4439,6	5780,6
13,6124	4455,4	5774,9
13,625716	4463,8	5770,5
13,639033	4487,6	5772,4
13,652353	4508,7	5785,9
13,665674	4532,1	5791,2
13,678986	4545,3	5801,8
13,692311	4564,1	5819,1
13,705636	4573,3	5823,6
13,718957	4568,8	5833
13,732278	4576,2	5833,2
13,745589	4608,1	5860,1
13,758906	4637,9	5849,3
13,772231	4655,4	5836,7

13,785543	4660,3	5852,9
13,798868	4670,3	5822,9
13,812189	4673,8	5839,4
13,825509	4695,7	5839,4
13,838836	4728,2	5832,6
13,852156	4763,2	5833,3
13,865468	4773,5	5842,3
13,878793	4811,1	5834,5
13,892109	4804,2	5852,7
13,905422	4797,7	5858,3
13,918734	4797,6	5886,4
13,932063	4814,7	5896,4
13,945354	4800,2	5910,7
13,95868	4777	5898,5
13,972005	4793,2	5897,4
13,985325	4801,4	5858,6
13,998646	4810,1	5884,9
14,011971	4797,5	5894
14,025292	4814,6	5895,5
14,038601	4837,3	5893,5
14,05193	4827,4	5902,4
14,065251	4820,9	5919,6
14,078571	4840,9	5904
14,091896	4834,2	5900,2
14,105221	4820,1	5868,8
14,118542	4831,8	5845,3
14,131863	4825,8	5888,6
14,145184	4822,7	5891,3
14,158513	4820,7	5870,1
14,171834	4821,8	5860,2
14,185129	4800,3	5855,3
14,198446	4782,6	5862,9
14,211762	4753,4	5881,3
14,225087	4747,5	5896,8
14,238407	4755	5889,2
14,251728	4748	5882,1
14,265045	4750,3	5862,6
14,27837	4769,8	5861,3
14,291695	4760,8	5874,1
14,305011	4763,5	5864,2
14,318323	4778,3	5820,7
14,331644	4766,4	5829,1
14,34496	4777	5801,5
14,358285	4780,4	5801,7
14,371601	4787,7	5813,1
14,384918	4797,9	5804,1
14,398234	4791,8	5838,6
14,411546	4783,8	5827,7
14,424871	4772,7	5815,1
14,438201	4782,2	5819
14,451517	4795,4	5824
14,464838	4828,2	5843,7

14,478158	4857	5831
14,491475	4875,5	5795,3
14,504795	4861,2	5771,7
14,518116	4834,6	5782,3
14,531437	4827,4	5791,6
14,544757	4823	5802,7
14,557827	4815,6	5792,3
14,571148	4817,2	5746,8
14,584468	4823	5724,6
14,597793	4831	5760,4
14,611114	4816,7	5761,9
14,62443	4793,7	5767,9
14,637747	4807,1	5764,8
14,651059	4810,7	5739,5
14,664388	4829,2	5731,5
14,677704	4836,2	5762,8
14,690674	4860,4	5756,1
14,703999	4832,2	5760,9
14,71732	4846,8	5756,7
14,730632	4831,3	5741,6
14,743953	4825,8	5743,7
14,757278	4838,1	5727,7
14,770594	4845,5	5721
14,783914	4863,3	5707,3
14,797235	4863,6	5712,7
14,810556	4884,2	5715,1
14,823877	4888,7	5728
14,837189	4875,5	5709,5
14,850509	4857,4	5700,1
14,863826	4865,7	5671,1
14,877142	4844,4	5682,6
14,890463	4817,8	5672,6
14,903783	4815,3	5662,9
14,917104	4812,1	5674,6
14,930421	4793,9	5659,9
14,943746	4839	5635,4
14,957071	4852,2	5642,1
14,970387	4870	5657,6
14,983703	4860,3	5660,7
14,997015	4872,3	5651,6
15,010336	4889,6	5647,8
15,023657	4888,5	5634,5
15,036983	4891,1	5631,5
15,050308	4886,5	5660,7
15,063624	4894,5	5647,4
15,076932	4876	5639,2
15,090257	4884,8	5641,3
15,103578	4889,6	5629
15,116894	4902,3	5625,4
15,130219	4902,5	5637,4
15,143536	4894,9	5614,9
15,156852	4907,3	5577,9

15,170177	4909,2	5575,8
15,183502	4895	5560,9
15,196818	4896,6	5561,8
15,210139	4905,9	5586,9
15,223464	4851,7	5563,3
15,236776	4890,8	5573,9
15,250101	4911,9	5584,3
15,263422	4922	5563,3
15,276738	4936,4	5527,6
15,290067	4966,2	5517,9
15,303397	4944,2	5514,9
15,316713	4950,2	5515,4
15,330038	4939,8	5529,9
15,343367	4942,4	5547,1
15,356692	4941,9	5565,1
15,370013	4938,2	5559,8
15,383329	4957,7	5565,7
15,396654	4929,8	5592,2
15,409984	4889,2	5592,2
15,423305	4890,6	5605
15,436621	4886,8	5605,6
15,449933	4862,6	5609,3
15,463254	4850,3	5622,9
15,47657	4837,4	5631,2
15,489891	4833,2	5648,3
15,503207	4859,1	5625,6
15,516528	4840,5	5612,5
15,529844	4847,5	5614
15,543165	4816,6	5626,9
15,55649	4800,6	5611,1
15,569806	4806,8	5627,9
15,583114	4802,7	5640,9
15,596434	4765,8	5669,1
15,609764	4749,3	5688,5
15,623089	4773,6	5704,7
15,636401	4795,8	5715,6
15,64973	4799,6	5730,1
15,663051	4792	5718,1
15,676372	4777,1	5717,3
15,689697	4800,1	5701,2
15,703017	4814	5696,8
15,716342	4815,4	5700,8
15,729663	4811,9	5714,6
15,742992	4811,4	5698,2
15,756313	4820	5716,5
15,769629	4791,7	5738,3
15,78295	4763,7	5714,1
15,796271	4754,2	5687,4
15,809596	4743,9	5687,5
15,822917	4734,5	5663,5
15,836233	4735,8	5635,7
15,849554	4728,5	5630,5

15,862879	4714,3	5634,2
15,876199	4723,1	5607,9
15,889511	4744,4	5593
15,902841	4729,9	5610,7
15,916166	4743,4	5623
15,929486	4757,5	5595,9
15,942807	4738,5	5604,6
15,956128	4753,4	5623,4
15,969457	4770,5	5627,4
15,982778	4794,2	5628
15,996094	4810,2	5637,2
16,009419	4758,8	5631,2
16,022744	4724,9	5632
16,036069	4736,4	5644,7
16,049386	4749,9	5629,4
16,062698	4726,9	5625,8
16,076018	4726,9	5638,1
16,089335	4697,8	5638,6
16,102656	4669,7	5632,2
16,115981	4675,4	5633
16,129297	4654,9	5632,8
16,142619	4652,3	5660
16,155944	4646,5	5671,6
16,169264	4644,3	5670,5
16,182585	4620,8	5672,3
16,19591	4600,2	5668,3
16,209231	4598,4	5665,8
16,222552	4625,9	5643,5
16,235877	4642,6	5633,5
16,249197	4632,4	5612,4
16,262514	4623,7	5600,4
16,27583	4601,2	5597,6
16,289155	4585,8	5614
16,30248	4587,7	5621,8
16,315801	4587,2	5635,2
16,329117	4568,4	5649,5
16,342429	4562,8	5661,1
16,355754	4565,7	5667,7
16,369079	4556,8	5662,9
16,382391	4539,4	5640,8
16,395712	4527,1	5653,8
16,409028	4511,7	5662,6
16,422353	4506	5638
16,435674	4496,4	5625,8
16,448995	4524,7	5601,3
16,462315	4507,5	5615
16,475627	4508,3	5588,8
16,488948	4501,6	5584,4
16,502278	4512,5	5581,2
16,515594	4507,2	5581,2
16,52891	4512,5	5583,6
16,542231	4487,2	5582,2

16,55556	4488,9	5598,7
16,568889	4504,3	5605,9
16,582202	4511,5	5600,1
16,595527	4531,2	5595,6
16,608852	4516,6	5584,7
16,622181	4526,3	5566,3
16,635502	4523,6	5607,5
16,648822	4515,1	5621,8
16,662139	4496,2	5614,5
16,675459	4471,3	5629,3
16,68878	4479	5643
16,702101	4486,2	5644,5
16,715417	4507,2	5638,9
16,728733	4543,4	5619,9
16,742054	4551,9	5588,6
16,755379	4550,8	5582,3
16,768704	4556,8	5576,7
16,782025	4557,9	5559,9
16,79535	4578,8	5539,6
16,808666	4610,9	5537,7
16,821987	4638,9	5552,1
16,835308	4675,5	5552,5
16,848624	4669	5547,5
16,86194	4641,2	5559,6
16,875265	4650,5	5568,5
16,888582	4642	5564,1
16,901898	4633,6	5557,2
16,915219	4653,7	5552,1
16,928544	4658,8	5557,2
16,941869	4661,8	5555,3
16,955194	4659,5	5543,6
16,968514	4643,9	5544,7
16,981835	4629,6	5538,2
16,995156	4623,7	5528,6
17,008485	4624,2	5507,9
17,021806	4629	5505,8
17,035131	4627,9	5497,2
17,048452	4679,2	5491,4
17,061777	4659,9	5499,1
17,075097	4673,8	5498,8
17,088409	4691,3	5523,6
17,101734	4709,2	5502,8
17,115055	4717,7	5501,3
17,12838	4717,6	5481
17,141701	4723,1	5484,6
17,155013	4729,5	5473,2
17,168334	4718,7	5490
17,181663	4739,1	5479,5
17,194979	4725	5494,4
17,208296	4725,2	5465,6
17,221616	4717,1	5456,4
17,234941	4699,8	5469,4

17,248266	4697	5490,7
17,261578	4686,2	5476,7
17,274903	4696,8	5485,4
17,288224	4722,2	5491,9
17,301549	4692	5495,9
17,314879	4704,2	5497,7
17,328204	4696,9	5478,7
17,34152	4696,4	5460,8
17,354836	4687,7	5455,7
17,368153	4692,5	5474,3
17,381473	4669,5	5465,8
17,39479	4659,5	5497,7
17,408102	4640	5495,9
17,421418	4620,6	5499,9
17,434743	4598,4	5490
17,448064	4561,5	5465,4
17,461389	4568,9	5471
17,474709	4547	5455,4
17,488026	4512,1	5465,6
17,501346	4480,8	5457,8
17,514667	4469,8	5464,1
17,527984	4480,1	5434,2
17,5413	4456,3	5412,4
17,554616	4440,4	5424,1
17,567933	4439,9	5398
17,581253	4464,2	5363,4
17,594571	4467,2	5382,1
17,607891	4465,1	5370,5
17,621203	4458,7	5337,4
17,634524	4446,3	5334,2
17,647853	4445	5323,8
17,661178	4444,5	5331,9
17,67449	4435	5356,2
17,687803	4428,2	5370,5
17,701114	4410,6	5366,5
17,714439	4424,2	5385,7
17,727764	4450	5416,5
17,741085	4456,9	5408,4
17,75441	4435	5409,6
17,767731	4415,3	5425
17,781052	4396	5453,7
17,794368	4366,2	5462,2
17,807693	4321,8	5480,8
17,821009	4309,4	5468,4
17,834321	4288,7	5449,7
17,847642	4252,9	5431,5
17,860963	4292	5413,6
17,874275	4290	5409,6
17,887596	4291,7	5416,8
17,900912	4290	5420,9
17,914233	4286,5	5408,5
17,927549	4286,4	5419,6

17,940869	4279,2	5421,5
17,95419	4277,4	5442,2
17,967507	4264,6	5433
17,980827	4265,5	5430,4
17,994139	4241,4	5419,3
18,007464	4254,8	5410,7
18,020781	4262,4	5407,4
18,034097	4254,6	5401,1
18,047426	4243,9	5394,5
18,060747	4233	5393,4
18,074063	4228,6	5389,1
18,087388	4241,3	5406,5
18,100714	4267,4	5422
18,114039	4269,5	5435,9
18,127355	4279,4	5462,7
18,14068	4281,6	5449,5
18,153996	4286,7	5462,3
18,167317	4278,6	5466
18,180637	4283,9	5484,6
18,193958	4283,4	5476,2
18,207283	4286,2	5501
18,220608	4292,9	5511
18,233925	4300,7	5491,7
18,247246	4280,8	5489,9
18,260571	4266,9	5508,3
18,273887	4256,2	5522,2
18,287199	4270,5	5523,4
18,300515	4273,3	5485,7
18,313832	4272,7	5489
18,327148	4265,5	5512,4
18,34046	4270,4	5514,2
18,353785	4267,7	5503,3
18,367101	4252,7	5502,2
18,380422	4249,9	5500
18,393738	4251,6	5503
18,40705	4256,1	5510,3
18,420375	4252,8	5485,5
18,433696	4244	5483,4
18,447026	4238,9	5476,8
18,460346	4216,1	5486
18,473662	4227,1	5502
18,486992	4244,3	5474
18,500317	4249,9	5494
18,513638	4263,2	5514,1
18,526954	4300,6	5496,1
18,540275	4297,9	5485,1
18,5536	4297,3	5477,1
18,566925	4282,6	5485,1
18,580241	4254,7	5483,2
18,593562	4259,2	5498,2
18,606883	4245,3	5517,5
18,620108	4240,2	5526,6

18,633428	4271,3	5531
18,646745	4261,2	5497,5
18,660066	4252,2	5486,6
18,673378	4252,2	5512,2
18,686694	4236,7	5501,3
18,700014	4241,7	5509,7
18,713344	4222,8	5518,4
18,726665	4215	5546,3
18,739985	4195,6	5555,9
18,753302	4171,8	5569,1
18,766627	4178,9	5614,8
18,779947	4193,3	5628,4
18,793268	4212,9	5606
18,80658	4205,2	5612,6
18,819905	4205,2	5613,8
18,833226	4207,3	5609,2
18,846542	4212,3	5600,1
18,859863	4226,7	5617
18,873175	4245,7	5593,9
18,886496	4249,4	5627,5
18,899808	4254,5	5640,4
18,913137	4284	5664,7
18,926466	4249,5	5672,5
18,939791	4232,7	5675,9
18,953108	4231	5677
18,966424	4207,3	5682,9
18,979749	4216,7	5661,8
18,99307	4209,6	5672,6
19,006382	4218,1	5671,2
19,019703	4198,5	5652,3
19,033027	4213	5637,9
19,046353	4234,1	5615,8
19,059669	4239	5603
19,072989	4229	5609,6
19,086319	4228,9	5595,7
19,099639	4198,9	5580,9
19,11296	4168,5	5583,1
19,126285	4161,9	5571
19,13961	4171	5532,9
19,152931	4138,4	5543,1
19,166252	4143,9	5553
19,179577	4155	5552
19,192898	4178,2	5538,4
19,206214	4211	5520,7
19,219526	4219,3	5507,2
19,232851	4213,2	5500,9
19,246171	4217	5490,7
19,259488	4213,7	5491,8
19,2728	4213,7	5489,9
19,286129	4219,2	5507,8
19,29945	4223	5522,3
19,312762	4215,4	5525,2

19,326083	4197	5549,3
19,339403	4209,3	5539,3
19,35272	4236,4	5551,3
19,366036	4225,8	5559,3
19,379348	4215,7	5567
19,392418	4229,6	5564,2
19,405734	4224,5	5545,9
19,419054	4238,9	5545,8
19,432366	4233,9	5570,9
19,445691	4250,1	5574,2
19,459017	4237,3	5567,9
19,472342	4246,7	5552,1
19,485658	4247,2	5546,9
19,498983	4245,6	5534,7
19,512308	4254,6	5555,4
19,525633	4266,8	5561,2
19,538949	4275,2	5570,1
19,552261	4278	5596,3
19,565586	4289,5	5612
19,578911	4274,5	5621,9
19,592033	4276,2	5589,5
19,605362	4275,6	5591,7
19,618687	4281,9	5567,8
19,632004	4284,6	5554,1
19,645333	4274,5	5562,2
19,658662	4291,3	5559,5
19,671979	4293,1	5578
19,685295	4286,5	5566,5
19,69862	4267,1	5560,3
19,711945	4262,6	5542,8
19,72527	4260,1	5543,1
19,738586	4271,2	5533,2
19,751907	4285,7	5528
19,765223	4285,7	5538,6
19,778548	4298,4	5530,2
19,791861	4296,3	5522,5
19,805181	4275	5514,6
19,818506	4278,3	5505,2
19,831827	4283,5	5502,2
19,845147	4274,5	5499,6
19,858473	4253,2	5502,1
19,871793	4220,4	5480,1
19,88511	4190,4	5465,8
19,89843	4207,7	5445,8
19,91176	4201,6	5429,7
19,925085	4198,8	5441,8
19,938401	4200,5	5451,8
19,951722	4206	5476,7
19,965038	4219,8	5484,4
19,978363	4212	5447,8
19,991679	4205,9	5438,6
20,004996	4204,8	5432

20,018317	4153,9	5433,1
20,031642	4129	5423,2
20,044962	4119,1	5404,8
20,058274	4121,9	5405,9
20,071595	4106,5	5390,5
20,084911	4097,7	5378,7
20,098228	4093,8	5386,5
20,111544	4081,1	5395,6
20,12486	4063,5	5400,4
20,138177	4045,3	5389,2
20,151502	4040,9	5386,8
20,164827	4052,8	5369,4
20,178148	4032	5358,3
20,191152	4034,1	5336,6
20,204468	4046,1	5321,4
20,217797	4058	5320,9
20,231123	4074,5	5312,5
20,244435	4066,8	5303,7
20,25776	4096,4	5311,6
20,27108	4124,5	5305,3
20,284405	4133,4	5325
20,297722	4133,3	5333,8
20,311034	4140	5351,2
20,324359	4146	5357,1
20,337684	4157,1	5373,6
20,351004	4163,1	5364,8
20,364316	4163,7	5366,6
20,377637	4168,7	5355,1
20,390958	4178,2	5381,7
20,404279	4164,4	5388,2
20,417599	4166,2	5378,3
20,43092	4150,2	5377
20,444241	4132,1	5368,2
20,457566	4122,7	5356,1
20,470886	4129,2	5358,6
20,484207	4108,9	5357,7
20,497528	4116	5357,3
20,510844	4112,1	5358,7
20,524169	4090	5372,9
20,537486	4071,3	5370,4
20,550806	4047,3	5381,7
20,564127	4043,5	5377,7
20,577456	4052,2	5371,6
20,590777	4056,5	5379,2
20,604098	4062	5372,7
20,617422	4082,1	5371
20,630752	4105,6	5359,9
20,644073	4143,9	5358,6
20,657389	4159,3	5357,9
20,670701	4158,2	5351,6
20,684026	4167	5365,8
20,697351	4152,7	5380,3

20,710672	4147,7	5362,7
20,723997	4159,7	5354
20,737322	4142,1	5356,9
20,750643	4137,2	5361,5
20,763963	4116,3	5372,9
20,777284	4118	5376,9
20,790604	4125,7	5374,9
20,803925	4142,5	5371,3
20,817242	4138	5371,7
20,830567	4171	5380,8
20,843887	4180,4	5394,5
20,857208	4202,3	5397,8
20,870524	4201,7	5395,7
20,883845	4214,4	5407,3
20,897161	4203	5406,2
20,910478	4210,7	5418,9
20,923803	4198,1	5426,9
20,937128	4203,7	5435,8
20,950444	4207,7	5452,5
20,963756	4228,2	5466
20,977072	4232,5	5469
20,990389	4223,7	5463,2
21,003705	4223,1	5461
21,01703	4225,9	5444,8
21,030347	4229,2	5423,4
21,043663	4245,2	5424,4
21,056975	4250,2	5425,2
21,0703	4251,3	5436,1
21,083629	4241,1	5445,3
21,09695	4253,4	5443,9
21,110271	4253,7	5431
21,123591	4256,5	5428,7
21,136912	4239,3	5434,1
21,150229	4241	5441
21,163541	4223,8	5467
21,176866	4221,7	5475,4
21,190182	4199,7	5487,2
21,203502	4200,2	5502,6
21,216823	4195,7	5516,9
21,230148	4189,6	5504,3
21,243465	4206,7	5527,5
21,256785	4196,2	5506,2
21,27011	4195,1	5507,8
21,283436	4200,1	5484,4
21,296752	4213,4	5455
21,310064	4213,9	5466,3
21,323393	4221,7	5476,5
21,336718	4215,8	5463,2
21,350039	4210,3	5447,5
21,363351	4189,7	5437,6
21,376672	4179,3	5447,3
21,389992	4186,5	5429,4

21,403313	4190,3	5428,7
21,416638	4181,6	5445,5
21,429963	4166,3	5457,9
21,443288	4144,9	5442
21,456604	4138,2	5426
21,469929	4104,2	5433,9
21,483246	4105,4	5423,4
21,496562	4095,5	5416,5
21,509879	4082,3	5438,8
21,523204	4078,9	5428,4
21,536533	4068,9	5424,4
21,549854	4068,2	5416,3
21,56317	4064,9	5406,1
21,576491	4033,3	5384
21,58982	4064,3	5366,8
21,602837	4068,2	5344,3
21,616154	4089	5338,2
21,629474	4096,1	5331,8
21,642795	4082,9	5320,5
21,656124	4075,3	5333,8
21,669445	4070,4	5326,5
21,682761	4075,8	5338,5
21,696082	4096,4	5335,8
21,709407	4108	5340,2
21,722728	4102,6	5343,9
21,73604	4111,2	5342,5
21,749356	4096,5	5341,9
21,762677	4072	5326
21,776002	4045,2	5315,8
21,789314	4020,7	5320,8
21,802636	4015,7	5325,5
21,815952	4017,1	5340,7
21,829264	4028,9	5333
21,842581	4033,2	5326,5
21,855897	4030,4	5311
21,869218	4026,5	5300
21,882534	4016,7	5285,5
21,89585	4013,4	5275
21,909158	4017,7	5262,3
21,922483	4008,3	5264,6
21,935812	4009,9	5288,8
21,949129	4016,4	5288,6
21,962454	4014,7	5281,4
21,975774	4009,7	5294,1
21,989095	3986,9	5297,1
22,002411	3974,5	5297,9
22,015724	3967	5267,6
22,02904	3968,6	5256,7
22,042356	3984,3	5251,4
22,055673	3987	5242,3
22,068993	3985,4	5236,2
22,08231	3986	5245,6

22,095622	4000,2	5250,2
22,108947	4005,1	5245,2
22,122272	4014,3	5231,3
22,135593	4019,2	5227,2
22,148909	3986,1	5232,8
22,162221	3980,7	5231,3
22,175546	3970,4	5214,8
22,188866	3962,8	5208,6
22,202191	3956,3	5203,4
22,215512	3950,2	5213,5
22,228837	3943,3	5200,8
22,242162	3956,8	5184,9
22,255483	3956,8	5196,6
22,268799	3967,5	5183,6
22,282116	3955,1	5176,1
22,295436	3958,3	5188,5
22,308757	3958,8	5188,5
22,322073	3939,2	5186,9
22,335398	3947,9	5165,7
22,348715	3960,8	5170,1
22,362031	3966,3	5179
22,375361	3957,6	5178
22,388681	3948,9	5181,3
22,401993	3946,2	5192,9
22,415305	3944,5	5200,5
22,42863	3919,2	5192
22,441951	3904,7	5236,7
22,455276	3907,3	5240,1
22,468597	3922,9	5268,3
22,481917	3907,3	5288,2
22,495234	3909,5	5282,6
22,50855	3915,9	5280
22,521875	3898,2	5289
22,535191	3907,3	5317,9
22,548508	3910	5302,4
22,561828	3915,8	5297,7
22,575154	3885,7	5288,3
22,588479	3855,6	5296,3
22,601795	3847	5312,6
22,615107	3821,8	5297,9
22,628436	3830,4	5289,3
22,641757	3827,7	5290,8
22,655078	3839,4	5284,8
22,668394	3865,2	5306,8
22,681715	3870	5298,8
22,695035	3882,9	5322,6
22,70836	3873,8	5316,9
22,721681	3855	5326,8
22,734997	3844,3	5322,2
22,748314	3837,9	5308,6
22,761639	3846	5334,6
22,774964	3864,8	5337,1

22,788284	3884,1	5339
22,801601	3890	5360,4
22,814917	3893,6	5354,3
22,828247	3909,2	5359,4
22,841572	3897,3	5375
22,854888	3897,9	5370,1
22,8682	3883,9	5357,5
22,881525	3900	5334,1
22,89485	3907	5340,2
22,908162	3902,1	5361,5
22,921474	3901,6	5351,1
22,934799	3893,5	5345,3
22,94812	3890,2	5336,7
22,961441	3890,1	5310,8
22,974757	3878,4	5296
22,988073	3898,7	5299,6
23,001394	3895	5295,6
23,014715	3928,3	5288,1
23,027927	3917,5	5284,8
23,041248	3924,5	5293,1
23,054577	3924,4	5289,6
23,067893	3908,9	5290
23,081106	3902,4	5295,4
23,094431	3902,4	5287,8
23,107756	3885,7	5301,3
23,121072	3888,3	5317,5
23,134393	3902,3	5323,3
23,147714	3897,4	5325,4
23,161047	3895,3	5353,5
23,174372	3882,4	5353,2
23,187684	3887,8	5330,5
23,201005	3875,5	5313,1
23,214334	3870,1	5302,8
23,227664	3862,1	5315,6
23,24098	3870,6	5326,5
23,254296	3872,9	5318,2
23,267617	3854,1	5322,2
23,280938	3833,8	5304,9
23,294258	3826,4	5285,5
23,307579	3799,7	5278
23,320904	3793,8	5270,8
23,334225	3802,3	5275,9
23,347546	3814,6	5271,6
23,360858	3818,3	5267,6
23,374178	3818,3	5282,3
23,387499	3822,1	5284
23,400807	3827,4	5268,9
23,414127	3822,6	5263,5
23,427444	3826,4	5310,2
23,44076	3823,6	5305,8
23,454085	3793,2	5321,4
23,467406	3778,3	5304,2

23,480726	3760,7	5297,4
23,494047	3759,6	5284,1
23,507372	3762,3	5271,9
23,520701	3758,1	5268,1
23,534026	3743,2	5252,7
23,547339	3724,6	5255,1
23,560655	3720,3	5262,5
23,573984	3738,4	5273
23,587301	3735,2	5289,8
23,600621	3752,8	5311
23,613942	3741,1	5315
23,627263	3723,5	5312,9
23,640583	3693,3	5310,4
23,653904	3689	5325,2
23,667229	3691,7	5351
23,68055	3695,9	5361,2
23,693866	3684,8	5366,6
23,707182	3694,3	5396,5
23,720503	3700,2	5428,3
23,733819	3705,5	5422,5
23,747136	3716,6	5395,4
23,760461	3717,2	5401,2
23,773786	3705,5	5432,6
23,787111	3699,1	5448,9
23,800432	3697	5444,6
23,813748	3691,7	5380,3
23,826757	3689	5362,7
23,840086	3689,6	5369,9
23,853407	3680,6	5382,1
23,866723	3683,2	5371
23,880053	3684,8	5398,3
23,893377	3683,7	5389,3
23,906698	3706	5390
23,919945	3703,9	5379,6
23,933262	3712,9	5375,2
23,946578	3702,9	5367,6
23,959894	3696,5	5368,4
23,973219	3700,2	5385,4
23,986544	3707,6	5389,7
23,999865	3712,9	5395,8
24,013186	3697	5421,1
24,026506	3706,6	5426,5
24,039827	3717,2	5444,3
24,053148	3717,2	5441
24,06646	3708,2	5432,7
24,079776	3694,9	5462,4
24,093101	3696,5	5439,9
24,106417	3693,8	5412,1
24,119734	3702,9	5408,9
24,133054	3697	5391,6
24,146367	3688	5373,6
24,159687	3689,1	5341,6

24,173008	3672,1	5314,7
24,186324	3650,9	5286,7
24,199645	3650,4	5306,1
24,212957	3664,7	5282,1
24,226282	3662	5261,7
24,239598	3645	5272,4
24,252915	3646,6	5301,9
24,266236	3643,9	5322,7
24,279552	3629,1	5346,3
24,292877	3628,6	5349,6
24,306202	3619	5329,9
24,319518	3632,8	5303,7
24,332834	3620,1	5320,1
24,346155	3643,9	5310,1
24,359485	3642,3	5315,4
24,372805	3641,8	5323,3
24,386122	3649,2	5309,6
24,399438	3645,5	5322,9
24,412754	3643,9	5349,4
24,426084	3643,4	5352
24,439404	3652,4	5350,2
24,452721	3666,2	5371,7
24,466046	3672,1	5356,9
24,479371	3675,8	5372,8
24,492691	3681,6	5375
24,506004	3692,3	5378,6
24,51932	3705	5403,4
24,532645	3705,5	5411,7
24,545675	3735,4	5408,4
24,558992	3751,9	5424,7
24,572312	3753,5	5439,5
24,585624	3757,8	5448,2
24,598945	3746,6	5454,4
24,612261	3753	5456,1
24,625573	3745	5442,4
24,638903	3743,4	5443,9
24,652223	3728,4	5454
24,665544	3718,9	5446,4
24,678865	3687,5	5468,9
24,69219	3667,3	5460,9
24,705506	3634,4	5445,4
24,718823	3639,2	5438,1
24,732143	3636,5	5459,8
24,74546	3636,5	5479
24,758776	3656,7	5475,1
24,77211	3648,7	5494,3
24,785435	3648,2	5500,4
24,798755	3644	5493,6
24,812072	3646,1	5486,3
24,825401	3645	5499,7
24,838726	3675,8	5495,7
24,852042	3674,7	5490,7

24,865359	3677,9	5480,9
24,878688	3685,3	5475,8
24,892013	3674,2	5458,1
24,905334	3670,5	5436,4
24,918646	3659,4	5443,3
24,931967	3654,1	5445,1
24,945292	3670	5436,4
24,958612	3666,3	5422
24,971933	3668,4	5438,6
24,985254	3649,8	5427,1
24,998574	3667,8	5423,4
25,011895	3655,7	5389,6
25,025216	3644	5389,2
25,038536	3654,1	5370,1
25,051857	3644	5392,5
25,065178	3631,3	5410,8
25,078499	3626,6	5392,8
25,091815	3634,5	5410,5
25,105131	3621,8	5411,9
25,118452	3625	5393,5
25,131768	3626,6	5399,6
25,145089	3627,6	5410,1
25,15841	3612,8	5396
25,17173	3604,4	5393,5
25,185047	3591,2	5402,2
25,198372	3589,6	5398,6
25,211693	3613,9	5388,1
25,225009	3605,9	5440
25,238325	3595,4	5470,8
25,251646	3588	5499
25,264971	3580,1	5517,5
25,278296	3563,7	5528,7
25,291621	3553,2	5542,5
25,304937	3538,4	5512
25,318258	3540	5502,3
25,33157	3529,9	5510,2
25,344895	3523,6	5503,3
25,358211	3511,5	5527,6
25,371532	3484,7	5517,8
25,384857	3497,8	5513,5
25,398173	3513,6	5500,1
25,41149	3528,9	5492,5
25,424811	3540	5483,8
25,438131	3548,4	5466,8
25,451456	3562,6	5443,7
25,464772	3562,1	5443,7
25,478089	3562,1	5440,8
25,491418	3590,1	5438,6
25,504743	3598	5433,6
25,51806	3604,4	5442,6
25,531372	3620,2	5426,7
25,544693	3631,3	5428,5

25,558013	3635,6	5446,2
25,571338	3621,8	5443,3
25,58465	3585,9	5463,5
25,597975	3567,5	5474,7
25,611296	3548,5	5479,8
25,624621	3548,6	5483,8
25,637933	3526	5503,4
25,651254	3544,3	5500,8
25,664579	3529,6	5483,8
25,677904	3522,3	5448,4
25,691216	3529,1	5425,3
25,704532	3544,9	5427,4
25,717848	3555,9	5432,8
25,731178	3558,6	5436,1
25,744498	3553,3	5419,9
25,757819	3537,5	5414,5
25,771136	3550,1	5437,5
25,784461	3552,1	5428,9
25,797786	3545,3	5441,5
25,811102	3541,6	5414,5
25,824409	3537,9	5422,8
25,837734	3535,2	5428,2
25,851055	3523,2	5436,8
25,864376	3515,3	5435
25,877692	3513,7	5448,7
25,891013	3507,9	5453
25,904329	3513,2	5456,6
25,91765	3525,3	5488,4
25,930966	3532,1	5528,9
25,944287	3531,1	5543
25,957603	3536,4	5562,6
25,970924	3524,3	5565,9
25,984249	3524,3	5556,9
25,99757	3516,4	5562
26,010891	3504,8	5580,8
26,024211	3502,7	5595
26,037536	3495,4	5640,1
26,050861	3490,7	5638,3
26,064177	3485	5684,3
26,07749	3487,1	5690,6
26,090819	3476,6	5679,6
26,10414	3475,1	5689,1
26,117456	3469,8	5640,5
26,130777	3465,6	5658,4
26,144098	3432,6	5697,8
26,157418	3428,3	5708,7
26,170739	3432	5696,5
26,184064	3430,5	5741,5
26,197384	3440,5	5769
26,210709	3450,9	5787,3
26,22403	3441,5	5774,8
26,237342	3439,9	5722,8

26,250663	3436,3	5723,5
26,263984	3451,4	5721
26,277309	3472,9	5693,9
26,290634	3463	5692,1
26,30395	3467,2	5697,9
26,317266	3464	5695,6
26,330596	3460,8	5688,3
26,343925	3455,6	5717,5
26,357246	3453	5671,8
26,370566	3468,7	5645,2
26,383761	3462,9	5644

Supplementary Figure 6f **ECIS: OA vs RA FLS, 2nd run (resistance - ohm)**

time	OA-FLS	RA-FLS
0,00055028	2306,4	3136,8
0,01603889	2398,5	3381,9
0,02929861	2508	3627,8
0,04261917	2653,2	3867,5
0,05594	2834,3	4136,6
0,06926056	3011,6	4418,3
0,08257694	3217,8	4729,6
0,09589778	3412,6	5036,9
0,10921833	3623,2	5362,8
0,12253028	3824,5	5775,1
0,13584667	4040,6	6203,6
0,1488425	4246	6621,2
0,16216306	4461	7052,9
0,17547944	4688,9	7532,5
0,18879583	4927,2	7969,6
0,20211639	5190	8405,7
0,21543278	5438	8805,5
0,22840694	5612,8	9139,1
0,24171889	5837,3	9469,7
0,25503972	6023,9	9713,3
0,26837333	6194,1	9940,5
0,28169389	6313,1	10138,9
0,29501028	6427,7	10345
0,30833528	6533,5	10555,3
0,32166028	6624,4	10684,9
0,33473833	6704,4	10803,8
0,34806333	6791,8	10925,8
0,36138833	6859,7	11049,5
0,37471361	6948,4	11147,3
0,38803861	7034,8	11259
0,40136361	7113,2	11357,4
0,41468417	7183,2	11420,4
0,428005	7265	11444,8
0,44132556	7319,2	11483,9
0,45464639	7391	11496,7
0,46796694	7416,4	11523,6
0,48128778	7461,1	11564,8
0,49461806	7447,4	11619,5
0,50793861	7484,9	11645,4
0,521255	7484,1	11705,6
0,53457583	7498,8	11745,2
0,547905	7541,5	11822,3
0,56122583	7560,4	11855,3
0,57454639	7580,8	11870,6
0,58787139	7641,2	11899,8
0,60119222	7683,1	11973,4
0,61451278	7745,5	12011,7

0,62783778	7785,5	12001,2
0,64115417	7832,5	11961,2
0,65447056	7880,3	11970,8
0,66778694	7955,9	11972,9
0,68111194	8003,8	12000,6
0,69444139	8057	12056,2
0,70775778	8088,6	12060,9
0,72107417	8134,3	12066,1
0,73439028	8169,4	12080,4
0,74771111	8205,7	12081,4
0,7610275	8223,6	12099,9
0,77434806	8263,5	12144
0,78766444	8269	12163,4
0,80098944	8278,3	12170,9
0,81431444	8262	12181,1
0,82763083	8278,3	12206,2
0,84094722	8284,9	12190
0,85427222	8303,1	12222,3
0,86759306	8313,8	12214,3
0,88090583	8279,4	12267,2
0,89422667	8257	12289,9
0,90753861	8277,9	12279,2
0,92085083	8302,4	12288,6
0,93417139	8323	12277,4
0,94749194	8334,6	12295,4
0,96081278	8383,1	12329,6
0,97412917	8376,4	12397,4
0,98744556	8362,7	12380,3
1,0007619	8338,7	12353,8
1,0140825	8369,2	12375,8
1,0274042	8391,6	12377,4
1,0407292	8430,7	12360,3
1,0540542	8458,8	12381,8
1,0673661	8525,1	12395,5
1,0806869	8600,6	12393,8
1,0940033	8619,3	12392
1,1073283	8575,8	12351,8
1,1206447	8596,4	12313,6
1,1339567	8566,8	12332,3
1,1472686	8571,6	12384,3
1,1605936	8543,4	12411,6
1,17391	8548,3	12437,4
1,1872222	8539,5	12427,1
1,2005428	8532,2	12416,7
1,2138678	8541,5	12420,3
1,2271928	8530,3	12378,4
1,2405136	8513,6	12401,1
1,2538256	8506,8	12440
1,2671464	8507,7	12418,8
1,2804583	8501,9	12384,5
1,2937789	8516,2	12370,4
1,3071039	8503,2	12374,4

1,3204203	8492,4	12360,3
1,3337367	8560,2	12295,8
1,3470617	8536,1	12278,7
1,3603867	8540,1	12302,4
1,3737075	8551,7	12287,8
1,3870239	8508,6	12240,5
1,4003403	8474	12212,4
1,4136653	8481,6	12196,8
1,4269817	8496,6	12240,5
1,4402936	8446	12253
1,4536056	8421,4	12226,2
1,4669219	8431,7	12192,9
1,4802428	8461,5	12182,8
1,4935589	8416,8	12141,2
1,5068797	8442,1	12133,7
1,5201917	8440,9	12106,7
1,5335125	8444,4	12119,2
1,5468375	8440,1	12124,7
1,5601633	8446	12143,9
1,5734797	8441,4	12130,9
1,5868006	8404,9	12114,1
1,6001256	8375,5	12182,6
1,6134419	8418,9	12206,1
1,6267581	8376,1	12266,9
1,6400875	8344,9	12215,2
1,6534125	8300,2	12179
1,6667289	8271,9	12140,4
1,6800408	8215,1	12131,5
1,6933617	8201,7	12116,4
1,7066781	8258,5	12091,4
1,7199944	8247	12093,8
1,7333064	8204,7	12056,3
1,7466269	8232,4	12015,7
1,7599433	8269,1	12007,6
1,7732683	8286,1	11948,1
1,7865806	8299,1	11986,7
1,7998969	8236,9	11941,4
1,8132219	8232,8	11854,3
1,8265469	8203,1	11804,2
1,8398675	8212,3	11761,1
1,8531839	8201,6	11751,7
1,8665047	8133,5	11781,3
1,8798167	8116,3	11728,4
1,8931461	8133,6	11749,7
1,9064622	8149,8	11725,7
1,9197786	8151,2	11688,5
1,933095	8122,1	11676,5
1,94642	8158,4	11670
1,9597408	8126,8	11533,6
1,9730528	8056,6	11456,7
1,9863778	8053,8	11368,5
1,9997028	8014,8	11393

2,0130278	7995,4	11367,4
2,0263486	7944,4	11295,7
2,0396692	7919,6	11316,9
2,0529942	7893,3	11321
2,0663192	7882,1	11323
2,0796444	7861,8	11388,4
2,0929617	7809,2	11384,2
2,1062781	7830,3	11326,8
2,1195944	7859,5	11266,1
2,1329194	7859,7	11330,2
2,1462358	7838,3	11291,9
2,1595478	7833,5	11349,2
2,1728728	7806,2	11323,1
2,1861933	7818,9	11216,6
2,1995142	7819,3	11102,5
2,2128347	7816,1	11105,2
2,2261556	7793,1	11091,6
2,2394761	7769,4	11095,6
2,2527969	7731,4	11068,1
2,2661219	7683,7	11071,3
2,2794469	7670,9	11153,2
2,2927678	7628,8	11134,7
2,3060883	7599,7	11128,7
2,3194133	7613,7	11176
2,3327297	7662	11156,5
2,3460461	7653,8	11014,3
2,3593625	7637,9	10974,9
2,3726917	7642,7	10911,4
2,3860081	7623,8	10891,3
2,3993244	7606	10897
2,4126453	7577,4	10818,4
2,4259658	7597,3	10833,7
2,4392867	7606,7	10834,8
2,4526072	7560,6	10893,5
2,4659192	7607,7	10854,9
2,47924	7608,9	10927,7
2,492565	7591,6	10895,8
2,5058814	7524,3	10891,6
2,5191933	7508,9	10833,5
2,5325142	7462,1	10780,3
2,5458306	7390,6	10721,3
2,5591556	7351,3	10620
2,5724719	7332,8	10658,9
2,5857925	7313,3	10650,2
2,5991175	7312,8	10585,6
2,6124425	7296,7	10498,4
2,6257544	7205,2	10533,3
2,6390753	7171,3	10547,5
2,6523958	7143,6	10522,7
2,6657211	7112	10465
2,6790417	7084,2	10396,6
2,6923581	7115,1	10297,7

2,70567	7160	10214,3
2,7189864	7216	10249,8
2,7323114	7282,5	10297,4
2,7456322	7248,6	10313,6
2,7589528	7265,2	10312,3
2,7722647	7278,6	10311,6
2,7855897	7315,8	10311,6
2,7988811	7288,4	10286,5
2,8122019	7275,9	10285,5
2,8255225	7280,7	10226,7
2,8388389	7255,9	10151,6
2,8521508	7212,8	10165,4
2,8654803	7151,8	10213,6
2,8788011	7160,1	10120,8
2,8921131	7190,7	10032,5
2,9054381	7160,8	9990,1
2,9187631	7130	9993,4
2,9320836	7118,9	9961,3
2,9454044	7109,2	9955,7
2,9587164	7086,7	9953,6
2,9720414	7068,9	9866,6
2,9853664	7036,5	9780,1
2,9986828	7014,1	9766,8
3,0120036	7012,8	9713,1
3,02532	7001,9	9748,7
3,0386364	6979,8	9637,2
3,0519569	6957,1	9679,4
3,0652819	6910,6	9587,2
3,0785983	6911,9	9472,6
3,0919189	6896,5	9482,2
3,1052442	6837,9	9438,7
3,1185647	6840,2	9395,7
3,1318856	6863	9353,2
3,1452017	6845,8	9313
3,1585139	6837,7	9193,5
3,1718389	6773,8	9180,9
3,1851639	6783,3	9133,8
3,1984803	6809,8	9064
3,2118053	6795,4	8974,4
3,2251347	6768,3	8907,8
3,2384508	6780,6	9008,8
3,2517717	6787,7	9056,6
3,2650933	6800,3	9015,4
3,2784139	6833,6	9009,3
3,2917303	6850,6	9068,8
3,3050467	6888,9	9023,4
3,3183717	6824	8971,8
3,3316925	6803	8958,6
3,3450131	6837,7	8960,1
3,3583381	6834,5	8962,1
3,3716631	6750,9	8927,6
3,3849794	6740,7	8885,3

3,3982914	6673,2	8879,9
3,4116122	6663,5	8854,9
3,4249372	6623,6	8796,7
3,4382622	6640,5	8768,5
3,4515831	6604,6	8768
3,464895	6551,8	8796,5
3,4782114	6543,8	8814,2
3,4915278	6507,7	8815,5
3,5045581	6519,1	8834,6
3,5178875	6520,2	8817,4
3,5312125	6498,9	8766,3
3,5445375	6497,7	8741,7
3,5578539	6479,9	8687,8
3,5711744	6465,6	8739
3,5844908	6426,1	8782,5
3,5978117	6351,6	8712,3
3,6111278	6364,4	8703
3,6244486	6372,5	8711,5
3,6377736	6359,6	8699,2
3,6510942	6389,5	8657,2
3,6644106	6360,4	8621,6
3,6777228	6352,4	8612,3
3,6910433	6311,9	8601,7
3,7043597	6264,7	8638,1
3,7176847	6220,9	8607,1
3,7310056	6135,8	8533,5
3,7443217	6133,8	8520,2
3,7576425	6121,1	8477,2
3,7709544	6132,5	8362,2
3,7842753	6113,1	8340,1
3,7975958	6109	8347
3,8109122	6122,4	8383,8
3,8242331	6134,5	8391,6
3,8375581	6162	8435,3
3,8508786	6149,3	8377,3
3,8642036	6181,5	8265,9
3,8775244	6240,2	8279,7
3,890845	6243,6	8208,3
3,9041658	6271,2	8216,3
3,9174733	6286	8267,7
3,9307942	6275,2	8258,2
3,9441147	6290,1	8260,8
3,957445	6257,6	8267,7
3,9707658	6220,5	8175,3
3,9840822	6190,2	8109,7
3,9973942	6225,2	8078,5
4,010715	6186,1	8070,9
4,02404	6180,8	8084,3
4,0373606	6167,3	8036,8
4,0506822	6193,5	8047,4
4,0640031	6211	8042,8
4,0773192	6190,2	7993,4

4,09064	6195,5	7975,1
4,1039606	6224,3	8006,3
4,1172728	6214,9	7999,3
4,1304114	6217,5	7970,2
4,1437278	6195,4	7978,9
4,1570572	6227	7927,2
4,1703778	6224,4	7921,2
4,1836897	6176,7	7874,2
4,1970147	6203,6	7818,5
4,2103311	6195,6	7805,1
4,2236561	6192,9	7779,8
4,2369811	6169,3	7804,7
4,2503019	6178,7	7760,9
4,2636183	6173,9	7742,4
4,2769347	6155,1	7731,2
4,2902511	6124,4	7728,3
4,3035847	6101	7728,9
4,3168967	6067	7730,9
4,3302217	6035	7738,1
4,3435467	5990,4	7766,2
4,3568675	5972,5	7725,9
4,3701839	5970,5	7725,5
4,3835044	6002,4	7707,4
4,3965219	6008,4	7700,8
4,4098425	5961,2	7729,7
4,4231633	5912,8	7684
4,4364839	5895	7668,1
4,4498089	5875,1	7629,7
4,4631253	5881,1	7667,2
4,4764383	5862,5	7616,2
4,4897547	5893,3	7648,8
4,5030883	5870,6	7689
4,5164178	5861,4	7682,7
4,5297342	5868,1	7749,8
4,5430503	5861	7722,6
4,5563667	5867,7	7770,6
4,5696917	5912,1	7763,8
4,5830167	5928,7	7727,3
4,5963331	5935,7	7676,4
4,6096453	5948,6	7625,2
4,6229703	5971,7	7595,7
4,6362953	6000,9	7540,4
4,6494208	5980,9	7494,9
4,6627417	6013,6	7563,6
4,6760536	6064,6	7583,7
4,6893786	6127,9	7509,4
4,7026994	6137,3	7431,1
4,71602	6139,3	7324,3
4,7293364	6133,2	7225,2
4,7426658	6104,8	7225,7
4,7557092	6089,7	7203,8
4,7690256	6027	7172,6

4,7823506	6015	7061
4,7956711	6032,3	7004,2
4,8087925	6003,7	7022,2
4,8221133	5994,4	6944,5
4,8354253	6024,3	6878,6
4,8487511	6016,3	6882,9
4,8620719	6002,9	6849,2
4,8753969	6022,7	6836,8
4,8887175	6016,2	6847
4,9020383	5998,9	6861,5
4,9153633	5982,3	6823,3
4,9286842	6003,4	6838,2
4,9420047	5992,9	6829,7
4,9553297	5953,8	6824,8
4,9686506	5931,9	6785,9
4,9819711	5806,3	6790,1
4,9952919	5787,6	6795,9
5,0086039	5763,8	6765,2
5,0219289	5678,5	6730,1
5,0352539	5654,5	6683,7
5,0485744	5713,6	6646,6
5,0618908	5727,2	6593,2
5,0752072	5736,3	6581
5,0885194	5751,4	6473
5,10184	5776,8	6403,1
5,1151608	5808,2	6434,9
5,1284814	5832,3	6425,5
5,1417892	5852,7	6341
5,1551097	5858,6	6267,2
5,1684306	5844,8	6215,6
5,1817511	5867,1	6146,9
5,1950806	5930,2	6123,2
5,2083969	5931,5	6104,3
5,2217133	5932,1	6063
5,2350383	5928,1	6027,8
5,24806	5933,2	6017,2
5,2613817	5930,8	5980,5
5,2746981	5904,6	5889
5,28801	5867,8	5848,2
5,301335	5825,1	5779,3
5,31466	5770,2	5768,2
5,3279764	5737,6	5835,2
5,34131	5696	5870,2
5,354635	5718,1	5898,5
5,36796	5703,8	5969,9
5,3812764	5742,2	6046,6
5,3945928	5788,4	6088,1
5,4079136	5800	6160
5,4212428	5776,4	6211,7
5,4345636	5752,4	6340,8
5,44788	5710,8	6458,9
5,461205	5679,7	6572,4

5,4745256	5686,2	6682,8
5,4878419	5676,5	6758,7
5,5011628	5670,7	6797,8
5,5144878	5662,9	6845,4
5,5278083	5642,2	6888,9
5,5411247	5670,7	6892,2
5,5544411	5658,4	6937,4
5,5677669	5605,3	6926,3
5,5810886	5668,8	6941,8
5,5944094	5729,8	7048,7
5,6077344	5800,7	7060,4
5,6210594	5821,6	7022
5,6343803	5809,8	7003,3
5,6476922	5837,3	7043,7
5,6610172	5810,6	7064,8
5,6743344	5804,7	7021,7
5,6876508	5832,1	6978,7
5,7009758	5802,7	7004,3
5,7142967	5821	6949,1
5,7276172	5819,6	6948,7
5,7409381	5878,9	6965,3
5,7542672	5970,8	6939,8
5,7675836	6040	6893,3
5,7809044	6108	6855,4
5,7942208	6115,8	6867,6
5,8075414	6133	6849,3
5,8208664	6064	6856
5,8341828	6011,9	6834,6
5,8475022	5965,2	6821,7
5,8608208	5951,4	6775
5,8741372	5919,3	6768,9
5,8874622	5853,3	6773,2
5,9007917	5792,6	6747,6
5,9141122	5780,9	6753,3
5,9274286	5736,7	6772,7
5,9407581	5719,8	6776,8
5,9540831	5719,7	6764,7
5,9673994	5728,7	6774,5
5,98072	5788	6773,6
5,9940492	5826	6808,1
6,0073711	5890,7	6825,7
6,0206917	5874,2	6757,9
6,0340125	5897	6751,4
6,0473375	5894,6	6718
6,0606625	5883,5	6738,3
6,0739744	5907,1	6708,2
6,0872953	5918,3	6720,2
6,1006158	5916,8	6712,9
6,1139322	5915,8	6703,5
6,1272486	5900,1	6719,1
6,1405736	5930,5	6701,8
6,15389	5953,3	6728,2

6,1672064	5945,4	6754,8
6,1805183	5977,6	6785,4
6,1938389	5962,6	6777,2
6,2071597	5971,8	6707,9
6,2204847	5956,9	6718,3
6,2338097	5933,7	6714,8
6,2471392	5946,8	6743,4
6,2604597	5916,5	6774,9
6,2737806	5918,4	6751,3
6,2871056	5955,6	6785,6
6,3004306	5895,3	6811,9
6,3137511	5874,5	6795,4
6,3270589	5827,5	6753
6,3403797	5807,5	6766,3
6,3537014	5822,1	6772,1
6,3670142	5826,9	6800
6,3803436	5830,2	6745,3
6,3936642	5842,2	6768,1
6,4069806	5829,1	6819,3
6,4203056	5779,9	6837,2
6,4336264	5723,6	6823,4
6,4469469	5722	6879,9
6,4602719	5725,9	6885,2
6,4735928	5760,7	6889,1
6,4869047	5760	6940
6,5002297	5743,1	6905
6,5135506	5749,6	6895,1
6,5268756	5805,4	6910
6,5401919	5837	6922,3
6,5535083	5853,6	6949,4
6,5668289	5807,4	6924,9
6,5801583	5789,1	6945,8
6,5934747	5746,5	7083,2
6,6067953	5759,8	7266,4
6,6200997	5751,8	7338,6
6,6334117	5835,4	7257,4
6,6467281	5902,6	7189,5
6,6600531	5931,8	7073
6,6733736	5891,7	6909,3
6,6866858	5875,5	6737
6,7000064	5824,2	6624,6
6,7133314	5771,1	6530,1
6,7266564	5774,3	6503,3
6,7399728	5761,1	6506,6
6,7532936	5744,9	6529,9
6,7666228	5752,8	6543,9
6,7799481	5781,2	6517,3
6,7932686	5789,3	6537,3
6,806585	5779	6562
6,8199014	5767,4	6621,7
6,8332219	5767,6	6705,6
6,8465469	5750,3	6743,2

6,8598633	5748,9	6758,8
6,8731842	5792,3	6787,3
6,8865006	5859,8	6870,3
6,8998256	5867,7	6956
6,9131461	5901,9	6971,6
6,9264581	5910,6	7024,6
6,9397744	5900	7040,5
6,9530908	5925,5	6996,6
6,9664158	5920,3	6994
6,9797367	5904,6	6966,9
6,9930617	5881,7	6995,9
7,0063781	5859,4	6977,8
7,0197031	5835,9	7041,7
7,0330236	5878,7	7035
7,0463314	5906,3	7037,3
7,0596519	5915,5	6990,3
7,0729772	5918,6	7041,9
7,0862933	5910,4	7066,1
7,0996142	5901,2	7048
7,1129436	5872,1	7027
7,1262686	5811,9	7025,1
7,139585	5787,8	6990,3
7,1528894	5764	6997,2
7,1662103	5755	6966,3
7,1795361	5737,1	6860
7,1928611	5736,9	6743
7,2061819	5779,8	6636,3
7,2195069	5828,1	6598,6
7,2328275	5863,3	6568,6
7,2461483	5876,1	6593,8
7,2594689	5831,4	6645,8
7,2727853	5823	6790,4
7,2861061	5877,1	6897,3
7,2994267	5944,8	7004,2
7,3127475	6041,5	7090,6
7,3260636	6107,2	7147,8
7,3393889	6124,1	7177,4
7,3527094	6138	7194
7,3660303	6127,9	7186,8
7,3793464	6078,9	7129,1
7,3926628	6018,1	7103,9
7,4059836	5916,5	7077,2
7,4193	5847,5	7027,6
7,432625	5744,2	6937,4
7,4459456	5637,9	6890,9
7,459275	5529,5	6855
7,4725956	5420,3	6890,6
7,4859164	5359,2	6874,9
7,4992414	5344,8	6890,5
7,5125619	5312,7	6841,8
7,5258783	5251,3	6879
7,5392033	5237,6	6954,3

7,5525286	5278,2	6963,4
7,5658536	5421	6961,9
7,5791742	5540,6	6954,4
7,5924861	5614,8	6943,3
7,6058111	5627,5	6935,5
7,6191364	5661,1	6950,7
7,6324525	5661,9	6940,3
7,6457778	5643	6945,1
7,6590983	5628,7	7008
7,6724189	5547,7	7126
7,6857147	5450,1	7207
7,6990397	5379,7	7233,6
7,7123614	5270	7262,2
7,7256864	5196,4	7295,5
7,7390069	5209,4	7292,4
7,7523278	5213,8	7320
7,7656397	5276,8	7225,6
7,7789606	5271,8	7191
7,7922811	5291,9	7134
7,8055931	5306,4	7086,2
7,8189094	5324,6	7141,3
7,8322344	5329,5	7284,5
7,8455553	5318,2	7413,5
7,8588717	5230,7	7538,2
7,8721967	5323	7535,5
7,8855217	5488,5	7579,1
7,8988381	5621,2	7493
7,9121631	5733,1	7464,1
7,9254836	5797,2	7395,7
7,9388131	5843,7	7339,3
7,952125	5847,6	7247,3
7,9654458	5828,8	7203,2
7,9787664	5817,3	7118
7,9920914	5744,5	7176,2
8,0054078	5705,6	7121,2
8,0187372	5615,7	7139,4
8,0320578	5569,4	7161,8
8,0453786	5580,8	7174,8
8,0586992	5621	7175,2
8,0720156	5673,4	7198,1
8,0853406	5678,4	7153,2
8,0986569	5676,5	7001,9
8,1119778	5688	6838,8
8,1252942	5707,8	6759
8,1386233	5672,8	6707,2
8,1519497	5630,6	6744,9
8,1652661	5585,3	6825,7
8,1785825	5556,3	6918,5
8,1919031	5587	7011,7
8,2052281	5703,3	7100
8,2185531	5834,6	7146,9
8,2318694	5974,8	7164,4

8,2451858	6028,7	7113,9
8,2585108	6033	7097,5
8,2718317	5979,9	7086,1
8,2851567	5979,5	7103,2
8,2984772	5912,4	7094,7
8,3118022	5821,3	7172,2
8,3251272	5735,8	7263
8,3384522	5725,6	7336,9
8,35177	5670,5	7343
8,3650864	5637,3	7359,4
8,3784028	5650,7	7400,5
8,3917233	5695,9	7455
8,4050442	5760,7	7485,2
8,4183647	5815,5	7435,2
8,4316853	5799,8	7421,8
8,4450061	5840,3	7382,8
8,4583267	5812,7	7363,4
8,4716475	5763,9	7344,7
8,4849681	5729,2	7343,2
8,4982803	5684,2	7312,5
8,5116053	5667,7	7301,5
8,5249303	5651	7288,1
8,5382508	5617	7309,5
8,5515631	5616,2	7304
8,5648836	5630,2	7341,9
8,5782086	5646,2	7357,6
8,5915306	5726,8	7387,4
8,6048514	5788,1	7376,2
8,6181775	5782,2	7406
8,6314983	5743,9	7366,3
8,6448147	5712,9	7370,4
8,6581267	5706,7	7407,3
8,6714517	5706,3	7459,7
8,6847811	5683,2	7482,6
8,6980972	5681,7	7431,3
8,7114181	5641,5	7425,4
8,7247386	5620,3	7397,6
8,7380681	5657,7	7383,2
8,7513931	5658,5	7369,5
8,7647094	5719,5	7356,8
8,7780214	5758,1	7296,2
8,7913422	5739	7256,9
8,8046672	5764	7156,4
8,8179878	5725,6	7078,6
8,8313086	5721,2	7013,9
8,8446336	5750,1	6932,1
8,8579586	5718,6	6906,7
8,8712836	5698,4	6904,5
8,8846	5672,9	6953
8,8979208	5656,5	7029,7
8,91125	5677,5	7052,5
8,9245708	5654,2	7096

8,9378872	5668,4	7137,5
8,9512122	5715,8	7170,8
8,9645328	5741,2	7083,2
8,9778536	5764,5	7066,9
8,99117	5807,2	7045,3
9,0044697	5812,2	7008,3
9,0177861	5778,6	6928
9,0311069	5740,1	6930,4
9,0444319	5696	6881,3
9,0577569	5635,4	6891,4
9,0710775	5580,5	6911,8
9,0844028	5622,6	6941,3
9,0977278	5649,4	6941,6
9,1110442	5682,2	6975,8
9,1243647	5686	6960,8
9,1376767	5721,2	6984,7
9,1509975	5776,1	6958,8
9,1643181	5783,5	6963,3
9,1776389	5764,4	6954,6
9,1909639	5766,9	6910,8
9,2042844	5759,3	6964,7
9,2176008	5738,4	6960,8
9,2309172	5730,4	6947,6
9,2442422	5690,5	6970,6
9,2575631	5677,1	6995,9
9,2708806	5670,2	7063,4
9,2842056	5657,1	7052,4
9,2975275	5657,8	7028,2
9,3108483	5661,3	6965,3
9,3241775	5658,8	6942,8
9,3374939	5623,7	6938
9,3508189	5640	6907,8
9,3641397	5638	6952,1
9,3774603	5664,7	7009,4
9,3907853	5717,6	7056,3
9,4041103	5853,3	7079,7
9,4174311	6009,1	7049,5
9,4307517	6100,2	7026
9,4440681	6112,4	7018,3
9,4573931	6067,3	6995,4
9,4707139	6042,9	6983,5
9,4840389	5970,3	6980,1
9,4970606	5877	6992,4
9,5103769	5816,4	6993,8
9,5236975	5766,6	7051,1
9,5370183	5774,7	7039,2
9,5503433	5734,7	7041,4
9,5636639	5717,7	6978,1
9,5769761	5671	6957,1
9,5902922	5624	6889,7
9,6036131	5576,4	6870,9
9,6169336	5561,2	6803,3

9,63025	5575,8	6850,9
9,6435708	5605,6	6889,9
9,6568925	5622,2	6910,4
9,6702131	5685,1	6912,3
9,6835381	5736,9	6960,5
9,6968589	5769	6989,2
9,7101794	5817,5	7003,1
9,7235003	5859,7	7074,6
9,7368208	5904,1	7110,3
9,7501458	5920	7096,3
9,7634622	5941,2	7134,3
9,7767744	5989,1	7117,9
9,7900906	6042	7112,5
9,80342	6012	7103,5
9,8167494	5995,9	7105,4
9,83007	5981,2	7139,2
9,8433864	5946,7	7168,1
9,8567028	5960,4	7147,1
9,8700278	5900,3	7110,6
9,8833528	5824,3	7113,4
9,8966647	5800,3	7069,2
9,9099769	5810,1	7055,3
9,9232975	5856,6	7074,4
9,9366139	5868,1	7027,8
9,9499356	5896,8	6960,8
9,9632606	5890,9	6901,1
9,9765769	5928,3	6935,7
9,9898933	5926,2	6970,7
10,003214	5930,3	6936,8
10,016535	5950,4	7057
10,029851	6006,3	7010,6
10,043163	6099,1	6982,2
10,056479	6125	6935,7
10,069809	6096,9	6924,8
10,083134	6089,6	6907,1
10,096112	6095,7	6916,8
10,109424	6028,1	6939
10,122745	5988,4	6995,9
10,136061	5939,2	7055,9
10,149392	5872,8	7069,7
10,162712	5893,9	7092,3
10,176029	5881,9	7120
10,189342	5853,3	7099,3
10,202658	5823,8	7059,5
10,215979	5842,1	7012,2
10,229304	5893,1	6883,3
10,24263	5935,9	6762,2
10,255951	5942,2	6701,6
10,269276	5943,3	6643,6
10,282592	6018,2	6622,9
10,295917	6081,9	6572,9
10,309238	6117,1	6552,7

10,322558	6161,8	6638,2
10,335879	6166,8	6668,4
10,349204	6133,5	6646,7
10,362525	6128,9	6647,2
10,375845	6150,9	6623,8
10,389157	6155,7	6601,8
10,402478	6166	6606
10,415799	6188,8	6628,9
10,429119	6199,1	6605,7
10,44244	6270	6599,2
10,455756	6264,2	6516,1
10,469069	6235,7	6434,1
10,482391	6201,9	6363,8
10,495708	6182,6	6323,9
10,509037	6148,2	6235,6
10,522358	6104,2	6179
10,535679	6075,8	6187,3
10,548995	6052,2	6218,6
10,562316	6064,1	6268,1
10,575641	6109,3	6292,4
10,588961	6128,8	6325,4
10,602282	6136,1	6357,6
10,615611	6156,7	6350,5
10,628933	6172,9	6348,5
10,642258	6156,1	6324,9
10,655583	6127,9	6283,6
10,668904	6122	6206,8
10,682216	6125,8	6125,1
10,695541	6189,2	6081,8
10,708874	6196,8	6073,5
10,722199	6196,7	6108,1
10,735516	6203,4	6095
10,748845	6247,4	6072,6
10,762166	6274,5	6122
10,775487	6281,2	6109,9
10,788803	6259,6	6174,4
10,802115	6274,4	6137,5
10,81544	6269,3	6172,7
10,828761	6228,7	6099,2
10,842081	6183,5	6109,2
10,855398	6145,2	6132
10,868723	6105,4	6174,5
10,882048	6020,6	6173,7
10,895368	5995,9	6134,8
10,908681	6005,1	6070,1
10,922002	6042,7	6090,9
10,935327	6094,3	6107,7
10,948644	6071	6012,2
10,96196	6053,3	5962,1
10,975281	6085,1	5931,2
10,988592	6149,4	5950,8
11,001913	6195,2	6006,8

11,015238	6197,7	6036,8
11,028555	6196,3	6010,9
11,04188	6179,8	6011,8
11,0552	6169,8	6016,1
11,068521	6169,9	6071,5
11,081846	6120,3	6072,7
11,095167	6127,8	6056,2
11,108488	6086,6	6060,9
11,121813	6067,9	5997,5
11,135126	6064,6	5945,4
11,148451	6010,6	5937,9
11,161772	6057,8	5967,9
11,175102	6144,3	5953
11,188418	6202,3	5918,4
11,201734	6225,4	5923,3
11,215046	6196,7	5961,2
11,228363	6129	5943
11,241684	6131,4	5942,4
11,255001	6115	5997,5
11,268326	6124,9	6003,8
11,281642	6098	6039,4
11,294963	6113	6035,8
11,308284	6141,8	6021,1
11,321604	6127,5	6020,4
11,334925	6158,9	6039,6
11,348241	6176,6	6025,8
11,361566	6186,8	6006,7
11,374887	6215,9	5962,2
11,388208	6245,9	5933,5
11,401524	6242	5953,3
11,414845	6235	5962,1
11,428166	6193,7	5979,5
11,441482	6147,2	6040,1
11,454804	6133	5991,5
11,468124	6097,9	6000,5
11,481445	6143,1	6030,7
11,494766	6166,9	6058
11,508091	6221,6	6066,7
11,521416	6203,5	6028,8
11,534737	6243,5	6046,5
11,548053	6217	5969,6
11,56137	6210,7	5936,9
11,574687	6173	5884
11,588003	6131,5	5903,4
11,601324	6117,7	5879,4
11,61464	6077	5884,2
11,627965	5999,1	5849,6
11,641286	6016,8	5832,1
11,654606	6084,1	5800,8
11,667927	6100,9	5753,6
11,681252	6082,4	5708,5
11,694577	6095,3	5728,1

11,707898	6083,1	5704,7
11,721223	6068,1	5701,1
11,734544	6089,6	5729
11,74786	6107,4	5719,5
11,761181	6052,7	5689,4
11,774497	6064	5681,8
11,787813	5958,6	5664,6
11,801138	5932,8	5674,6
11,81445	5896,2	5687,4
11,827763	5893,8	5647,3
11,841074	5877,8	5639,7
11,854395	5853,7	5580,5
11,867711	5905,8	5586
11,881036	5914,9	5582,9
11,894357	5925,7	5559
11,907682	5880,2	5563,3
11,921003	5838	5556,4
11,934328	5789,6	5548,6
11,947644	5771,9	5542,2
11,960965	5805,8	5538,6
11,974286	5857,3	5547,4
11,987612	5887,4	5547,4
12,000937	5899,4	5516
12,014253	5943,6	5506,3
12,027574	5937,5	5483,2
12,04089	5995,8	5467,9
12,05419	6008,7	5442,1
12,067502	5928	5408,4
12,080832	5946	5377,9
12,094152	5958	5371,3
12,107473	5939,6	5375,7
12,120794	5917,3	5414,2
12,134119	5843,1	5399,9
12,147445	5798,4	5426,2
12,160764	5858,6	5431,4
12,174088	5879,6	5406,5
12,187413	5836,8	5437,3
12,200724	5852,6	5446,7
12,214041	5820,1	5437,9
12,227361	5854,5	5429,3
12,240682	5871,6	5418,8
12,253999	5904,3	5440,6
12,267315	5854,1	5451,8
12,280636	5857,8	5463,5
12,293969	5754,5	5434,2
12,307299	5739,4	5442,3
12,320619	5742,4	5459,9
12,333936	5746,8	5502,2
12,347265	5760,2	5499,8
12,36059	5796,3	5490,2
12,373902	5824,4	5493,1
12,387214	5853,9	5501,1

12,400539	5854,9	5540,6
12,413864	5820,1	5588,7
12,427181	5815,4	5559,7
12,440497	5748,2	5576,6
12,453817	5746,7	5585,1
12,467147	5709,7	5591,9
12,480469	5672,6	5596,5
12,493794	5674,8	5611,4
12,50711	5707,1	5626,1
12,520422	5730,5	5543,4
12,533739	5779,6	5549,5
12,547056	5778,7	5539,4
12,560381	5768,8	5565,2
12,573697	5785,2	5590,4
12,587018	5777,7	5598,7
12,600334	5775,8	5614,5
12,613655	5752,9	5589,5
12,626976	5736,8	5531,3
12,640296	5755,8	5529,9
12,653613	5744,8	5525
12,666929	5730,6	5530,6
12,680254	5704,1	5552
12,693579	5713,2	5543
12,706896	5733,6	5536,9
12,720212	5668,9	5570,6
12,733533	5680,4	5541,7
12,746849	5648,9	5527,9
12,760165	5635,6	5489
12,77349	5677	5478,2
12,786811	5703,9	5436,2
12,800127	5733,4	5393,8
12,813454	5742,1	5388,4
12,826783	5792,8	5407,5
12,840099	5836,5	5376,4
12,85342	5802,3	5327,4
12,866741	5862,6	5284,9
12,880066	5831,9	5290,1
12,893391	5790,6	5309,1
12,906703	5780,7	5263,1
12,920015	5755,1	5333,1
12,933336	5736,7	5317,3
12,946652	5676	5300
12,959978	5600,9	5326,1
12,973303	5614,9	5360,7
12,986619	5660,2	5346,5
12,99994	5653,7	5323,4
13,013261	5691,5	5299,4
13,026581	5700,9	5333,7
13,039902	5754,9	5317,9
13,053223	5744,4	5311
13,066539	5786,4	5296,5
13,079864	5755,7	5287,1

13,093193	5721,7	5320,6
13,10651	5715,7	5286,5
13,119826	5723,8	5294,2
13,133151	5704,3	5276,1
13,146467	5730,8	5300,4
13,159785	5733,1	5321,7
13,173101	5655,5	5334,3
13,186413	5664,9	5311,7
13,199738	5766,3	5335,4
13,213059	5810,8	5303
13,226375	5844,9	5277,9
13,239696	5839,3	5275,4
13,253012	5871,4	5265,1
13,266324	5822	5263,4
13,279641	5842,5	5265,9
13,292961	5812,3	5259,9
13,30628	5764,5	5280,8
13,319605	5780,5	5303,9
13,332921	5763,1	5296
13,346242	5732,5	5323,4
13,359567	5717,6	5303,8
13,372896	5712,1	5306
13,386217	5701,9	5322,9
13,399529	5733,9	5341,3
13,412841	5751,9	5299
13,426161	5763,6	5304,1
13,439201	5791,7	5326,1
13,45253	5799,3	5313,1
13,465851	5730,9	5312,5
13,479176	5733,7	5277,4
13,492505	5727,1	5318,7
13,50583	5737,9	5278,7
13,519142	5786,3	5265
13,532467	5836,8	5271,1
13,545796	5875,7	5287,2
13,559117	5878,8	5306,5
13,572433	5887,7	5338,2
13,585758	5851,5	5362,9
13,599079	5838	5429,4
13,6124	5803,8	5418,7
13,625716	5837,7	5413
13,639033	5866,5	5375,6
13,652353	5871	5340,3
13,665674	5886,1	5290,6
13,678986	5943,4	5330,6
13,692311	5936,1	5329,1
13,705636	5928,7	5354,8
13,718957	5920,8	5331,6
13,732278	5930	5344
13,745589	5932,5	5360,3
13,758906	5921,6	5415,9
13,772231	5876,8	5367

13,785543	5858,9	5372,6
13,798868	5844,3	5387,2
13,812189	5806,6	5370,9
13,825509	5781,4	5397,5
13,838836	5770,8	5403,6
13,852156	5818,9	5394,8
13,865468	5826,7	5427,2
13,878793	5858,3	5435,1
13,892109	5867,5	5439,5
13,905422	5862,2	5419,4
13,918734	5809,8	5450,1
13,932063	5797	5446,4
13,945354	5825,4	5438,8
13,95868	5800,6	5424,1
13,972005	5799,4	5449,6
13,985325	5794	5383,4
13,998646	5837,8	5367,6
14,011971	5829,7	5335,6
14,025292	5797	5299,4
14,038601	5766,1	5280,6
14,05193	5781,6	5271,2
14,065251	5772	5229
14,078571	5739,5	5205,6
14,091896	5717	5197,2
14,105221	5695,9	5215,8
14,118542	5724,6	5198,5
14,131863	5720,6	5207,5
14,145184	5728,5	5206,5
14,158513	5776,2	5209,6
14,171834	5792	5246,1
14,185129	5818,6	5227
14,198446	5801,2	5238,9
14,211762	5811,4	5288,1
14,225087	5777,3	5291,2
14,238407	5757,9	5324,1
14,251728	5747,1	5333,7
14,265045	5707,6	5362,4
14,27837	5717,5	5361,2
14,291695	5686,7	5367,5
14,305011	5654,9	5391,7
14,318323	5650,3	5365,6
14,331644	5709,4	5362,6
14,34496	5755,5	5367,5
14,358285	5809,3	5341,3
14,371601	5795,9	5358,7
14,384918	5806,2	5376,8
14,398234	5798,2	5385,2
14,411546	5821,3	5381,5
14,424871	5792,4	5391,9
14,438201	5754,7	5387,7
14,451517	5713,2	5376,9
14,464838	5686,8	5354,4

14,478158	5670,8	5353,4
14,491475	5658,4	5368,7
14,504795	5733	5379,6
14,518116	5712,4	5410,1
14,531437	5778,5	5425,5
14,544757	5825,6	5434,6
14,557827	5796,3	5443,2
14,571148	5770,3	5451,3
14,584468	5790	5402,4
14,597793	5766	5422,1
14,611114	5757,2	5454,5
14,62443	5670,4	5479
14,637747	5664,2	5507,3
14,651059	5654	5546,2
14,664388	5656,5	5563,5
14,677704	5657,2	5586,3
14,690674	5709,5	5591,7
14,703999	5773,6	5556
14,71732	5800,2	5554,5
14,730632	5852,5	5536
14,743953	5835,7	5504,5
14,757278	5830,5	5509,7
14,770594	5848,9	5502,9
14,783914	5874,4	5466
14,797235	5860,3	5436,8
14,810556	5835,2	5419
14,823877	5860,4	5390,3
14,837189	5879,7	5395
14,850509	5904,6	5400
14,863826	5981,9	5422,8
14,877142	6019,3	5452,3
14,890463	6075,3	5478,3
14,903783	6174,9	5472,5
14,917104	6167,4	5480,4
14,930421	6100,9	5526,7
14,943746	6053,1	5517,3
14,957071	6060	5538,6
14,970387	6001,5	5511,2
14,983703	6016,6	5488,2
14,997015	5960,8	5522,3
15,010336	5892,3	5539,1
15,023657	5870,8	5576,1
15,036983	5935,1	5562,9
15,050308	5947,1	5555,1
15,063624	5964	5553,4
15,076932	5973	5557,8
15,090257	5993	5594,5
15,103578	5969,6	5588,7
15,116894	5944,2	5565,7
15,130219	5929,6	5562,6
15,143536	5848,7	5544,4
15,156852	5778,3	5538,9

15,170177	5721	5504,1
15,183502	5727,3	5474,1
15,196818	5705,2	5462,9
15,210139	5707,4	5457,6
15,223464	5722,1	5480,5
15,236776	5756,8	5530,9
15,250101	5832,8	5550
15,263422	5834,9	5548,5
15,276738	5854,3	5541,7
15,290067	5929,5	5546,4
15,303397	5890,8	5551,4
15,316713	5885,3	5569,2
15,330038	5870,4	5556,6
15,343367	5869,4	5581,8
15,356692	5835,9	5565,5
15,370013	5752,5	5551,9
15,383329	5646,8	5554,2
15,396654	5661,9	5535
15,409984	5679,3	5550,1
15,423305	5722,2	5547,9
15,436621	5739,3	5546,4
15,449933	5745,7	5577,9
15,463254	5756,8	5565,5
15,47657	5765,8	5595,5
15,489891	5714,8	5625,3
15,503207	5727,8	5650
15,516528	5733,6	5700,1
15,529844	5707,7	5726,2
15,543165	5685,3	5734,3
15,55649	5634,2	5720,6
15,569806	5649	5722,3
15,583114	5638,4	5678,7
15,596434	5658,8	5672,1
15,609764	5683	5675,3
15,623089	5670,6	5673,4
15,636401	5675,6	5681
15,64973	5680,1	5642,5
15,663051	5715	5617,1
15,676372	5712	5580,2
15,689697	5698,8	5562,2
15,703017	5630,1	5498,5
15,716342	5634,7	5435,8
15,729663	5660,9	5405,6
15,742992	5647,3	5373,9
15,756313	5653,5	5308,5
15,769629	5720,2	5260,6
15,78295	5740,2	5230,3
15,796271	5768,4	5252,6
15,809596	5819,4	5270,5
15,822917	5846,5	5302,8
15,836233	5857,4	5280,7
15,849554	5835	5293,1

15,862879	5869	5301,5
15,876199	5849,4	5325,7
15,889511	5814,9	5372,6
15,902841	5825,7	5351,7
15,916166	5825	5358,3
15,929486	5817,3	5325,2
15,942807	5846,5	5348,1
15,956128	5852	5280
15,969457	5864,3	5222,8
15,982778	5832,5	5211,3
15,996094	5828,5	5194,3
16,009419	5833,7	5177
16,022744	5795	5151,1
16,036069	5802,3	5098,2
16,049386	5711,9	5074,2
16,062698	5655,2	5061
16,076018	5636,7	5084,4
16,089335	5612,8	5072,4
16,102656	5604,8	5113,3
16,115981	5621,9	5159,6
16,129297	5627,6	5138,1
16,142619	5664,6	5147,8
16,155944	5675,6	5157,3
16,169264	5711,5	5171,8
16,182585	5700,5	5217,1
16,19591	5692,3	5206,9
16,209231	5687,6	5220,3
16,222552	5670,7	5212,1
16,235877	5661	5209,2
16,249197	5630,7	5229,2
16,262514	5580,7	5236,6
16,27583	5527,2	5275,7
16,289155	5496,2	5278,6
16,30248	5504,3	5303,4
16,315801	5559,5	5304,8
16,329117	5603,5	5274,6
16,342429	5628,4	5269,2
16,355754	5648,3	5248,9
16,369079	5671,7	5297,3
16,382391	5712,7	5321
16,395712	5723,7	5340,5
16,409028	5719	5349,1
16,422353	5701,7	5382,7
16,435674	5657,7	5391,6
16,448995	5666,2	5386,1
16,462315	5627,4	5386,9
16,475627	5630,1	5378,3
16,488948	5681,1	5396,5
16,502278	5704,7	5465,8
16,515594	5733,1	5484,8
16,52891	5728,3	5536,5
16,542231	5695,7	5534,6

16,55556	5675,7	5550,6
16,568889	5684,6	5534,4
16,582202	5716,8	5507,5
16,595527	5755,4	5514,1
16,608852	5783,3	5544,6
16,622181	5766,3	5532,2
16,635502	5702,8	5487
16,648822	5707,3	5482,8
16,662139	5671,8	5495,1
16,675459	5650,5	5510,7
16,68878	5655,4	5531,7
16,702101	5655,8	5511,4
16,715417	5691,8	5554,6
16,728733	5724,1	5561,6
16,742054	5764,1	5536
16,755379	5723,2	5540,7
16,768704	5707,6	5602,2
16,782025	5695,4	5584,8
16,79535	5639,3	5562,2
16,808666	5614,1	5563,9
16,821987	5622,4	5565,9
16,835308	5569,9	5563,7
16,848624	5623,1	5531,9
16,86194	5639,6	5505,5
16,875265	5626,3	5516
16,888582	5623,8	5481,1
16,901898	5636	5474,5
16,915219	5654,7	5507,5
16,928544	5652,2	5530,9
16,941869	5627,7	5591,6
16,955194	5594,4	5626
16,968514	5601,3	5633,1
16,981835	5596,2	5659,8
16,995156	5596,9	5656,3
17,008485	5591,5	5656,5
17,021806	5636,3	5657,5
17,035131	5701,7	5670,4
17,048452	5696,9	5675,1
17,061777	5760,8	5706,1
17,075097	5713,4	5712,6
17,088409	5682,5	5778,2
17,101734	5670,3	5806,3
17,115055	5692,8	5804,9
17,12838	5690	5785,4
17,141701	5714,1	5714,3
17,155013	5762,5	5649,7
17,168334	5773,6	5649,3
17,181663	5745,7	5688,9
17,194979	5761,4	5726,8
17,208296	5804,6	5757,4
17,221616	5862,1	5750,8
17,234941	5858,2	5722,4

17,248266	5854,4	5702,7
17,261578	5897,7	5693,1
17,274903	5906,9	5665,8
17,288224	5911,8	5666,8
17,301549	5940,5	5662,6
17,314879	5907,5	5652,6
17,328204	5892,6	5644,3
17,34152	5868,3	5577,4
17,354836	5815	5620
17,368153	5734,9	5643,8
17,381473	5715,5	5666,7
17,39479	5744	5706,3
17,408102	5748,7	5733,4
17,421418	5741,4	5722,7
17,434743	5730,9	5730,4
17,448064	5731,5	5736,4
17,461389	5699,3	5690,6
17,474709	5719,1	5727,9
17,488026	5728,4	5772,7
17,501346	5753,8	5800,1
17,514667	5763,7	5839,1
17,527984	5754,8	5868,4
17,5413	5730,5	5845,5
17,554616	5725,2	5852,2
17,567933	5707,4	5771,5
17,581253	5704,8	5716,2
17,594571	5746,6	5708
17,607891	5740,3	5657,8
17,621203	5712,6	5592,6
17,634524	5739,5	5559,9
17,647853	5728,3	5569,1
17,661178	5710,4	5647,7
17,67449	5706,2	5672,8
17,687803	5682,7	5693,2
17,701114	5614,2	5700,1
17,714439	5602,2	5712,4
17,727764	5637,4	5743,1
17,741085	5639,3	5753,1
17,75441	5669,1	5787,7
17,767731	5696,5	5802,8
17,781052	5715,9	5787,3
17,794368	5691,1	5800,3
17,807693	5690,3	5821
17,821009	5674,6	5828,6
17,834321	5664,1	5807,9
17,847642	5650,7	5837,8
17,860963	5570,2	5858,6
17,874275	5498,7	5842,7
17,887596	5473	5837,6
17,900912	5447,1	5837,6
17,914233	5446,8	5787,6
17,927549	5464,7	5769,1

17,940869	5467,6	5790,9
17,95419	5491,1	5810,5
17,967507	5469,9	5809,4
17,980827	5456,9	5835,5
17,994139	5488,7	5855,2
18,007464	5468,8	5888,4
18,020781	5486,8	5924,6
18,034097	5482,7	5891,5
18,047426	5443,4	5898,7
18,060747	5433,9	5860,7
18,074063	5403,3	5847,2
18,087388	5335,1	5801
18,100714	5321,7	5763,5
18,114039	5350,9	5725,1
18,127355	5370,2	5758,1
18,14068	5413,5	5757,5
18,153996	5441,4	5740,6
18,167317	5470,3	5730,9
18,180637	5498	5697,4
18,193958	5512,1	5614,7
18,207283	5468,6	5589,2
18,220608	5456,6	5589,4
18,233925	5454	5553,3
18,247246	5395,6	5503,7
18,260571	5399,6	5491,5
18,273887	5406,8	5480,5
18,287199	5416,2	5459,6
18,300515	5434,3	5481,3
18,313832	5426,7	5490,5
18,327148	5442,4	5486,2
18,34046	5412,9	5477,2
18,353785	5410,3	5498,7
18,367101	5409,4	5477,2
18,380422	5389,4	5477,5
18,393738	5403,2	5508
18,40705	5403,3	5529,5
18,420375	5378,6	5573,1
18,433696	5350	5624,8
18,447026	5305,2	5646,9
18,460346	5320,6	5667,1
18,473662	5378,9	5656,6
18,486992	5427,2	5664
18,500317	5460,5	5648,1
18,513638	5462,6	5621,9
18,526954	5453,4	5646,3
18,540275	5476,1	5656,4
18,5536	5477,7	5677,6
18,566925	5479,4	5702,8
18,580241	5510,5	5713,6
18,593562	5514,4	5704
18,606883	5525,4	5721,4
18,620108	5523,1	5712,4

18,633428	5528,4	5690,4
18,646745	5575,6	5659,9
18,660066	5582,6	5637,8
18,673378	5544,2	5639,8
18,686694	5570,4	5635,1
18,700014	5605,1	5671,4
18,713344	5628,2	5688,1
18,726665	5651,6	5670,4
18,739985	5596,9	5652,4
18,753302	5535,3	5599,9
18,766627	5513,6	5563,3
18,779947	5473,4	5519
18,793268	5469,6	5458
18,80658	5429,1	5435,1
18,819905	5434,5	5432
18,833226	5387,4	5506,8
18,846542	5399,5	5503,1
18,859863	5396,8	5524,4
18,873175	5401,9	5525,3
18,886496	5432,2	5560,4
18,899808	5440	5533,5
18,913137	5419,3	5510,8
18,926466	5425,8	5526,1
18,939791	5417,5	5499,7
18,953108	5422,2	5498
18,966424	5422,2	5475,1
18,979749	5438,5	5476,7
18,99307	5446	5512,9
19,006382	5431,6	5555,4
19,019703	5477	5571,6
19,033027	5518,2	5593,1
19,046353	5536,3	5609,5
19,059669	5587,8	5596,3
19,072989	5571,3	5580,7
19,086319	5515,9	5587,2
19,099639	5528,8	5608,4
19,11296	5483,4	5578,1
19,126285	5448,3	5588,3
19,13961	5443,5	5584,3
19,152931	5393,1	5603
19,166252	5368,5	5639,6
19,179577	5351	5666,1
19,192898	5335,7	5706
19,206214	5375,8	5743,4
19,219526	5403,2	5736,4
19,232851	5441,2	5753,8
19,246171	5441,8	5715,4
19,259488	5450,6	5711,4
19,2728	5438	5631,3
19,286129	5419,6	5609,9
19,29945	5399	5587
19,312762	5367,3	5631,4

19,326083	5292,7	5698,7
19,339403	5270	5715,9
19,35272	5261,9	5716,3
19,366036	5284,8	5688,5
19,379348	5322	5710,3
19,392418	5384,4	5679,1
19,405734	5423,1	5655,1
19,419054	5466,7	5675,9
19,432366	5454,2	5648,9
19,445691	5454,5	5641,7
19,459017	5438	5669,8
19,472342	5415,9	5719,9
19,485658	5403,5	5754,8
19,498983	5363,7	5764,1
19,512308	5348,1	5784,4
19,525633	5306	5744,7
19,538949	5312,8	5700
19,552261	5312,1	5652,2
19,565586	5302,4	5664,9
19,578911	5289,9	5672,1
19,592033	5344,9	5654,7
19,605362	5354,7	5638
19,618687	5355,7	5659,3
19,632004	5335,8	5699,2
19,645333	5363	5739
19,658662	5345,2	5745,9
19,671979	5327,1	5757
19,685295	5305,6	5738
19,69862	5318,8	5757,9
19,711945	5250,4	5794,2
19,72527	5205,7	5764,9
19,738586	5213,5	5762
19,751907	5273,2	5736,5
19,765223	5310,2	5762,5
19,778548	5327,6	5787,4
19,791861	5338,1	5830,1
19,805181	5337	5868,9
19,818506	5329,3	5896,6
19,831827	5342,3	5888,7
19,845147	5345,3	5888,4
19,858473	5367,6	5831,8
19,871793	5375,4	5788,2
19,88511	5344,4	5812,7
19,89843	5284,1	5788,8
19,91176	5233,2	5733,1
19,925085	5243,6	5727,3
19,938401	5261,5	5743,7
19,951722	5303,9	5771,7
19,965038	5321,7	5808
19,978363	5318,5	5842,1
19,991679	5328,1	5827,3
20,004996	5320,6	5823,1

20,018317	5305,6	5812,8
20,031642	5290,6	5764,9
20,044962	5249,8	5750,6
20,058274	5219	5718,7
20,071595	5195,9	5689
20,084911	5178	5679,1
20,098228	5181,8	5658
20,111544	5168,4	5667,6
20,12486	5206,7	5673
20,138177	5231,5	5728,2
20,151502	5245,1	5775,4
20,164827	5267,8	5793,6
20,178148	5263	5799,8
20,191152	5246,6	5770,4
20,204468	5248,7	5790,3
20,217797	5219,8	5765,8
20,231123	5215,9	5776,8
20,244435	5245,9	5738,1
20,25776	5226,6	5739,9
20,27108	5230,5	5719,3
20,284405	5244,4	5697,2
20,297722	5251	5698,5
20,311034	5228,8	5703,8
20,324359	5251,4	5695,5
20,337684	5264,1	5672,7
20,351004	5271,3	5628,4
20,364316	5303,4	5596,1
20,377637	5315,4	5606,8
20,390958	5317,7	5567,7
20,404279	5274,5	5546,3
20,417599	5250,1	5568,1
20,43092	5227,4	5581,3
20,444241	5263,9	5590,1
20,457566	5265,5	5619,9
20,470886	5287,1	5651
20,484207	5309,6	5683,8
20,497528	5336,1	5687,5
20,510844	5358,7	5682,5
20,524169	5336,1	5735,1
20,537486	5274	5761
20,550806	5196,9	5754,8
20,564127	5183,5	5769,8
20,577456	5143,8	5724,8
20,590777	5102,4	5748,9
20,604098	5091,5	5770,2
20,617422	5129,4	5769,8
20,630752	5132	5770,9
20,644073	5145,1	5771,4
20,657389	5182,5	5736,8
20,670701	5219,3	5702,5
20,684026	5228,7	5650,5
20,697351	5187,5	5610

20,710672	5191,3	5504,3
20,723997	5197,7	5477,6
20,737322	5148,8	5508,6
20,750643	5119	5524,1
20,763963	5135,6	5587,3
20,777284	5158	5584,1
20,790604	5161	5627,8
20,803925	5156,5	5666,4
20,817242	5172,9	5656,8
20,830567	5173,7	5675
20,843887	5203,3	5696
20,857208	5230,6	5664
20,870524	5285,2	5638,6
20,883845	5340,9	5630,4
20,897161	5355,3	5601,8
20,910478	5359,9	5637,2
20,923803	5348,6	5636,1
20,937128	5359,1	5670,3
20,950444	5309,5	5683,1
20,963756	5285,4	5704,6
20,977072	5258,1	5719,2
20,990389	5227,4	5744,3
21,003705	5194,5	5733,7
21,01703	5243,5	5813,4
21,030347	5274,7	5833,5
21,043663	5262	5851,5
21,056975	5246	5862,9
21,0703	5232,4	5908,6
21,083629	5225,5	5885,9
21,09695	5235,4	5936,9
21,110271	5255,3	5920,4
21,123591	5275,1	5944,1
21,136912	5288,3	5978,7
21,150229	5322,6	5991,9
21,163541	5286,4	5997,1
21,176866	5274,8	6033,5
21,190182	5304,7	6050,1
21,203502	5333,9	6067,8
21,216823	5406,3	6041,8
21,230148	5436,2	6053,9
21,243465	5429,5	6097,6
21,256785	5454,8	6067,1
21,27011	5457,6	6045,5
21,283436	5429,5	6078,8
21,296752	5339,4	6088,7
21,310064	5347,7	6093,8
21,323393	5359,7	6170
21,336718	5325,6	6162
21,350039	5289,8	6192
21,363351	5277,7	6180,2
21,376672	5329,7	6180,9
21,389992	5418,4	6182

21,403313	5435,7	6165,7
21,416638	5442,2	6154,9
21,429963	5477	6144
21,443288	5482	6110,1
21,456604	5484,8	6095,2
21,469929	5458,5	6101,3
21,483246	5467,3	6103
21,496562	5460,8	6122,8
21,509879	5490,7	6148,1
21,523204	5481,5	6130,2
21,536533	5469,6	6120,8
21,549854	5513,5	6046,9
21,56317	5509,8	6035,6
21,576491	5510,5	6040,4
21,58982	5517,6	6076,5
21,602837	5511	6066,9
21,616154	5522,4	6058,1
21,629474	5510	6073,5
21,642795	5462	6046,4
21,656124	5453,7	6045,3
21,669445	5457,2	6074
21,682761	5462	6068,2
21,696082	5464,9	6075,2
21,709407	5452,3	6084,8
21,722728	5467,2	6092,1
21,73604	5439,7	6120,3
21,749356	5424,5	6185,7
21,762677	5430,4	6249,7
21,776002	5406,8	6336,8
21,789314	5358,4	6363,6
21,802636	5351	6342,1
21,815952	5347,3	6338,9
21,829264	5327,9	6365
21,842581	5309,5	6329,6
21,855897	5354,8	6268,2
21,869218	5320,5	6237,8
21,882534	5261,7	6211,7
21,89585	5219,4	6223,1
21,909158	5164,5	6236,7
21,922483	5215,1	6197,9
21,935812	5239,1	6170,6
21,949129	5238,3	6129
21,962454	5244,8	6109,7
21,975774	5190,7	6041,2
21,989095	5192,1	6076,2
22,002411	5189,9	6133,2
22,015724	5188,8	6157
22,02904	5198,4	6194,9
22,042356	5127,6	6253,1
22,055673	5114,9	6266,4
22,068993	5153,5	6269,6
22,08231	5170,3	6258,7

22,095622	5223,5	6244,8
22,108947	5257,9	6250,6
22,122272	5272,4	6278,4
22,135593	5318,6	6310,5
22,148909	5282,4	6302,8
22,162221	5294,1	6324,3
22,175546	5284,4	6341
22,188866	5254,6	6307,2
22,202191	5236,5	6228,5
22,215512	5192	6184,1
22,228837	5175,7	6177,3
22,242162	5106,3	6203,9
22,255483	5088,4	6175,4
22,268799	5059,7	6199,1
22,282116	5037,1	6226,7
22,295436	5060,4	6203,6
22,308757	5048,7	6212,5
22,322073	5071	6229,2
22,335398	5108,8	6228,4
22,348715	5123,1	6171,9
22,362031	5194,2	6180,3
22,375361	5227,8	6177,5
22,388681	5214	6188,6
22,401993	5219,9	6209,4
22,415305	5198,4	6181,4
22,42863	5131,8	6217,2
22,441951	5093,7	6185,5
22,455276	5016,9	6164,8
22,468597	4942,3	6158,2
22,481917	4989,4	6135,3
22,495234	4979,4	6157,3
22,50855	5009,2	6120,2
22,521875	5059,9	6138
22,535191	5128,9	6114
22,548508	5106,9	6078,4
22,561828	5068,8	6065,9
22,575154	5103,2	6046,8
22,588479	5079,8	6062,6
22,601795	5089	6059,8
22,615107	5061,9	6041,3
22,628436	5045	6044,1
22,641757	5020	6043
22,655078	5021,5	6049,3
22,668394	5037,8	6104,9
22,681715	5048	6126,1
22,695035	5050,1	6118,6
22,70836	4998,5	6137
22,721681	4978,8	6115,8
22,734997	4942,2	6121,3
22,748314	4964,4	6112,1
22,761639	4920,4	6113,1
22,774964	4905,6	6078,4

22,788284	4918,2	6056,4
22,801601	4950,7	6032,5
22,814917	4936,8	6054,2
22,828247	4939,1	6051,2
22,841572	4915,5	6053,2
22,854888	4913,2	6051,4
22,8682	4929,5	5992,7
22,881525	4919,3	6001,9
22,89485	4929,6	5988,7
22,908162	4908,1	5974
22,921474	4894,2	5958,1
22,934799	4867,4	5924,7
22,94812	4885,3	5849,1
22,961441	4914,6	5844,2
22,974757	4915,8	5832,5
22,988073	4947,2	5832,5
23,001394	4990,5	5797,3
23,014715	5026,1	5802,2
23,027927	5030,9	5746,4
23,041248	5052,1	5729,2
23,054577	5066,1	5745,4
23,067893	5066,5	5734,2
23,081106	5063,9	5750,1
23,094431	5059,9	5783,8
23,107756	5051,4	5788,4
23,121072	5061,5	5760,1
23,134393	5074	5784,4
23,147714	5062,6	5779,6
23,161047	5020,1	5782,7
23,174372	4958,7	5765,1
23,187684	5004,1	5823,7
23,201005	5001,1	5855,8
23,214334	5039,5	5853,3
23,227664	5091,4	5843,2
23,24098	5116,9	5848,6
23,254296	5138,1	5877,6
23,267617	5125,2	5806,3
23,280938	5165,7	5791,1
23,294258	5219,4	5779,6
23,307579	5173,5	5763
23,320904	5154,8	5754,2
23,334225	5092,3	5705,4
23,347546	5059,1	5754,7
23,360858	5029,2	5753,3
23,374178	5050,3	5778,4
23,387499	5028,3	5762
23,400807	5029,4	5757,7
23,414127	5043,3	5803,7
23,427444	5036,4	5830,1
23,44076	4994,9	5861,7
23,454085	5016	5883,5
23,467406	5026,9	5914,2

23,480726	5027,6	5951,8
23,494047	5057,8	5910,4
23,507372	5058,9	5912,1
23,520701	5048,8	5927
23,534026	5041	5979,1
23,547339	5017	5992,1
23,560655	4995,8	6040,7
23,573984	4971,3	6074,8
23,587301	5011,6	6096,5
23,600621	5051,2	6170,4
23,613942	5049,7	6134,1
23,627263	5107,2	6159,4
23,640583	5117,6	6170,1
23,653904	5127	6195,5
23,667229	5119,6	6219,2
23,68055	5114,7	6252,5
23,693866	5121,5	6262,3
23,707182	5130,3	6228,7
23,720503	5139,6	6246,6
23,733819	5135,3	6283,3
23,747136	5147	6269,2
23,760461	5179,8	6258,1
23,773786	5190,8	6280,4
23,787111	5158,5	6299,8
23,800432	5151,7	6274,3
23,813748	5151,6	6257,1
23,826757	5155,8	6236,5
23,840086	5173,4	6230,6
23,853407	5225,8	6224,9
23,866723	5236,4	6186,4
23,880053	5225,7	6167,4
23,893377	5258,1	6168,4
23,906698	5274,9	6155,5
23,919945	5308,3	6219,9
23,933262	5271,1	6269,9
23,946578	5280	6268,4
23,959894	5243,6	6283,7
23,973219	5256,2	6253,1
23,986544	5273,1	6287,2
23,999865	5223	6317,4
24,013186	5222,8	6286,9
24,026506	5259,8	6268,5
24,039827	5308,8	6249
24,053148	5302,7	6276,2
24,06646	5282,7	6235,7
24,079776	5268,2	6248,6
24,093101	5312,7	6255,2
24,106417	5293,5	6249
24,119734	5286,1	6249,2
24,133054	5288,2	6237,9
24,146367	5334,7	6216,1
24,159687	5346,4	6216,1

24,173008	5345,4	6199,7
24,186324	5315,6	6178,8
24,199645	5274,7	6156,9
24,212957	5298,6	6116,9
24,226282	5298	6108,3
24,239598	5314	6118,1
24,252915	5330,6	6145
24,266236	5336,2	6141,9
24,279552	5347,4	6136,5
24,292877	5365,4	6125,4
24,306202	5321,8	6085,6
24,319518	5380,3	6044,8
24,332834	5321,5	6077,2
24,346155	5302,3	6012,4
24,359485	5289,7	5995,7
24,372805	5296,3	5967,6
24,386122	5266,5	5952,5
24,399438	5261,2	5952,7
24,412754	5291,1	5920,1
24,426084	5347	5925,4
24,439404	5367,6	5914,7
24,452721	5430,8	5929,7
24,466046	5471,9	5887,8
24,479371	5465,2	5907
24,492691	5459,8	5942,8
24,506004	5467	5939,1
24,51932	5458,5	5932,8
24,532645	5449,6	5898,4
24,545675	5415,1	5883,9
24,558992	5429,3	5885,1
24,572312	5401,5	5920,3
24,585624	5371,5	5925
24,598945	5335,6	5900,7
24,612261	5305	5882,9
24,625573	5301,2	5850,3
24,638903	5362	5856,7
24,652223	5403,9	5868,6
24,665544	5419	5830,2
24,678865	5398,6	5810,7
24,69219	5449,7	5777,1
24,705506	5498,8	5805,2
24,718823	5530,4	5771,4
24,732143	5519,8	5774,3
24,74546	5490,8	5775,3
24,758776	5539,3	5779,2
24,77211	5577,8	5749,7
24,785435	5578,6	5736,4
24,798755	5564,8	5656,8
24,812072	5530,2	5667
24,825401	5471,9	5722,2
24,838726	5433,1	5753,9
24,852042	5378	5845,7

24,865359	5372,4	5858,3
24,878688	5379,9	5867,7
24,892013	5388,1	5857,2
24,905334	5352,5	5867,4
24,918646	5320,1	5872,6
24,931967	5291,4	5880,3
24,945292	5303	5910,5
24,958612	5326,4	5916,3
24,971933	5384,4	5942
24,985254	5359,2	5993
24,998574	5347,8	6021,2
25,011895	5355,2	6087,2
25,025216	5382,9	6094,8
25,038536	5403,6	6064,2
25,051857	5417,6	6061,5
25,065178	5413,9	6053
25,078499	5434	6057,4
25,091815	5464,5	6041,2
25,105131	5474,3	6070,2
25,118452	5467,4	6126,8
25,131768	5410,3	6153,5
25,145089	5358,7	6172,5
25,15841	5364,4	6173,8
25,17173	5349,9	6130,1
25,185047	5301,9	6145,7
25,198372	5302,8	6116,8
25,211693	5282	6122,1
25,225009	5311,4	6135,5
25,238325	5283,5	6106
25,251646	5274	6132,2
25,264971	5259	6122,7
25,278296	5246,1	6145,3
25,291621	5244	6170,4
25,304937	5236,4	6200,8
25,318258	5205,5	6220,7
25,33157	5215,3	6209,7
25,344895	5210,8	6204,2
25,358211	5195,1	6145,8
25,371532	5182,8	6085,4
25,384857	5202	6066,8
25,398173	5214,6	6009
25,41149	5213,4	5992,1
25,424811	5241	6025,8
25,438131	5252	6016,4
25,451456	5250,3	6029,9
25,464772	5274,5	6032,1
25,478089	5285	6066,4
25,491418	5252,7	6077,6
25,504743	5233,3	6093,1
25,51806	5213,7	6040,7
25,531372	5194	6065,6
25,544693	5203,3	6060,9

25,558013	5249,2	6046,1
25,571338	5267,5	5990
25,58465	5282,7	5963,4
25,597975	5281,8	5959,7
25,611296	5332,4	5992,3
25,624621	5354,2	5994,8
25,637933	5353,4	5991,7
25,651254	5367,3	5970,3
25,664579	5354,7	5960,5
25,677904	5361,8	5951,5
25,691216	5367,5	5968,2
25,704532	5351,5	5997,6
25,717848	5328,1	5982,7
25,731178	5288,9	6025
25,744498	5257,1	6034,7
25,757819	5251,6	6038,6
25,771136	5292,3	6080,2
25,784461	5286	6054,6
25,797786	5299,6	6054
25,811102	5326,2	6048,2
25,824409	5340,5	6091,7
25,837734	5383,9	6062,4
25,851055	5357,6	6087,9
25,864376	5349,5	6055,2
25,877692	5303,5	6048,3
25,891013	5284,5	6073,4
25,904329	5302,7	6022,5
25,91765	5261,8	6038,9
25,930966	5292,1	6047,4
25,944287	5256,8	6077,6
25,957603	5239,8	6070,1
25,970924	5236,4	6059
25,984249	5232,5	6070,1
25,99757	5246,7	6048,4
26,010891	5234,8	6019,8
26,024211	5156,4	6019,6
26,037536	5168,9	6044,5
26,050861	5177,3	6029
26,064177	5177,7	6001,2
26,07749	5120	5974
26,090819	5147,7	5941,5
26,10414	5052,1	5961,2
26,117456	5042,2	5967,6
26,130777	5034,1	5965,7
26,144098	5041,6	5929,5
26,157418	5039,2	5933,6
26,170739	5022,6	5886,5
26,184064	5032,8	5909,1
26,197384	5045,9	5945,5
26,210709	5022,8	5928,3
26,22403	5043,3	5908,8
26,237342	5108,1	5835,8

26,250663	5071,4	5793
26,263984	5075,1	5761,6
26,277309	5085,5	5748,4
26,290634	5090,1	5757,5
26,30395	5102,4	5750,6
26,317266	5084,2	5759,2
26,330596	5057,1	5733
26,343925	5012,7	5738,8
26,357246	4972,8	5702,3
26,370566	4953,8	5724,9
26,383761	4900,5	5724,2

Supplementary Figure 6g **ECIS: OA vs RA FLS, 3rd run (resistance - ohm)**

time	OA-FLS	RA-FLS
0,00055028	2170,1	2463,3
0,01603889	2250,1	2550
0,02929861	2350,4	2659,1
0,04261917	2487,7	2784,7
0,05594	2615,6	2937,9
0,06926056	2738,5	3094,1
0,08257694	2863,7	3275,5
0,09589778	3024,9	3480,5
0,10921833	3185,9	3665,8
0,12253028	3352,7	3853,2
0,13584667	3497,4	4038,8
0,1488425	3645,6	4200,8
0,16216306	3843,2	4360,7
0,17547944	3996,1	4542,3
0,18879583	4211,1	4700,5
0,20211639	4439,6	4842
0,21543278	4659,6	4998
0,22840694	4906	5138,4
0,24171889	5154,2	5252,5
0,25503972	5406,5	5373,4
0,26837333	5606,9	5485,9
0,28169389	5830,5	5597
0,29501028	6018,1	5704,1
0,30833528	6203,6	5803,1
0,32166028	6336,4	5911,5
0,33473833	6481,3	5994,6
0,34806333	6655,9	6095,9
0,36138833	6769	6167
0,37471361	6881,6	6241,9
0,38803861	6950,7	6328,4
0,40136361	7073,2	6419,8
0,41468417	7199,9	6497,7
0,428005	7331	6554,7
0,44132556	7441	6609,4
0,45464639	7469,1	6689,7
0,46796694	7526,6	6758,7
0,48128778	7617,5	6816,2
0,49461806	7722,9	6885,3
0,50793861	7803,2	6958,5
0,521255	7823,1	7017,5
0,53457583	7851	7074,6
0,547905	7875,7	7139,3
0,56122583	7883,1	7204,3
0,57454639	7950,2	7273,2
0,58787139	7971	7343
0,60119222	8019,7	7404,7
0,61451278	8031,2	7452,9

0,62783778	8072,2	7481
0,64115417	8188	7498,9
0,65447056	8287,7	7551,6
0,66778694	8367,9	7587,1
0,68111194	8355,8	7648,3
0,69444139	8344,4	7698,3
0,70775778	8434,7	7737,6
0,72107417	8424	7744,1
0,73439028	8466,9	7774,6
0,74771111	8474,8	7819
0,7610275	8487,8	7866,5
0,77434806	8523,4	7892,9
0,78766444	8577	7921,8
0,80098944	8652,2	7948
0,81431444	8679,3	7957,1
0,82763083	8698,3	7978,5
0,84094722	8702,1	8015,6
0,85427222	8786,8	8045,9
0,86759306	8792,5	8087,6
0,88090583	8835,6	8106,7
0,89422667	8876,8	8125,1
0,90753861	8960,3	8151,4
0,92085083	8972	8181,3
0,93417139	9005,8	8205
0,94749194	8970,8	8230,1
0,96081278	8957,6	8232,9
0,97412917	9084,3	8261,8
0,98744556	9115,7	8297,8
1,0007619	9178,5	8343,4
1,0140825	9110,4	8382,4
1,0274042	9117,8	8416,9
1,0407292	9141,2	8451,8
1,0540542	9161,8	8495,1
1,0673661	9235,4	8522,9
1,0806869	9257,2	8542,5
1,0940033	9168,2	8586,7
1,1073283	9184,6	8610,7
1,1206447	9139,3	8633,6
1,1339567	9162,6	8642,1
1,1472686	9176,1	8641,5
1,1605936	9230,4	8650,7
1,17391	9264,1	8654
1,1872222	9217,4	8679,4
1,2005428	9232	8706,4
1,2138678	9240,5	8728,1
1,2271928	9267	8751,5
1,2405136	9239,5	8778,4
1,2538256	9245,6	8790,1
1,2671464	9216,7	8801,7
1,2804583	9190,2	8800,9
1,2937789	9196,9	8820
1,3071039	9241,9	8867,5

1,3204203	9145,8	8878,1
1,3337367	9147,3	8883
1,3470617	9179,8	8913,5
1,3603867	9160	8932,6
1,3737075	9139,3	8939,3
1,3870239	9179,2	8952,5
1,4003403	9133,2	8974,4
1,4136653	9036,7	9017,9
1,4269817	9045,1	9034,1
1,4402936	8993,9	9060,6
1,4536056	8948	9079,3
1,4669219	8902,5	9098,1
1,4802428	8882,8	9136,4
1,4935589	8908,2	9134,7
1,5068797	8948	9107
1,5201917	8887,7	9129,2
1,5335125	8919,6	9151
1,5468375	8890,6	9177,6
1,5601633	8930,9	9191,1
1,5734797	8834	9218,8
1,5868006	8811,8	9222,7
1,6001256	8748,4	9261,9
1,6134419	8757,1	9284
1,6267581	8814,3	9310,8
1,6400875	8831,7	9341,4
1,6534125	8841,4	9365,4
1,6667289	8792,2	9386,4
1,6800408	8780,7	9413,6
1,6933617	8715,8	9398,6
1,7066781	8683	9424
1,7199944	8660,7	9428,7
1,7333064	8661,1	9464,2
1,7466269	8644,2	9485,4
1,7599433	8584,1	9523
1,7732683	8550,3	9567,6
1,7865806	8525,8	9600,8
1,7998969	8507,8	9626,5
1,8132219	8450,4	9658,5
1,8265469	8361,3	9660,2
1,8398675	8424,3	9677,6
1,8531839	8336,5	9705,8
1,8665047	8349,9	9727,3
1,8798167	8357	9725,8
1,8931461	8397,2	9749,3
1,9064622	8390,8	9750
1,9197786	8353,1	9774,3
1,933095	8323,3	9782,9
1,94642	8270,9	9837,7
1,9597408	8261,2	9849,8
1,9730528	8192,6	9897
1,9863778	8164	9909,1
1,9997028	8147,7	9926,2

2,0130278	8040,7	9921,7
2,0263486	7970,4	9935,2
2,0396692	7968,6	9962,7
2,0529942	7986,9	9960
2,0663192	8067	9946,9
2,0796444	8091,3	9956,8
2,0929617	8048,6	9971,1
2,1062781	7982,4	9983,4
2,1195944	7913,1	9976,3
2,1329194	7868,5	9953,3
2,1462358	7742,1	9954,3
2,1595478	7705	9960,2
2,1728728	7644,5	9994,9
2,1861933	7676,7	9995,5
2,1995142	7701,1	9983,1
2,2128347	7753,4	10012,2
2,2261556	7770,4	10035,7
2,2394761	7665,9	10109,3
2,2527969	7685	10119,6
2,2661219	7668,9	10109,8
2,2794469	7655,9	10140,8
2,2927678	7701,8	10171,8
2,3060883	7675,5	10166,1
2,3194133	7670,4	10187,1
2,3327297	7660,2	10189,6
2,3460461	7685,4	10145,3
2,3593625	7668,4	10149,5
2,3726917	7652,4	10173,8
2,3860081	7637,1	10216,6
2,3993244	7613,3	10216,7
2,4126453	7613,4	10241
2,4259658	7590,7	10296,5
2,4392867	7596,8	10321,7
2,4526072	7572,4	10341,9
2,4659192	7591	10336
2,47924	7549,4	10331
2,492565	7535,8	10335,2
2,5058814	7594,1	10316,7
2,5191933	7589,5	10304,1
2,5325142	7578,4	10326,8
2,5458306	7509,8	10342,8
2,5591556	7532	10341,1
2,5724719	7483,4	10332,7
2,5857925	7504	10359,6
2,5991175	7404,5	10347,8
2,6124425	7365	10373,1
2,6257544	7381,2	10372,3
2,6390753	7317,9	10400,9
2,6523958	7307,6	10398,3
2,6657211	7361,5	10465,9
2,6790417	7334,8	10506,6
2,6923581	7341,5	10522,6

2,70567	7227,8	10555,7
2,7189864	7244,4	10579,5
2,7323114	7194,2	10580,4
2,7456322	7191,9	10603,3
2,7589528	7198	10582,1
2,7722647	7227,9	10584,5
2,7855897	7223,1	10607,5
2,7988811	7226,6	10596,5
2,8122019	7216,6	10571,8
2,8255225	7170,9	10593,1
2,8388389	7157,8	10565,8
2,8521508	7217,3	10590,4
2,8654803	7250,2	10625,3
2,8788011	7246,9	10616
2,8921131	7173	10573,5
2,9054381	7169,7	10534,4
2,9187631	7170,1	10536
2,9320836	7165,2	10515,5
2,9454044	7129,6	10485,7
2,9587164	7166,3	10507,7
2,9720414	7150,5	10526,2
2,9853664	7103,6	10533,7
2,9986828	7170,7	10517,5
3,0120036	7170,4	10529,2
3,02532	7187,4	10506,3
3,0386364	7148,8	10536,1
3,0519569	7085,6	10513,3
3,0652819	7039,6	10505,7
3,0785983	6962,1	10486,2
3,0919189	6916,4	10494,6
3,1052442	6856,6	10493,8
3,1185647	6899,2	10486,9
3,1318856	6854	10523,9
3,1452017	6845,1	10546,7
3,1585139	6874,9	10584,8
3,1718389	6881,8	10572
3,1851639	6832,4	10562,6
3,1984803	6869,8	10558,3
3,2118053	6863,7	10557,5
3,2251347	6833,8	10559
3,2384508	6838	10579,3
3,2517717	6832,6	10589,4
3,2650933	6788,5	10634,3
3,2784139	6756,2	10612,1
3,2917303	6750,1	10660,7
3,3050467	6723	10653,2
3,3183717	6746,1	10599,9
3,3316925	6730,8	10601,7
3,3450131	6737,1	10566,5
3,3583381	6724,2	10570,4
3,3716631	6750,4	10569,4
3,3849794	6705,2	10582,5

3,3982914	6759,9	10621,6
3,4116122	6754	10637,2
3,4249372	6758,5	10577,4
3,4382622	6702,8	10582,6
3,4515831	6719	10564,9
3,464895	6629,1	10495,5
3,4782114	6650,9	10425,2
3,4915278	6647,8	10412,3
3,5045581	6665,2	10404,4
3,5178875	6661,6	10463,7
3,5312125	6663,5	10479,8
3,5445375	6639,9	10599,5
3,5578539	6591,7	10605,4
3,5711744	6588	10612,8
3,5844908	6601,2	10626,2
3,5978117	6663	10591,8
3,6111278	6646,9	10665,8
3,6244486	6716,5	10704,9
3,6377736	6733,9	10698,9
3,6510942	6729,7	10713,5
3,6644106	6660,5	10737,2
3,6777228	6617,2	10776,2
3,6910433	6594,1	10808,8
3,7043597	6586,1	10872,3
3,7176847	6566,4	10929,3
3,7310056	6549	10886
3,7443217	6453,4	10855,4
3,7576425	6427,8	10849,5
3,7709544	6380,4	10875,9
3,7842753	6408,5	10936,2
3,7975958	6490,3	10912,9
3,8109122	6499,1	10876,2
3,8242331	6510,4	10839,3
3,8375581	6454,9	10854,4
3,8508786	6419,5	10846,7
3,8642036	6436,2	10793,7
3,8775244	6389,2	10773,4
3,890845	6413,1	10773,6
3,9041658	6432	10838,3
3,9174733	6462,5	10843,2
3,9307942	6458,3	10871,7
3,9441147	6440,1	10886,3
3,957445	6420,9	10860
3,9707658	6457,2	10807
3,9840822	6444,2	10848,7
3,9973942	6400,7	10837,9
4,010715	6371,4	10797,4
4,02404	6392,6	10787,4
4,0373606	6412,9	10763,6
4,0506822	6449,5	10653,1
4,0640031	6428,7	10645,7
4,0773192	6381,8	10655

4,09064	6400,8	10713,5
4,1039606	6358,9	10642,5
4,1172728	6294,1	10650,1
4,1304114	6237,7	10613
4,1437278	6186,3	10633
4,1570572	6158,6	10551,2
4,1703778	6147,8	10591
4,1836897	6128,4	10605,1
4,1970147	6132,6	10691,2
4,2103311	6087,4	10755
4,2236561	6063,7	10748,2
4,2369811	6088,9	10737,2
4,2503019	6041,2	10724,4
4,2636183	6064,6	10658,9
4,2769347	6075,8	10688,6
4,2902511	6038,6	10625,1
4,3035847	6018,2	10645,3
4,3168967	5987,9	10530,5
4,3302217	5960,8	10558,2
4,3435467	5947	10600,4
4,3568675	5917,3	10639,2
4,3701839	5897,6	10674,3
4,3835044	5921,1	10691,9
4,3965219	5957,4	10579,5
4,4098425	5903,9	10579,9
4,4231633	5905,6	10573,4
4,4364839	5907,7	10525,2
4,4498089	5950,9	10612,4
4,4631253	5979,1	10653,8
4,4764383	6010,9	10666,1
4,4897547	5980,2	10688,8
4,5030883	5955	10692
4,5164178	5941,1	10706,9
4,5297342	5919,4	10702
4,5430503	5894,5	10697,2
4,5563667	5851,9	10682,2
4,5696917	5910,2	10762,7
4,5830167	5923,8	10689,2
4,5963331	5930,8	10715,7
4,6096453	5967,7	10720,1
4,6229703	5963	10790,8
4,6362953	5931,5	10697,8
4,6494208	5917,4	10687,1
4,6627417	5912,2	10670,4
4,6760536	5887,3	10591,9
4,6893786	5847	10529,4
4,7026994	5843,2	10427,7
4,71602	5923	10331,3
4,7293364	5999,7	10386,6
4,7426658	6094	10290,3
4,7557092	6167,1	10222,6
4,7690256	6196	10164,3

4,7823506	6227,6	10151,3
4,7956711	6202,1	10212,1
4,8087925	6166,9	10280,7
4,8221133	6107,9	10261,7
4,8354253	6037,4	10293,6
4,8487511	5988,9	10250,8
4,8620719	5979,5	10280
4,8753969	5958	10182,5
4,8887175	5990,1	10102,4
4,9020383	6023,7	10076,9
4,9153633	6007,7	10028,4
4,9286842	5950,4	10018,9
4,9420047	5889,8	10008,1
4,9553297	5897,5	9952,3
4,9686506	5885,9	9889,3
4,9819711	5869,1	9991,7
4,9952919	5907,1	10010,7
5,0086039	5955	10040,1
5,0219289	5916,8	10026,1
5,0352539	5905,1	10063,8
5,0485744	5864,6	10100
5,0618908	5834,3	10026,7
5,0752072	5839	9967,2
5,0885194	5844,4	9929
5,10184	5852,4	9913,5
5,1151608	5895,8	9957,5
5,1284814	5951,6	9996,1
5,1417892	6019,1	9997,2
5,1551097	6024,9	9921,1
5,1684306	6055	9881,7
5,1817511	6049,1	9768,9
5,1950806	6072,3	9797,3
5,2083969	6048,1	9778,6
5,2217133	6081,8	9824,6
5,2350383	6103,1	9699,2
5,24806	6100,2	9702,6
5,2613817	6084,9	9801,1
5,2746981	6079,4	9800,4
5,28801	6073,1	9825,7
5,301335	6054,5	9816,7
5,31466	6029,6	9783,2
5,3279764	6053,2	9794,9
5,34131	6064,1	9750,4
5,354635	6092,9	9686,7
5,36796	6072	9623,9
5,3812764	6102,8	9639,4
5,3945928	6119,3	9661,3
5,4079136	6136	9665,8
5,4212428	6109,3	9662,9
5,4345636	6026,8	9597,2
5,44788	5977,9	9600,9
5,461205	5957,2	9626,1

5,4745256	6002	9628,8
5,4878419	6071,7	9567,3
5,5011628	6085,1	9485,5
5,5144878	5995,2	9483,1
5,5278083	5928,4	9511,3
5,5411247	5908,6	9471,1
5,5544411	5893,9	9431,3
5,5677669	5935	9385,8
5,5810886	5981,8	9359,1
5,5944094	5997,2	9363,4
5,6077344	6011,7	9367,9
5,6210594	6050,2	9332,6
5,6343803	6022,6	9292,4
5,6476922	6002,1	9203,1
5,6610172	5997,8	9133,6
5,6743344	6008,1	9124,7
5,6876508	6023,9	9100,5
5,7009758	5997,1	9042,7
5,7142967	5954,7	9014,1
5,7276172	5913,2	9043,8
5,7409381	5833,7	8999,8
5,7542672	5782,6	8930,2
5,7675836	5787,1	8972,5
5,7809044	5832,1	8914,3
5,7942208	5844,1	8893,7
5,8075414	5821,2	8925,7
5,8208664	5833,6	9067,3
5,8341828	5847,2	8985,6
5,8475022	5789,7	8969,8
5,8608208	5736,9	8951,6
5,8741372	5707,4	8945,8
5,8874622	5669,9	8868,8
5,9007917	5719,2	8841,2
5,9141122	5754,8	8903,5
5,9274286	5673,2	8874,4
5,9407581	5667,4	8884,1
5,9540831	5648,4	8838,1
5,9673994	5620	8900
5,98072	5632,1	8915,1
5,9940492	5599	8855,1
6,0073711	5598,1	8845,1
6,0206917	5564	8864,1
6,0340125	5519,4	8828,5
6,0473375	5479,6	8781,4
6,0606625	5465,5	8803
6,0739744	5447,5	8889,9
6,0872953	5470,9	8836,9
6,1006158	5465,8	8806,5
6,1139322	5532,1	8709,6
6,1272486	5547,2	8633,1
6,1405736	5525	8606,1
6,15389	5528,8	8669,7

6,1672064	5530,7	8719,9
6,1805183	5503,5	8691,7
6,1938389	5481,6	8620,9
6,2071597	5456,5	8619,8
6,2204847	5426,2	8647,2
6,2338097	5398,4	8630,6
6,2471392	5422,8	8588,5
6,2604597	5461,7	8604,8
6,2737806	5413,1	8576,7
6,2871056	5351	8528,9
6,3004306	5347,2	8500,2
6,3137511	5347,8	8469,7
6,3270589	5348,4	8452,3
6,3403797	5364,5	8444,8
6,3537014	5377,6	8477,1
6,3670142	5380,1	8455,2
6,3803436	5399,5	8465,2
6,3936642	5378,1	8425,5
6,4069806	5317,5	8358,7
6,4203056	5318,7	8338,1
6,4336264	5317,9	8325,1
6,4469469	5376	8377,5
6,4602719	5391,8	8433,6
6,4735928	5420,7	8469,4
6,4869047	5381,6	8432,5
6,5002297	5378,5	8347,3
6,5135506	5349,8	8334,3
6,5268756	5306,8	8365,4
6,5401919	5254,1	8312
6,5535083	5224,9	8298,6
6,5668289	5183,6	8294,3
6,5801583	5155,1	8336,8
6,5934747	5162,9	8306,8
6,6067953	5161	8225,3
6,6200997	5121,5	8242,1
6,6334117	5095	8247,9
6,6467281	5110,3	8188,3
6,6600531	5115,3	8158,5
6,6733736	5111,7	8236,4
6,6866858	5134,6	8250
6,7000064	5145,1	8222,4
6,7133314	5242	8210,2
6,7266564	5270,6	8242,4
6,7399728	5326,5	8199,2
6,7532936	5343,9	8162,2
6,7666228	5401,9	8086,7
6,7799481	5437,7	8096
6,7932686	5449,5	8023,7
6,806585	5512,6	8021,6
6,8199014	5512	8008,8
6,8332219	5552,7	7907,9
6,8465469	5618,5	7845,8

6,8598633	5639,9	7902,4
6,8731842	5662,2	7864,6
6,8865006	5713,8	7826,5
6,8998256	5638,3	7869,3
6,9131461	5628,6	7818,7
6,9264581	5610,3	7773,6
6,9397744	5621	7742,2
6,9530908	5580,3	7799,1
6,9664158	5525,8	7838
6,9797367	5518,4	7838,3
6,9930617	5517,5	7810,5
7,0063781	5519,9	7879,8
7,0197031	5490,4	7844,3
7,0330236	5519,3	7852,2
7,0463314	5509,9	7811,3
7,0596519	5458,1	7840,9
7,0729772	5493,8	7805,9
7,0862933	5499,6	7827,7
7,0996142	5595,9	7780,3
7,1129436	5602,1	7744,6
7,1262686	5595,6	7763,5
7,139585	5608,2	7743,3
7,1528894	5606,7	7779,3
7,1662103	5644,8	7883,4
7,1795361	5624,3	7913,3
7,1928611	5608,3	7884,8
7,2061819	5646,2	7832,4
7,2195069	5635,5	7850,6
7,2328275	5658,9	7921,7
7,2461483	5669,9	7914,6
7,2594689	5649,9	7921,5
7,2727853	5656,9	7959,1
7,2861061	5678	7973,6
7,2994267	5673	7934,1
7,3127475	5654	7980,8
7,3260636	5666,1	8070,2
7,3393889	5678,5	8096,7
7,3527094	5675,5	8064,8
7,3660303	5686,9	8114
7,3793464	5706,1	8049,4
7,3926628	5734,5	8077,5
7,4059836	5765	8073,4
7,4193	5822,8	8041
7,432625	5890,5	8080,1
7,4459456	5857,5	7960,1
7,459275	5812,3	7896,7
7,4725956	5818,2	7863,5
7,4859164	5806,9	7827,9
7,4992414	5812,6	7796,5
7,5125619	5802,7	7763,4
7,5258783	5792,6	7705
7,5392033	5803,6	7657,4

7,5525286	5786,2	7661,4
7,5658536	5783,5	7715,7
7,5791742	5725,5	7709,2
7,5924861	5744,2	7738,6
7,6058111	5742,3	7763,8
7,6191364	5716,1	7797,2
7,6324525	5670,8	7865
7,6457778	5608,6	7916,3
7,6590983	5630,9	8060,1
7,6724189	5633,7	8106,9
7,6857147	5677,9	8103,7
7,6990397	5680	8098,6
7,7123614	5604	8106,5
7,7256864	5503,3	8134,3
7,7390069	5449,2	8165,6
7,7523278	5393,5	8165,5
7,7656397	5449,1	8174,4
7,7789606	5497,7	8112,4
7,7922811	5541,6	8094,5
7,8055931	5616,9	8085,9
7,8189094	5663,9	8071,2
7,8322344	5716,9	7971,7
7,8455553	5773,7	8002,5
7,8588717	5895,9	8054,6
7,8721967	5838,4	8075,3
7,8855217	5791,4	8089,5
7,8988381	5808,4	8135
7,9121631	5770,9	8217,5
7,9254836	5792,1	8195,7
7,9388131	5877,9	8203,9
7,952125	5885,1	8266,7
7,9654458	5944,6	8295,4
7,9787664	5956	8277,8
7,9920914	5978,5	8233
8,0054078	5948,9	8189,6
8,0187372	5961,2	8152,6
8,0320578	5898,6	8110,1
8,0453786	5891,4	8050,6
8,0586992	5886	8036,6
8,0720156	5853	7979,1
8,0853406	5835,3	7911,1
8,0986569	5859,4	7898,1
8,1119778	5823,8	7813,3
8,1252942	5793,5	7740
8,1386233	5738,1	7674,4
8,1519497	5694,4	7645
8,1652661	5761,4	7602
8,1785825	5731,2	7627,9
8,1919031	5677,6	7656,4
8,2052281	5676,4	7648,7
8,2185531	5622	7607,1
8,2318694	5596,6	7625,6

8,2451858	5569	7537,4
8,2585108	5544,3	7598
8,2718317	5586,4	7712
8,2851567	5594,7	7785,3
8,2984772	5611,1	7859,5
8,3118022	5578,1	7936,1
8,3251272	5546,7	8002,6
8,3384522	5520,8	8013,3
8,35177	5537,9	8047
8,3650864	5588,4	8102,2
8,3784028	5531,7	8112,7
8,3917233	5514,1	8122,9
8,4050442	5485,1	8158,5
8,4183647	5481,4	8188,6
8,4316853	5492,8	8189,8
8,4450061	5516,4	8174
8,4583267	5534,4	8115,9
8,4716475	5532,9	8160,3
8,4849681	5485,8	8178,5
8,4982803	5405,3	8166,6
8,5116053	5454,5	8199,4
8,5249303	5433,7	8162
8,5382508	5471,1	8151,3
8,5515631	5462	8150,7
8,5648836	5518,1	8217,3
8,5782086	5559,1	8206,8
8,5915306	5619,3	8109,9
8,6048514	5555,9	8044,7
8,6181775	5619	8018,7
8,6314983	5646	8015,6
8,6448147	5640,3	8061,7
8,6581267	5629,6	8065,2
8,6714517	5662,9	8057,2
8,6847811	5687,9	8079
8,6980972	5686,7	8129,4
8,7114181	5693,2	8045
8,7247386	5715,5	8055
8,7380681	5669,7	8089
8,7513931	5591,3	8099,3
8,7647094	5559,8	8101,7
8,7780214	5573,1	8029,6
8,7913422	5567,1	8010,3
8,8046672	5608,7	8071,7
8,8179878	5572,7	8044,5
8,8313086	5535,3	8049,7
8,8446336	5498,6	8133,4
8,8579586	5476,1	8147
8,8712836	5426	8194
8,8846	5399	8244,1
8,8979208	5437,7	8232,2
8,91125	5485,7	8197
8,9245708	5519,9	8084,8

8,9378872	5488,6	8064,4
8,9512122	5480,5	8057,1
8,9645328	5508	7987,6
8,9778536	5554	8065,2
8,99117	5520,1	8113,1
9,0044697	5537,2	8157,4
9,0177861	5497,1	8141,9
9,0311069	5530,2	8158,6
9,0444319	5567,3	8150,1
9,0577569	5579,9	8102,3
9,0710775	5572,1	8076,3
9,0844028	5607,6	7990,1
9,0977278	5641	7965,4
9,1110442	5685,5	7895,7
9,1243647	5698,5	7859,8
9,1376767	5717,9	7828,4
9,1509975	5686,8	7779
9,1643181	5743,9	7796
9,1776389	5745,2	7813,8
9,1909639	5707,9	7829,9
9,2042844	5629,7	7806,8
9,2176008	5675,2	7762,6
9,2309172	5659,9	7686,4
9,2442422	5613,5	7677
9,2575631	5594	7682
9,2708806	5621,7	7658,8
9,2842056	5661,9	7608
9,2975275	5639,1	7583,3
9,3108483	5713,3	7565,1
9,3241775	5706,6	7521,5
9,3374939	5678,1	7537,8
9,3508189	5633,5	7554,1
9,3641397	5615,1	7559,7
9,3774603	5586,5	7544,7
9,3907853	5537,4	7538,2
9,4041103	5550,1	7510,5
9,4174311	5535,7	7485,5
9,4307517	5522,5	7378,3
9,4440681	5479,7	7317,8
9,4573931	5444	7266,2
9,4707139	5357,5	7209,4
9,4840389	5327,4	7216,2
9,4970606	5262,6	7173,7
9,5103769	5167	7210,7
9,5236975	5232	7183,9
9,5370183	5295,9	7238,9
9,5503433	5364,5	7270,1
9,5636639	5460,2	7267
9,5769761	5472	7294,6
9,5902922	5533,6	7318,3
9,6036131	5530,7	7399,8
9,6169336	5573,7	7440,3

9,63025	5600	7499,7
9,6435708	5573,5	7493,6
9,6568925	5523,7	7523,4
9,6702131	5517,8	7459,5
9,6835381	5487	7423,6
9,6968589	5468,2	7442
9,7101794	5390,4	7352,3
9,7235003	5384,2	7286,9
9,7368208	5329,1	7224
9,7501458	5306,2	7211,4
9,7634622	5318,3	7100,8
9,7767744	5341,9	7097,6
9,7900906	5392,9	7156,1
9,80342	5443	7210,9
9,8167494	5528,6	7253,7
9,83007	5588,8	7324,6
9,8433864	5567,4	7342,6
9,8567028	5594,2	7390,5
9,8700278	5618,5	7428,5
9,8833528	5602,4	7461,1
9,8966647	5606,3	7496
9,9099769	5619,7	7519,1
9,9232975	5664,3	7510,4
9,9366139	5694,9	7565,6
9,9499356	5763,5	7521,5
9,9632606	5793,3	7584,9
9,9765769	5770,1	7519,8
9,9898933	5719,9	7498,5
10,003214	5746,8	7433,5
10,016535	5756	7364,8
10,029851	5764,4	7327
10,043163	5819,8	7352,9
10,056479	5812,7	7354,2
10,069809	5808,5	7355,8
10,083134	5831,3	7423,7
10,096112	5795,4	7393,4
10,109424	5778,5	7406
10,122745	5780,8	7391,9
10,136061	5818	7392,5
10,149392	5874,4	7480,6
10,162712	5892,5	7462,7
10,176029	5964,7	7514,2
10,189342	6045,5	7583,4
10,202658	6059,9	7613
10,215979	6075,9	7615,8
10,229304	6070,2	7678,8
10,24263	6075	7728,3
10,255951	6053,1	7752,6
10,269276	6015,6	7764,6
10,282592	6025,5	7776,9
10,295917	6089,8	7748,4
10,309238	6103,1	7705,3

10,322558	6127,8	7694
10,335879	6099,5	7643,5
10,349204	6084,7	7683,3
10,362525	6065,8	7726
10,375845	6103,7	7707
10,389157	6103,7	7698,5
10,402478	6074,3	7655,1
10,415799	6071,8	7609,8
10,429119	6085,4	7604,6
10,44244	6074,8	7553,7
10,455756	6056,2	7455,3
10,469069	6115,4	7338,6
10,482391	6097,8	7262,4
10,495708	6073,2	7173,2
10,509037	6029,4	7177,1
10,522358	5956,8	7208,4
10,535679	5980,2	7270,9
10,548995	5955,2	7305,6
10,562316	5954,4	7303,7
10,575641	5894,4	7291,3
10,588961	5881,8	7258,9
10,602282	5871,2	7236,9
10,615611	5851,6	7192,5
10,628933	5898,1	7194
10,642258	5883,2	7209,7
10,655583	5897,6	7243,2
10,668904	5880,3	7251,3
10,682216	5895	7183,7
10,695541	5896,2	7177,5
10,708874	5851,5	7175,2
10,722199	5797,8	7199,3
10,735516	5770,8	7166,4
10,748845	5774,3	7154,3
10,762166	5814,5	7060,7
10,775487	5757	7012,7
10,788803	5743,1	7004,3
10,802115	5680,9	7023,5
10,81544	5700,7	7043,4
10,828761	5668,3	7095,7
10,842081	5680,4	7105,2
10,855398	5721,6	7110,8
10,868723	5768,1	7178,8
10,882048	5771,7	7171,6
10,895368	5765,5	7149,3
10,908681	5804,2	7096,1
10,922002	5706,5	7113
10,935327	5714,4	7125,6
10,948644	5770,7	7092,6
10,96196	5729,3	7043,7
10,975281	5710,2	7064,4
10,988592	5747,2	7082,8
11,001913	5755	7103,1

11,015238	5752,4	7137
11,028555	5731,4	7167,5
11,04188	5747,5	7144,2
11,0552	5757,4	7174,1
11,068521	5755	7195,1
11,081846	5802,9	7179,2
11,095167	5794,9	7164,1
11,108488	5781,7	7131,9
11,121813	5764,5	7136,1
11,135126	5749,9	7134,8
11,148451	5787,2	7105
11,161772	5786,8	7085,8
11,175102	5814,6	7016,5
11,188418	5841,7	7018,8
11,201734	5833,5	7054,2
11,215046	5810	7017,9
11,228363	5774,5	6920,5
11,241684	5734,4	6964,3
11,255001	5785,9	6974,6
11,268326	5798,8	6977,4
11,281642	5767,7	6922,3
11,294963	5747,9	6822
11,308284	5737,2	6757,9
11,321604	5670,9	6711,8
11,334925	5618,9	6648,9
11,348241	5628,7	6668
11,361566	5608,3	6674,1
11,374887	5611	6681
11,388208	5610,5	6630,9
11,401524	5624,6	6593,5
11,414845	5666,7	6624,5
11,428166	5623,3	6607,3
11,441482	5600,3	6617,4
11,454804	5588	6641,1
11,468124	5547,7	6661,4
11,481445	5520,9	6617,5
11,494766	5515,1	6616,6
11,508091	5412,5	6546,7
11,521416	5411,9	6522,9
11,534737	5419,2	6514,7
11,548053	5457,7	6521,6
11,56137	5467	6551,3
11,574687	5472,9	6584,3
11,588003	5485,5	6619,2
11,601324	5501,6	6591,1
11,61464	5520,2	6609,2
11,627965	5483,7	6678,2
11,641286	5480,4	6708,9
11,654606	5520	6788
11,667927	5513,7	6868,4
11,681252	5529,8	6884,2
11,694577	5475,5	6892,9

11,707898	5509,4	6911,9
11,721223	5522,1	6909,7
11,734544	5575,1	6833,9
11,74786	5587,3	6756,1
11,761181	5566,9	6811,2
11,774497	5522,4	6749,1
11,787813	5501	6773,5
11,801138	5485,6	6790,4
11,81445	5454	6809,4
11,827763	5454,4	6834,1
11,841074	5431,3	6893,9
11,854395	5414,8	6898,8
11,867711	5406,4	6926,1
11,881036	5405,7	6894,5
11,894357	5395,5	6962,3
11,907682	5414,6	6852,1
11,921003	5410,7	6737,6
11,934328	5423,7	6667,2
11,947644	5435,2	6674,2
11,960965	5401,6	6743,3
11,974286	5379,4	6792,8
11,987612	5375,8	6784,3
12,000937	5363,1	6840,4
12,014253	5343,1	6812,4
12,027574	5357,9	6776,9
12,04089	5336,6	6793,1
12,05419	5387,5	6785,4
12,067502	5363,9	6694,8
12,080832	5412	6588,7
12,094152	5481,5	6545,5
12,107473	5476,6	6416,1
12,120794	5432,7	6383
12,134119	5432,5	6393,1
12,147445	5421,9	6315,3
12,160764	5412,3	6313,9
12,174088	5392,2	6339,5
12,187413	5394,2	6370
12,200724	5369,5	6476,1
12,214041	5329,6	6557,2
12,227361	5313,2	6611
12,240682	5286,9	6650,8
12,253999	5302,6	6649,3
12,267315	5282,1	6634,6
12,280636	5307,5	6684,7
12,293969	5335,7	6693
12,307299	5320,7	6681,6
12,320619	5330,4	6608,1
12,333936	5367,6	6591,1
12,347265	5376,6	6580,2
12,36059	5396,7	6643,2
12,373902	5384,1	6714,9
12,387214	5407,4	6737,5

12,400539	5405,6	6754
12,413864	5451,2	6758,6
12,427181	5457,6	6753,5
12,440497	5461,3	6696,7
12,453817	5501,5	6744,7
12,467147	5444,7	6715,6
12,480469	5451,3	6705,7
12,493794	5485	6724,1
12,50711	5473,9	6703
12,520422	5428,7	6634,1
12,533739	5387,9	6642,6
12,547056	5350,1	6624,9
12,560381	5334,5	6587,2
12,573697	5310,3	6558,5
12,587018	5332,9	6597,8
12,600334	5360,6	6639
12,613655	5424,7	6646,9
12,626976	5451,7	6696,6
12,640296	5395,2	6680,2
12,653613	5364,4	6666,3
12,666929	5354,2	6634,7
12,680254	5392,4	6624
12,693579	5393	6636,6
12,706896	5402,3	6637
12,720212	5388,6	6635,5
12,733533	5485,4	6633,7
12,746849	5526,1	6597,2
12,760165	5552	6664,7
12,77349	5488,4	6619,4
12,786811	5417,1	6621,5
12,800127	5419,5	6631,3
12,813454	5464,3	6661,2
12,826783	5498,4	6652,8
12,840099	5498,2	6621,7
12,85342	5545,9	6618,1
12,866741	5498,3	6586,5
12,880066	5464,4	6656,4
12,893391	5475,6	6679,6
12,906703	5471,4	6707,5
12,920015	5511,2	6664,1
12,933336	5517	6624,5
12,946652	5496,4	6633,4
12,959978	5449,9	6660,4
12,973303	5434,8	6635,7
12,986619	5424,7	6656,2
12,99994	5375,6	6654
13,013261	5362,2	6626,6
13,026581	5342,4	6619,7
13,039902	5288,7	6622,2
13,053223	5219,8	6610,5
13,066539	5169,6	6664,1
13,079864	5160,3	6693

13,093193	5128,7	6690,3
13,10651	5103,1	6683,5
13,119826	5110,5	6668,5
13,133151	5117,9	6636
13,146467	5147,9	6688,2
13,159785	5171	6745,9
13,173101	5144,2	6757,8
13,186413	5136,3	6714,3
13,199738	5098,8	6731,5
13,213059	5129,1	6719,8
13,226375	5117,1	6648,9
13,239696	5078,1	6661,9
13,253012	5094,6	6653,9
13,266324	5063,6	6694,1
13,279641	5094,1	6709,3
13,292961	5079,9	6659,6
13,30628	5011,3	6636,6
13,319605	4980,6	6667,5
13,332921	4936,2	6602,1
13,346242	4916,3	6605,9
13,359567	4952,6	6613,8
13,372896	4946,4	6619,9
13,386217	4971,1	6620,1
13,399529	5018,7	6560,5
13,412841	5028,8	6607,9
13,426161	5008,5	6616,9
13,439201	4973,8	6629
13,45253	4967,2	6685,1
13,465851	4985,3	6690,6
13,479176	5018,9	6722,4
13,492505	5065,5	6774,8
13,50583	5071,9	6775,3
13,519142	5039,4	6757,7
13,532467	5022,3	6727,5
13,545796	5021,5	6741
13,559117	5012	6746,3
13,572433	4996,6	6731,8
13,585758	4965,6	6777,4
13,599079	5005,2	6812,6
13,6124	4977,5	6793,2
13,625716	4979,2	6784,1
13,639033	4967,2	6799,7
13,652353	4991,6	6806,5
13,665674	5007	6771,3
13,678986	4978	6665,6
13,692311	4985,8	6644,4
13,705636	4987,7	6633,9
13,718957	4981,3	6643,3
13,732278	4995,2	6648
13,745589	4974	6618,7
13,758906	4932,7	6626,9
13,772231	4945,4	6562,5

13,785543	4939,4	6625,1
13,798868	4950,1	6695,1
13,812189	4962,6	6745,5
13,825509	5004,1	6708,2
13,838836	5019	6705,5
13,852156	5026,9	6702
13,865468	5032	6714,5
13,878793	5041,1	6786
13,892109	5040,5	6825,7
13,905422	4996,8	6868
13,918734	5000,9	6791,4
13,932063	4945,7	6763,1
13,945354	4937,9	6813
13,95868	4964,9	6803,7
13,972005	4977,5	6857,4
13,985325	5016	6864,6
13,998646	5056,3	6968,8
14,011971	5042,2	6892,9
14,025292	5049,6	6875,3
14,038601	5003,8	6899,2
14,05193	4939,7	6938,6
14,065251	4857,8	6898,8
14,078571	4882,1	6887,1
14,091896	4889,5	6793,5
14,105221	4893	6758,4
14,118542	4908,6	6777,8
14,131863	4861,5	6744,3
14,145184	4842,3	6758,7
14,158513	4855,4	6787,9
14,171834	4878,1	6783,7
14,185129	4947,6	6762,7
14,198446	4927,6	6679,8
14,211762	4923	6636,9
14,225087	4921,6	6614,5
14,238407	4900,7	6682,3
14,251728	4903,1	6638,8
14,265045	4894,8	6615,5
14,27837	4878,6	6658
14,291695	4926,5	6699,7
14,305011	4886,4	6675,6
14,318323	4904,5	6638,6
14,331644	4905,1	6571
14,34496	4935,7	6562,2
14,358285	4951,4	6583,2
14,371601	4968,8	6573
14,384918	4932,7	6600,9
14,398234	4929	6658,7
14,411546	4959,6	6617,4
14,424871	4972,3	6548,6
14,438201	4987,6	6536,7
14,451517	4969,7	6590,8
14,464838	4990	6576,5

14,478158	5009,2	6530,5
14,491475	4950,2	6519,5
14,504795	4962,7	6496,8
14,518116	4933,5	6514,7
14,531437	4931,2	6563,5
14,544757	4930,2	6600,6
14,557827	4918,3	6506,6
14,571148	4921,3	6505,4
14,584468	4917	6474,5
14,597793	4951,7	6466,5
14,611114	4993,5	6421,2
14,62443	4984,6	6410,2
14,637747	4978,6	6416,7
14,651059	5006,8	6407,4
14,664388	4982,7	6413,9
14,677704	4980,7	6386,8
14,690674	4962,3	6368,6
14,703999	4942,5	6385,6
14,71732	4889,3	6443,8
14,730632	4874,7	6446,3
14,743953	4844,9	6401,6
14,757278	4847,3	6401
14,770594	4849,1	6401,8
14,783914	4860,4	6429,8
14,797235	4846,7	6393,3
14,810556	4890,8	6406,7
14,823877	4902,3	6365,3
14,837189	4904,5	6333,7
14,850509	4960,1	6302,8
14,863826	4971,8	6297,7
14,877142	4998,5	6258,6
14,890463	5043,9	6194,4
14,903783	5051,7	6202,7
14,917104	5036,6	6154,2
14,930421	5062,1	6084,1
14,943746	5078,9	6090
14,957071	5084,6	6117,3
14,970387	5090,1	6120,5
14,983703	5061,2	6161,7
14,997015	5045,8	6196,8
15,010336	5088,8	6218,2
15,023657	5066,4	6181,7
15,036983	5036,5	6196,9
15,050308	5010,6	6230,2
15,063624	4955,4	6232,7
15,076932	4931,8	6221,4
15,090257	4926,5	6248,1
15,103578	4927,9	6281,7
15,116894	4945,2	6304
15,130219	4932,5	6290,1
15,143536	4941,5	6313,7
15,156852	4953,4	6346,7

15,170177	4898,8	6346,6
15,183502	4911,5	6343,5
15,196818	4922,9	6382,6
15,210139	4917,5	6382,5
15,223464	4960,4	6363,1
15,236776	4984,9	6394,2
15,250101	4944,8	6386,7
15,263422	4934,5	6413
15,276738	4921,9	6376,3
15,290067	4906,2	6311,9
15,303397	4900,1	6326,6
15,316713	4895,1	6349,2
15,330038	4908,4	6363,2
15,343367	4946,5	6395,6
15,356692	4972,6	6426,2
15,370013	4953,5	6481,4
15,383329	4991,2	6478,8
15,396654	4989,4	6449
15,409984	4984,6	6514,3
15,423305	4964,7	6618,5
15,436621	4953,3	6600,7
15,449933	4935,9	6644,7
15,463254	4942,1	6631,4
15,47657	4924,2	6630,4
15,489891	4951,6	6652,7
15,503207	4918,8	6673,7
15,516528	4940,3	6685
15,529844	4952,9	6674,7
15,543165	4970,8	6652,9
15,55649	4986,7	6745,4
15,569806	4998,6	6781,3
15,583114	4969,5	6780,3
15,596434	4951,1	6764,1
15,609764	4954,9	6717,9
15,623089	4963,2	6659,8
15,636401	4954,1	6573,9
15,64973	4935,7	6595,8
15,663051	4902,1	6595,6
15,676372	4902,1	6544,7
15,689697	4891,9	6468,3
15,703017	4903,3	6449,5
15,716342	4928,1	6501,8
15,729663	4943,5	6487,1
15,742992	4926,2	6495,4
15,756313	4928	6476,4
15,769629	4929,5	6522,6
15,78295	4947,9	6497,7
15,796271	4951,7	6496,7
15,809596	4950	6465,6
15,822917	4909,3	6480,1
15,836233	4921,2	6480,2
15,849554	4901,9	6494,3

15,862879	4874,6	6538,4
15,876199	4832,4	6590,6
15,889511	4833,4	6633
15,902841	4855,3	6627
15,916166	4831,5	6602,1
15,929486	4807,2	6658,7
15,942807	4813,7	6695,8
15,956128	4824,3	6710,4
15,969457	4843,9	6710,8
15,982778	4861,7	6776,3
15,996094	4886,6	6869,9
16,009419	4866,9	6915,7
16,022744	4845,7	6902,8
16,036069	4856,8	6884,7
16,049386	4860,3	6858,4
16,062698	4854,8	6875
16,076018	4862,9	6801
16,089335	4881,1	6748,2
16,102656	4878,7	6660
16,115981	4878,5	6721,8
16,129297	4876,1	6736,2
16,142619	4872,8	6683,5
16,155944	4873,3	6616,7
16,169264	4921,8	6611,8
16,182585	4933	6616,7
16,19591	4918,3	6639,6
16,209231	4954,8	6635,5
16,222552	5007,8	6581,2
16,235877	4998,3	6562,2
16,249197	4961,2	6512,9
16,262514	4935,3	6442,7
16,27583	4938,8	6407,5
16,289155	4918,7	6437,5
16,30248	4950	6507,4
16,315801	4931,3	6519,3
16,329117	4954,6	6555,6
16,342429	4959,4	6508,7
16,355754	4962,9	6543,9
16,369079	4946,9	6528,3
16,382391	4914,3	6546,5
16,395712	4908,4	6535,3
16,409028	4888,5	6532,7
16,422353	4861,9	6523,8
16,435674	4839,8	6524,7
16,448995	4857,4	6468,3
16,462315	4871,2	6436,6
16,475627	4866	6414,4
16,488948	4837,1	6402,4
16,502278	4835,9	6456,8
16,515594	4863,1	6497,3
16,52891	4859,6	6463,4
16,542231	4849,7	6407,6

16,55556	4869,9	6425
16,568889	4871,7	6372,2
16,582202	4882,6	6333,7
16,595527	4884,3	6266,1
16,608852	4898	6294,5
16,622181	4891,6	6281,2
16,635502	4867,8	6318,1
16,648822	4879,1	6275
16,662139	4862,5	6281,1
16,675459	4890,4	6317,6
16,68878	4867,8	6306,8
16,702101	4896,1	6358
16,715417	4927,4	6303,9
16,728733	4926,3	6289,1
16,742054	4911,5	6317,5
16,755379	4934,7	6308,2
16,768704	4927,6	6281,6
16,782025	4925,3	6280,3
16,79535	4938,4	6245,3
16,808666	4943,3	6260,1
16,821987	4937,7	6255,5
16,835308	4974,6	6235,9
16,848624	5019,5	6253,7
16,86194	5043,3	6251,9
16,875265	5033,1	6279,5
16,888582	5062,7	6281,9
16,901898	5071	6252,3
16,915219	5093,2	6167,8
16,928544	5094,1	6111
16,941869	5090	6122,3
16,955194	5101,2	6087,5
16,968514	5038,2	6137,4
16,981835	5011,3	6162,1
16,995156	5016,1	6202,3
17,008485	5054,4	6202,4
17,021806	5032,8	6309,8
17,035131	5007,8	6305,9
17,048452	4987	6301,2
17,061777	4994,2	6265,6
17,075097	4992,7	6272,4
17,088409	5038,2	6275
17,101734	5050,1	6285,6
17,115055	5066,5	6272,1
17,12838	5092,6	6258,6
17,141701	5092,1	6254
17,155013	5054,2	6217,5
17,168334	5045,2	6245,3
17,181663	5030,3	6261,2
17,194979	5020	6266,5
17,208296	5013,8	6259,1
17,221616	5040,7	6232,4
17,234941	5110,8	6194,9

17,248266	5100,2	6181,7
17,261578	5124,2	6140,7
17,274903	5112,7	6148,1
17,288224	5064,7	6172,3
17,301549	5038,3	6156,8
17,314879	5024,6	6172,1
17,328204	5009,1	6207,9
17,34152	4992,5	6172,9
17,354836	4982,2	6174,9
17,368153	4972,6	6204,8
17,381473	4962,8	6226,8
17,39479	4964,7	6238
17,408102	5013,8	6236,9
17,421418	5004,5	6223,6
17,434743	4996,1	6274,4
17,448064	4935,1	6248,4
17,461389	4953,1	6268,1
17,474709	4969,8	6260,7
17,488026	4966,7	6147,4
17,501346	4992,6	6150,7
17,514667	4987,2	6204,6
17,527984	4967,7	6174
17,5413	4960,6	6178,7
17,554616	4980,3	6217,5
17,567933	4988,2	6201,7
17,581253	4999,4	6134,4
17,594571	4984,8	6178,4
17,607891	4977	6218,4
17,621203	4953,4	6301,4
17,634524	4939,5	6407,7
17,647853	4948,9	6447,9
17,661178	4929,9	6403,9
17,67449	4891,3	6410,5
17,687803	4839,8	6391,8
17,701114	4839,7	6344,7
17,714439	4829,7	6291,8
17,727764	4822,6	6249,2
17,741085	4808,5	6220,5
17,75441	4788,9	6252,5
17,767731	4763,1	6264,5
17,781052	4751,1	6254,5
17,794368	4750,6	6270,8
17,807693	4741,7	6294,2
17,821009	4735,9	6346,4
17,834321	4724,7	6345,5
17,847642	4722,9	6358,9
17,860963	4666,6	6366,2
17,874275	4698,7	6389,1
17,887596	4699,9	6421,6
17,900912	4701,1	6395,9
17,914233	4733,4	6420,6
17,927549	4735,7	6373,1

17,940869	4734,4	6312,8
17,95419	4805,6	6297,7
17,967507	4804,9	6316,4
17,980827	4810,2	6289,5
17,994139	4793	6311,6
18,007464	4783,1	6331,8
18,020781	4762,4	6348,2
18,034097	4768,8	6313,8
18,047426	4759,5	6314
18,060747	4724,8	6276,7
18,074063	4704,3	6303,8
18,087388	4712,5	6367,4
18,100714	4709,5	6455,2
18,114039	4720,7	6430,4
18,127355	4741,2	6364,9
18,14068	4714,8	6335,2
18,153996	4703,7	6274,9
18,167317	4711,3	6307,7
18,180637	4760,7	6315,1
18,193958	4728,3	6292,9
18,207283	4728,4	6278,5
18,220608	4738,5	6259,9
18,233925	4779,8	6302,1
18,247246	4769,7	6340,9
18,260571	4752,1	6364,5
18,273887	4780,2	6415,7
18,287199	4793,7	6447,2
18,300515	4773,1	6431,1
18,313832	4793,5	6409,6
18,327148	4787	6430,8
18,34046	4758,1	6448,3
18,353785	4787	6459,5
18,367101	4777,1	6463,4
18,380422	4731,8	6496,7
18,393738	4728,4	6552,9
18,40705	4747,8	6518,6
18,420375	4790,2	6476,5
18,433696	4811,4	6450
18,447026	4847,3	6432,2
18,460346	4869,2	6412,8
18,473662	4914	6394,5
18,486992	4942,4	6447,4
18,500317	4960,1	6432,1
18,513638	4934,1	6422,5
18,526954	4934,8	6433,7
18,540275	4927,5	6490,6
18,5536	4914	6490,4
18,566925	4875,6	6475,3
18,580241	4863,2	6495
18,593562	4878,1	6547,5
18,606883	4872	6559,7
18,620108	4857,8	6540,7

18,633428	4867,3	6574
18,646745	4859,6	6538,9
18,660066	4855,7	6552,3
18,673378	4873,5	6583,2
18,686694	4885,5	6595,1
18,700014	4844,8	6607,4
18,713344	4818,3	6592,2
18,726665	4832,6	6595,8
18,739985	4832,5	6568,7
18,753302	4845,4	6570,7
18,766627	4854,3	6592,8
18,779947	4831,8	6595,5
18,793268	4847,2	6574,4
18,80658	4835,9	6574,8
18,819905	4830	6558,3
18,833226	4806,3	6507,2
18,846542	4776,7	6467,1
18,859863	4773,1	6397,7
18,873175	4776,7	6390,9
18,886496	4810,4	6391,5
18,899808	4805,8	6346,6
18,913137	4820,7	6337,8
18,926466	4821,2	6333,1
18,939791	4817,8	6343
18,953108	4797	6379,5
18,966424	4792,8	6435,4
18,979749	4828,3	6456,8
18,99307	4823,4	6433,2
19,006382	4823,9	6406,9
19,019703	4868,8	6395,3
19,033027	4846,8	6415,2
19,046353	4865,1	6430,4
19,059669	4856,5	6399,6
19,072989	4877,3	6311,7
19,086319	4873,8	6315,6
19,099639	4868	6345
19,11296	4893,9	6348,2
19,126285	4870,6	6354,4
19,13961	4877,8	6337,9
19,152931	4853,3	6274,6
19,166252	4851,6	6314,8
19,179577	4811,4	6269,5
19,192898	4827,4	6277,4
19,206214	4822,2	6274,3
19,219526	4811,2	6289,7
19,232851	4780,4	6279
19,246171	4810,4	6252
19,259488	4775	6200,9
19,2728	4759,1	6207,6
19,286129	4746,7	6245,7
19,29945	4751	6268,6
19,312762	4722,3	6295,4

19,326083	4713,6	6342,5
19,339403	4711,3	6409,3
19,35272	4751,8	6427,4
19,366036	4799	6438,8
19,379348	4774	6394,5
19,392418	4789,3	6386,7
19,405734	4794,7	6398,1
19,419054	4769,4	6383,7
19,432366	4747,5	6405,2
19,445691	4722,9	6362,6
19,459017	4717,7	6313,1
19,472342	4738,3	6267,2
19,485658	4767	6255,8
19,498983	4754,6	6236,6
19,512308	4754	6270,9
19,525633	4779,8	6307,4
19,538949	4834,6	6326,1
19,552261	4842,9	6399,1
19,565586	4828,6	6425,5
19,578911	4818	6454,7
19,592033	4790,7	6404,8
19,605362	4791,4	6436,4
19,618687	4786,7	6427,8
19,632004	4767,9	6479,3
19,645333	4761,5	6435,3
19,658662	4730,9	6507,4
19,671979	4701	6496,3
19,685295	4697,5	6476,1
19,69862	4674,7	6473
19,711945	4635	6446,6
19,72527	4595,3	6471,3
19,738586	4594,2	6475,4
19,751907	4556,3	6564,5
19,765223	4553,3	6548
19,778548	4574,3	6551,5
19,791861	4594,6	6596,9
19,805181	4596,3	6588,8
19,818506	4609,7	6635,8
19,831827	4600,3	6684,9
19,845147	4605,6	6654,8
19,858473	4616,6	6653,3
19,871793	4618,4	6642,7
19,88511	4613,1	6662,5
19,89843	4617,1	6646,5
19,91176	4613	6603,8
19,925085	4593,1	6595
19,938401	4605,3	6605,2
19,951722	4621,6	6546,2
19,965038	4626,8	6448,9
19,978363	4636,1	6401,3
19,991679	4643,2	6387,1
20,004996	4615,1	6434,3

20,018317	4611	6492,2
20,031642	4613,9	6492,9
20,044962	4591,8	6495,3
20,058274	4584,9	6599
20,071595	4581,4	6586,9
20,084911	4548,3	6646
20,098228	4535	6671,4
20,111544	4510,7	6664,2
20,12486	4508,4	6687,2
20,138177	4527,5	6677,9
20,151502	4529,8	6698,8
20,164827	4546,6	6715,7
20,178148	4539,1	6721,2
20,191152	4525,2	6700,6
20,204468	4528,1	6748,3
20,217797	4535,7	6690,1
20,231123	4538	6725,8
20,244435	4563,7	6764,2
20,25776	4570,8	6747,6
20,27108	4596,4	6751,7
20,284405	4606,4	6702,2
20,297722	4569,6	6733,5
20,311034	4571,3	6745,7
20,324359	4565,5	6687,4
20,337684	4549,2	6685,8
20,351004	4547,4	6666,2
20,364316	4535,2	6654,5
20,377637	4536,4	6670,9
20,390958	4549,7	6722,1
20,404279	4564,1	6674
20,417599	4589,6	6658,5
20,43092	4599,5	6635,8
20,444241	4586,6	6618,6
20,457566	4566,8	6642,2
20,470886	4567,4	6672,9
20,484207	4558,1	6683,8
20,497528	4553	6648,5
20,510844	4569,3	6671
20,524169	4571,6	6694,1
20,537486	4547,1	6704,2
20,550806	4581,4	6699,1
20,564127	4608,7	6704,7
20,577456	4606,3	6641,6
20,590777	4628,5	6571,4
20,604098	4653,5	6555,4
20,617422	4667	6532,1
20,630752	4623,3	6602,4
20,644073	4603,4	6638,3
20,657389	4585,9	6634,6
20,670701	4599,8	6668,6
20,684026	4580,7	6612,1
20,697351	4572,5	6639,5

20,710672	4567,9	6658,1
20,723997	4578,4	6690,6
20,737322	4638,9	6663,7
20,750643	4628,4	6685,9
20,763963	4620,7	6680,6
20,777284	4637,1	6729,6
20,790604	4624,5	6755,9
20,803925	4651,9	6731,9
20,817242	4655,4	6708,1
20,830567	4685,8	6729,4
20,843887	4698,1	6679,4
20,857208	4711,7	6643,9
20,870524	4720,7	6671,8
20,883845	4701,2	6702,4
20,897161	4690,7	6663,3
20,910478	4629,2	6687,2
20,923803	4674	6698,8
20,937128	4658,7	6662,7
20,950444	4660,9	6645
20,963756	4650,9	6651,9
20,977072	4631,6	6646,9
20,990389	4636,9	6656,7
21,003705	4615,3	6619,1
21,01703	4593,3	6636,2
21,030347	4550,8	6670,9
21,043663	4550,7	6695,4
21,056975	4558,1	6716,4
21,0703	4535,5	6742,8
21,083629	4518,7	6706,5
21,09695	4513,6	6704,7
21,110271	4554,1	6705,9
21,123591	4538,5	6754
21,136912	4561,9	6770,6
21,150229	4545,6	6777,5
21,163541	4559,6	6805,5
21,176866	4578,8	6784,8
21,190182	4602,2	6768,5
21,203502	4581,7	6791,4
21,216823	4550,4	6792,5
21,230148	4562,6	6820,1
21,243465	4547,5	6836,7
21,256785	4540	6918,2
21,27011	4518,4	6913,6
21,283436	4533,5	6946,3
21,296752	4548	6942,9
21,310064	4523	6921,4
21,323393	4524,1	6899,8
21,336718	4565,3	6904,5
21,350039	4617,1	6850,9
21,363351	4630,1	6824,6
21,376672	4625	6790,9
21,389992	4610,4	6768,5

21,403313	4584,8	6788,3
21,416638	4624,6	6780,7
21,429963	4629,4	6779,3
21,443288	4648,7	6750,4
21,456604	4650,4	6713,8
21,469929	4652,1	6768,1
21,483246	4633,8	6750,1
21,496562	4589,4	6786,4
21,509879	4567,3	6767,8
21,523204	4601,1	6704,8
21,536533	4611,3	6588,5
21,549854	4600,2	6589,9
21,56317	4617	6581,7
21,576491	4644,9	6573,4
21,58982	4625,1	6540
21,602837	4610,5	6556,5
21,616154	4607,4	6589,7
21,629474	4609,7	6533,9
21,642795	4598,6	6471,6
21,656124	4553,3	6430,8
21,669445	4534,8	6471,2
21,682761	4556,8	6414,4
21,696082	4557,9	6394,8
21,709407	4557,9	6448,1
21,722728	4557,3	6485,2
21,73604	4539,4	6498,1
21,749356	4562,6	6524,3
21,762677	4588,6	6494,1
21,776002	4588,7	6527,5
21,789314	4547,6	6512,7
21,802636	4536,6	6459
21,815952	4497,8	6474,9
21,829264	4503	6432,6
21,842581	4484	6427,3
21,855897	4481,6	6432,1
21,869218	4460,2	6421,8
21,882534	4488,5	6358,3
21,89585	4511,6	6341,2
21,909158	4542,2	6330
21,922483	4564,2	6315,3
21,935812	4579,4	6321,7
21,949129	4603,3	6273,2
21,962454	4573,1	6266
21,975774	4577,7	6270,8
21,989095	4591,7	6227,8
22,002411	4624,3	6216,9
22,015724	4612	6188,4
22,02904	4657,9	6155
22,042356	4623	6133,2
22,055673	4643,3	6101,5
22,068993	4653,2	6093,6
22,08231	4608,4	6042,4

22,095622	4607,8	6059
22,108947	4632,8	6099,5
22,122272	4631	6096,5
22,135593	4580	6166,6
22,148909	4537,6	6190,3
22,162221	4508,7	6180,7
22,175546	4515	6156,7
22,188866	4513,9	6155,7
22,202191	4515,6	6166,3
22,215512	4479,7	6177,8
22,228837	4447,5	6191,7
22,242162	4418,2	6212,3
22,255483	4409,5	6185,1
22,268799	4412,4	6179,8
22,282116	4411,9	6219,2
22,295436	4408,4	6238,4
22,308757	4413,6	6218,3
22,322073	4413,7	6230,4
22,335398	4436,1	6204,7
22,348715	4435,6	6149,5
22,362031	4464,4	6181
22,375361	4449,4	6183,1
22,388681	4462,1	6143,7
22,401993	4471,4	6143,7
22,415305	4480,6	6181
22,42863	4490,5	6191,7
22,441951	4496,3	6202,7
22,455276	4506,1	6220,1
22,468597	4525,7	6247,3
22,481917	4538,5	6264,6
22,495234	4547,8	6273,6
22,50855	4528,7	6283,8
22,521875	4548,4	6286,4
22,535191	4572,7	6312,1
22,548508	4569,9	6304
22,561828	4625,7	6306,4
22,575154	4577,5	6278,8
22,588479	4554,2	6230,5
22,601795	4557,7	6216,6
22,615107	4538,6	6246,9
22,628436	4533,3	6245,7
22,641757	4495,1	6222,7
22,655078	4507,9	6200,9
22,668394	4490,5	6242,2
22,681715	4480,6	6247,5
22,695035	4485,8	6250,7
22,70836	4481,2	6277,5
22,721681	4478,4	6237,1
22,734997	4477,7	6235
22,748314	4481,2	6214,2
22,761639	4448,8	6214,1
22,774964	4436,7	6183,4

22,788284	4394,7	6212,3
22,801601	4353,4	6247,6
22,814917	4355,7	6188,9
22,828247	4355,1	6176,9
22,841572	4353,4	6195,6
22,854888	4346,5	6213
22,8682	4371,1	6171,5
22,881525	4413,1	6224,2
22,89485	4403,6	6205,5
22,908162	4368,8	6170,9
22,921474	4405,6	6253,1
22,934799	4378	6297,4
22,94812	4380,9	6253,4
22,961441	4382	6245,5
22,974757	4387,7	6197,6
22,988073	4376,8	6220,4
23,001394	4386	6253,8
23,014715	4388,8	6259,4
23,027927	4421,5	6266,6
23,041248	4460,1	6241,1
23,054577	4442,8	6217,7
23,067893	4460,1	6168,8
23,081106	4469,9	6156,9
23,094431	4486,7	6206,3
23,107756	4454,4	6200,4
23,121072	4427,9	6195
23,134393	4439,3	6204,3
23,147714	4481,3	6203,7
23,161047	4461,8	6165,5
23,174372	4458,3	6150,1
23,187684	4455,5	6168
23,201005	4423,9	6144,3
23,214334	4427,9	6115,7
23,227664	4423,9	6131,8
23,24098	4424,4	6137,5
23,254296	4450,4	6136,3
23,267617	4449,3	6120,3
23,280938	4440,6	6113,6
23,294258	4440,1	6134,9
23,307579	4423,4	6112
23,320904	4439,5	6123,6
23,334225	4431,4	6074
23,347546	4400,3	6073,9
23,360858	4376,3	6062
23,374178	4360,2	6028,6
23,387499	4368,3	6017,8
23,400807	4382	6013,8
23,414127	4406,7	6037,7
23,427444	4418,2	6014,4
23,44076	4427,4	6022,6
23,454085	4427,4	6096,4
23,467406	4429,1	6090,9

23,480726	4426,8	6081,1
23,494047	4423,4	6071,5
23,507372	4396,3	6078,8
23,520701	4380,9	6092,9
23,534026	4347,6	6101,5
23,547339	4348,8	6089,7
23,560655	4335,6	6081
23,573984	4329,3	6053,9
23,587301	4341,3	6022,7
23,600621	4340,2	6027,9
23,613942	4323,6	6034,6
23,627263	4323	5977,8
23,640583	4331	5938,9
23,653904	4316,7	5923,8
23,667229	4315,6	5929,2
23,68055	4314,5	5961,4
23,693866	4311,6	5982,5
23,707182	4306,5	5995,5
23,720503	4298,5	6090,2
23,733819	4310,5	6063,6
23,747136	4299,1	6108,3
23,760461	4312,7	6106,4
23,773786	4309,9	6095,2
23,787111	4288,2	6088,6
23,800432	4267,1	6059,7
23,813748	4280,2	6041,8
23,826757	4280,8	6049,7
23,840086	4267,1	6020
23,853407	4267,1	6000,8
23,866723	4288,2	6040,1
23,880053	4300,2	5965,1
23,893377	4317,9	5942,2
23,906698	4340,7	5936,3
23,919945	4336,7	6020,9
23,933262	4323,6	6009
23,946578	4297,9	6016,3
23,959894	4280,8	6018,8
23,973219	4280,2	6051
23,986544	4251,8	6107,2
23,999865	4259,7	6133,5
24,013186	4249	6125,6
24,026506	4276,8	6147,4
24,039827	4239,9	6122,3
24,053148	4225,7	6117,9
24,06646	4196,3	6112,7
24,079776	4161,3	6141,8
24,093101	4132,5	6181,9
24,106417	4088,7	6210,4
24,119734	4088,1	6230,5
24,133054	4075,7	6257,2
24,146367	4126,9	6313,7
24,159687	4137	6339,8

24,173008	4132,5	6314,2
24,186324	4133,1	6304,9
24,199645	4115,6	6308,2
24,212957	4119	6280,1
24,226282	4125,2	6258
24,239598	4105,6	6255,9
24,252915	4132,6	6199,7
24,266236	4132,1	6144,8
24,279552	4132,1	6165,5
24,292877	4093,9	6180,4
24,306202	4098,4	6228,5
24,319518	4104,6	6302,8
24,332834	4083,3	6350,7
24,346155	4056,9	6387,5
24,359485	4035,1	6384,1
24,372805	4025	6364,6
24,386122	4004,8	6347,9
24,399438	3985,9	6382,4
24,412754	4004,8	6417
24,426084	3985,8	6385,1
24,439404	4000,3	6350,1
24,452721	4037,2	6313
24,466046	4042,3	6214,1
24,479371	4002	6183,2
24,492691	4018,2	6183,3
24,506004	3976,8	6164
24,51932	3962,8	6181,6
24,532645	3961,8	6243,9
24,545675	3963,4	6283,7
24,558992	3969,7	6312,2
24,572312	3988,1	6308,1
24,585624	4015	6295
24,598945	4010,5	6298,4
24,612261	4016,1	6282,3
24,625573	4000,5	6275,9
24,638903	4004,9	6268,3
24,652223	3993,2	6262
24,665544	3989,5	6245,1
24,678865	3980,5	6203,8
24,69219	3997,8	6152,1
24,705506	4034,1	6094,7
24,718823	4021,9	6105,3
24,732143	4014,6	6099,4
24,74546	4015,2	6115,3
24,758776	4036,5	6157,4
24,77211	4048,2	6162,8
24,785435	4035,4	6138,5
24,798755	4018,6	6165,4
24,812072	4025,8	6181,6
24,825401	4010,8	6157,2
24,838726	3992,9	6161,2
24,852042	3981,7	6163,9

24,865359	3961,1	6194
24,878688	3945,6	6215,1
24,892013	3937,2	6246,2
24,905334	3919,9	6192,5
24,918646	3913,8	6158,4
24,931967	3906,1	6096,9
24,945292	3915,5	6082
24,958612	3885,5	6087,3
24,971933	3880,5	6086,6
24,985254	3872,7	6060,1
24,998574	3876	6067,4
25,011895	3849,6	6110,8
25,025216	3866,9	6156,1
25,038536	3842,6	6155,4
25,051857	3801,2	6194
25,065178	3810,6	6274,1
25,078499	3829,8	6324
25,091815	3796,6	6333,9
25,105131	3780,1	6402,2
25,118452	3792,8	6413,6
25,131768	3789,4	6413,1
25,145089	3794,9	6366,9
25,15841	3789,4	6352,9
25,17173	3814,8	6347,9
25,185047	3839,6	6355,1
25,198372	3838	6354,7
25,211693	3829,6	6322,5
25,225009	3835,7	6342,3
25,238325	3824,7	6288,6
25,251646	3831,8	6248,9
25,264971	3824,1	6194,5
25,278296	3808,6	6170,5
25,291621	3816,3	6196,3
25,304937	3806,4	6206,2
25,318258	3789,2	6262,8
25,33157	3771,6	6289,1
25,344895	3752,3	6286,3
25,358211	3745,2	6247,3
25,371532	3769	6284,2
25,384857	3781,2	6290,7
25,398173	3794,9	6334,2
25,41149	3774,5	6353,6
25,424811	3790,5	6378,5
25,438131	3787,3	6418,4
25,451456	3781,2	6382,2
25,464772	3782,9	6384,2
25,478089	3777	6399,7
25,491418	3760,6	6369,1
25,504743	3749,6	6350,3
25,51806	3765	6334,4
25,531372	3750,6	6368,8
25,544693	3746,5	6353

25,558013	3727,9	6334,5
25,571338	3710,5	6302,1
25,58465	3708,3	6286,1
25,597975	3705,2	6242,8
25,611296	3708,4	6249,3
25,624621	3713,3	6255,2
25,637933	3721,4	6189,9
25,651254	3709,8	6208,3
25,664579	3707	6262,9
25,677904	3699,7	6299
25,691216	3700,2	6276,4
25,704532	3701,3	6290
25,717848	3700,9	6291,9
25,731178	3701,9	6289,9
25,744498	3714,1	6240,2
25,757819	3728,9	6241,2
25,771136	3745,5	6246,5
25,784461	3746,5	6221,9
25,797786	3749,9	6248,5
25,811102	3759,8	6208,1
25,824409	3772,9	6200,3
25,837734	3792,7	6226,4
25,851055	3790	6225
25,864376	3766,2	6215
25,877692	3776,6	6193,1
25,891013	3755,7	6189,1
25,904329	3731,5	6144,3
25,91765	3709	6120,6
25,930966	3724,4	6117,3
25,944287	3730,4	6110,4
25,957603	3756,8	6108,4
25,970924	3772,1	6163
25,984249	3796,3	6117,7
25,99757	3785,3	6123,8
26,010891	3800,2	6114,1
26,024211	3809,5	6141,3
26,037536	3799,6	6143,2
26,050861	3802,4	6124,4
26,064177	3792,1	6076,1
26,07749	3785,7	6062,3
26,090819	3801,4	6032
26,10414	3795,9	6026,1
26,117456	3797	5971
26,130777	3784,3	5981,3
26,144098	3798,6	5996,9
26,157418	3781,6	5999,4
26,170739	3768,4	6023,2
26,184064	3764,5	6013,3
26,197384	3770,6	6019,4
26,210709	3771	6047
26,22403	3784,3	6057,3
26,237342	3778,4	6053,2

26,250663	3776,2	6094,7
26,263984	3760,3	6074,3
26,277309	3765,7	6092,1
26,290634	3770,7	6031
26,30395	3767,8	5938
26,317266	3757,9	5870,8
26,330596	3750,2	5880,6
26,343925	3757,9	5892,8
26,357246	3737	5912,4
26,370566	3707,9	5962,4
26,383761	3693,2	5943,2

Supplementary Figure 8 Immunoblotting for Lasp1 and GFP expression in *Lasp1* $^{-/-}$ MEFs

Lasp1 expression

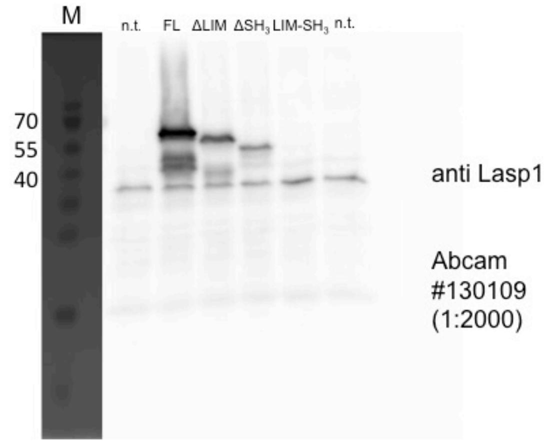

M = marker

27.07.16

GFP expression

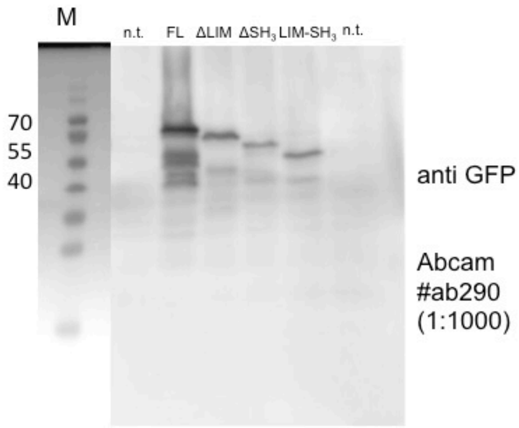

M = marker

28.07.16

Supplementary Figure 9a Histomorphometric analysis of total cartilage area (%)

wt	*Lasp1* $^{-/-}$	hTNFtg	hTNFtg/*Lasp1* $^{-/-}$
3,72	7,08	1,92	5,5
3,22	8,28	1,92	7,3
2,88	5,14	2,52	5,0
6,04	6,25	2,91	4,88
5,5	4,69	2,81	2,98
6,29	5,3	2,4	4,51
5,38	8,12	2,26	5,38
6,07	4,23	3,05	3,63
7,6		4,77	3,96
6,4		5,57	3,3

Supplementary Figure 9b Evaluation of MMP3 levels following TNF stimulation (pg/ml)

wt FLS	*Lasp1-/-* FLS	wt FLS [mTNF]	*Lasp1-/-* FLS [mTNF]
4,09556853	5,2190799	51,08357643	54,87202918
3,84724792	5,10346144	46,72282716	51,37359542
4,33003507	5,65217382	41,73963148	73,16331138
2,99804245	3,33564844	31,04574488	91,91632913
3,14097006	3,26472744	144,562252	123,4425795

Supplementary Figure 9c **Evaluation of MMP9 levels following TNFa stimulation (pg/ml)**

wt FLS	*Lasp1-/-* FLS	wt FLS [mTNF]	*Lasp1-/-* FLS [mTNF]
0,0205	0,0192	0,4158	0,2941
0,0264	0,0189	0,2393	0,1612
0,0213	0,0317	0,2279	0,5009

Supplementary Figure 10 FACS analysis (%)

PDPN+ CD90-

hTNFtg	hTNFtg/*Lasp1* $^{-/-}$
14,2	21,8
12,4	21,6

PDPN+ CD90+

hTNFtg	hTNFtg/*Lasp1* $^{-/-}$
37,6	45,5
30,5	39,9

PDPN- CD90+

hTNFtg	hTNFtg/*Lasp1* $^{-/-}$
13,1	8,24
15,5	11,5

Supplementary Figure 11 Immunoblotting for Src and AKT expression after IL1ß stimulation

pSrc expression IL1ß stimulation

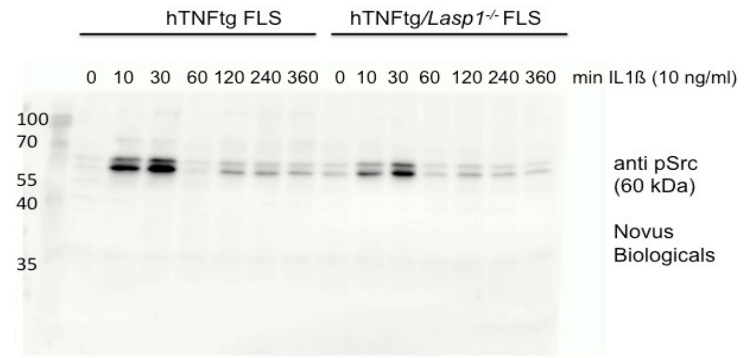

08.06.16

Src expression IL1ß stimulation

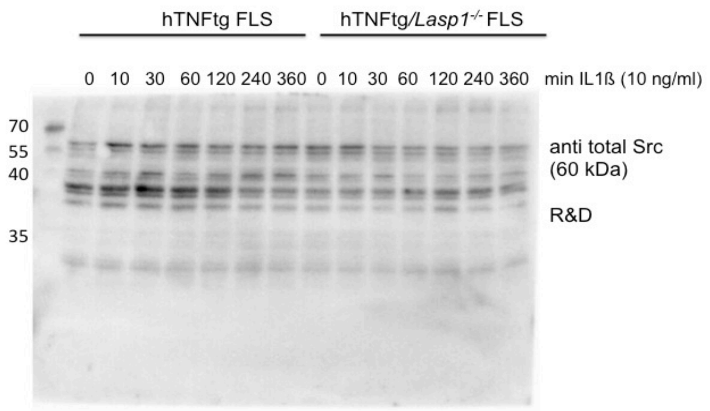

09.06.16

pAKT expression IL1ß stimulation

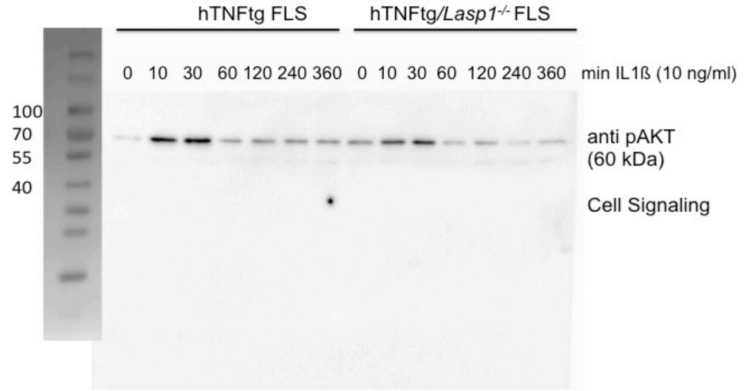

13.07.17

AKT expression IL1ß stimulation

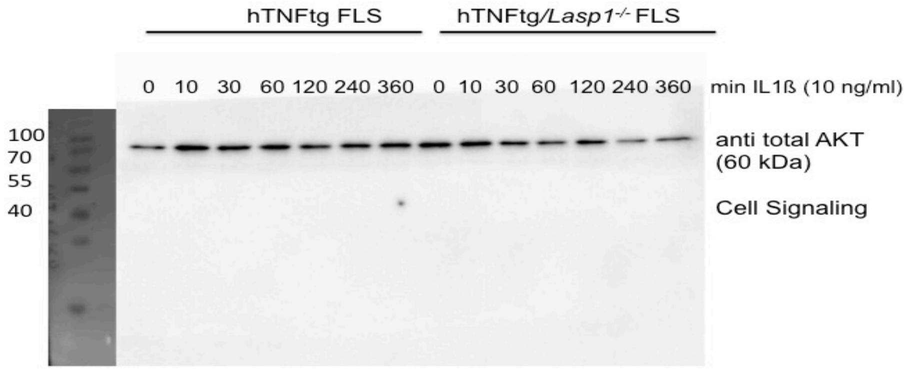

14.07.17

Supplementary Table 1 Primers for genotyping

Genotype	Forward primer sequence (5'-3')	Reverse primer sequence (5'-3')
Lasp1-/-	CACACTCGCGTCTGTTTCTCCAGC	GCACCTCTAACCTCCTGCACACACC

PCR conditions
95°C 1 min
35x (68°C 1 min, 72°C 2 min)
72°C 6 min
10°C ∞

Genotype	Forward primer sequence (5'-3')	Reverse primer sequence (5'-3')
**hTNFtg**	TACCCCCTCCTTCAGACACC	GCCCTTCATAATATCCCCCA
**hTNFtg wt**	GAGGGCCGAAGCTGCGGCTGGGT	GGTGGCGATTGGCTTGGCGAG

PCR conditions
95°C 30 s
35x (58.5°C 30 s, 72°C 45 s)
72°C 5 min
10°C ∞

Supplementary Table 2 Primers for sequencing

DMR	PCR target
**1**	chr11:97,831,969-97,832,197
**2**	chr11:97,805,999-97,806,286

DMR	Forward primer sequence (5'-3')	Reverse primer sequence (5'-3')
**1**	cttgcttcctggcacgagAGGGAGAGAGAGGTTTGATTTTATT	caggaaacagctatgacCCCCACCCTTAACATTCTCTATAC
**2**	cttgcttcctggcacgagTGTTAGGGGTAGAGGATTTTTTATG	caggaaacagctatgacTATATACCTCAACCACACCCATATC

1st PCR conditions
95°C 15 min
40x (Ta 61°C, C95°C 30 s, 72°C 1 min)
72°C 10 min

2nd PCR conditions
95°C 15 min
35x (Ta 60°C, 95°C 30 s, 72°C 1 min)
72°C 10 min

Ta = annealing temperature

Supplementary Table 3 Primers for Sequences used for Quantitative Real-Time PCR

Gene	Primer sequence (5'-3')
m_Lasp1_for	TCT CCG CCT CAA GCA ACAG
m_Lasp1_rev	CTG ATC TGG TC CTG GGT CTC C
m_GAPDH_for	AGC AAG GAC ACT GAG CAA GAG AGG
m_GAPDH_rev	GGG TCT GGG ATG GAA ATT GTG AGG
m_Hprt_for	AGC TAC TGT AAT GAT CAG TCAA CG
m_Hprt_rev	AGA GGT CCT TTT CAC CAG CA

qPCR conditions
95°C 3 min
40x (95°C 30 s, 72°C 45 s)
40x (C95°C 30 s, 72°C 1 min)
Melting curve

for = forward
rev = reverse
